# Stepwise and reversible assembly of [2Fe–2S] rhombs to [8Fe–8S] clusters and their topological interconversions

**DOI:** 10.1038/s41557-025-01895-9

**Published:** 2025-08-20

**Authors:** Liam Grunwald, Micha L. Weber, Henrik Seng, Martin Clémancey, Patrick Dubourdeaux, Hongxin Wang, Michael Wörle, Yoshitaka Yoda, Stephen P. Cramer, Geneviève Blondin, Victor Mougel

**Affiliations:** 1 Department of Chemistry and Applied Biosciences (D-CHAB), Swiss Federal Institute of Technology Zürich (ETHZ), Zürich, Switzerland; 2 University of Grenoble Alpes, CNRS, Commissariat a l’energie atomique et aux energies alternatives (CEA), Institut de Recherche Interdisciplinaire de Grenoble (IRIG), Laboratoire de Chimie et Biologie des Metaux (LCBM), Physicochimie des Metaux en Biologie (PMB), CEA Grenoble, Grenoble, France; 3 SETI Institute, Mountain View, CA USA; 4 Precision Spectroscopy Division, SPring-8/JASRI, Sayo, Japan

**Keywords:** Inorganic chemistry, Coordination chemistry

## Abstract

Among all enzymatic metallocofactors, those found in nitrogenases, the P and L or M clusters, stand out for their intricate structures. They are assembled by proteins of the Nif gene cluster from Fe_2_S_2_ rhombs—the smallest building blocks in FeS cluster chemistry—through a sequence of reactions constructing a Fe_8_S_8_ precursor. To advance our understanding of how enzymes selectively build such elaborate inorganic molecules, here we parallel the biosynthetic pathway by reporting the rational stepwise assembly of [Fe_8_S_8_]^*m+*^ (*m* = 2, 4, 6) clusters from [Fe_2_S_2_]^2+^ rhombs within an extensive cyclic synthetic network. A [Fe_8_S_8_]^4+^ cluster of unique topology is identified, for which we coin the term ‘interlocked’ double cubane. As a molecular analogue of the NifB K cluster, a proposed precursor to both the P and L or M clusters, its preparation and the characterization of all related intermediates, offers fundamental insights into the molecular mechanisms governing the assembly of both biogenic and synthetic FeS clusters.

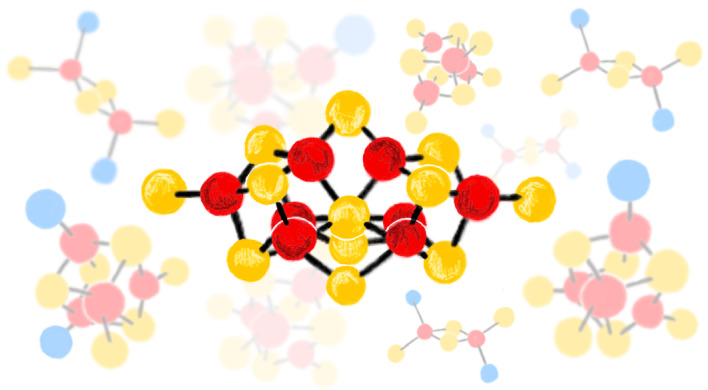

## Abstract (web summary)

Elucidating the nature of the metallocofactors in nitrogenase enzymes, and preparing synthetic analogues of these clusters, is a classic target for bioinorganic chemists. Now the transformation of [Fe_2_S_2_]^2+^ rhombs to [Fe_8_S_8_]^*n*+^ clusters has been achieved through a series of redox- and ligand-substitution reactions.

## Main

**Fig. 1:**
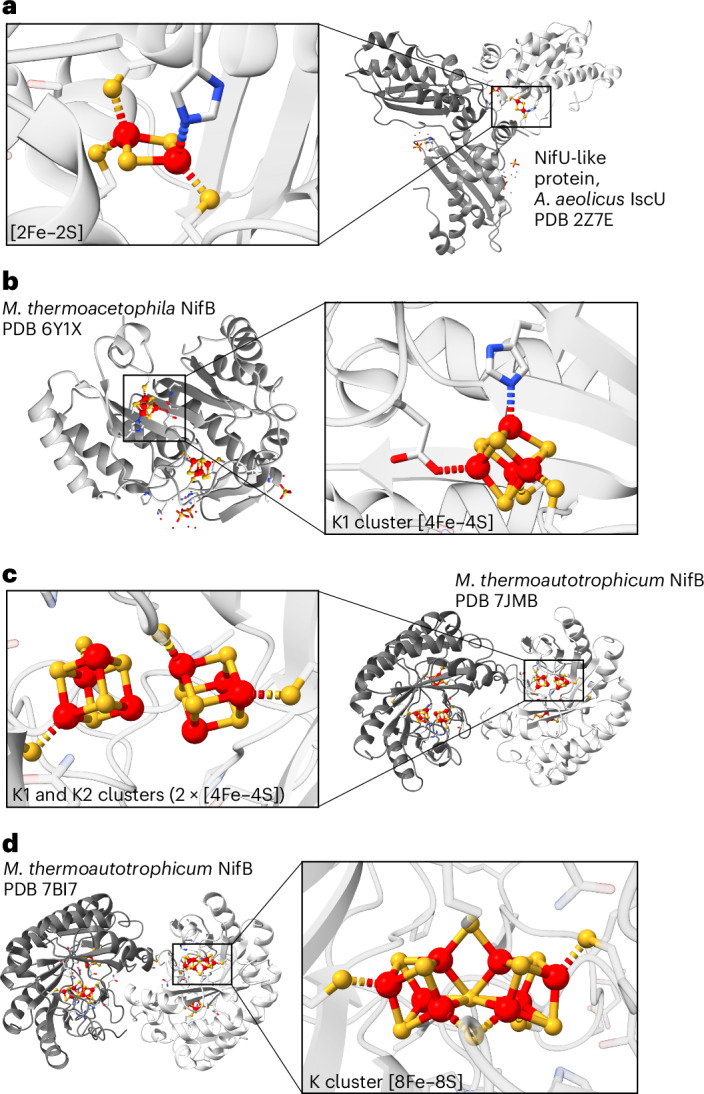
Selected crystal structures of enzymes involved in M-cluster maturation. **a**, Crystal structure of the NifU-like protein, *Aquifex aeolicus* IscU with a bound [2Fe–2S] cluster (Protein Data Bank (PDB) ID 2Z7E)^13^. A close-up view of the metallocofactor is shown as well. **b**, Crystal structure of *Methanotrix thermoacetophila* NifB with bound radical SAM and K1 clusters (PDB 6Y1X)^14^. A close-up view of the K1 cluster is shown alongside. **c**,**d**, Crystal structure refinements of *Methanobacterium thermoautotrophicum* NifB with a full complement of FeS cofactors, as proposed by ref. ^15^ (**c**; PDB 7JMB) and ref. ^16^ (**d**; PDB 7BI7). An alternative view of the [8Fe–8S] K cluster (**d**) is presented in Supplementary Fig. 97. For each solution of the structure, a close-up view of the K1 and K2 clusters (**c**) and the K cluster (**d**) are provided.

**Fig. 2:**
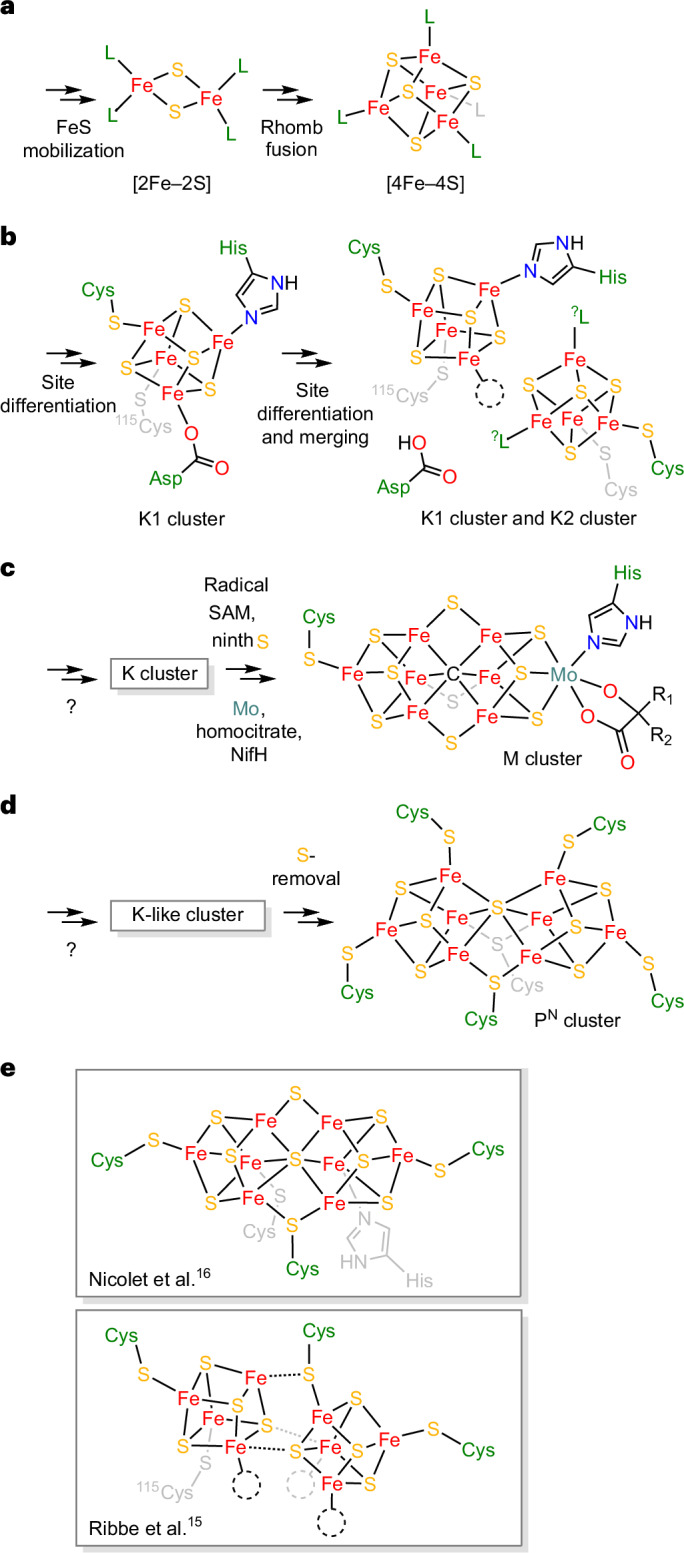
Scheme illustrating the FeS cluster conversions required for P- and M-cluster maturation and the central role of a K-cluster intermediate. **a**, Conversion of Fe and S into Fe_2_S_2_ clusters in NifU and NifS and their reductive fusion to Fe_4_S_4_ clusters. Here the arbitrary ligand, L, is usually a cysteinate or histidine residue, but may also refer to any other amino acid able and known to bind to Fe_4_S_4_ cofactors, for example, aspartate. **b**, Site differentiation and spatial merging of Fe_4_S_4_ clusters to form the K1 and K2 clusters in NifB. **c**, Last steps of M-cluster maturation from the Fe_8_S_8_ K-cluster intermediate, encompassing installation of the additional carbide and sulfide ligands, as well as the Mo ion. **d**, A hypothetically simple route from a K- (or K-like-) cluster precursor to the P^N^cluster via S(*µ*^2^) removal. **e**, Structures of the NifB K cluster proposed in refs. ^16^ and ^15^.

Iron–sulfur (FeS) clusters function as enzymatic metallocofactors in many critical metabolic processes^1,2^. While most of them are bi-, tri- or tetranuclear, larger assemblies are rare and have only been structurally characterized within two families: as *μ*^2^-sulfido-bridged double-cubane-type clusters in the aptly named double-cubane cluster protein^3,4^ and, more prominently, as the octanuclear P, L and M clusters found in nitrogenases and nitrogenase-like enzymes^5–8^. The biosyntheses of these clusters, particularly nitrogenases, are intricate and proceed through distinct yet mechanistically related pathways. Even though the P and M clusters mature separately from one another, both are assembled by proteins encoded in the *Nif* gene cluster through the intermediacy of a Fe_8_S_8_ precursor^7–10^; in a first step, NifS and NifU mobilize Fe and S to produce Fe_2_S_2_ rhombs, which are reductively combined to Fe_4_S_4_ cubanes^7,8,11–13^ (Figs. 1a and 2a). Towards the M cluster, two of the Fe_4_S_4_ cubanes are recruited by NifB, site differentiated^14^ (Figs. 1b and 2b), merged (Fig. 2b) and fused to form the Fe_8_S_8_ precursor. NifB itself contains three Fe_4_S_4_ clusters: one is a radical SAM cluster, while the other two, referred to as the K1 and K2 clusters, constitute the building blocks for the M cluster. A recent crystallographic structure of the enzyme revealed that the K1 and K2 clusters may be coordinatively unsaturated or bound to each other (Figs. 1c and 2e), forming a double cubane^15^. Based on an alternative refinement of the original diffraction data, another arrangement of the clusters was also proposed: rather than existing as two discrete Fe_4_S_4_ units, the K1 and K2 clusters may adopt a configuration in which they form a single Fe_8_S_8_ complex, simply termed the K cluster^16^. Its FeS topology is similar to that of the Fe_8_S_7_ P^N^cluster, but is distinguished by an eighth *μ*^2^(S^2−^) ligand (Figs. 1d and 2e and Supplementary Fig. 97). During subsequent stages, several enzymes work together with NifB to functionalize this K cluster with a carbide and a ninth *μ*^2^(S^2−^) ligand, yielding the Fe_8_S_9_C L cluster. The latter is then transferred to NifEN, wherein a hetero-metal (Mo or V) and a homocitrate ligand are installed, enabling the formation of the final MFe_7_S_9_C M cluster^7,8,17–20^ (Fig. 2c). By contrast, although it also requires many extra proteins, the Fe_8_S_8_ precursor to the P cluster, which is potentially K-cluster-like, matures directly within NifDK via the loss of one of its S atoms^7,8,21,22^ (Fig. 2d).

Parallel to the structural and biosynthetic characterizations of these metallocofactors (vide supra)^7,8,23–25^, chemists also pursued their synthetic replication^26–29^. Notable examples include (1) the fusion of catechol- or tris(pyrazole)borate- and phosphine- or chloride-supported MFe_3_S_4_ (M=V, Mo, W) heterocubanes^30,31^ to form edge-bridged double-heterocubanes^32–35^, which rearrange to M_2_Fe_6_S_7_ clusters on treatment with hydrosulfide or hydroselenide^36–38^; (2) P-cluster models of the type Fe_8_S_(7-*x*)_O_*x*_ (*x* = 0, 1)^39–44^, which are accessible by self-assembly, but whose mechanism of formation remains elusive^40,41,44^; and (3) all-Fe, ligand-, edge- and face-bridged double or multicubanes, which were stabilized by phosphines, N-heterocyclic carbenes or tripodal chelating tris-thiolates as supporting ligands^45–49^. However, although successful at replicating several of the nitrogenases’ metallocofactors’ core structures, these synthetic results remain disconnected from the biosynthetic pathways, because they do not capture the stepwise and coordinated assembly of simple FeS building blocks into complex clusters.

Departing from traditional approaches, we thus describe here a rational, cyclic and reversible chemical network of FeS cluster interconversions, enabling the stepwise assembly of synthetic [Fe_2_S_2_]^2+^ rhombs—the simplest FeS building blocks—to [Fe_8_S_8_]^*m*+^ (*m* = 2, 4, 6) clusters. Among these, the [Fe_8_S_8_]^4+^ cluster exhibits the same ‘core’ FeS topology as that proposed for the K cluster in ref. ^16^ (Figs. 1d and 2e), which we herein call an ‘interlocked’ double cubane (**ildc**). Its ultimate and penultimate precursors are themselves Fe_8_S_8_ clusters, namely, a ligand-bridged [Fe_8_S_8_]^6+^ or an edge-bridged [Fe_8_S_8_]^2+^ double cubane, respectively. Both resemble the structure that was proposed in ref. ^15^ for the K1 and K2 clusters (Figs. 1c and 2e). On the basis of the characterization of these clusters’ electronic, vibrational and ^57^Fe nuclear spectroscopic signatures, it is suggested that they could aid the identification of such intermediates in NifB and NifDK, respectively, while also providing fundamental insight into the factors governing their formation and interconversion.

## Results

### Rationalizing oxidation-state-dependent cluster conversions

**Fig. 3:**
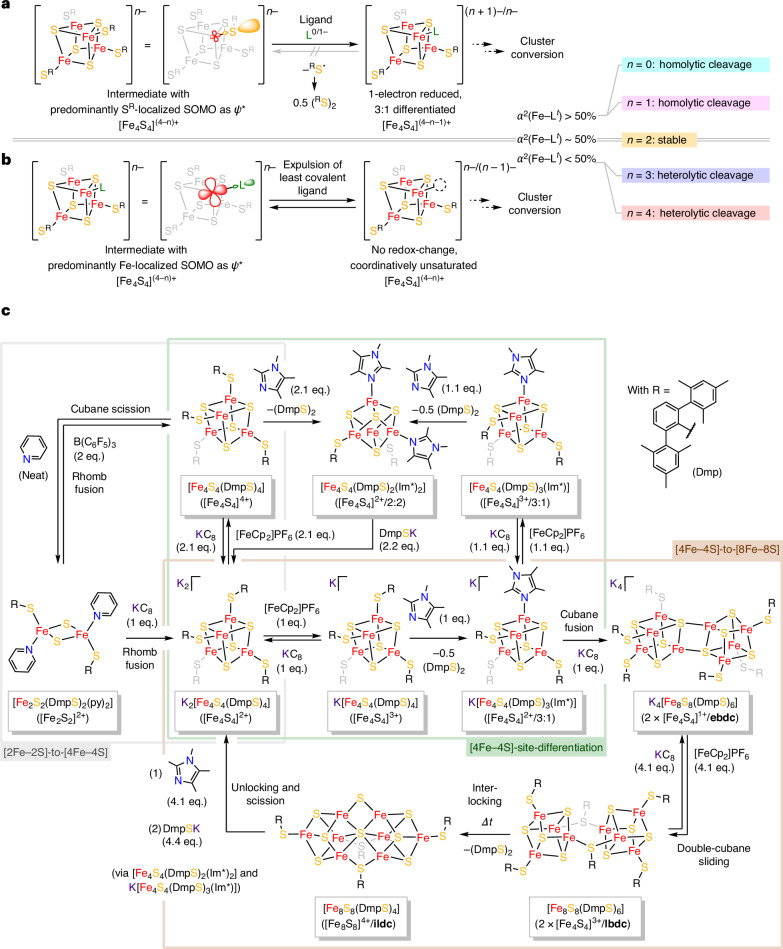
Strategy and synthetic network for the FeS cluster conversions in this work. **a**, Mechanism for oxidatively triggered covalency-driven (reductive) site differentiation of [Fe_4_S_4_(RS)_4_]^*n*−^ clusters by an arbitrary ligand, L, via homolytic bond cleavage. More specifically, L refers to a (*μ*^1^-bound) neutral or anionic dative ligand, which is less covalent than a thiolate and may be weakly *π* acidic. The overall cluster negative charge (*n*) increases by 1 if L is anionic and remains the same if L is neutral. By contrast, the core reduction level, (4 − *n*), decreases by 1, with each ligand substitution. **b**, Electronic origin of redox-neutral ligand loss from a reduced Fe_4_S_4_ complex via heterolytic bond cleavage. Here the overall negative cluster charge (*n*) decreases by 1 if L is anionic, and remains the same if L is neutral. Both behaviours (those shown in **a** and **b**, respectively) are ascribed to specific [Fe_4_S_4_]^(4−*n*)*+*^ oxidation states based on our study of the corresponding Fe–L^*t*^ (where L^*t*^ is a terminal ligand) bond covalencies (*α*^2^)^54^. **c**, Synthetic pathway from a [Fe_2_S_2_]^2+^ complex via canonical and site-differentiated [Fe_4_S_4_]^(4−*n*)*+*^ (*n* = 1, 2, 3) complexes to edge-bridged and ligand-bridged [Fe_8_S_8_]^2+^/[Fe_8_S_8_]^6+^ double cubanes, as well as an interlocked [Fe_8_S_8_]^4+^ cluster, topologically paralleling the proposed K cluster’s architecture. Equivalents (eq.) of reagents are given with respect to the educt cluster. In cases, where reductive elimination of disulfide occurs, this is indicated and the reaction was balanced accordingly. In all clusters, R denotes the 2,6-dimesitylphenyl residue. Below each structure, the corresponding sum formula and the overall core oxidation state/substitution pattern/cluster topology are indicated. Refer to the Supplementary Information for the exact synthetic conditions of each step.

The initial goal of our synthetic strategy was to achieve site differentiation, as this was proposed to play a key role in inducing cluster conversion^14,50^. In enzymes, Fe_4_S_4_-site differentiation occurs as the regiospecific substitution of a cysteine ligand, typically by histidine^51^, driven by an asymmetric cofactor binding pocket. Efforts to replicate this behaviour have been explored using rigid multidentate thiolate ligands^49,52^ or bulky N-heterocyclic carbenes^53^. However, these strategies are not transposable to [Fe_4_S_4_(RS)_4_]^*n*−^ clusters bearing simple monodentate thiolate ligands due to the latter’s high symmetry, which prevents precise control over the stoichiometry of ligand substitutions. Leveraging our group’s expertise with the K_*n*_[Fe_4_S_4_(DmpS)_4_] (*n* = 0–4; where Dmp is 2,6-dimesitylphenyl) model system^54–56^, and specifically the evaluation of the oxidation-state dependence of the Fe–S(*μ*^3^) and Fe–S(thiolate) bond covalency using X-ray absorption spectroscopy, we rationalized an original strategy to promote ligand exchange, rooted in the cluster’s electronic structure (Fig. 3a,b)^54^; the covalent character of a chemical bond is evaluated using the *α*^2^-parameter, which is often referred to as ‘covalency’^57^. However, *α*^2^ quantifies the ligand character in unoccupied (or half-occupied) metal 3*d* orbitals, which are covalently mixed with the ligand orbitals (here, S 3*p*), forming a ψ*(Fe–L) frontier molecular orbital. The *α*^2^-parameter, which may adopt any value between 0% and 100%, hence carries information about the localization of the frontier molecular orbital or, reciprocally, the polarization of the Fe–L bond. As such, and counterintuitively, the covalent character of a bond is maximal when it is not polarized, that is, when *α*^2^ is ~50%. For this work, it is important to realize that cubanes in the [Fe_4_S_4_]^3+^/[Fe_4_S_4_]^4+^ oxidation states are characterized by exceptionally large values of *α*^2^ for their Fe–S(thiolate) bonds (>50%). This means that the singly occupied ψ*(Fe–S^R^) molecular orbitals are predominantly localized on (or polarized towards) S, and therefore the binding of a less covalent ligand to Fe can trigger homolytic cleavage of the Fe–S(thiolate) bond. As the bonding electrons (that is, those in the corresponding *ψ*(Fe–S^R^) orbitals) are predominantly localized on Fe, this process reduces the Fe_4_S_4_ core by one electron, while the thiolate is formally oxidized to a radical species (Fig. 3a). At the same time, because of the core’s reduction, the remaining Fe–S(thiolate) bonds will be again more ‘covalent’ than before (*α*^2^ closer to 50%), and dimerization of the thiolate radical renders the reaction irreversible. This ensures that only a specific number of thiolate ligands are exchanged per cluster, allowing control of the substitution stoichiometry. Such mechanisms are disfavoured in cubanes in the [Fe_4_S_4_]^0^/[Fe_4_S_4_]^1+^ oxidation states, which exhibit low Fe–S(thiolate) bond covalencies (<50%). In contrast to their oxidized congeners, the fact that their singly occupied ψ*(Fe–L/S^R^) molecular orbitals are predominantly localized on Fe and their *ψ*(Fe–L/S^R^) orbitals remain on the ligand or thiolate makes them prone to (heterolytic) ligand exchange mechanisms (Fig. 3b).

### Constructing and traversing the synthetic cycle

The considerations outlined above were successfully implemented to construct the synthetic cycle shown in Fig. 3c, wherein repeated site differentiation and redox chemistry enabled FeS cluster conversion. Detailed procedures, additional synthetic explorations, as well as supporting discussions of the basic characterization data for all shown compounds are compiled in the Supplementary Information. Figure 3c highlights the central role of the canonical [Fe_4_S_4_]^2+^ complex, K_2_[Fe_4_S_4_(DmpS)_4_], which constitutes the junction, connecting the [2Fe–2S]-to-[4Fe–4S], [4Fe–4S]-site-differentiating, as well as [4Fe–4S]-to-[8Fe–8S] cluster interconversion cycles (highlighted in grey, green and brown boxes, respectively).

As anticipated from the Fe–S(thiolate) bond covalency considerations (Fig. 3a,b), [4Fe–4S]-site-differentiation is enabled by cluster oxidation: the redox congeners of K_2_[Fe_4_S_4_(DmpS)_4_] in the [Fe_4_S_4_]^3+^ and [Fe_4_S_4_]^4+^ states readily undergo homolytic Fe–S(thiolate) bond cleavage to exchange their DmpS^−^ligands for the less covalent 1,2,4,5-tetramethylimidazole (Im*) ligand. Thereby, the cluster core is reduced by one electron per additional Im*, until the [Fe_4_S_4_]^2+^ state is reached. This enabled the syntheses of K[Fe_4_S_4_(DmpS)_3_(Im*)] (ref. ^58^), [Fe_4_S_4_(DmpS)_3_(Im*)] and [Fe_4_S_4_(DmpS)_2_(Im*)_2_], whereby the last two are interconvertible depending on the stoichiometry of Im*, owing to the [Fe_4_S_4_]^3+^ redox level of [Fe_4_S_4_(DmpS)_3_(Im*)]. Conversely, because the [Fe_4_S_4_]^2+^–(DmpS^−^) bond is more covalent than the [Fe_4_S_4_]^2+^–(Im*) bond, Im* ligands are easily substituted from [Fe_4_S_4_(DmpS)_3_(Im*)]^−^ or [Fe_4_S_4_(DmpS)_2_(Im*)_2_] by stoichiometric DmpSK, re-forming K_2_[Fe_4_S_4_(DmpS)_4_].

The [2Fe–2S]-to-[4Fe–4S] cluster conversion chemistry occurs on reaching the all-ferric oxidation state^59^: [Fe_4_S_4_(DmpS)_4_] readily coordinates four pyridine (py) molecules, resulting in cubane scission, to form 2 equivalents of [Fe_2_S_2_(DmpS)_2_(py)_2_]. Pyridine ligands can be removed reversibly using B(C_6_F_5_)_3_, leading to the fusion of two [Fe_2_S_2_]^2+^ rhombs and re-forming [Fe_4_S_4_(DmpS)_4_], while the reduction of [Fe_2_S_2_(DmpS)_2_(py)_2_] reconstitutes K_2_[Fe_4_S_4_(DmpS)_4_] through the fusion of two transient [Fe_2_S_2_]^1+^ synthons. Notably, the fusion of a [Fe_2_S_2_]^2+^ with a [Fe_2_S_2_]^1+^ cluster, yielding a [Fe_4_S_4_]^3+^ complex, was not observed.

**Fig. 4:**
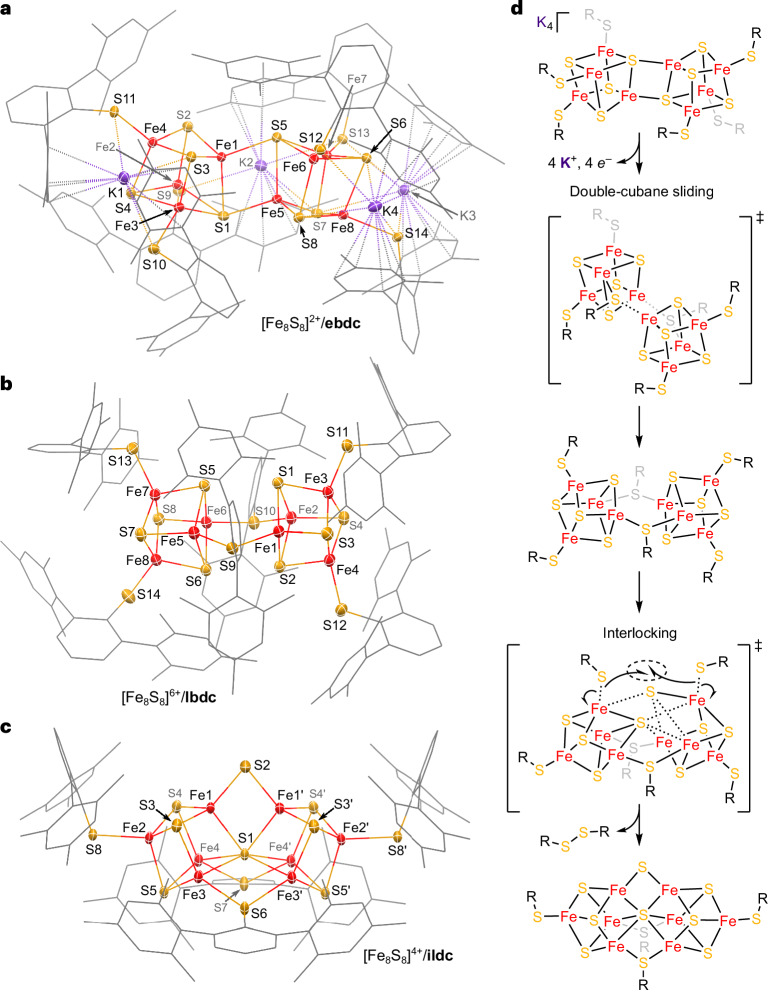
Structures and mechanistic interconversion of [Fe_8_S_8_]^*m+*^ (*m* = 2, 4, 6) clusters. **a**–**c**, Solid-state molecular structures of **ebdc** (**a**), **lbdc** (**b**) and **ildc** (**c**) in crystals of K_4_[Fe_8_S_8_(DmpS)_6_]·3.5(C_7_H_8_), [Fe_8_S_8_(DmpS)_6_]·3(C_7_H_8_) and [Fe_8_S_8_(DmpS)_4_], respectively. Displacement ellipsoids are shown at the 50% probability level for **ebdc** and **ildc**, and at 30% for **lbdc**, and are only displayed for the Fe, S and K atoms. Cocrystallized solvent molecules and hydrogen atoms have been omitted for clarity. **d**, Proposed mechanism for the interconversions of the three Fe_8_S_8_ clusters (**ebdc**, **lbdc** and **ildc**). Structures marked by a double dagger are postulated transition states.

In contrast to the [2Fe–2S]-to-[4Fe–4S] and [4Fe–4S]-site-differentiation cycles, [4Fe–4S]-to-[8Fe–8S] conversion chemistry is initiated reductively: addition of one electron and one K^+^ ion to K[Fe_4_S_4_(DmpS)_3_(Im*)] yields the edge-bridged [Fe_8_S_8_]^2+^ double cubane K_4_[Fe_8_S_8_(DmpS)_6_] (**ebdc**; Fig. 4a) via fusion of two site-differentiated [Fe_4_S_4_]^1+^ cubanes. This result is counterintuitive, considering that the Im*-substitution of one of the thiolate ligands shifts the cluster’s redox potential anodically, which, in turn, suggests an overall better stabilization of lower oxidation states (Supplementary Fig. 68). However, ligand-field considerations offer a different perspective regarding the cluster’s reactivity: reduction affords less covalent Fe_4_S_4_–ligand bonds^55^, enabling the facile heterolytic dissociation of the least covalent one (arguably that to Im*) and generating a coordinatively unsaturated Fe centre. The self-coordination of this intermediate via two *µ*^4^(S^2−^) ligands, affording **ebdc**, results in a more stable ligand field, in which net covalency is maximized (that is, *α*^2^ approaching 50%), compared with the one in which Im* ligation is maintained. **ebdc** can be reversibly oxidized by up to four electrons; its fully oxidized congener, however, does not possess an edge-bridged architecture. Instead, the four-electron oxidation is accompanied by what we propose to be a sliding motion of the two Fe_4_S_4_ subclusters, until they symmetrically face each other. Concomitantly, two of the *µ*^1^(DmpS^−^) ligands convert to *µ*^2^(DmpS^−^) ligands, yielding the ligand-bridged [Fe_8_S_8_]^6+^ double cubane [Fe_8_S_8_(DmpS)_6_] (**lbdc**; Fig. 4b,d). Although **lbdc** is formally ‘homoleptic’, the inequivalent DmpS^−^ ligands (bridging and terminal) render it site differentiated. We thus found that the primary coordination sphere is destabilized enough to induce spontaneous cluster conversion: metastable **lbdc** slowly converts to the ‘interlocked’ [Fe_8_S_8_]^4+^ double cubane, [Fe_8_S_8_(DmpS)_4_] (**ildc**; Fig. 4c), probably through a transition state initiated by a tilting motion of **lbdc**’s two ligand-bridged Fe_4_S_4_ subclusters, whereby the *µ*^2^(DmpS^−^) ligands act as hinges. Simultaneously, two *µ*^3^(S^2−^) ligands rearrange to one *µ*^2^(S^2−^) and one *µ*^6^(S^2−^) ligand, while two of the formerly *µ*^1^(DmpS^−^) ligands undergo reductive elimination (Fig. 4d). This mechanistic scenario is supported by the fact that stoichiometric amounts of (DmpS)_2_ were observed through ^1^H NMR (Supplementary Figs. 54–56) in situ during the transformation of **lbdc** to **ildc**. Given the precedents of covalency-driven homolytic Fe–S(thiolate) bond cleavage at [Fe_4_S_4_]^3+^/[Fe_4_S_4_]^4+^ clusters in this work, it seems reasonable to assume that the reductive elimination of (DmpS)_2_ from **lbdc**—formally composed of two [Fe_4_S_4_]^3+^ clusters—occurs homolytically as well. Although **ildc** appears coordinatively saturated and electrochemically robust (Supplementary Fig. 72), it readily converts back to Fe_4_S_4_ complexes in presence of competing ligands: 4 equivalents of Im* or DmpSK ‘unlock’ and cleave the double cubane, yielding 2 equivalents of [Fe_4_S_4_(DmpS)_2_(Im*)_2_] or K_2_[Fe_4_S_4_(DmpS)_4_], respectively, both of which are interconvertible themselves. As such, these transformations close the [4Fe–4S]-to-[8Fe–8S] cycle and integrate it into the network of [2Fe–2S]-to-[4Fe–4S]-to-[8Fe–8S] conversions (Fig. 3c).

Beyond these results, our synthetic explorations lead to the discovery of a variety of additional reactivity patterns, yielding unique FeS architectures and contributing to our fundamental understanding of this type of cluster conversion chemistry (Supplementary Figs. 2–5 and 81–91). Two species are particularly noteworthy: the edge-bridged [Fe_12_S_12_]^o^ triple cubane, K_6_[Fe_12_S_12_(DmpS)_6_] (**ebtc**; Supplementary Figs. 4 and 89), the preparation of which underscores the validity of our synthetic strategy (Fig. 3b), and [Fe_24_S_24_(DmpS)_10_] (Supplementary Figs. 3 and 88), an extremely large structurally characterized molecular FeS cluster.

### Electronic and vibrational signatures of the [8Fe–8S] clusters

**Fig. 5:**
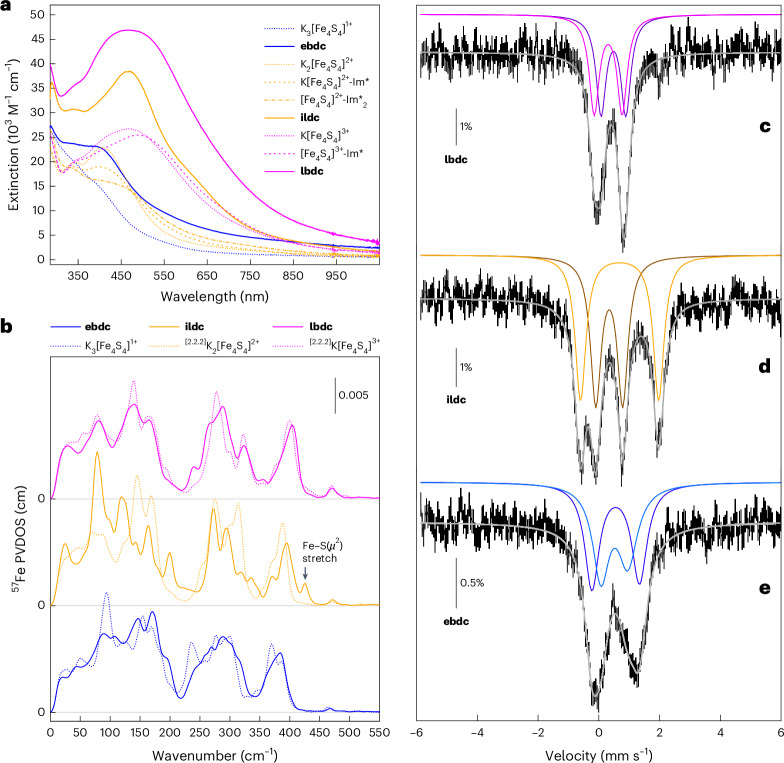
UV-vis electronic absorption, ^57^Fe NRVS PVDOS and ^57^Fe Mössbauer spectra of the [8Fe–8S] clusters. **a**, UV-vis electronic absorption spectra of 1 × 10^−4^ M toluene solutions of **lbdc** (magenta), **ildc** (yellow) and **ebdc** (blue), are shown as bold solid lines. For comparison, the spectra of the canonical and site-differentiated Fe_4_S_4_ complexes in the relevant oxidation states are also shown: they include K_3_[Fe_4_S_4_(DmpS)_4_] (dotted blue), K_2_[Fe_4_S_4_(DmpS)_4_] (dotted yellow), K[Fe_4_S_4_(DmpS)_3_(Im*)] (dashed yellow) and [Fe_4_S_4_(DmpS)_2_(Im*)_2_] (dotted-dashed yellow), K[Fe_4_S_4_(DmpS)_4_] (dotted magenta) and [Fe_4_S_4_(DmpS)_3_(Im*)] (dashed magenta). **b**, ^57^Fe NRVS PVDOS spectra of **lbdc**, **ildc** and **ebdc** (solid coloured lines). For comparison, the spectra of the corresponding canonical cubanes in their respective oxidation states are shown alongside, as dotted coloured lines. All spectra were recorded on powdered ^57^Fe enriched (>95%) samples between 30 K and 50 K. **c**–**e**, The 80 K Mössbauer spectra (vertical bars) recorded on powder samples of **lbdc** (**c**), **ildc** (**d**) and **ebdc** (**e**). No external magnetic field was applied in **d** and **e**, whereas a 0.06 T external magnetic field was applied along the *γ* ray direction in **c**. Simulations are overlaid as thick grey solid lines and components are displayed above the spectra as coloured thin solid lines. The nuclear parameters are the following: doublet 1 (violet) *δ* = 0.48 mm s^−1^, *∆E*_Q_ = 0.81 mm s^−1^; doublet 2 (magenta) *δ* = 0.31 mm s^−1^, *∆E*_Q_ = 0.92 mm s^−1^; common linewidth *Γ*_FWHM_ = 0.39 mm s^−1^ (**c**); doublet 1 (brown) *δ* = 0.34 mm s^−1^, *∆E*_Q_ = 0.89 mm s^−1^; doublet 2 (yellow) *δ* = 0.68 mm s^−1^, *∆E*_Q_ = 2.57 mm s^−1^; common linewidth *Γ*_FWHM_ = 0.43 mm s^−1^ (**d**) and doublet 1 (light blue): *δ* = 0.51 mm s^−1^, *∆E*_Q_ = 0.89 mm s^−1^, *Γ*_FWHM_ = 0.66/0.78 mm s^−1^; doublet 2 (dark blue): *δ* = 0.55 mm s^−1^, *∆E*_Q_ = 1.56 mm s^−1^, *Γ*_FWHM_ = 0.57/0.61 mm s^−1^ (**e**). FWHM, full-width at half-maximum.

We proposed that the change in the topology of the Fe_8_S_8_ clusters would be reflected in their spectroscopic properties. To this end, the spectroscopic signatures of **ebdc**, **lbdc** and **ildc** were compared by ultraviolet-visible light (UV-vis) electronic absorption, ^57^Fe nuclear resonance vibrational spectroscopy (NRVS) and ^57^Fe Mössbauer spectroscopy (Fig. 5). While **ebdc** and **lbdc** exhibit UV-vis electronic absorption spectra with features at similar energies as their [Fe_4_S_4_]^1+^/[Fe_4_S_4_]^3+^ redox congeners, **ildc** is characterized by electronic transitions that are atypical for an FeS cluster possessing equal numbers of Fe^II^ and Fe^III^ ions, and thus, an average oxidation state of Fe^2.5^; the intense peak at 467 nm falls within the range of the maxima observed for the more oxidized, Fe^2.75^-containing clusters^55^ and the shoulder at 631 nm is equally unusual (Fig. 5a). Similarly, the ^57^Fe partial vibrational density of states (PVDOS) spectra of **ebdc** and **lbdc** appear close to those of the canonical [Fe_4_S_4_]^1+^ and [Fe_4_S_4_]^3+^ complexes, respectively^60–62^, whereas **ildc** shows distinct vibrational bands, which deviate from the modes observed for its [Fe_4_S_4_]^2+^ counterpart (Fig. 5b). The most notable differences are evident in the region of the spectrum associated with FeS bending and twisting (or ‘breathing’) motions, around 80–220 cm^−1^. This shows that, while **ebdc** and **lbdc** are formally Fe_8_S_8_ clusters, their vibrational (and electronic) behaviour closely resembles that of cubanes because they retain intact Fe_4_S_4_ building blocks. Conversely, the FeS skeleton of **ildc** is topologically rearranged, rendering its properties distinct from those of Fe_4_S_4_ complexes. The most intense mode in its ^57^Fe PVDOS spectrum occurs at approximately 80 cm^−1^. A simulation of the normal modes using density functional theory (DFT) revealed a vibrational profile similar to experiment (Supplementary Fig. 111), wherein the most intense mode occurs around 100 cm^−1^; the latter identifying as ‘asymmetric twisting’ of the two Fe_4_S_3_ subunits. A mode at roughly 200 cm^−1^ is of further interest, because FeS cubanes usually display minimal intensity in this region^61^. Similar vibrations have, however, been observed for the M cluster, which possesses a *µ*^6^(C^4−^) ligand and an associated cluster breathing mode around 180–190 cm^−1^ (ref. ^63^). Inspection of the calculated modes around 200 cm^−1^ confirmed that the cluster topology of **ildc**, possessing a *µ*^6^(S^2−^) ligand (S1), indeed induces breathing modes at comparable energies (around 204 cm^−1^), despite the substantially different mass of its central *μ*^6^ ligand. This similar energy results from the fact that, in this particular mode, the central *µ*^6^ atom is not significantly displaced compared with its surrounding Fe and S atoms. A very different situation was observed for the mode appearing at 599 cm^−1^ for the M cluster^63^, which involves significant motion of the central atom. For **ildc**, simulation suggests that it appears at around 260 cm^−1^, 295–305 cm^−1^ and 370 cm^−1^ (corresponding to the three respective dimensions of *µ*^6^(S^2−^) motion). As for the cubanes, the PVDOS around 400 cm^−1^ is primarily derived from Fe–S(thiolate) stretches ^60–62^. Notably, however, the displacement of the other distinct sulfide ligand, namely, the *µ*^2^(S^2−^) sulfide (S2), occurs at a rather high energy of 420 cm^−1^ (marked by a black arrow in Fig. 5b). This mode is neither found in cubanes, nor in double cubanes such as **lbdc** or **ebdc** (Fig. 5b), and it should also not occur in Fe_8_S_7_ P^N^-type clusters, which lack *µ*^2^(S^2−^) ligands. We thus anticipate that this vibrational feature could serve as a useful signature to identify such a K cluster in the more complex biological environments.

### Valence topologies of the [8Fe–8S] clusters

To further understand the asystematic electronic and vibrational properties of **ildc**, compared with **ebdc** and **lbdc**, the valence topology of the three Fe_8_S_8_ clusters was rationalized by ^57^Fe Mössbauer spectroscopy (Fig. 5c–e): **lbdc** and **ebdc** exhibit zero-field 80 K powder spectra with two main absorption lines (Fig. 5c,e), while **ildc** shows four (Fig. 5d). Regardless of the simulation model (refer to the Supplementary Information), the average isomer shift of the spectra, *δ*_avg_, coincides well with the clusters’ average Fe oxidation states^55^. However, while the simulations of the spectra of **ebdc** and **lbdc** suggest delocalized Fe valences, typical for Fe_4_S_4_ complexes (individual isomer shifts, *δ*_i_, are 0.45 < *δ*_i_ < 0.58 mm s^−1^ for **ebdc** and 0.31 < *δ*_i_ < 0.48 mm s^−1^ for **lbdc**), the unique simulation of **ildc**’s spectrum presents discrete doublets at *δ*_i_ = 0.34 and 0.68 mm s^−1^ (Fig. 5d). These values are substantiated by the analysis performed on the 5.7 K spectra recorded with 0.06 to 7 T magnetic fields applied along the *γ* ray direction (Supplementary Fig. 102a–d) and that performed on the 293 K zero-field spectrum (Supplementary Fig. 101 and Supplementary Table 11), evidencing a diamagnetic ground state with localized ferric and ferrous sites, even at room temperature. Therefore, we establish a Robin–Day class I (valence-trapped) mixed-valence state in **ildc**, for which a bond-valence sum analysis of the crystallographic structure suggests that [Fe3,Fe4] are in the +II and Fe1 and Fe2 in the +III oxidation states (Supplementary Table 10). Although there are conflicting interpretations of Mössbauer spectra in the literature^40,44^, similar valence-trapped ground states were suggested for the *μ*^6^(S^2−^)-ligand containing [Fe_8_S_7_]^4+^ clusters (possessing six Fe^II^ and two Fe^III^ atoms)^64^. However, to date, no direct confirmation of this phenomenon by variable-temperature variable-field Mössbauer spectroscopy has been provided, and the origin and nature of the valence-trapped state has not been rationalized. The fact that the [Fe_4_S_4_]^2+^ subcluster motif in **ildc** (possessing two Fe^II^ and two Fe^III^ atoms) has localized Fe^II^ and Fe^III^ sites is particularly intriguing, because it stands in contrast with the fully delocalized [Fe_4_S_4_]^2+^ (two Fe^II^ and two Fe^III^) cluster core of ‘neat’ cubanes and the (at least partially) delocalized cuboidal [Fe_4_S_3_C]^0^-motif of the M cluster (likewise possessing two Fe^II^ and two Fe^III^ atoms)^65–67^.

**Fig. 6:**
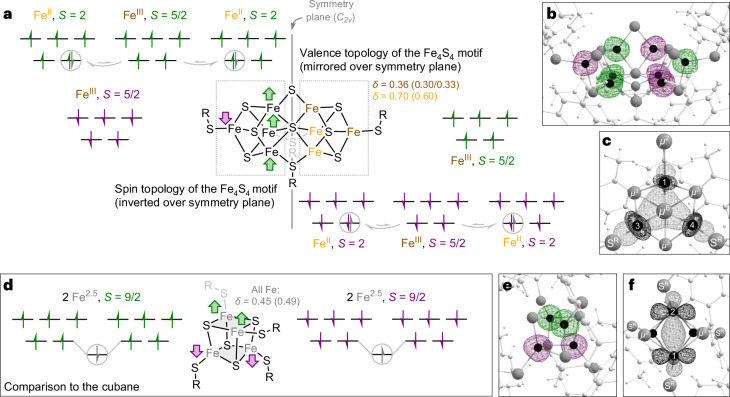
Electronic basis for the valence trapping in **ildc**, compared with the [Fe_4_S_4_]^2+^ cubane. **a**, Qualitative molecular orbital diagram for the electronic structure of **ildc** calculated with DFT. The *α* spins are drawn in green and *β* spins in purple. Local minority spins pertinent to SDD are drawn in grey and the corresponding molecular orbital is circled in grey. The spin and valence topologies of the complex are schematically drawn in separate (symmetric) halves of the molecule. For completion, the former is inverted, while the latter is mirrored over the symmetry plane indicated as a grey line. The experimental ^57^Fe Mössbauer isomer shift values associated with each Fe site are shown alongside, with the respective theoretical DFT-derived isomer shift value given in brackets. **b**, Calculated spin-density isosurface plot (isodensity cut-off 0.015) for **ildc** (left). **c**, Isosurface plot (isodensity cut-off 0.04) of one of the four equivalent orbitals relevant to the evaluation of SDD, which are highlighted in a grey circle in **a**. The corresponding atomic populations (*φ*) are *φ*(Fe1) = 16% and *φ*([Fe3,Fe4]) = 77%. **d**, Comparative qualitative molecular orbital diagrams for [Fe_4_S_4_(DmpS)_4_]^2−^ and its associated measured (and calculated) ^57^Fe Mössbauer isomer shifts determined at equivalent temperature (refer to Supplementary Fig. 103 for the spectra and simulations), and at the equivalent level of theory. The Fe_2_S_2_ subclusters across which SDD occurs are highlighted in grey. **e**,**f**, Comparative spin-density isosurface plot (**e**) and one of the two equivalent orbitals relevant to SDD for [Fe_4_S_4_(DmpS)_4_]^2−^ (**f**). The corresponding atomic populations (*φ*) are *φ*(Fe1) = 47% and *φ*(Fe2) = 48%.

This apparent discrepancy prompted us to investigate possible electronic origins of the trapped valences in **ildc** by complementing our spectroscopic investigations with broken-symmetry DFT calculations. The qualitative molecular orbital diagram of the energetically most favoured broken-symmetry solution is summarized in Fig. 6a, alongside its spin-density isosurface plot (Fig. 6b). Among all converged wavefunctions, this one produced ^57^Fe Mössbauer isomer shifts and PVDOS in best agreement with experiment (Fig. 6a, Supplementary Table 12 and Supplementary Fig. 111) and is thus in clear support of the valence-trapped configuration. Although they are not real molecular properties (observables), the populations of localized orbitals were then evaluated to assess and visualize electronic delocalization: while this method predicts perfectly symmetric double-exchange (or spin-dependent delocalization, SDD) on the Fe_2_S_2_ subclusters of [Fe_4_S_4_(DmpS)_4_]^2−^ (Fig. 6d–f), the comparable orbitals of **ildc** are significantly desymmetrized towards the itinerant electrons residing on [Fe3,Fe4] (Fig. 6c). This model (Fig. 6a) thus constitutes an appropriate electronic basis for the valence-trapped state of **ildc**.

From a structural perspective, the geometric parameters of the delocalized [Fe_4_S_4_]^2+^ core of [Fe_4_S_4_(DmpS)_4_]^2−^, the trapped [Fe_4_S_4_]^2+^ subcluster of **ildc** and the (at least partially) delocalized [Fe_4_S_3_C]^0^ motif of the M cluster were considered (Supplementary Fig. 110): while the average Fe–S(*μ*^3^) bonds in both **ildc** (2.283(2) Å) and the M cluster (2.274 Å)^68^ are similar to those of canonical [Fe_4_S_4_]^2+^ clusters (2.285 Å)^55,56^, the Fe–L(*μ*^6^) bonds to the central ligand are significantly longer in **ildc** (2.436(2) Å; L=S) and significantly shorter in the M cluster (1.990 Å; L=C)^68^. Therefore, whereas the M cluster’s ‘compacted’ [Fe_2_S(*μ*^3^)C(*μ*^6^)]^1−^ subclusters appear to sustain SDD between their Fe atoms, probably due to the short Fe–C bonds and high charge-to-bridged-atom ratio of the *μ*^6^(C^4−^) ligand, **ildc**’s ‘expanded’ [Fe_2_S(*μ*^3^)S(*μ*^6^)]^1+^ subclusters do not, offering a simple geometric argument for the observed effect.

## Discussion

The sequence of reactions required to traverse the entire network of FeS cluster conversions shown in Fig. 3c offers valuable insights into potential mechanisms at stake in the biogenesis of the nitrogenase P-, L- and M-cluster cofactors. All three octa-Fe clusters (**ebdc**, **lbdc** and **ildc**) can serve as molecular models for the Fe_8_S_8_ K cluster, the structure of which remains a topic of discussion (vide supra) and, together, may represent molecular snapshots of the [4Fe–4S]-to-[8Fe–8S] fusion occurring in NifB^7,8^. We foresee that our analysis of the clusters’ essential spectroscopic fingerprints (Fig. 5) will aid in confirming, respectively disproving their involvement in the corresponding cofactor maturation pathways. For this, particularly the trapped valences and the identification of the characteristic Fe–S(*μ*^2^) stretching vibration in **ildc** should represent useful spectroscopic features. However, we point out that among the three Fe_8_S_8_ complexes, **ildc** is distinct and exhibits identical FeS topology to that proposed recently in ref. ^16^. Our results show that such a structure can be systematically assembled through stepwise synthesis, beginning from an [Fe_2_S_2_]^2+^ complex as the simplest FeS cluster building block. Depending on whether they occur in a concerted or stepwise manner, this involves approximately nine fundamental chemical steps:

1. reduction of two [Fe_2_S_2_]^2+^ clusters to [Fe_2_S_2_]^1+^ clusters;
2. fusion of two [Fe_2_S_2_]^1+^ clusters to form a canonical [Fe_4_S_4_]^2+^ cluster;
3. one-electron oxidation to a [Fe_4_S_4_]^3+^ cluster;
4. reductive site differentiation of the canonical cluster by a labile noncanonical ligand, regenerating a [Fe_4_S_4_]^2+^ cluster;
5. reduction, leading to the loss of the labile noncanonical ligand, yielding a coordinatively unsaturated [Fe_4_S_4_]^1+^ cubane;
6. fusion of two coordinatively unsaturated cubanes to form an edge-bridged double cubane;
7. oxidation of the edge-bridged double cubane;
8. rearrangement of the cluster architecture into a ligand-bridged double cubane through a sliding motion and
9. interlocking of the two Fe_4_S_4_ subclusters by mobilization of *µ*^3^(S^2−^) ligands and a tilting motion along the hinge provided by the two bridging ligands, concomitant with reductive elimination of disulfide.

These cluster conversions are exclusively driven by alternating redox- and site-differentiation reactions, which repeatedly destabilize the FeS cluster’s primary coordination sphere and sequentially force it to relax into a new, more stable structure, while also increasing its size. In a broader context, we thus emphasize that two key factors appear to govern FeS cluster conversion chemistry: (1) the FeS cluster’s ligands, and (2) its oxidation state. In enzymes, these are controlled by the residues accessible in the cofactor binding pocket and the local electrochemical potential. We also emphasize that the cluster oxidation state and the associated ligand covalencies control whether and how Fe_4_S_4_ site differentiation occurs; polarized Fe–S(thiolate) bonds are prone to cleave either homolytically if *α*^2^ is >50%, or heterolytically if *α*^2^ is <50% (with respect to the ligand). In between, highly covalent bonds (*α*^2^ ≈ 50%) are stable, rendering [Fe_4_S_4_]^2+^ complexes the thermodynamic sink of FeS cluster conversion chemistry. This was exactly reflected in the reactivity observed in this work, where we demonstrated homolytic Fe–ligand bond cleavage for [Fe_4_S_4_]^3+^ and [Fe_4_S_4_]^4+^ complexes versus heterolytic bond cleavage in [Fe_4_S_4_]^1+^ and [Fe_4_S_4_]^0^, as well as that observed in previous studies, where we reported the aggregation of [Fe_4_S_4_]^0^ clusters via (heterolytic) loss of DmpS^−^ ligands^56^. Given that the reduction potential of the DmpS^−^ ligand (roughly −1.2 to −1.4 V versus normal hydrogen electrode; Supplementary Fig. 59) closely matches that of cysteinate (−1.38 to −1.45 V versus normal hydrogen electrode)^69^, it appears plausible that similar considerations could govern the reactivity of natural Fe_4_S_4_ cofactors.

Furthermore, our results indicate that **ildc**, or a topologically similar cluster, could represent a key intermediate at which the biosynthetic pathways of the P and M clusters diverge. Considering the uncertainties of knowing whether the K1 and K2 clusters fuse to an interlocked topology before or after the C-atom transfer from SAM^7,8,17,18^, and whether the eighth S of the P cluster is lost before, during or following cluster interlocking^21,22^, it is plausible that an interlocked topology of fused Fe_4_S_4_ synthons serves as a precursor in both biosynthetic pathways, aligning with the fact that they are thought to be evolutionarily related^9,10^.

## Conclusion

Altogether, this work introduced an original approach to control FeS cluster conversion chemistry through alternating redox- and site-differentiation reactions. On this basis, we replicated the initial steps of M-cluster maturation, starting from a Fe_2_S_2_ rhomb, and ultimately arriving at a model for the Fe_8_S_8_ K cluster, within a coherent synthetic cycle. The isolation and characterization of all (meta)stable intermediate products allowed rationalizing the conditions driving each of the (electro)chemical steps and provides a framework to better understand and identify similar species during the biogenesis of the P and M clusters.

## Methods

Experiments were carried out under a dry, oxygen-free Ar atmosphere using Schlenk-line and glove-box techniques. All solvents and reagents were rigorously dried and deoxygenated before use. Compounds were experimentally characterized by various techniques, including single-crystal X-ray diffraction, ^1^H/^13^C NMR, UV-vis electronic absorption, cyclic voltammetry, ^57^Fe NRVS, ^57^Fe Mössbauer and elemental analysis. Furthermore, **ildc** and [Fe_4_S_4_(DmpS)_4_]^2−^ were theoretically probed using DFT calculations. Further details are available in the Supplementary Information with this article.

## Online content

Any methods, additional references, Nature Portfolio reporting summaries, source data, extended data, supplementary information, acknowledgements, peer review information; details of author contributions and competing interests; and statements of data and code availability are available at 10.1038/s41557-025-01895-9.

## Supplementary information

Supplementary Information Supplementary Methods (synthetic procedures, instrumentation, computational details), Discussion, Notes 1–6, Figs. 1–112, Tables 1–12 and Refs. 1–74.

Supplementary Data 1 XYZ format coordinates for the broken-symmetry DFT-optimized geometry of **ildc**.

Supplementary Video 1 GIF animations of broken-symmetry DFT-calculated normal modes of **ildc**.

## Data Availability

The Supplementary Information (in PDF format) contains all synthetic procedures, experimental details, characterization data, computational methods and results, as well as additional supporting discussions relevant to this work. All unprocessed raw data for nonstandard characterization techniques (NRVS and ^57^Fe Mössbauer spectra) that were used to obtain the graphs shown in Figs. 5a–e and/or the Supplementary Information are provided by the authors from the ETH Zürich Research Collection at 10.3929/ethz-b-000739805. All molecular structures derived from single-crystal X-ray diffraction analyses are deposited as crystallographic information files in the Cambridge Structural Database under the accession numbers 2389114 ([Fe_2_S_2_(DmpS)_2_(py)_2_]), 2389123 (**ebdc**), 2389116 (**lbdc**), 2389115 (**ildc**, unit cell 2), 2389110 (**ildc**, unit cell 1), 2389111 ([Fe_4_S_4_(DmpS)_2_(Im)_2_]·K[Fe_4_S_4_(DmpS)_3_(Im)]), 2389112 ([Fe_4_S_4_(DmpS)_3_(Im)]·0.5[Fe(Im)_6_]), 2389122 ([Fe_10_S_10_(DmpS)_6_(Im)_2_]), 2389124 ([Fe_4_S_4_(DmpS)_4_]·0.5[Fe(MeCN)_6_]), 2389127 ([Fe_4_S_4_(DmpS)_3_(THF)_3_]), 2389125 (DmpSSSDmp), 2389120 (Im*·B(C_6_F_5_)_3_), 2389119 ([Fe_24_S_24_(DmpS)_10_]), 2389109 (K_6_[Fe_12_S_12_(DmpS)_6_]), 2389126 ([Fe_4_S_4_(DmpS)_3_(Im*)]), 2389118 (K[Fe_4_S_4_(DmpS)_3_(Im*)]), 2389117 (^[18-C-6]^K[Fe_4_S_4_(DmpS)_3_(Im*)]), 2389113 ([Fe_4_S_4_(DmpS)_2_(Im*)_2_]), 2389121 ([Fe_8_S_7_(DmpS)_3_Cl_2_]) and 2389108 ([Fe(DmpS)Cl]_2_). Copies of the data can be obtained free of charge at https://www.ccdc.cam.ac.uk/structures/. All data were analysed using standard software plugins whenever appropriate, as described in the Supplementary Information. The DFT-optimized XYZ coordinates of **ildc** are available on Supplementary Data 1 and animations of the theoretical normal modes are provided in Supplementary Video 1.

## Code availability

All data were analysed using standard software plugins whenever appropriate, as described in the Supplementary Information.
