# Supplementary material for: Stepwise and reversible assembly of [2Fe–2S] rhombs to [8Fe–8S] clusters and their topological interconversions
Source: Nat Chem. 2025 Aug 20;17(10):1586–95. doi: 10.1038/s41557-025-01895-9 (PMC12491067; doi:10.1038/s41557-025-01895-9)
Supplement: Supplementary file 1 — Supplementary Methods (synthetic procedures, instrumentation, computational details), Discussion, Notes 1–6, Figs. 1–112, Tables 1–12 and Refs. 1–74. [file 41557_2025_1895_MOESM1_ESM.pdf]

nature chemistry

Supplementary information https://doi.org/10.1038/s41557-025-01895-9

Stepwise and reversible assembly of [2Fe–2S] rhombs to [8Fe–8S] clusters and their topological interconversions

In the format provided by the authors and unedited

General Considerations **2**
Supporting Discussion of Syntheses and Characterization Data **4**
The [2Fe-2S] cluster *4*
The [4Fe-4S] clusters *4*
The [8Fe-8S] clusters *5*
Simulation of [8Fe-8S] Mössbauer spectra *7*
Synthetic Procedures **8**
DmpSK *8*
$DmpS^{[18-C-6]}K$ *8*
$(DmpS)_2$ *8*
$[Fe_2S_2(DmpS)_2(py)]$ and its interconversions *9*
$K[Fe_4S_4(DmpS)_3(Im^*)]$ *11*
$[Fe_4S_4(DmpS)_3(Im^*)]$ *12*
$^{[2.2.2]}K[Fe_4S_4(DmpS)_3(Im^*)]$ *12*
$^{[18-C-6]}K[Fe_4S_4(DmpS)_3(Im^*)]$ *13*
$[Fe_4S_4(DmpS)_2(Im^*)_2]$ *13*
*$K_4[Fe_8S_8(DmpS)_6]$ (**ebdc**)* *14*
*$[Fe_8S_8(DmpS)_4]$ (**ildc**)* *15*
*$[Fe_8S_8(DmpS)_6]$ (**lbdc**)* *15*
Conversion of $[Fe_4S_4(DmpS)_2(Im^*)_2]$ to $K_2[Fe_4S_4(DmpS)_4]$ *16*
*Conversion of **ildc** to $K_2[Fe_4S_4(DmpS)_4]$* *16*
*Initial preparation/identification of **ildc** and synthetic attempts toward its direct synthesis from $[Fe_4S_4(DmpS)_4]$ and $[Fe_4S_4(DmpS)_2(Im^*)_2]$* *17*
*$K_6[Fe_{12}S_{12}(DmpS)_6]$ (**ebtc**)* *21*
Other Non-Preparative Procedures and Transformations of the Oxidized Fe_4S_4 Complexes **22**
Reaction of $[Fe_4S_4]^{3+}$ with 1-Me-imidazole *22*
Reaction of $[Fe_4S_4]^{4+}$ with excess 1-Me-imidazole *23*
Reaction of the $[Fe_4S_4]^{4+}$ cubane with sub-stoichiometric 1-Me-imidazole *23*
Reaction of the $[Fe_4S_4]^{4+}$ cubane with excess acetonitrile *23*
*Reaction of the $[Fe_4S_4]^{4+}$ cubane and **lbdc** with excess THF* *23*
UV-vis Electronic Absorption Spectroscopy **25**
^1H and ^{13}C NMR Spectroscopy **35**
Cyclic Voltammetry **72**
Crystallographic Details **84**
Crystallographer's remarks and tables *86*
*BVS analysis of **ildc*** *96*
Structure images *97*
Structural figures for supporting discussion *109*
Supplementary ^{57}Fe Mössbauer Spectra and Simulations **110**
Density Functional Theory Calculations for *ildc* **116**
References **125**

General Considerations

All canonical iron-sulfur cubane complexes studied in this work, including their ^{57}Fe labelled derivatives were synthesized according to the procedures we published in the literature, from [Fe(DmpS)$_2$] and [Fe(DmpS)(TripS)]$_2$.[1-4] K[BArF$_{24}$] was synthesized according to literature methods, from $BF_3{\cdot}OEt_2$ and 3,5-trifluoromethyl-1-bromobenzene,[5] and purified by repeated recrystallization from DCM/THF (1:1) layered with hexane until the supernatant and product appeared entirely colorless. [^{n}Bu$_4$N][PF$_6$] was purchased from Sigma Aldrich and recrystallized from hot ethanol prior to use. Ferrocenium hexafluorophosphate ([FeCp$_2$]PF$_6$) was synthesized as part of ETH Zürich's AOCP II inorganic and organic chemistry laboratory practical course by 3rd semester students according to the literature methods.[6,7] 1,2,4,5-tetramethylimidazole (Im*) was purchased from TCI and used as received. Toluene, pentane, acetonitrile and dichloromethane were purified using a Vigor® Solvent purification system (SPS) and degassed by 4 freeze-pump-thaw cycles prior to use. THF and diethyl ether were purified on the SPS and dried separately again, by stirring over K/benzophenone, followed by distillation and freeze-pump-thaw cycles. All spectroscopic sample preparations and syntheses were carried out inside Ar-filled gloveboxes (Vigor®), where oxygen and moisture levels were maintained below 1 ppm—unless otherwise stated.

NMR spectra were recorded on a 300 MHz Bruker AVII spectrometer at room temperature. ^{1}H and ^{13}C spectra are reported in parts per million (ppm) and are calibrated with respect to the corresponding solvent residual peak. ^{13}C NMR spectra were recorded with complete proton decoupling and the spectra are likewise calibrated with respect to the corresponding solvent residual peak. Multiplet signals are reported as follows: s=singlet, d=doublet, t=triplet, q=quartet, quint=quintet, sept=septet, m=multiplet, b=broad, or combinations thereof. Peaks in the ^{1}H spectra are labelled with numbers from 1-5 for the five inequivalent protons of the DmpS$^-$ ligand and otherwise, if other ligands are attached to the cluster (*i.e.* Im*).

UV-vis electronic absorption data were collected on an Agilent Cary 60 UV-Vis Spectrophotometer, which was connected to a sampling probe (d=2 mm or 5 mm) inside the Glovebox with an optical fiber. Measurements were performed on $1{\cdot}10^{-4}$ M toluene solutions, unless stated otherwise. Spectral bands are classified as peaks (p) or shoulders (sh).

Electrochemical data were collected in a 20 mL or 5 mL cell, depending on convenience, using glassy carbon as working electrode (diameter 3 mm), a platinum wire as counter-electrode and a silver wire dipped in a 0.01 M solution of $AgNO_3$ in a 0.1 M solution of [^{n}Bu$_4$N][PF$_6$] in MeCN as reference. The reference electrode was separated from the CV cell using a guard filled with the same electrolyte as used in the cell. The potential was controlled by a BioLogic SP-300 potentiostat. The mixture was stirred, and the working electrode polished after every scan.

Elemental analyses were carried out in the Mikrolabor of ETH Zürich on a LECO TruSpec® Micro spectrometer.

^{57}Fe Mössbauer spectra were recorded on powder samples contained in Delrin cups between 5 and 80 K on a low-field Mössbauer spectrometer equipped with a Janis CCR 5 K cryostat or on a high-field Mössbauer spectrometer equipped with an Oxford Instruments Spectromag 4000 cryostat containing an 8 T split-pair superconducting magnet. Both spectrometers were operated in a constant acceleration mode in transmission geometry. The isomer shifts were referenced against that of a room-temperature metallic iron foil. Analysis of the data was performed with the WMOSS Mössbauer Spectral Analysis Software (www.wmoss.org, 2012–2013, Web Research, Edina) and with a home-made program (SimuMoss software. C. Charavay, S. Segard, F. Edon, M. Clémancey, G. Blondin. CEA/iRTSV, CNRS, Univ. Grenoble Alpes).[8] Hereby, velocity and absorption values were obtained after the classical folding procedure of the crude recorded data (channel number and counts of photons per channel). The error is the square root value of the counts expressed in percentage versus the counts associated to the baseline.

Recordings were performed on powder samples of the complexes synthetized with ^{57}Fe in natural abundance with the exception of ***ildc***. The sample of ***ildc*** for Mössbauer analysis was prepared from a mix of precursor compounds of two different synthetic batches; One from a 95% enriched sample, and one from a natural abundance sample in the approximate ratio of 1:7. Assuming that the ^{57}Fe will be statistically distributed in the product, the final enrichment of the sample should have been ca. 12%.

^{57}Fe NRVS was measured with a published method[9-12] at SPring-8 BL19LXU in operational C mode—with a 145.5 ns interval between X-ray pulses to match the 1/e nuclear decay period for ^{57}Fe. The X-ray beam from the planar undulator (ΔE~100 eV) was first monochromatized to ~1 eV bandwidth by a high heat load monochromator (HHLM), and then further monochromatized to 0.8 meV linewidth centered at the nuclear resonant energy of ^{57}Fe (14.4 keV) by a high-resolution monochromator (HRM). In the orientation, which is perpendicular to the incident X-ray beam, a 2×2 avalanche photodiodes array (APDs) was used to detect the ^{57}Fe nuclear fluorescence ($h\nu_1$) and the Fe K fluorescence ($h\nu_2$) from internal conversion following the nuclear resonance excitation at 14.4 keV. Since APDs have fast response and high time resolution, the weak nuclear scattering signal (e.g. τ=143 ns for ^{57}Fe) can be well separated from the huge electronic scattering background in the time domain, avoiding a low throughput diffraction spectrometer.[9-12] Then the pure nuclear scattering signal *vs*. the vibrational energies form the raw NRVS spectra. For most of the iron-sulfur complexes, an even time of 3 seconds per point (3 s/p) was used to scan the whole spectrum from –240 to 540 cm^{-1}. For the complexes with possible high energy features, the scan range was extended to 600 cm^{-1} and sectional scans were used to emphasize the possible high energy region. For example, 6 s/p was used for the 400-600 cm^{-1} region while 3 s/p was used for the region from -240 cm^{-1} to 400 cm^{-1}. Each final spectrum in this publication is the average from 6-12 such scans. The collected raw NRVS scans was processed via spectra.tools[9,13] which combines the data format transformation with the PHOENIX software package[14] to yield ^{57}Fe partial vibrational density of states (PVDOS), which is a pure molecular property and is independent of any experimental conditions. In principle, the calculation of element specific PVDOS involves the summation of the raw NRVS, Fourier-log calculation[13,15] and spectral normalization through Lipkin's sum rule.
To maintain the samples' integrity during the measurement and to increase the scattering intensity in the Stoke branch, the NRVS samples were cooled with a helium flow cold finger cryostat or an enclosed helium circulator maintained at 10K. Nevertheless, the real sample temperatures determined from NRVS analysis *via* spectra.tools were found to range from 40 to 65 K due to the extremely close sample-detector distance.[9,16]

Supporting Discussion of Syntheses and Characterization Data

The [2Fe-2S] cluster

Upon dissolution of all-ferric [Fe_4S_4(DmpS)$_4$] in neat pyridine, single-crystalline di-ferric [Fe_2S_2(DmpS)$_2$(py)$_2$] can be isolated in 77% yield from a pyridine solution layered with pentane (Fig. 3C and Supplementary Fig. 1). It has a characteristic ^{1}H NMR spectrum (Supplementary Fig. 27), accounting for all signals of the DmpS$^–$ and pyridine ligands, and a UV-vis electronic absorption spectrum in line with those of similar synthetic or native [Fe_2S_2]$^{2+}$ clusters.[17-20] Furthermore, ^{57}Fe Mössbauer spectroscopy conducted at 5.3 K and 0.06 T as well as at 5.3 K and 7 T evidences nuclear parameters supporting the fact that it contains two high-spin ferric sites, which are antiferromagnetically coupled to adopt a S=0 spin ground state: δ=0.31 mm s^{-1}, ΔE_Q=0.85 mm s^{-1} and η=1.00 (Supplementary Fig. 98). The 0.31 mm s^{-1} isomer shift value is similar to that observed for ferric sites in biological 2Fe-2S clusters presenting a mixed Cys/His coordination as in IscR,[21] MitoNEET[22] and Apd1 mutants.[23] In analogy to what has been reported by Tatsumi for [Fe_4S_4(N(TMS)$_2$)$_4$]/[Fe_2S_2(N(TMS)$_2$)(py)$_2$],[24] this reaction can be quantitatively reversed by treatment of [Fe_2S_2(DmpS)$_2$(py)$_2$] with stoichiometric amounts of B(C_6F_5)$_3$ (Supplementary Figs. 7A, 30 and 31). However, in contrast to Tatsumi's system, the Fe_4S_4 cluster scission does not proceed cleanly if pyridine is not used as a neat reagent in great excess (Supplementary Figs. 7B, 28 and 29). [Fe_2S_2(DmpS)$_2$(py)$_2$] can be reduced over 1 equivalent of potassium graphite (KC_8), yielding the [Fe_4S_4]$^{2+}$ cubane complex, K_2[Fe_4S_4(DmpS)$_4$][1] (Fig. 3C and Supplementary Fig. 1A), as the product of fusion between two transient [Fe_2S_2]$^{1+}$ rhombs. This behavior is reflected in the cyclic voltammogram of [Fe_2S_2(DmpS)$_2$(py)$_2$] recorded in 1,2-difluorobenzene (*o*-DFB), wherein a cathodic current at –1.21 V *vs*. Fc/Fc$^+$ indicates an irreversible chemical step (Supplementary Fig. 60).

The [4Fe-4S] clusters

One-electron oxidation of K_2[Fe_4S_4(DmpS)$_4$] with 1 equivalent of [$FeCp_2$]PF_6 followed by treatment with 1 equivalent of 1,2,4,5-tetramethylimidazole (Im*) results in the formation of the 3:1 site-differentiated cluster K[Fe_4S_4(DmpS)$_3$(Im*)] (Fig. 3C). The K$^+$ ion can be sequestered from the complex into 18-crown-6 ether or [2.2.2]-cryptand, yielding well-separated cation-anion pairs. The oxidation of [Fe_4S_4(DmpS)$_3$(Im*)]$^–$ preserves the [Fe_4S_4]$^{2+/3+}$ architecture, forming [Fe_4S_4(DmpS)$_3$(Im*)], which can be further site-differentiated to the [Fe_4S_4(DmpS)$_2$(Im*)$_2$] cluster by treatment with an additional equivalent of Im* (Fig. 3C). Alternatively, [Fe_4S_4(DmpS)$_2$(Im*)$_2$] can be synthesized from all-ferric [Fe_4S_4(DmpS)$_4$], upon treatment with 2 equivalents of Im* (Fig. 3C). Treatment of [Fe_4S_4(DmpS)$_2$(Im*)$_2$] with stoichiometric amounts of DmpSK causes substitution of the Im* ligands, restoring the canonical [Fe_4S_4]$^{2+}$ cubane, K_2[Fe_4S_4(DmpS)$_4$], *via* K[Fe_4S_4(DmpS)$_3$(Im*)] as intermediate (Supplementary Fig. 57).
All the site-differentiated cubane clusters present ^{1}H NMR (and ^{13}C NMR, where applicable) spectra, accounting for the protons on all ligands (Supplementary Figs. 32-38 and 41-45). Additionally, all Fe_4S_4 complexes, save for $^{[2.2.2]}$K[Fe_4S_4(DmpS)$_3$(Im*)], have been structurally characterized in the solid state by single-crystal X-ray diffraction analyses (Supplementary Figs. 77-80). Between [Fe_4S_4(DmpS)$_4$]$^{2–}$, [Fe_4S_4(DmpS)$_3$(Im*)]$^–$ and [Fe_4S_4(DmpS)$_2$(Im*)$_2$], sequential Im* substitution, decreases the net extinction of the UV-vis electronic absorption spectrum as it concomitantly becomes broader and absorbs over a wider energy range (Supplementary Fig. 15A). Thereby, however, the energy position of the extinction maxima appears to remain constant. Between [Fe_4S_4(DmpS)$_4$]$^–$ and [Fe_4S_4(DmpS)$_3$(Im*)], The UV-vis electronic absorption spectra are similar, but the main extinction maximum redshifts from 469 nm in [Fe_4S_4(DmpS)$_4$]$^–$ to 489 nm in [Fe_4S_4(DmpS)$_3$(Im*)]. Furthermore, cyclic voltammetry evidences that each Im*-substitution of one

of the thiolate ligands shifts the cluster's redox potential by *ca*. +450 mV (Supplementary Figs. 61-68), both for the $[Fe_4S_4]^{2+/3+}$ and the $[Fe_4S_4]^{1+/2+}$ redox couples. In analogy to our previous work, the voltammograms appear different in presence and absence of K^+ cations in the electrolyte, as we demonstrate, for example for $[Fe_4S_4(DmpS)_3(Im^*)]^{0/1-}$ (Supplementary Fig. 64).
Among all $[Fe_4S_4]^{n+}$ oxidation states, the all-ferric one appeared to hold a special place, because it exhibited a broad array of reactivity: As detailed in the following sections, a variety of conditions involving the presence of neutral, weak N-donor molecules (besides Im*) leads to reductive site-differentiation, cluster scission, FeS cluster aggregation, or even the explicitly observed cluster disruption *via* Fe-removal from the cubane core. Accordingly, these transformations are schematically summarized in Supplementary Fig. 5.

The [8Fe-8S] clusters

Reduction of $K[Fe_4S_4(DmpS)_3(Im^*)]$ over 1 equivalent of KC_8 results in the formation of the edge-bridged $[Fe_8S_8]^{2+}$ double-cubane, $K_4[Fe_8S_8(DmpS)_6]$ (***ebdc***, Figs. 3C and 4A) in which the $[Fe_4S_4]^{1+}$ complex preferably undergoes self-fusion rather than maintaining its 3:1 site-differentiated structure with a bound Im* ligand. ***ebdc*** is characterized by a poorly resolved ^1H NMR spectrum, both in THF-*d*8, and in C_6D_6, suggesting a dynamic behavior in solution as well as strong paramagnetism and its UV-vis electronic absorption spectrum shows a nearly featureless rise in extinction at lower wavelength, with two discernible shoulders at 405 and 336 nm (Fig. 5A). In the solid state, ***ebdc*** crystallizes in the space group $P\bar{1}$, containing two cubanes linked *via* one of their respective edges. Both are coordinated by three $DmpS^-$ ligands, yet they display distinct coordination environments in the second sphere: One of the cubanes maintains close contacts with three K^+ ions (K2, K3, K4), whereas the other interacts closely with only one K^+ ion (K1) and shares another (K2) with the first cubane. The cyclic voltammogram of ***ebdc*** recorded in a 0.1 M solution of $[^nBu_4N][PF_6]$ in *o*-DFB exhibits a large number of quasi-reversible redox waves between –1.88 V and –0.11 V *vs*. Fc/Fc^+ (Supplementary Fig. 69A). The near perfect overlap of two consecutive scans of the voltammogram at all the investigated scan rates (from 10 mV s^{-1} to 1 V s^{-1}) suggests that all observed processes are chemically reversible. By analogy with what we had described for the canonical Fe_4S_4 complexes,[1] the scan rate dependency of the anodic peak potentials of the four oxidative events (Supplementary Fig. 69B) is associated with the kinetics of K^+ ion de-coordination from the structure. In addition, and similar to the behavior of other K^+-ion containing FeS clusters reported by us,[1,2] the voltammogram of ***ebdc*** appears different if recorded in a 0.1 M $K[BArF_{24}]$ solution in THF as supporting electrolyte (Supplementary Fig. 70).
Stirring of a toluene solution of ***ebdc*** over 4 equivalents of $[FeCp_2]PF_6$, followed by fast crystallization of the product from the concentrated mother liquor by layering with pentane, leads to the formation of single-crystals of the ligand-bridged double-cubane, $[Fe_8S_8(DmpS)_6]$ (***lbdc***, Fig. 4B). In the solid state, ***lbdc*** crystallizes in the space group $P2_1/c$, exhibiting two $[Fe_4S_4]^{3+}$ cubanes bridged *via* two $\mu^2(DmpS^-)$ ligands (Fig. 4B). The four-electron oxidation is reversible, as demonstrated by treating ***lbdc*** with 4.1 equivalents of KC_8, which results in the regeneration of ***ebdc*** (Fig. 3C). In C_6D_6 solution, the ***lbdc***'s bridging ligands are clearly distinguishable by their ^1H NMR spectroscopic signature which differs from that of the terminal $\mu^1(DmpS^-)$ ligands (Supplementary Fig. 51). This strongly suggests that the ligand-bridged structure is preserved in solution, with the inequivalence of the two sets of signals arising from distinct magnetic environments around the bridging and terminal ligands.[25] Obtaining such a ligand-bridged structure is only possible in toluene, or other non-coordinating solvents. Dissolution of ***lbdc*** in THF immediately leads to the scission of the double-cubane and the formation of 3:1 site-differentiated $[Fe_4S_4(DmpS)_3(THF)_3]$, in direct analogy with the reactivity observed with the all-ferric cubane $[Fe_4S_4(DmpS)_4]$[26] (Supplementary Fig. 85). ***lbdc*** is further characterized by an electronic absorption spectrum with a large molar extinction

coefficient of its maximum (46.9·10^3 at 471 nm; Fig. 5A), which has a similar energy as that observed for ***lbdc's*** canonical and 3:1 site-differentiated redox congeners.[1] The cyclic voltammogram of ***lbdc***, recorded in *o*-DFB, is very close to that of the canonical all-ferric cubane, [Fe_4S_4(DmpS)$_4$], showing two quasi-reversible features at –1.00 V and –0.18 V as well as a broad current response between them, at –0.52 V, and an irreversible reduction at –2.34 V *vs*. Fc/Fc$^+$ (Supplementary Fig. 71). Notably, our attempts to generate Fe_8S_8 clusters in oxidation states between [Fe_8S_8]$^{2+}$ (that of ***ebdc***) and [Fe_8S_8]$^{6+}$ (that of ***lbdc***) by treating ***ebdc*** with <4 equivalents of oxidant were unsuccessful, as we repeatedly crystallized either one or the other (or a canonical Fe_4S_4) complex from the reaction mixture. This indicates the occurrence of redox disproportionation and ligand-scrambling equilibria. Upon prolonged standing of a dilute toluene solution of ***lbdc*** layered with pentane, single-crystals of the interlocked double-cubane, [Fe_8S_8(DmpS)$_4$] (***ildc***, Figs. 3C and 4C), form as the main product, albeit in low yield (*ca.* 15-40%, depending on the duration of the crystallization process). Relatedly, monitoring a dilute C_6D_6 solution *via* ^{1}H NMR spectroscopy over the course of 10 days shows that it slowly forms ***ildc*** as major product, firmly establishing ***lbdc*** as a metastable intermediate in the pathway to ***ildc*** (Supplementary Figs. 54-56 and Supplementary Note 5). The corresponding ^{1}H NMR spectrum of ***ildc***, recorded in C_6D_6 solution, shows a complex set of signals in the diamagnetic region of the spectrum (Supplementary Fig. 49). This complexity arises from the different coordination-modes of the two sets of DmpS$^-$-ligands: Two of these (S8) coordinate in a μ^1- and two (S6 and S7) in a μ^2-fashion (Fig. 4C). Additional splitting of some of the signals suggests that the rotation of the mesitylene groups is sterically hindered, rendering the *endo*/*exo*-Me protons of the μ^2(DmpS$^-$) ligand inequivalent. In the solid state, ***ildc*** crystallizes in the space group $P2_1/m$, only half of the molecule present in the asymmetric unit. Unfortunately, our efforts to accelerate and promote the formation of ***ildc*** with heat or light irradiation have so far been unsuccessful. Alternative synthetic routes were also explored, starting from [Fe_4S_4(DmpS)$_4$] and [Fe_4S_4(DmpS)$_2$(Im*)$_2$], but they also failed (*vide infra*). Notably, however, we observed that, in analogy to the reduction of K[Fe_4S_4(DmpS)$_3$(Im*)] yielding ***ebdc***, the reduction of [Fe_4S_4(DmpS)$_2$(Im*)$_2$] over 2 equivalents of KC_8 yields an edge-bridged [$Fe_{12}S_{12}$]^0 triple-cubane K_6[$Fe_{12}S_{12}$(DmpS)$_6$] (***ebtc***; Supplementary Fig. 3). This underscores the validity of our proposed general strategy for Fe_4S_4 cluster site-differentiation *via* redox-neutral ligand-loss from reduced complexes (Fig. 3B). Furthermore, while removal of Im* from [Fe_4S_4(DmpS)$_2$(Im*)$_2$] by B(C_6F_5)$_3$ does indeed occur, forming Im*·B(C_6F_5)$_3$ (Supplementary Fig. 87), the resulting putative coordinatively unsaturated [Fe_4S_4(DmpS)$_2$] cubanes do not fuse and interlock in a well-defined manner. Instead, this reaction led to the identification of [$Fe_{24}S_{24}$(DmpS)$_{10}$] among the reaction products, which is—to the best of our knowledge—the largest molecular FeS cluster ever characterized by single-crystal X-ray diffraction (Supplementary Figs. 4 and 88), spanning ca. 2 nm in diameter, and the product of uncontrolled FeS cluster aggregation. Similarly, attempts to generate ***lbdc*** by treating [Fe_4S_4(DmpS)$_3$(Im*)][26] with B(C_6F_5)$_3$ (Supplementary Fig. 17) were also unsuccessful. These failed attempts underscore the importance of the formation of ***lbdc*** from ***ebdc*** in a controlled fashion, and in absence of coordinating molecules, for its subsequent conversion to ***ildc***.

ildc exhibits a UV-vis electronic absorption spectrum, characterized by an intense peak at 467 nm and a shoulder at 631 nm (Fig. 5A). Its cyclic voltammogram recorded in a 0.1 M solution of [^{n}Bu$_4$N][PF$_6$] in DCM exhibits two quasi-reversible currents at –0.58 and –1.19 V *vs*. Fc/Fc$^+$, putatively marking the reductions of the [Fe_8S_8]$^{4+}$ core to the [Fe_8S_8]$^{3+}$ and [Fe_8S_8]$^{2+}$ oxidation states, respectively (Supplementary Fig. 72). Despite this apparent robustness at the electrochemical timescale, ***ildc***'s structural integrity is not maintained upon treatment with a competing ligand: Addition of 4 equivalents of Im* to a toluene solution of ***ildc*** leads to the formation of [Fe_4S_4(DmpS)$_2$(Im*)$_2$] (Fig. 3C and Supplementary Fig. 80), resulting from the "unlocking" and scission of the structure. Relatedly, this cleavage also occurs directly, upon treatment of ***ildc*** with 4

equivalents of DmpSK, resulting in the formation of two equivalents of $K_2[Fe_4S_4(DmpS)_4]$ (Supplementary Fig. 58).

<u>Simulation of [8Fe-8S] Mössbauer spectra</u>

The low- or zero-field 80 K powder spectra of ***ebdc***, ***lbdc*** and ***ildc*** are displayed in Figs. 5C-E as black vertical bars. Whereas two absorption lines are observed for ***ebdc*** and ***lbdc***, four are detected for ***ildc***. To limit the number of unknowns, two doublets were considered to reproduce the spectra in all in simulations.

Due to the large linewidth and the lack of symmetry of ***ebdc*** according to its solid-state structure, we think that two sites are obviously not enough, and simulations assuming four, three or two doublets were all tested; the one shown in Fig. 5E of course being the one assuming two. However, all the simulations present the same average isomer shift value, namely 0.53 mm s^{-1}. This high value indicates a major ferrous character and is indeed similar to those observed for $[Fe_4S_4]^{1+}$ clusters.[1,27-29] To improve the resolution of the individual doublets, we also evaluated the 80 K zero-field spectrum of ***ebdc*** recorded on a ^{57}Fe-enriched sample (>95%) dissolved in toluene (*ca.* 1.5-2 mM). Therein, the line-broadening intermolecular interactions often occurring in the solid-state spectra of magnetic compounds should be more suppressed. Accordingly, the solution spectrum evidenced at least three sites and could be reproduced well with a simulation of nested doublets in a 2:1:1 ratio (Supplementary Fig. 99). The deduced average isomer shift of 0.54 mm s^{-1} is perfectly in line with that obtained from the simulation of the powder spectrum, and the individual δ-values range from 0.45 to 0.58 mm s^{-1}.

Two doublets in a 1:1 ratio allowed to reproduce the spectrum of ***lbdc*** (Fig. 5C). Those displayed in Fig. 5C present isomer shifts that differ by more than 0.1 mm s^{-1}, while a 0.05 mm s^{-1} difference is determined for the other pairing (Supplementary Fig. 100). Similarly to ***ebdc***, whatever the simulation, a 0.39 mm s^{-1} average isomer shift value is deduced. This value indicates a higher oxidation state for this cluster and is consistent with $[Fe_4S_4]^{3+}$ cubanes.[1,30,31] In contrast, the four distinct lines in the ***ildc***'s spectrum can be paired in a single way, forming two equally contributing nested doublets (Fig. 5D) with 0.34 mm s^{-1} and 0.68 mm s^{-1} isomer shift values, respectively. The two intertwined doublets are centered at 0.09 and 0.93 mm s^{-1}, which are unreasonably low and high isomer shift values, respectively, for Fe ions in sulfide-/thiolate-ligated tetrahedral environment. Furthermore, the four 5.7 K spectra reproduced in Supplementary Figs. 102A-D were simultaneously simulated using a homemade program. In analogy to the simulation of the zero-field 80 K spectrum, two diamagnetic Fe sites were considered in a 1:1 ratio. It can be noticed that the line positions are satisfyingly reproduced but not the intensities. We thus suspected the contribution of a close lying paramagnetic excited state. Accordingly, four Fe sites with fictitious 1/2-electronic spin and interacting by pairs were considered to reproduce the spectra recorded at 2, 4 and 7 T. The same anisotropic exchange interaction was assumed in the two pairs in order that the four sites experienced the same spin ladder ($\widehat{S_a}\,\widetilde{J_{ab}}\,\widehat{S_b}$ convention). Anisotropy then allowed introducing a ZFS effect in the excited S=1 state. To reduce the number of unknowns, isotropic hyperfine interactions were considered. This hypothesis led to significant improvement of the simulation of the 7 T-spectrum, and only to a marginal one at lower field (Supplementary Figs. 102E-H). Therefore, while this model sustained the hypothesis of magnetic contributions, it may still be too restricted to reproduce all the observed features.

Synthetic Procedures

DmpSK

In an Ar-filled glovebox, solid KH (85.4 mg, 2.13 mmol, 0.95 equiv.) was added to a stirred solution of DmpSH (752.0 mg, 2.17 mmol, 1.00 equiv.) in toluene (20 mL) in a 25 mL scintillation vial at room temperature. Immediately, the mixture turned cloudy and H_2 evolution ensued. While venting the formed gas from the loosely capped vial into the Ar-atmosphere of the glovebox, stirring was continued until no more gas evolution could be observed (*ca.* 2 h). Subsequently, the suspension was placed in the –35°C freezer overnight. On the next day, the colourless precipitate was filtered off by means of a frit and washed with 2x4 mL of cold toluene. Drying of the white powder *in vacuo* yielded 769.0 mg (1.99 mmol, 92%) of the analytically pure product.
Single crystals suitable for X-ray diffraction were obtained upon prolonged standing of a THF/pentane (1:1) solution of the compound (10 mg in 3 mL) at –35°C.

^{1}H NMR (300 MHz, THF-*d*8): δ [ppm] 6.87 (s, 4H), 6.66 (dd, *J*=8.1,6.3 Hz, 1H), 6.55 (d, *J*=6.8 Hz, 2H), 2.39 (s, 6H), 2.31 (s, 12H).
^{13}C NMR (75 MHz, THF-*d*8): δ [ppm] 154.8, 146.1, 143.5, 137.8, 134.9, 127.9, 127.1, 118.7, 21.7, 20.7.

$DmpS^{[18\text{-}C\text{-}6]}K$

A THF solution (4 mL) of DmpSK (201.6 mg, 0.524 mmol, 1.0 equiv.) was combined with a THF solution (4 mL) of 18-crown-6 (139.2 mg, 0.527 mmol, 1.0 equiv.) while vigorously stirring. Immediately, a colourless precipitate formed, and stirring was continued for 30 minutes. Subsequently, the mixture was concentrated to about half its original volume and stored in the –35°C freezer for 1 h. The colourless precipitate was decanted, washed with diethyl ether (2x2 mL) and dried in vacuo, yielding 267.0 mg (0.371 mmol, 71%) of product.
Single crystals suitable for X-ray diffraction analysis were grown by storing a saturated THF/diethyl ether solution (*ca.* 2:1) at –35°C.

^{1}H NMR (300 MHz, THF-*d*8): δ [ppm] 7.47 (t, *J*=7.6 Hz, 1H), 7.13 (d, *J*=7.6 Hz, 2H), 6.95 (s, 4H), 3.62 (s, 24H), 2.32 (s, 6H), 2.06 (s, 12H).
^{13}C NMR (75 MHz, THF-*d*8): δ [ppm] 146.0, 138.0, 137.5, 136.3, 130.5, 130.2, 129.5, 128.6, 70.7, 21.2, 20.8.

$(DmpS)_2$

Solid $[FeCp_2]PF_6$ (130.5 mg, 0.394 mmol, 1.0 equiv.) was added to a stirred THF solution (5 mL) of DmpSK (149.0 mg, 0.387 mmol, 1.0 equiv.) at room temperature, and stirred until the next day. The mixture was filtered, and the light-yellow filtrate was concentrated to 2 mL. The solution was layered with double its volume of pentane and stored at –35°C. After two days, the product was collected as light-yellow crystals, which were of diffraction quality, and were decanted, washed with pentane and dried in vacuo, yielding 67 mg (0.097 mmol, 50%) $(DmpS)_2$.

^{1}H NMR (300 MHz, THF-*d*8): δ [ppm] 7.18 (t, *J*=7.5 Hz, 1H), 6.81 (d, *J*=7.5 Hz, 2H), 6.75 (s, 4H), 2.28 (s, 6H), 1.67 (s, 12H).

^{13}C NMR (75 MHz, THF-*d*8): δ [ppm] 144.4, 138.7, 137.4, 137.4, 137.2, 130.4, 128.8, 128.3, 21.4, 21.0.

[$Fe_2S_2(DmpS)_2$(py)] and its interconversions

[$Fe_4S_4(DmpS)_4$] (151.3 mg, 0.080 mmol, 1.0 equiv.) was dissolved in neat pyridine (4 mL) and stirred for 20 minutes. The dark purplish red mixture was subsequently filtered, separating a small amount of dark solid, and the solution was layered with a copious amount of pentane. After 2 days, a large amount of thin elongated block shaped crystals had formed, which were decanted, washed with pentane (2x2 mL) and dried *in vacuo*, yielding 145.1 mg (0.061 mmol, 77%) of [$Fe_2S_2(DmpS)_2(py)_2$] as the desired product.
The obtained product crystals were single crystals of X-ray diffraction quality, so some of them were separated from the bulk prior to drying and placed in a puddle of perfluoropolyalkylether oil on a microscope slide.

^{1}H NMR (300 MHz, CD_2Cl_2): δ [ppm] 12.19 (bs, 2H), 9.86 (bs, 2H), 6.63 (s, 4H), 6.53 (bs, 1H), 3.25 (bs, 1H), 2.33 (s, 6H), 2.17 (bs, 12H).
^{1}H NMR (300 MHz, C_6D_6): δ [ppm] 11.35 (bs, 2H), 9.89 (bs, 2H), 6.55 (s, 4H), 5.54 (bs, 1H), 3.40 (s, 1H), 2.44 (bs, 12H), 2.18 (s, 6H).
UV-vis ($1 \cdot 10^{-4}$ M in toluene): λ [nm] (ε [10^3 M^{-1} cm^{-1}]) 351 (sh, 12.0), 394 (p, 16.8), 450 (p, 14.9), 510 (p, 14.4), 595 (sh, 9.6), 722 (sh, 1.7).
Elemental analysis: ($C_{58}H_{60}Fe_2N_2S_4 \cdot 2C_5H_5N$) expected: C 69.02%, H 5.96%, N 4.74%; found: C 68.93%, H 5.83%, N 4.76%.

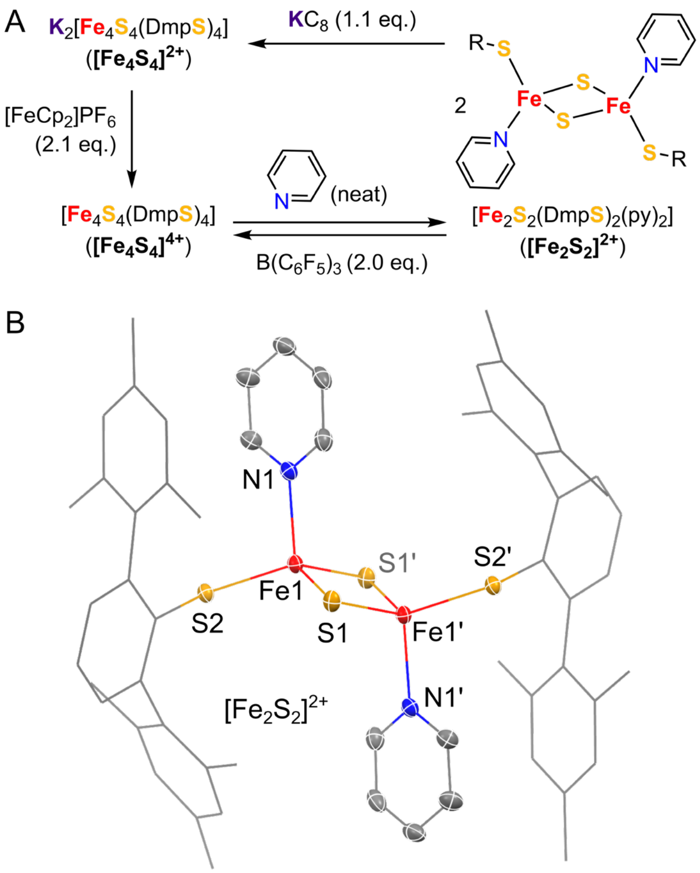

Supplementary Figure 1. (A) Synthesis and interconversion reactions of $[Fe_2S_2(DmpS)_2(py)_2]$. (B) Solid-state molecular Structure of $[Fe_2S_2(DmpS)_2(py)_2]$ in crystals of $[Fe_2S_2(DmpS)_2(py)_2]\cdot py_2$. Displacement ellipsoids are displayed at the 50% probability level only for Fe, S and the pyridine ligands. Co-crystallized solvent and H-atoms were omitted for clarity.

Conversion back to $[Fe_4S_4(DmpS)_4]$: A J. Young NMR tube was charged with 10.1 mg (0.008 mmol, 1.0 equiv.) of $[Fe_2S_2(DmpS)_2(py)_2]$ dissolved in CD_2Cl_2, and an NMR spectrum was recorded in order to confirm the spectroscopic purity of the sample. Subsequently the tube was reintroduced into the glovebox, where it was charged with an additional 17.3 mg (0.034 mmol, 4.1 equiv.) $B(C_6F_5)_3$. Immediately after the addition, the color of the mixture changed from dark red to dark blueish black—the typical color of the all-ferric cubane.[1] Successful and quantitative formation of $[Fe_4S_4(DmpS)_4]$ was confirmed by comparison of the spectroscopic signature of the NMR spectrum recorded after addition of $B(C_6F_5)_3$ (Supplementary Fig. 30) with that of a neat sample of $[Fe_4S_4(DmpS)_4]$ (Supplementary Fig. 31).
Furthermore, the very same reaction was monitored *via* UV-vis electronic absorption spectroscopy, evidencing that upon addition of 4 or more equivalents of $B(C_6F_5)_3$, the spectrum of $[Fe_4S_4(DmpS)_4]$ is obtained in a quantitative ratio with respect to the starting concentration of $[Fe_2S_2(DmpS)_2(py)_2]$ (Supplementary Fig. 7A). Note that we were not able to perform the reverse reaction, *i.e.* forming $[Fe_2S_2(DmpS)_2(py)_2]$ from $[Fe_4S_4(DmpS)_4]$ with stoichiometric amounts of pyridine cleanly. The UV-vis traces of the two reactions are not superimposable (Supplementary Fig. 7B), and the NMR spectra of $[Fe_4S_4(DmpS)_4]$ after addition of 4 or 20 equivalents of pyridine (Supplementary Figs. 28 and 29) evidence that $[Fe_2S_2(DmpS)_2(py)_2]$ is formed as one of the major products, judging by the characteristic peaks at 12.19 and 9.86 ppm, but it is not the exclusive product of the reaction. Likely there is a complex chemical equilibrium in solution, that may involve other site-differentiated Fe_4S_4, Fe_2S_2, or even higher-nuclearity and disrupted clusters, like we observed upon reaction the all-ferric cubane with similar N-donor ligands (*vide infra*).

Reduction to $K_2[Fe_4S_4(DmpS)_4]$: KC_8 (8.2 mg, 0.061 mmol, 1.0 equiv.) was added to a solution of $[Fe_2S_2(DmpS)_2(py)_2]$ (70.1 mg, 0.059 mmol, 1.0 equiv.) in toluene (5 mL), and the resulting mixture

was stirred for 1 h. Subsequently, it was filtered and set for crystallization by layering the mother liquor with copious amounts of HMDSO at room temperature. After 1 week, large single-crystals of $K_2[Fe_4S_4(DmpS)_4]$ were decanted, washed with pentane and dried, yielding 40.2 mg (0.019 mmol, 67%) of product. Its identity was confirmed by single-crystal X-ray diffraction, as well as ^1H NMR spectroscopy, which was both in line with our previously reported data for this compound.
Note that the analogous reaction could be performed with 0.5 equiv. of KC_8 as the reductant. Also in this case, we observed the formation of single-crystals of $K_2[Fe_4S_4(DmpS)_4]$, rather than its singly-oxidized congener, $K[Fe_4S_4(DmpS)_4]$, albeit in much lower yield.

$K[Fe_4S_4(DmpS)_3(Im^*)]$

Standard procedure - Method A): In an Ar-filled glovebox, solid $[FeCp_2]PF_6$ (159.9 mg, 0.483 mmol, 1.00 equiv.) was added to a stirred solution of $K_2[Fe_4S_4(DmpS)_4]$ (1000.2 mg, 0.479 mmol, 1.00 equiv.) in toluene (40 mL) in a 100 mL Schlenk flask. Already after a short period of time, the colour of the reaction mixture changed from dark yellowish brown to a dark purplish brown, and stirring was continued for 4 h. Afterwards, the mixture was filtered, separating a small amount of a dark solid, and the filtrate was treated with a solution of 1,2,4,5-tetramethylimidazole (Im*; 60.0 mg, 0.483 mmol, 1.00 equiv.) in toluene (4 mL). After addition, the colour immediately changed back to a dark yellowish brown and stirring was continued for an additional 2 h. Subsequently, the mixture was concentrated to roughly 5 mL, filtered again and layered with a generous amount of pentane for crystallization. After 4 days, the product was isolated as black aggregates of microcrystallites which were decanted, washed with portions of pentane until the washings turned colourless, and dried *in vacuo*, yielding 546.3 mg $K[Fe_4S_4(DmpS)_3(Im^*)]$ (0.352 mmol, 73%). Single-crystals suitable for X-ray diffraction analysis were grown by prolonged undisturbed standing of a dilute solution of the compound in a toluene/pentane mixture (*ca.* 1:1) at ambient temperature.

Standard procedure - Method B): A solution of Im* (13.3 mg, 0.107 mmol, 1.1 equiv.) in toluene was dropwise added to a stirred solution of $K[Fe_4S_4(DmpS)_4]$ (182.0 mg, 0.098 mmol, 1.0 equiv.) in toluene (4 mL), whereupon the color immediately changed from dark purplish black to a dark yellowish brown. Stirring was continued for 2 h. Subsequently, the mixture was concentrated to about 2 mL, filtered, and layered with a copious amount of pentane. Diffusion of the phases at room temperature yielded 98.0 mg (0.063 mmol, 65%) of $K[Fe_4S_4(DmpS)_3(Im^*)]$ as aggregates of microcrystals after 3 days, which were decanted, washed with pentane and dried.

From $[Fe_4S_4(DmpS)_3(Im^*)]$: This reaction was carried out on an NMR scale. To this end, $[Fe_4S_4(DmpS)_3(Im^*)]$ (16.9 mg, 0.011 mmol, 1.0 equiv.) were dissolved in C_6D_6 in an NMR tube equipped with a J. Young valve. A spectrum of the starting material was measured in order to confirm its spectroscopic purity. Subsequently, the solution was poured onto a slurry of KC_8 (1.7 mg, 0.013 mmol, 1.2 equiv.) in a small amount of C_6D_6 and the mixture was taken-up into a Pasteur pipette a couple of times for mixing. Subsequently, it was pressed through a filter plug back into the J. Young NMR tube, thereby removing the graphite, and a spectrum was recorded (Supplementary Fig. 40). While the data clearly evidences the formation of $K[Fe_4S_4(DmpS)_3(Im^*)]$ as the major product, numerous other peaks appeared throughout the spectrum, likely evidencing the formation of ***ebdc***, or related products due to overreduction of the cubane beyond the $[Fe_4S_4]^{2+}$ oxidation state (*vide infra*). Nonetheless, the reversibility of the currents observed *via* cyclic voltammetry (Supplementary Figs. 61, 62 and 64-66) for this redox reaction are further evidence supporting its reversibility.

^1H NMR (300 MHz, C_6D_6): δ [ppm] 8.33 (d, *J*=6.6 Hz, 6H), 7.01 (s, 12H), 6.06 (bs, 3H), 4.76 (bs, 3H), 3.31 (s, 3H), 2.77 (s, 3H), 2.58 (bs, 36H), 2.42 (s, 18H), 1.97 (s, 3H).
^1H NMR (300 MHz, CD_2Cl_2): δ [ppm] 8.20 (d, *J*=6.8 Hz, 6H), 6.98 (s, 12H), 6.14 (bs, 3H), 5.02 (bs, 3H), 4.72 (s, 3H), 2.87 (s, 3H), 2.50 (s, 18H), 2.23 (bs, 39H).
UV-vis ($1\cdot10^{-4}$ M in toluene): λ [nm] (ε [10^3 M^{-1} cm^{-1}]) 341 (p, 18.9), 401 (p, 18.9).

Elemental analysis: ($C_{79}H_{87}Fe_4KN_2S_7{\cdot}1.5C_5H_{12}$) expected: C 62.60%, H 6.38%, N 1.69%; found: C 62.50%, H 6.45%, N 1.55%.

[Fe_4S_4(DmpS)$_3$(Im*)]

For the synthesis of [Fe_4S_4(DmpS)$_3$(Im*)], K[Fe_4S_4(DmpS)$_3$(Im*)] (116.0 mg, 0.075 mmol, 1.0 equiv.) was dissolved in toluene (10 mL) in a 20 mL scintillation vial at room temperature in an Ar-filled glovebox. To this, while stirring, solid [$FeCp_2$]PF_6 (27.7 mg, 0.083 mmol, 1.10 equiv.) were added and the mixture stirred for 2 h. During this time, the colour changed from a dark yellowish brown to a dark blue/purplish black. Afterwards, the mixture was filtered, the filtrate was concentrated to approximately 2 mL, filtered again and layered with a copious amount of HMDSO for crystallization. After 3 days, the product was collected as black needle-shaped crystals, which were decanted, washed with HMDSO until the washings turned colourless and dried *in vacuo*, yielding 100.6 mg (0.063 mmol, 84%) of [Fe_4S_4(DmpS)$_3$(Im*)]. The characterization data is congruent with that found in the literature.[26] Note that no ^{13}C resonances of the Im* ligand were found.
The isolated crystals were suitable for X-ray diffraction analysis, so some of them were separated from the bulk of the product prior to drying and suspended in a puddle of perfluoropolyalkylether oil, and the identity of the product was further confirmed by its solid-state molecular structure (Supplementary Fig. 77). We report this structure, because owing to the synthesis and crystallization conditions used by us, the unit cell appears to have slightly different parameters than the one previously reported.[26]

^{1}H NMR (300 MHz, C_2Cl_2): δ [ppm] 14.74 (bs, 3H), 11.20 (s, 3H), 8.16 (d, *J*=7.3 Hz, 6H), 7.07 (s, 3H), 6.81 (s, 12H), 6.43 (t, *J*=7.2 Hz, 3H), 2.49 (s, 18H), 2.41 (s, 36H), 1.78 (bs, 3H).
^{1}H NMR (300 MHz, toluene-*d*8): δ [ppm] 13.16 (bs, 3H), 9.03 (s, 3H), 8.32 (d, *J*=7.3 Hz, 6H), 6.83 (s, 12H), 5.99 (t, *J*=7.3 Hz, 1H), 5.52 (s, 3H), 2.72 (s, 36H), 2.47 (s, 18H), 1.78 (bs, 3H).
^{13}C NMR (75 MHz, toluene-*d*8): δ [ppm] 175.8, 143.3, 142.3, 140.8, 138.1, 137.2, 134.0, 130.8, 36.6, 18.4.
^{1}H NMR (300 MHz, C_6D_6): δ[ppm] 13.27 (bs, 3H), 8.98 (s, 3H), 8.40 (d, *J*=7.3 Hz, 6H), 6.88 (s, 12H), 6.03 (t, *J*=7.3 Hz, 3H), 5.50 (s, 3H), 2.78 (s, 36H), 2.48 (s, 18H), 1.64 (bs, 3H).
^{13}C NMR (75 MHz, C_6D_6): δ[ppm] 175.7, 142.8, 141.9, 139.6, 133.9, 132.7, 130.5, 129.3, 35.2, 32.0, 21.4, 20.7, 17.8.
UV-vis ($1{\cdot}10^{-4}$ M in toluene): λ [nm] (ε [10^3 M^{-1} cm^{-1}]) 345 (sh, 20.1), 489 (p, 25.4).
Elemental analysis: ($C_{79}H_{87}Fe_4N_2S_7$) expected: C 62.74%, H 5.80%, N 1.85%; found: C 62.90%, H 5.92%, N 1.89%.

$^{[2.2.2]}$K[Fe_4S_4(DmpS)$_3$(Im*)]

In an Ar-filled glovebox, K[Fe_4S_4(DmpS)$_3$(Im*)] (186.0 mg, 0.120 mmol, 1.0 equiv.) was dissolved in toluene (20 mL) and filtered to remove any insoluble impurities. Upon the addition of [2.2.2]-cryptand (*i.e.* 4,7,13,16,21,24-hexaoxa-1,10-diazabicyclo[8.8.8]hexacosane; 50.82 mg, 0.135 mmol, 1.1 equiv.) as a solid to the stirred solution, precipitation of a black solid on the vial walls ensued and stirring was continued for 30 min. The liquid was decanted and the precipitate was slurried and washed with pentane (2x2 mL) before being dried *in vacuo*. As the precipitate was still sticking to the vial walls, it was slightly crushed before being washed again with toluene (2x2mL) and subsequently pentane (2x2 mL). After drying *in vacuo*, 87.3 mg (0.043 mmol, 36%) of a black solid was obtained.
Unfortunately, our attempts to recrystallize the product, as well as direct crystallization from the reaction failed to produce single-crystals suitable for X-ray diffraction analysis.

^{1}H NMR (300 MHz, CD_2Cl_2): δ [ppm] 8.18 (d, *J*=6.8 Hz, 6H), 6.81 (s, 12H), 5.85 (bs, 3H), 4.43 (s, 3H), 3.93 (bs, 3H), 3.57 (s, 12H), 3.51 (m, 12H), 2.63 (s, 3H), 2.52 (m, 12H), 2.29 (bs, 54H), 1.86 (bs, 3H).
UV-vis ($1\cdot10^{-4}$ M in DCM): λ [nm] (ε [10^3 M^{-1} cm^{-1}]) 340 (sh, 18.0), 430 (sh, 15.5).
Elemental analysis: ($C_{97}H_{123}Fe_4KN_4O_6S_7\cdot C_7H_8$) expected: C 61.84%, H 6.54%, N 2.77%; found: C 61.94%, H 6.66%, N 2.69%.

$^{[18\text{-}C\text{-}6]}$K[$Fe_4S_4$(DmpS)$_3$(Im*)]

In an Ar-filled glovebox, K[Fe_4S_4(DmpS)$_3$(Im*)] (203.5 mg, 0.131 mmol, 1.0 equiv.) was dissolved in toluene (20 mL). While stirring, 18-crown-6-ether (38.9 mg, 0.147 mmol, 1.1 equiv.) was added as a solid and stirring continued for 60 min. The solution was filtered and concentrated (to approx. 4 mL) before being layered with pentane (20 mL) for crystallization. After 3 days, shiny, medium sized black crystals had formed. The mother liquor was decanted off and the crystals washed with pentane (2x5 mL) before being dried *in vacuo*, yielding 207.0 mg (1.09 mmol, 83%) of brown-black crystals. Single-crystals suitable for X-ray diffraction analysis were grown by direct crystallization from the filtered reaction mixture. To this end, vapor diffusion with pentane as the volatile antisolvent was utilized, yielding aggregates, which separated into arrowhead-shaped single-crystals when crushed.

^{1}H NMR (300 MHz, CD_2Cl_2): δ [ppm] 8.17 (d, *J*=6.8 Hz, 6H), 6.80 (s, 12H), 5.87 (bs, 3H), 4.45 (s, 3H), 3.95 (bs, 3H), 3.59 (s, 24H), 2.64 (s, 3H), 2.29 (s, 18H), 2.25 (bs, 36H), 1.85 (bs, 3H).
UV-vis ($1\cdot10^{-4}$ M in DCM): λ [nm] (ε [10^3 M^{-1} cm^{-1}]) 340 (sh, 20.1), 430 (sh, 17.7).
Elemental analysis: ($C_{91}H_{111}Fe_4KN_2O_6S_7\cdot0.9\ C_7H_8$) expected: C 61.55%, H 6.28%, N 1.48%; found: C 61.47%, H 6.33%, N 1.53%.

[Fe_4S_4(DmpS)$_2$(Im*)$_2$]

Standard Procedure - Method A): Solid [$FeCp_2$]PF_6 (101.9 mg, 0.308 mmol, 2.2 equiv.) was added to a solution of K_2[Fe_4S_4(DmpS)$_4$] (290.3 mg, 0.139 mmol, 1.0 equiv.) in toluene, and the mixture was stirred for 3 h. During this time, the mixture changed colour from dark yellowish brown to dark blueish black. Subsequently, the mixture was filtered, and a solution of Im* (40.3 mg, 0.324 mmol, 2.2 equiv.) in toluene was dropwise added while stirring. Afterwards, stirring was continued for another 1 h. The resulting dark brown mixture was taken to dryness completely. Then, the residue was extracted into 1,2-difluorobenzene and (DmpS)$_2$ was separated from the mixture as a pale-yellow powder by filtration. The solution was again taken to dryness and the residue was re-dissolved in 3 mL THF, filtered, and layered with a generous amount of pentane. After 3 days, the product was isolated as large, elongated block-shaped crystals, which were decanted, washed with pentane and dried, yielding 120.2 mg (0.088 mmol, 63%) of [Fe_4S_4(DmpS)$_2$(Im*)$_2$].

Standard Procedure - Method B): A toluene solution of Im* (52.6 mg, 0.424 mmol, 2.2 equiv.) was dropwise added to a toluene solution (5 mL) of [Fe_4S_4(DmpS)$_4$] (360.7 mg, 0.190 mmol, 1.0 equiv.) while stirring at room temperature. Upon addition of Im*, the colour immediately changed from a dark blueish black to dark brown, and stirring was continued for 2 h. Afterwards, the mixture was taken to dryness. The residue was extracted into 1,2-difluorobenzene and (DmpS)$_2$ was separated from the mixture as a pale-yellow powder by filtration. Subsequently, the solution was again taken to dryness, and the residue was re-dissolved in THF (3 mL). Next, the resulting dark brown solution was filtered before being layered with pentane for crystallization. After 3 days, the product was isolated as large, elongated block-shaped crystals, which were decanted, washed with pentane and dried, yielding 219.4 mg (0.161 mmol, 84%) of [Fe_4S_4(DmpS)$_2$(Im*)$_2$]. Single-crystals suitable for X-ray diffraction analysis were grown by vapor diffusion of pentane into a THF solution of the compound at room temperature.

From [Fe_4S_4(DmpS)$_3$(Im)]:* This reaction was carried out on an NMR scale, in order to monitor the product formation *in-situ*. To this end, a J. Young NMR tube was loaded with 16.9 mg (0.011 mmol, 1.0 equiv.) of [Fe_4S_4(DmpS)$_3$(Im*)], and a pristine spectrum was recorded to confirm the pure starting material. Subsequently, solid Im* (1.5 mg, 0.012 mmol, 1.1 equiv.) was added to the tube and the mixture was shaken vigorously. ^{1}H NMR spectroscopy, conducted on the crude reaction mixture evidences the quantitative conversion of the 3:1 site-differentiated cubane to its 2:2 site-differentiated derivative (Supplementary Fig. 39), because the arising peaks coincide with those established for a pure, independently synthesized sample of [Fe_4S_4(DmpS)$_2$(Im*)$_2$]. Furthermore, the anticipated formation of stoichiometric amounts of the disulfide, (DmpS)$_2$, was observed.
A similar conclusion can be drawn from the corresponding UV-vis titration experiment, where the characteristic trace of [Fe_4S_4(DmpS)$_2$(Im*)$_2$] emerges upon sequential addition of Im* to a solution of [Fe_4S_4(DmpS)$_3$(Im*)] in toluene (Supplementary Fig. 18).

*From [Fe_8S_8(DmpS)$_4$] (**ildc**):* [Fe_8S_8(DmpS)$_4$] (20.0 mg, 0.009 mmol, 1.0 equiv.) was suspended in toluene (7 mL), and stirred at high rpm. To this, a solution of Im* (5.2 mg, 0.040 mmol, 4.1 equiv.) in toluene (3 mL) was added and the resulting mixture was stirred overnight. During this time, most solids dissolved, while the color remained a dark yellowish brown. On the next day, the reaction mixture was concentrated to ca. 5 mL, filtered, and layered with pentane (ca. 8 mL). After three days, dark thin plate-shaped crystals of the product, [Fe_4S_4(DmpS)$_2$(Im*)$_2$] were decanted, washed with pentane and dried, yielding 14.6 mg (0.011 mmol, 56%) of product.
Single-crystals suitable for X-ray diffraction analysis were grown by vapor diffusion of pentane into a THF solution of the product at room temperature. ^{1}H NMR and UV-vis electronic absorption spectroscopy were used to establish the identity of the bulk, evidencing spectra which were congruent with those measured on the compound isolated by the standard procedure.

^{1}H NMR (300 MHz, CD_2Cl_2): δ [ppm] 8.44 (d, J=6.7 Hz, 2H), 6.82 (s, 4H), 6.04 (bs, 1H), 4.61 (s, 3H), 4.37 (bs, 3H), 2.82 (s, 3H), 2.36 (s, 6H), 2.29 (bs, 12H).
^{13}C NMR (75 MHz, CD_2Cl_2): δ [ppm] 179.6, 140.5, 137.0, 134.4, 131.5, 130.7, 129.3, 32.2, 29.1, 28.1, 26.3, 21.2, 10.4.
^{1}H NMR (300 MHz, C_6D_6): δ [ppm] 8.77 (d, J=6.7 Hz, 2H), 6.56 (bs, 3H), 6.04 (bs, 1H), 4.27 (bs, 3H), 3.20 (s, 3H), 2.77 (bs, 12H), 2.46 (s, 6H), 1.89 (s, 3H).
UV-vis ($1\cdot10^{-4}$ M in toluene): λ [nm] (ε [10^3 M^{-1} cm^{-1}]) 340 (sh, 18.5), 448 (sh, 25.4).
Elemental analysis: ($C_{62}H_{74}Fe_4N_4S_6\cdot C_4H_8O\cdot 0.5C_5H_{12}$) expected: C 58.80%, H 6.34%, N 4.00%; found: C 58.78%, H 6.48%, N 3.97%.

<u>K_4[Fe_8S_8(DmpS)$_6$] (***ebdc***)</u>

Standard procedure: KC_8 (18.0 mg, 0.133 mmol, 1.0 equiv.) was added as a solid to a solution of K[Fe_4S_4(DmpS)$_3$(Im*)] (200.1 mg, 0.129 mmol, 1.0 equiv.) in toluene (8 mL) at room temperature, while stirring. A subtle colour change from dark yellowish brown to a less yellowish dark brown occurred and stirring was continued for 1 h. Subsequently, the mixture was concentrated to approximately 4-5 mL, filtered, and layered with a generous amount of pentane. After 1 week, small black crystallites were decanted, washed with pentane until the washings turned colourless, and dried *in vacuo*, yielding 158.8 mg (0.049 mmol, 76%) of K_4[Fe_8S_8(DmpS)$_6$] as the desired product.
Single crystals suitable for X-ray diffraction analysis were grown by vapor diffusion of pentane into a dilute toluene solution of the compound at room temperature.
^{1}H NMR spectra of this compound, recorded in C_6D_6 and THF-*d*8, respectively, are shown below (Supplementary Figs. 46-S48), but a peak assignment was not possible.

From [Fe_8S_8(DmpS)$_6$]: A freshly lyophilized sample of [Fe_8S_8(DmpS)$_6$] (30.2 mg, 0.010 mmol, 1.0 equiv.) was dissolved in toluene (5 mL) and KC_8 (5.5 mg, 0.041 mmol, 4.1 equiv.) was added as a

solid. The solution immediately turned from dark purplish to dark yellowish brown, and stirring was continued for 30 minutes. Afterwards, graphite was filtered off, and the mother liquor was layered with pentane. After 1 week, 12.3 mg (0.004 mmol, 38%) of product were collected as a black, microcrystalline solid. Larger single-crystals of $K_4[Fe_8S_8(DmpS)_6]$, were grown as described above, from a dilute sample of the mother liquor, *via* vapor diffusion of pentane. The identity of the product was thus confirmed by single-crystal X-ray diffraction, as well as UV-vis electronic absorption spectroscopy (of the bulk microcrystalline sample).

UV-vis ($1\cdot10^{-4}$ M in toluene): λ [nm] (ε [10^3 M^{-1} cm^{-1}]) 339 (sh, 23.7), 399 (sh, 23.0).
UV-vis ($1\cdot10^{-4}$ M in THF): λ [nm] (ε [10^3 M^{-1} cm^{-1}]) 336 (sh, 24.5), 405 (p, 26.3), 572 (sh, 10.8).
Elemental analysis: ($C_{144}H_{150}Fe_8K_4S_{14}\cdot0.5C_5H_{12}$) expected: C 59.27%, H 5.30%; found: C 59.17%, H 5.33%.

$[Fe_8S_8(DmpS)_4]$ (***ildc***)

In a typical preparation, $[FeCp_2]PF_6$ (42.4 mg, 0.128 mmol, 4.1 equiv.) was added as a solid to a stirred solution of $K_4[Fe_8S_8(DmpS)_6]$ (100.0 mg, 0.031 mmol, 1.0 equiv.) in toluene (8 mL) at room temperature. Over a time period of several hours, a gradual colour change to dark purplish brown occurred, which we would typically associate with an $[Fe_4S_4]^{3+}$ oxidation state.[1] After 6-8 h, the mixture was concentrated to approximately half its original volume (4-5 mL), and filtered. The solution was then layered with double its volume of pentane and left to stand in an undisturbed location. After 10 days, small black crystals of $[Fe_8S_8(DmpS)_4]$ formed, which were decanted and washed with DCM (ca. 4 mL), toluene (ca. 8 mL) and pentane (ca. 4 mL) until the washings turned completely colourless, or slightly yellowish-brown. Thereby, ***Ibdc***, and small amounts of $K[Fe_4S_4(DmpS)_4]$ which both have the characteristic colour of the $[Fe_4S_4]^{3+}$ cubane were washed off. The crystals were finally dried *in vacuo*, yielding 19.3 mg (0.009 mmol, 30%) of the desired product.
The obtained product crystals were single crystals of X-ray diffraction quality, so some of them were separated from the bulk prior to drying and placed in a puddle of perfluoropolyalkylether oil on a microscope slide.
In our hands, the yield of this reaction could be enhanced to up to 40%, by prolonging the time given for crystallization. In that case—*i.e.* the one resulting in a 40% yield,—crystallization was carried out over the course of 1.5 months.
ildc has very low solubility properties. In order to solubilize a sample in toluene, benzene or DCM for analysis, intense stirring or sonication are necessary over a prolonged period of time.

^1H NMR (300 MHz, C_6D_6): δ [ppm] 11.74 (s, 4H), 10.95 (bs, 2H), 10.47 (bt, 2H), 5.72 (s, 8H), 4.21 (bd, 4H), 4.05 (s, 12H), 3.25 (bs, 8H), 2.20 (s, 12H), –0.36 (s, 24H), –2.56-(–2.84) (m, 12H).
^1H NMR (300 MHz, CD_2Cl_2): δ [ppm] 12.35 (bd, 4H), 10.74 (t, *J*=7.5 Hz, 2H), 6.80 (s, 2H), 5.68 (s, 8H), 4.13 (d, *J*=7.8 Hz, 4H), 4.08 (s, 12H), 2.34 (s, 4H), 2.23 (s, 12H), –0.64 (s, 24H), –2.14-(–2.49) (m, ~12H).
UV-vis ($1\cdot10^{-4}$ M in toluene): λ [nm] (ε [10^3 M^{-1} cm^{-1}]) 342 (p, 30.7), 467 (p, 38.4), 631 (sh, 15.5).
Elemental analysis: ($C_{96}H_{100}Fe_8S_{12}\cdot C_7H_8\cdot0.5C_5H_{12}$) expected: C 57.25%, H 5.19%; found: C 57.11%, H 5.27%.

$[Fe_8S_8(DmpS)_6]$ (***Ibdc***)

$K_4[Fe_8S_8(DmpS)_6]$ (252.9 mg, 0.078 mg, 1.0 equiv.) was dissolved in toluene (20 mL). While stirring, $[FeCp_2]PF_6$ (105.9 mg, 0.320 mmol, 4.1 equiv.) was added as a solid. After few minutes, the color had turned from brownish black to purplish black. Stirring was continued for 4 h, after which the solvent was partially removed (to 10 mL) *in vacuo*, and the mixture filtered and layered with pentane

(35 mL) for crystallization. After 6 days, aggregates of medium-sized black crystals had formed. These crystals were decanted and washed with pentane until the washings turned clear (10 mL). The solid was dried *in vacuo*, yielding 145.4 mg. Benzene (5 mL in one portion) was added to this solid, and gently swirled, which caused the ***lbdc*** to dissolve, coloring the liquid dark purplish black. The liquid was decanted off and filtered before being frozen (–30°C). This frozen solution was lyophilized, yielding a fluffy dark solid consisting of ***lbdc*** with <4% ***ildc*** impurity based on a ^{1}H NMR analysis (37.6 mg, 0.0135 mmol, 17%).
Alternatively, instead of benzene, toluene can be used to extract ***lbdc*** from the crude recrystallized product, and precipitated from the solution by layering with a large amount of HMDSO overnight.
Single-crystals suitable for X-ray diffraction analysis were grown by recrystallization from a concentrated toluene solution by vapor diffusion with pentane.

^{1}H NMR (300 MHz, C_6D_6): δ [ppm] 11.99 (bd, 2H), 8.23 (bs, 2H), 6.79 (s, 8H), 6.62 (bd, 4H), 5.80 (s, 4H), 2.89 (s, 24H), 2.81 (bs, 12H), 2.61 (s, 6H), 2.10 (s, 12H), –0.41 (bs, 1H).
^{1}H NMR (300 MHz, CD_2Cl_2): δ [ppm] 12.29 (bd, 2H), 8.63 (bs, 2H), 6.68 (s, 8H), 6.47 (bd, 4H), 5.55 (s, 4H), 2.56 (bs, 36 H), 2.44 (s, 6H), 2.02 (s, 12H), –0.64 (s, 1H).
UV-vis ($1\cdot10^{-4}$ M in toluene): λ [nm] (ε [10^3 M^{-1} cm^{-1}]) 344 (sh, 35.9), 471 (p, 46.9).
Elemental analysis: ($C_{144}H_{150}Fe_8S_{14}\cdot0.5C_6H_{18}Si_2O$) expected: C 62.04%, H 5.53%; found: C 61.94%, H 5.67%.

Conversion of $[Fe_4S_4(DmpS)_2(Im^*)_2]$ to $K_2[Fe_4S_4(DmpS)_4]$

This experiment was conducted on an NMR scale. To this end, inside an Ar-filled glovebox, a J. Young NMR tube was charged with 12.1 mg $[Fe_4S_4(DmpS)_2(Im^*)_2]$ (0.009 mmol, 1.0 equiv.), which was dissolved in CD_2Cl_2, and a spectrum was recorded. Subsequently, the tube was re-introduced into the glovebox, and a THF-*d*8 solution of DmpSK (3.6 mg, 0.009 mmol, 1.1 equiv.) was added. A spectrum was again recorded, and another 1.1 equiv. of DmpSK were added as a solid (3.6 mg, 0.009 mmol). All spectra, as well as those of select reference compounds relevant for this reaction (Im*, $K_2[Fe_4S_4(DmpS)_4]$) are compiled in Supplementary Fig. 57, showing the quantitative and clean conversion of the 2:2 site-differentiated cubane, $[Fe_4S_4(DmpS)_2(Im^*)_2]$, back to its canonical congener, $K_2[Fe_4S_4(DmpS)_4]$.
The same becomes evident from the corresponding UV-vis titration experiment, conducted under the analogous conditions, which is shown in Supplementary Fig. 20, and exhibits the clear trend that is anticipated based on the comparison of the spectra recorded on the isolated compounds (Supplementary Fig. 15A).

Conversion of ***ildc*** to $K_2[Fe_4S_4(DmpS)_4]$

This experiment was conducted on an NMR scale. To this end, inside an Ar-filled glovebox, a 5 mL scintillation vial was charged with 11.4 mg $[Fe_8S_8(DmpS)_4]$ (0.005 mmol, 1.0 equiv.), 9.2 mg DmpSK (0.023 mmol, 4.4 equiv.) and approximately 800 µL THF-*d*8. The resulting dark brown suspension was stirred over the course of 2 days. During this time, the solids gradually dissolved, and afterwards, the mixture was filtered into a J. Young NMR tube.
A ^{1}H NMR spectrum was recorded (Supplementary Fig. 58), which conformed the successful formation of $K_2[Fe_4S_4(DmpS)_4]$, when the latter was compared to a spectrum of a neat sample.

Initial preparation/identification of ***ildc*** and synthetic attempts toward its direct synthesis from $[Fe_4S_4(DmpS)_4]$ and $[Fe_4S_4(DmpS)_2(Im^*)_2]$

ildc was originally identified upon attempted crystallization of $[Fe_4S_4(DmpS)_4]$ from a concentrated dichloromethane solution. We outline a typical preparation:

$K_2[Fe_4S_4(DmpS)_4]$ (300.5 mg, 0.140 mmol, 1.0 equiv.) and $[FeCp_2]PF_6$ (105.6 mg, 0.318 mmol, 2.2 equiv.) were combined in toluene (15 mL) and the resulting mixture was stirred for 3 h. Subsequently, KPF_6 was centrifuged off and the supernatant was taken to dryness, before being slurried with pentane and dried again to remove residual toluene. Afterward, the black solid was dissolved in DCM (10 mL) and layered with an equivalent amount of pentane (10 mL). The vial was placed in an undisturbed location of the glovebox and left to stand for 10 days. After this time, a crop of single crystals had formed, identifying as $[Fe_8S_8(DmpS)_4]\cdot CH_2Cl_2$ (34.2 mg, 0.016 mmol, 22%), which were decanted, washed with pentane and dried.

^{1}H NMR (300 MHz, C_6D_6): δ [ppm] 11.74 (s, 4H), 10.95 (bs, 2H), 10.47 (bt, 2H), 5.72 (s, 8H), 4.21 (d, 4H), 4.05 (s, 12H), 3.25 (bs, 8H), 2.20 (s, 12H), –0.36 (s, 24H), –2.56-(–2.84) (m, 12H).
UV-vis ($1\cdot10^{-4}$ M in toluene): λ [nm] (ε [10^3 M^{-1} cm^{-1}]) 342 (p, 26.1), 467 (p, 35.5), 631 (sh, 15.4).
Elemental analysis: ($C_{96}H_{100}Fe_8S_{12}\cdot C_2H_2Cl_2$) expected: C 53.94%, H 4.71%; found: C 53.82%, H 5.09%.

Upon further standing of the mother liquor for approximately two weeks' time, another crop was collected (56.0 mg), which contained three kinds of single crystals: (*i*) $[Fe_8S_8(DmpS)_4]\cdot CH_2Cl_2$, (*ii*) $[Fe_8S_7(DmpS)_3Cl_2]$ (Supplementary Fig. 90), as well as, (*iii*) $[Fe(DmpS)Cl]_2$ (Supplementary Fig. 91).

Supplementary Note 1. The formation of ***ildc*** from the all-ferric cubane in DCM is peculiar, and we suspect that it is based on the existence of a non-negligible equilibrium between [Fe_4S_4(DmpS)$_4$] and 0.5 equiv. of ***lbdc*** and the DmpS$^{\bullet}$ radical, or disulfide, respectively. Given the formation of chloride-substituted FeS complexes from the same reaction mixture (*vide supra*), it seems likely that this is promoted by a weak "solvating" interaction between DCM chlorine atoms and the ferric ions, leading to dynamic reductive site-differentiation equilibria, promoted by high Fe^{III}-S(thiolate) bond covalency (Supplementary Fig. 2).[32]

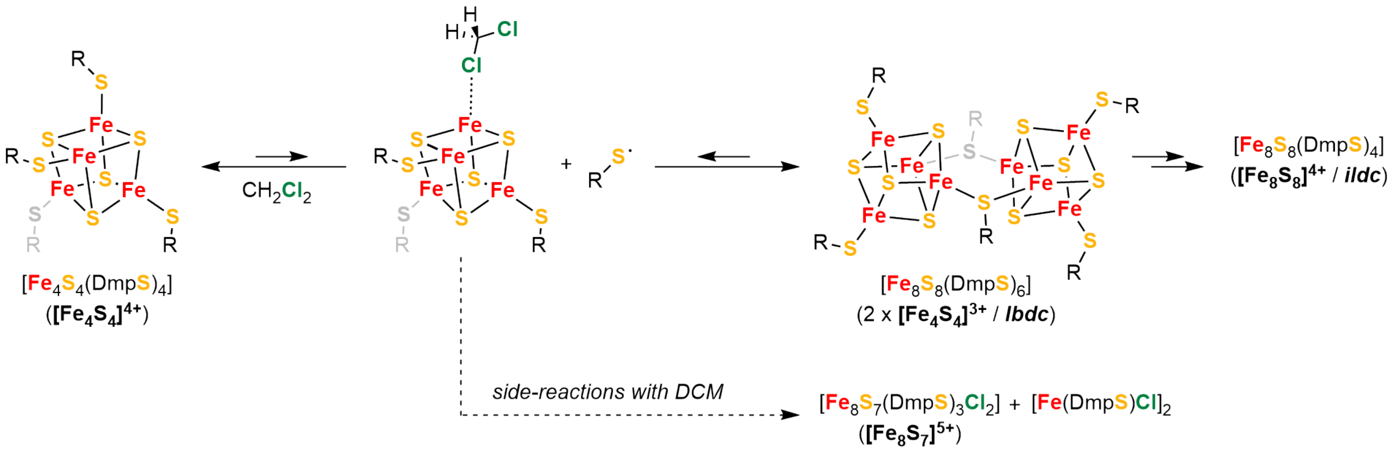

Supplementary Figure 2. Proposed mechanism of the formation of ***ildc*** from the all-ferric cubane in dichloromethane.

Based on this initial success, we had considered two other Fe_4S_4 complexes as promising synthetic precursors to ***ildc***: (*i*) [Fe_4S_4(DmpS)$_4$], from which two-electron reduction and twofold loss of ligand (or direct reductive elimination of disulfide) should lead to ***ildc***, and (*ii*) [Fe_4S_4(DmpS)$_2$(Im*)$_2$], from which removal of Im* ligands without redox change could result in formation of ***ildc***.
We elaborate some specifics of our strategies and attempts in more detail:

(*i*) Because ***ildc*** forms (among other products) over time from [Fe_4S_4(DmpS)$_4$] in DCM (*vide supra*), we hoped that either irradiation of a toluene solution of the all-ferric cubane with light or heating it would promote the reductive elimination of (DmpS)$_2$, concomitant with the formation of 0.5 equiv. of ***ildc***. However, despite multiple attempts, our synthetic efforts to do so failed. While Supplementary Fig. 19 shows that [Fe_4S_4(DmpS)$_4$] is indeed sensitive toward UV-light, the photoreaction monitored by UV-vis electronic absorption spectroscopy does not evidence formation of ***ildc***. Also, our attempts to characterize the products of this reaction, if carried out on a preparative scale, failed altogether.
In a next attempt, we considered the fact that a small disulfide, (PhS)$_2$, might facilitate the reductive elimination of DmpS-ligands from the cubane, because the formation of DmpS-SPh should be less hindered by sterics than that of (DmpS)$_2$. However, [Fe_4S_4(DmpS)$_4$] does not appear to react with (PhS)$_2$ (Supplementary Figs. 4A and 16A). Last, we considered that the reduction of [Fe_4S_4(DmpS)$_4$] with Mg(THF)$_3$[$C_{14}H_8$] might yield ***ildc***; This reagent provides the (two-electron) reducing equivalents to produce the redox state of ***ildc***, while the Lewis acidic Mg^{2+} ion might bind the thiolates. Unfortunately, these attempts also remained unsuccessful (Supplementary Figs. 4A and 16A).

(*ii*) Another potential synthetic route is based on triggering the removal of two Im* ligands from [Fe_4S_4(DmpS)$_2$(Im*)$_2$] by two equiv. of B(C_6F_5)$_3$. To this end, in a typical attempt, a solution B(C_6F_5)$_3$ (123.7 mg, 0.241 mmol, 2.1 equiv.) in toluene (3 mL) was added to a solution of [Fe_4S_4(DmpS)$_2$(Im*)$_2$] (150.2 mg, 0.110 mmol, 1.0 equiv.) in toluene (7 mL). The resulting mixture was stirred overnight, with no obvious color-change occurring. On the next day, the solution was concentrated to about half its volume, filtered and set for crystallization by vapor diffusion with

pentane. Monitoring this reaction *via* UV-vis electronic absorption spectroscopy, however, did not indicate formation of ***ildc*** (Supplementary Figs. 3A and 16B).

While we were able to confirm that Im* ligands are successfully removed from the cubane *via* crystallographic characterization of Im*·B(C_6F_5)$_3$ from the reaction mixture (Supplementary Fig. 87), we were not able to detect the formation of ***ildc***. Among the many possible reaction products, we did however, identify and structurally characterize [$Fe_{24}S_{24}$(DmpS)$_{10}$] (Supplementary Figs. 3 and 88), single-crystals of which formed after 4 weeks of undisturbed standing. We recognize this as the product of chaotic FeS cluster aggregation. While this synthesis is far from quantitative, we believe that its molecular structure is noteworthy, because it is—to the best of our knowledge—the largest reported molecular FeS cluster complex, spanning 2 nm from side-to side on its longest dimension (Supplementary Figs. 3 and 88).

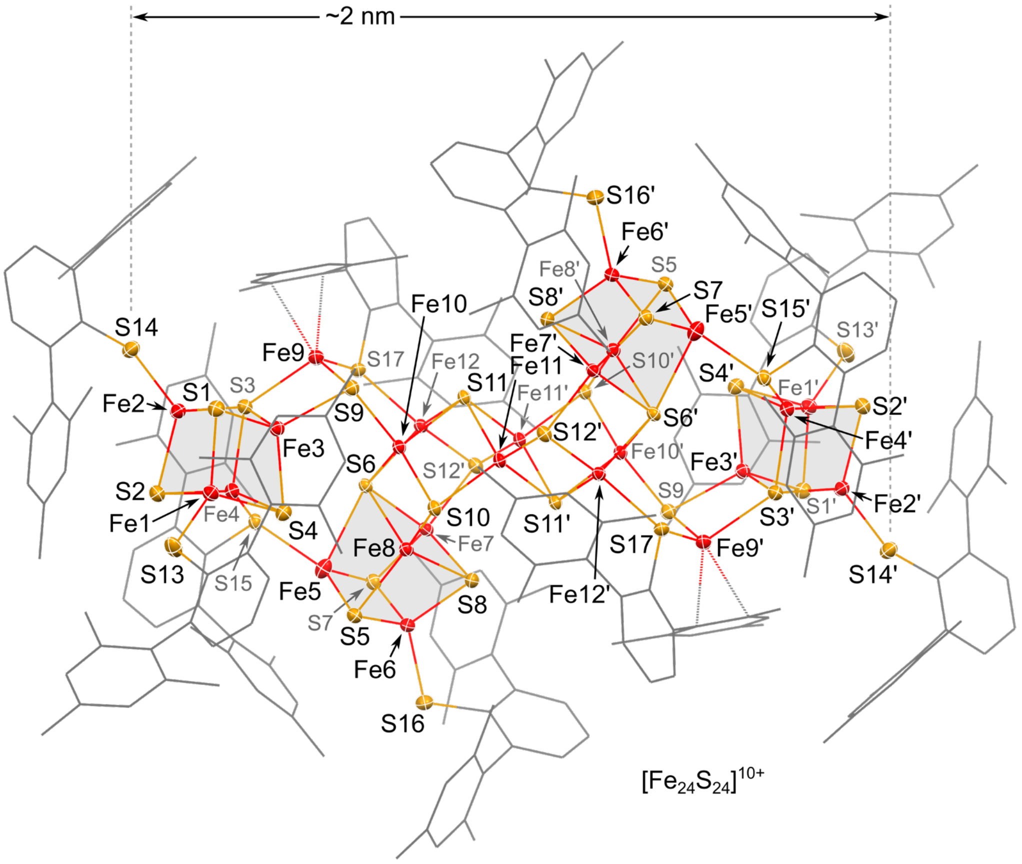

Supplementary Figure 3. Solid-state molecular structure of [$Fe_{24}S_{24}$(DmpS)$_{10}$] in crystals of [$Fe_{24}S_{24}$(DmpS)$_{10}$]·8(C_7H_8). Displacement ellipsoids are shown at the 50% probability level, only for the Fe and S atoms. For clarity, hydrogen atoms as well as co-crystallized solvent molecules have been omitted. Four Fe_4S_4-units within the structure have been highlighted by shaded *grey* areas.

Supplementary Note 2. Because we observed that higher-nuclearity clusters (8Fe–8S) could be constructed by triggering the loss of Im* ligands from reduced 3:1 site-differentiated Fe_4S_4 complexes (*i.e.* forming ***ebdc*** by reduction of $K[Fe_4S_4(DmpS)_3(Im^*)]$; Figs. 3B,C), we investigated the analogous reactivity of the 2:2 site-differentiated Fe_4S_4 derivative, $[Fe_4S_4(DmpS)_2(Im^*)_2]$. In fact, we were pleased to find that the twofold reduction of $[Fe_4S_4(DmpS)_2(Im^*)_2]$ accordingly leads to the twofold loss of Im*, and the formation of an edge-bridged triple-cubane (***ebtc***) $[Fe_{12}S_{12}]^0$ assembly, $K_6[Fe_{12}S_{12}(DmpS)_6]$ (Supplementary Figs. 4A,B and 89). Importantly, we should point-out that this result underscores the notion that the redox-neutral loss of weakly covalent ligands from reduced Fe_4S_4 complexes, as proposed in Fig. 3B of the main text, is in fact a good general strategy for the construction of higher-nuclearity FeS clusters. For ***ebtc***, a typical preparation is described below.

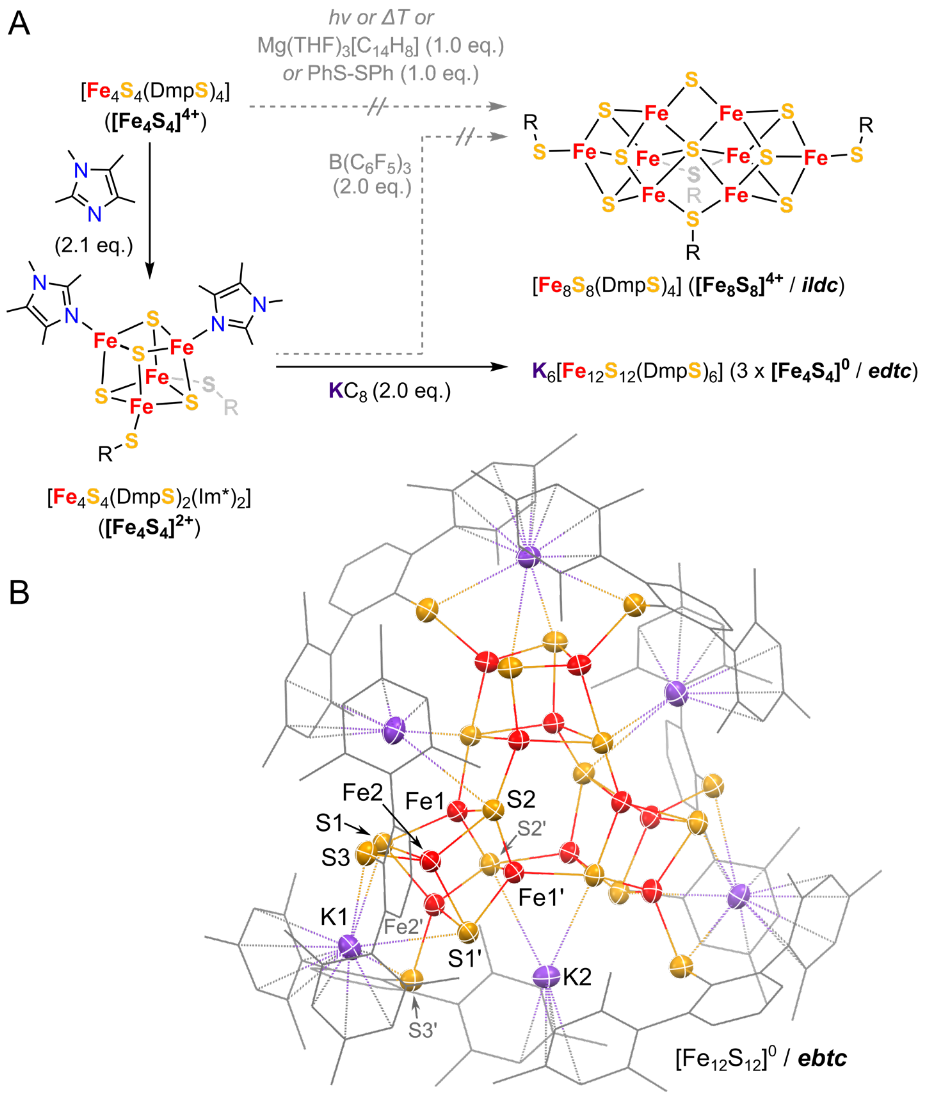

Supplementary Figure 4. (A) Synthetic attempts from $[Fe_4S_4(DmpS)_4]$ and $[Fe_4S_4(DmpS)_2(Im^*)_2]$ toward ***ildc***, as well as conditions for the synthesis of $K_6[Fe_{12}S_{12}(DmpS)_6]$ (***ebtc***) from $[Fe_4S_4(DmpS)_2(Im^*)_2]$. (B) Solid-state molecular structure of $K_6[Fe_{12}S_{12}(DmpS)_6]$ in crystals of $K_6[Fe_{12}S_{12}(DmpS)_6]\cdot 2(C_5H_{12})\cdot 3(C_7H_8)$. Displacement ellipsoids are shown at the 30% probability level for the Fe, S and K atoms. Hydrogen atoms and co-crystallized solvent molecules are omitted for clarity.

$K_6[Fe_{12}S_{12}(DmpS)_6]$ (***ebtc***)

$[Fe_4S_4(DmpS)_2(Im^*)_2]$ (50.1 mg, 0.037 mmol, 1.0 equiv.) were dissolved in toluene (5 mL) and KC_8 (10.9 mg, 0.081 mmol, 2.1 equiv.) was added to this solution as a slurry in toluene. The resulting mixture was stirred for 1 h. Afterwards, graphite was filtered off, and the reaction mixture was layered with a generous amount of pentane, leading to the formation of a black precipitate after 3 days. The precipitate was decanted, washed with pentane and dried, yielding 19.8 mg (0.006 mmol, 46%) of product.
Among a large amount of black precipitate, small single crystals suitable for X-ray diffraction analysis grew by vapor diffusion of pentane into a dilute toluene solution of the product at room temperature. The ^{1}H NMR spectrum recorded in THF-*d*8 shows signals of the solvent, as well as those of co-crystallized solvent, and is otherwise featureless (Supplementary Fig. 53). This indicates that the compound is NMR silent.

UV-vis ($1{\cdot}10^{-4}$ M in THF): λ [nm] (ε [10^3 M^{-1} cm^{-1}]) 407 (p, 29.8), 562 (sh, 22.2).
Elemental analysis: ($C_{154}H_{174}Fe_{12}K_6S_{18}{\cdot}0.5C_7H_8$) expected: C 53.24%, H 5.05%; found: C 53.31%, H 5.18%.

Other Non-Preparative Procedures and Transformations of the Oxidized Fe_4S_4 Complexes

Supplementary Note 3. None of the procedures described below were carried out in a preparative fashion. The reaction products were identified based on single-crystal X-ray diffraction of species that crystallized from the reaction's mother liquor under various conditions. This constitutes the main technique for the identification of reaction products in cluster chemistry in general. By no means do we claim that they are the exclusive products of the reaction. If a large enough quantity of the compound could be isolated, we provide its UV-vis absorption spectrum as a basic identifier. We emphasize that these transformations capture the complexity and diversity of this chemistry, and should be of use to other researchers in the field of synthetic FeS cluster chemistry to identify and anticipate similar reactivities in their studies.

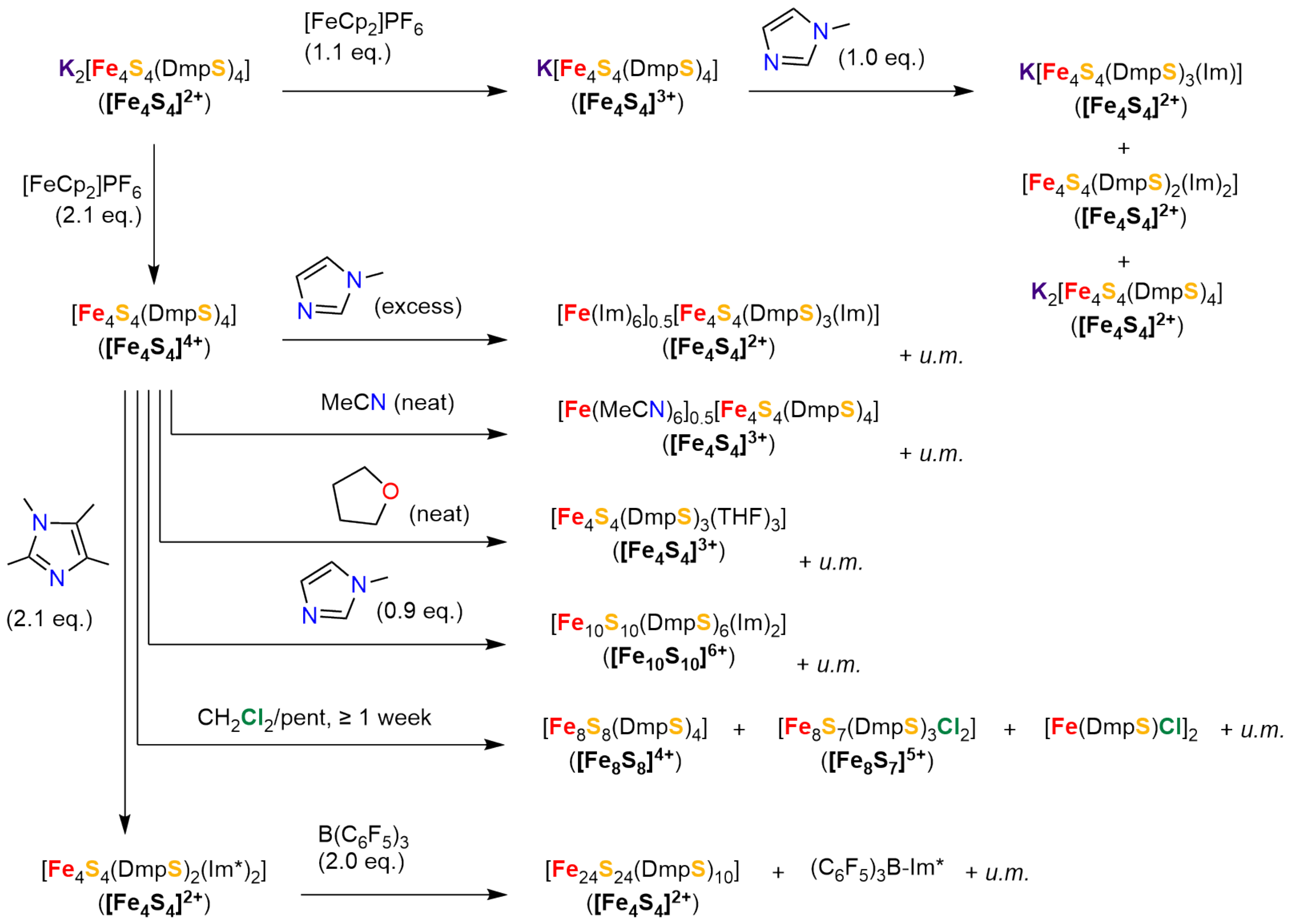

Supplementary Figure 5. Summary of selected transformations of the $[Fe_4S_4]^{3+}$ and $[Fe_4S_4]^{3+}$ cubanes with varying weak N-donor ligands and in various solvents.

<u>Reaction of $[Fe_4S_4]^{3+}$ with 1-Me-imidazole</u>

$K[Fe_4S_4(DmpS)_4]$ (59.9 mg, 0.032 mmol, 1.00 equiv.) was dissolved in toluene (5 mL) and treated with 35 µL of a 1 M solution of Im (Im=1-methylimidazole; 0.035 mmol, 1.1 equiv.) in toluene. The mixture turned from dark purplish brown to dark yellowish brown immediately. Stirring was continued for 2 h, after which the mixture was concentrated to approximately 2 mL, filtered and set for crystallization by vapor diffusion with pentane.
After 3 weeks, two types of small black single-crystals were obtained, which were suitable for X-ray diffraction analysis. Based on this, we were able to assign the products of the reaction as, (*i*) $K_2[Fe_4S_4(DmpS)_4]$, the structure of which we reported previously,[1] and, (*ii*), $[Fe_4S_4(DmpS)_2(Im)_2]\cdot K[Fe_4S_4(DmpS)_3(Im)]$ (Supplementary Fig. 81). The characterization of all three of these species within the same reaction mixture indicates significant ligand scrambling between them.

Reaction of $[Fe_4S_4]^{4+}$ with excess 1-Me-imidazole

$[Fe_4S_4(DmpS)_4]$ (100 mg, 0.057 mmol, 1.00 equiv.) was dissolved in toluene (10 mL) and Im (50 mg, 0.609 mmol, 10.6 equiv.) was added (Im=1-methylimidazole). The mixture turned dark yellowish immediately and was subsequently stirred for 2 h. The solution was concentrated *in vacuo* to half its original volume, filtered and layered with pentane. After two days, the product, obtained as large black crystals, was washed with pentane and dried (26 mg, 27%). Single crystals suitable for X-ray diffraction analysis were obtained by vapor diffusion of a toluene solution with pentane, identifying the product being $[Fe_4S_4(DmpS)_3(Im)]\cdot 0.5[Fe(Im)_6]$.

UV-vis ($1\cdot 10^{-4}$ M in toluene): λ [nm] (ε [10^3 M^{-1} cm^{-1}]) 346 (sh, 20.7), 408 (p, 18.4).

Reaction of the $[Fe_4S_4]^{4+}$ cubane with sub-stoichiometric 1-Me-imidazole

A 1 M solution of Im (47 µL, 0.047 mmol, 0.90 equiv.) was added to a stirred solution of $[Fe_4S_4(DmpS)_4]$ (99.7 mg, 0.053 mmol, 1.00 equiv.) in toluene (10 mL). The mixture immediately turned from dark blueish black to dark purple, and stirring was continued for 1 h. Afterward, the mixture was concentrated to approximately 5 mL, and layered with pentane. After 2 weeks, a small number of single crystals of $[Fe_{10}S_{10}(DmpS)_6(Im)_2]$ were decanted, washed with pentane and dried, yielding 13.0 mg of product. Due to the odd stoichiometry of this reaction, we forego the analysis of a yield.
The crystals were of X-ray diffraction quality, so some of them were separated from the bulk prior to drying and placed in a puddle of perfluoropolyalkylether oil on a microscope slide.

UV-vis ($1\cdot 10^{-4}$ M in toluene): λ [nm] (ε [10^3 M^{-1} cm^{-1}]) 357 (p, 43.3), 498 (p, 54.5).

Supplementary Note 4. Even though the UV-vis titration experiments of $[Fe_4S_4(DmpS)_4]$ and $K[Fe_4S_4(DmpS)_4]$ with Im and Im*, respectively (Supplementary Fig. 8) appear very similar, these experiments show that although similar intermediates may form during the reactions of the imidazoles with the cubanes, the stability of these differ depending on the imidazole derivative (and more broadly, depending on the N-donor ligand's properties; Supplementary Fig. 4).

Reaction of the $[Fe_4S_4]^{4+}$ cubane with excess acetonitrile

$[Fe_4S_4(DmpS)_4]$ (28.4 mg, 0.015 mmol, 1.00 equiv.) was dissolved in MeCN (2 mL), layered with toluene (3 mL) and left to stand. After two weeks, a few large single-crystals grew, which were suitable for X-ray diffraction analysis, revealing the identity of the main product being $[Fe_4S_4(DmpS)_4]\cdot 0.5[Fe(MeCN)_6]$ (Supplementary Fig. 84). The yield was not measured.

Reaction of the $[Fe_4S_4]^{4+}$ cubane and ***Ibdc*** with excess THF

$[Fe_4S_4(DmpS)_4]$ (250.1 mg, 0.131 mmol, 1.00 equiv.) was dissolved in THF (10 mL) and stirred for 4 h. The solution was concentrated, filtered and layered with HMDSO. After two days, the crystalline, black product was washed with HMDSO and dried (33 mg, 14%). The crystalline product was analyzed by single-crystal XRD and UV-vis electronic absorption spectroscopy to approve its identity being $[Fe_4S_4(DmpS)_3(THF)_3]$ (Supplementary Fig. 85).[26] Notably, crystals of the disulfide DmpSSDmp (Supplementary Fig. 95) and, more peculiarly, of the trisulfide, DmpSSSDmp, formed in the mother liquor some days later (Supplementary Fig. 86), hinting toward an ill-defined reaction mechanism and potential Fe_4S_4 core disruption.

Similarly, upon dissolution of 20.3 mg (0.007 mmol, 1.00 equiv.) of ***Ibdc*** in THF, followed by layering with copious amounts of HMDSO, single-crystals of [$Fe_4S_4(DmpS)_3(THF)_3$] (5.7 mg, 0.004 mmol, 27%) could be obtained in low yield.

UV-vis ($1{\cdot}10^{-4}$ M in toluene): λ [nm] (ε [10^3 M^{-1} cm^{-1}]) 345 (sh, 15.6), 408 (p, 20.2).

UV-vis Electronic Absorption Spectroscopy

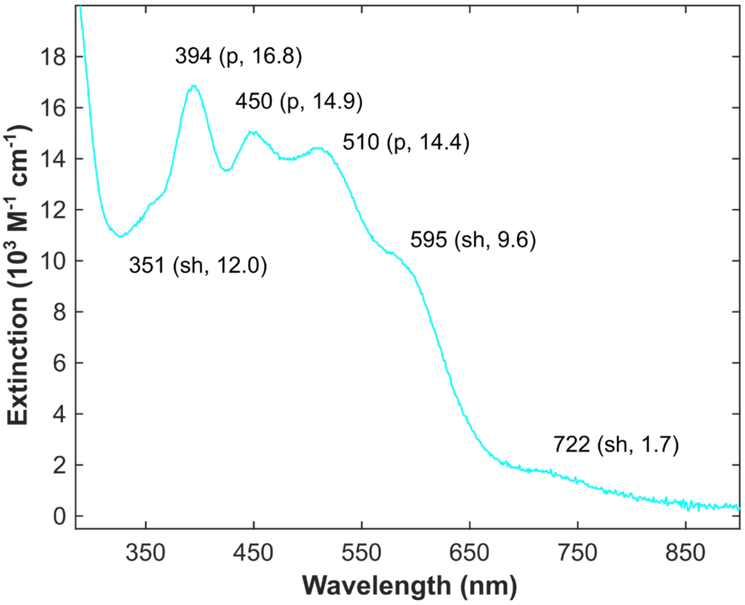

Supplementary Figure 6. UV-vis electronic absorption spectrum of a $1 \cdot 10^{-4}$ M toluene solution of $[Fe_2S_2(DmpS)_2(py)_2]$ measured at room temperature.

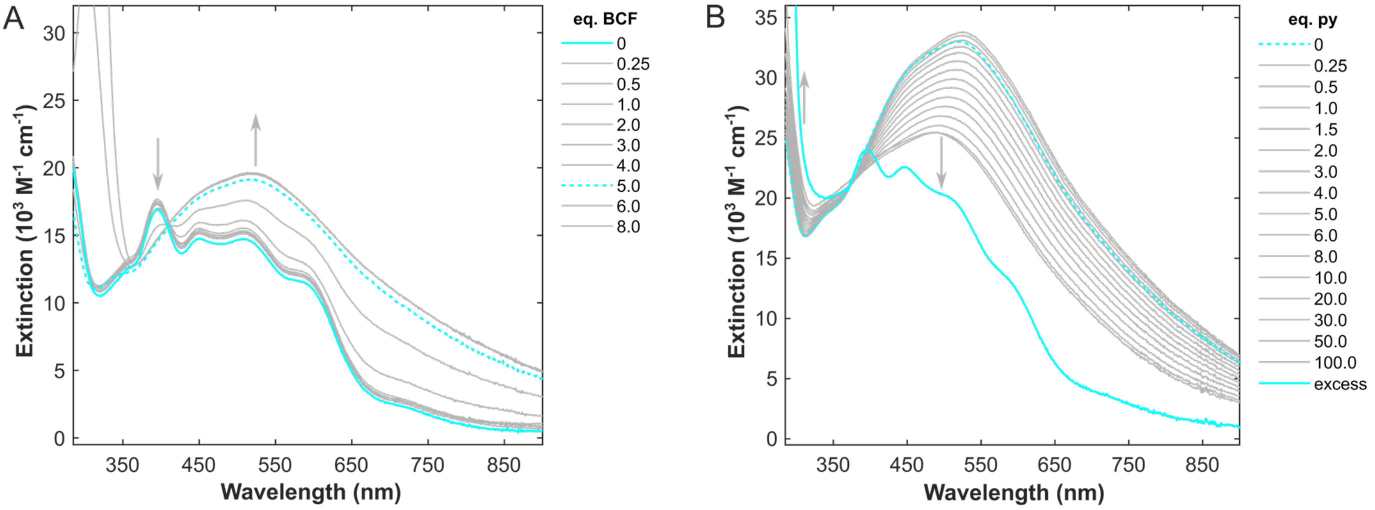

Supplementary Figure 7. (A) UV-vis electronic absorption spectrum of a $1 \cdot 10^{-4}$ M toluene solution of $[Fe_2S_2(DmpS)_2(py)_2]$ measured at room temperature and the evolution of the spectrum upon titration with stoichiometric amounts of $B(C_6F_5)_3$ (*grey lines* and *arrows*). After addition of 4-5 equiv. of the Lewis acid, the expected spectrum of $[Fe_4S_4(DmpS)_4]$ appeared. (B) Reverse experiment to (A); Titration of a $1 \cdot 10^{-4}$ M toluene solution of $[Fe_4S_4(DmpS)_4]$ with stoichiometric amounts of pyridine (*grey lines* and *arrows*).

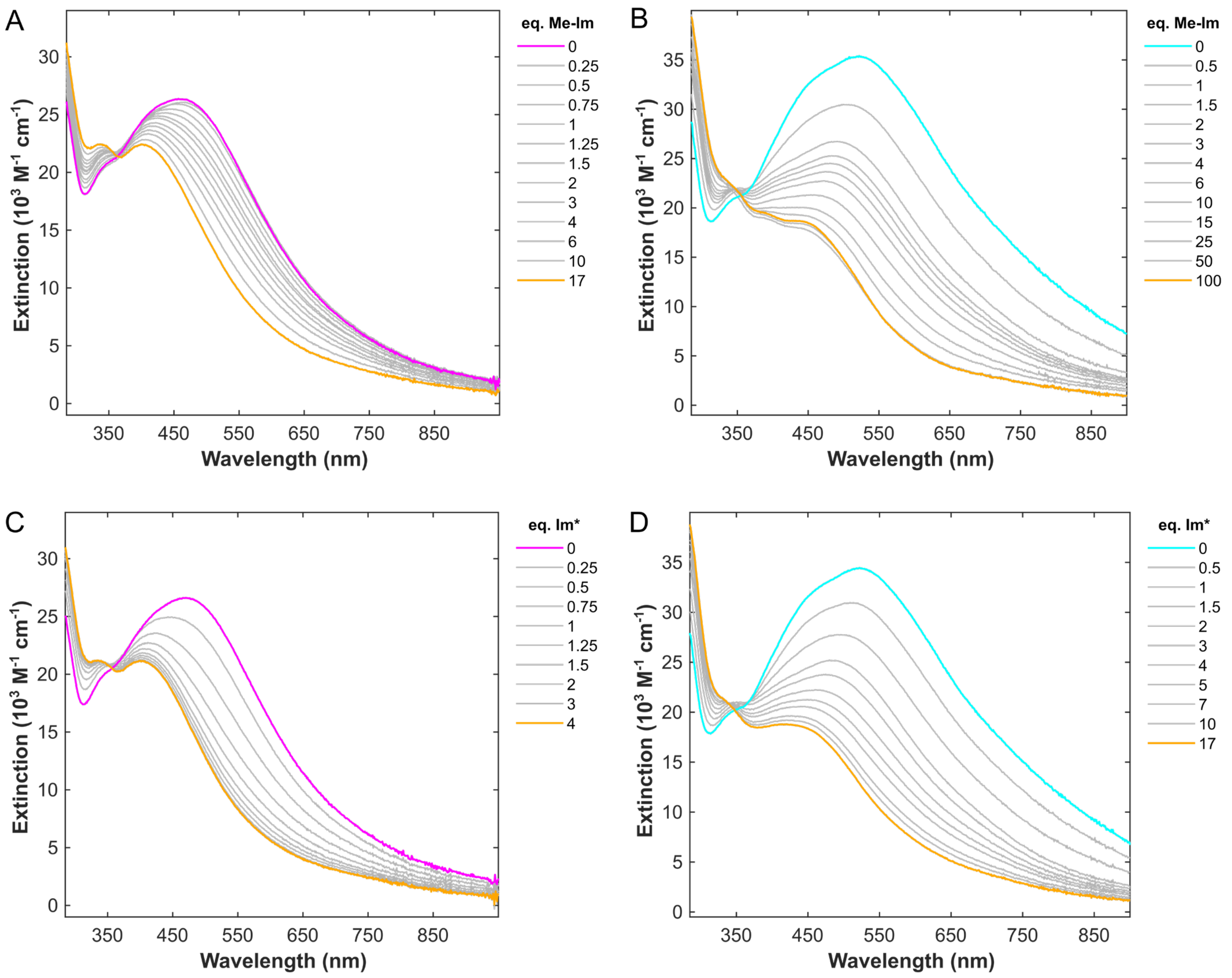

Supplementary Figure 8. (A,B) UV-vis electronic absorption spectra of 1·10^–4^ M toluene solutions of K[Fe_4S_4(DmpS)$_4$] (A) and [Fe_4S_4(DmpS)$_4$] (B), respectively, and their evolution upon titration with stoichiometric amounts of 1-Me-imidazole (Im). (C,D) UV-vis electronic absorption spectra of $1\cdot10^{-4}$ M toluene solutions of K[Fe_4S_4(DmpS)$_4$] (C) and [Fe_4S_4(DmpS)$_4$] (D), respectively, and their evolution upon titration with stoichiometric amounts of 1,2,4,5-tetramethylimidazole (Im*).

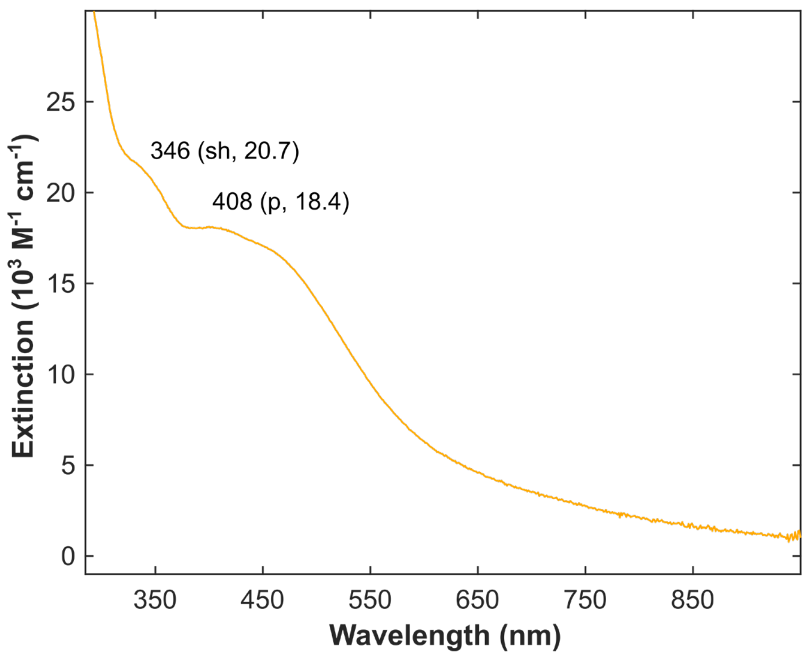

Supplementary Figure 9. UV-vis electronic absorption spectrum of a $1{\cdot}10^{-4}$ M toluene solution of $[Fe_4S_4(DmpS)_3(Im)]{\cdot}0.5[Fe(Im)_6]$ recorded at room temperature.

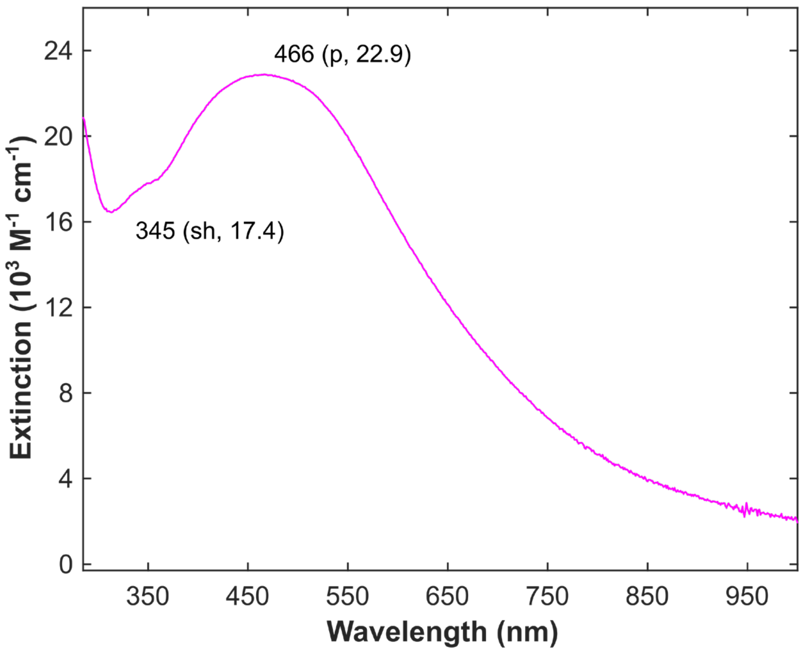

Supplementary Figure 10. UV-vis electronic absorption spectrum of a $1{\cdot}10^{-4}$ M toluene solution of $[Fe_4S_4(DmpS)_3(THF)_3]$ recorded at room temperature.

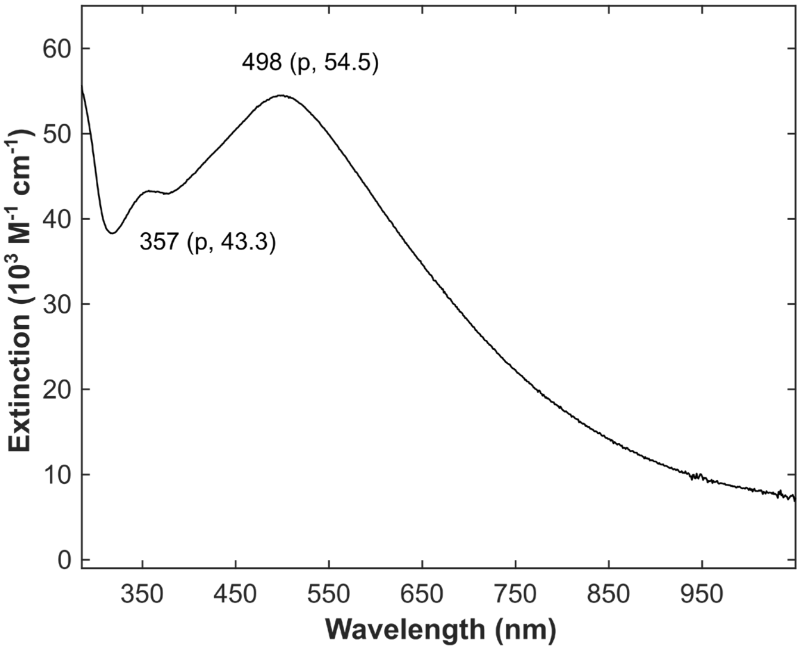

Supplementary Figure 11. UV-vis electronic absorption spectrum of a 1·10^{-4} M toluene solution of [$Fe_{10}S_{10}$(DmpS)$_6$(Im)$_2$] recorded at room temperature.

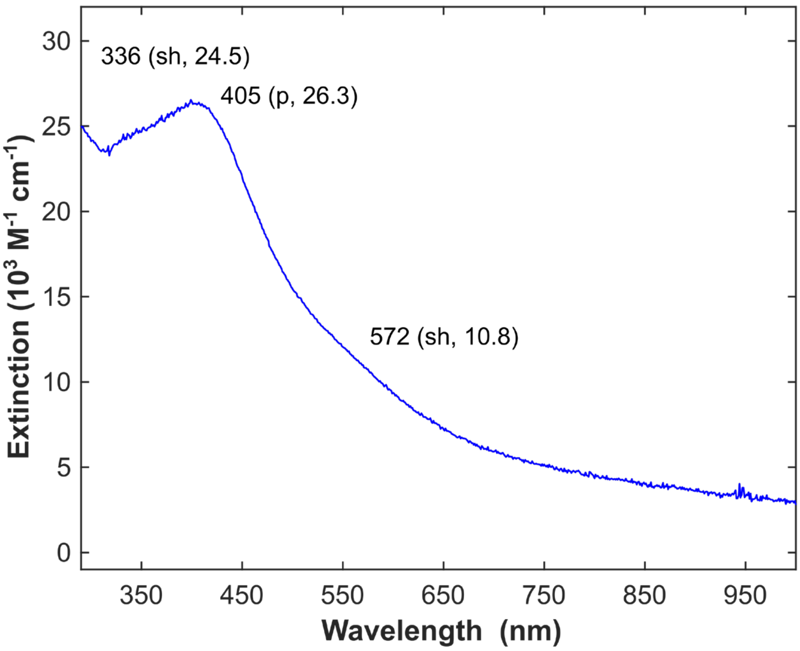

Supplementary Figure 12. UV-vis electronic absorption spectrum of a 1·10^{-4} M THF solution of K_4[Fe_8S_8(DmpS)$_6$] recorded at room temperature.

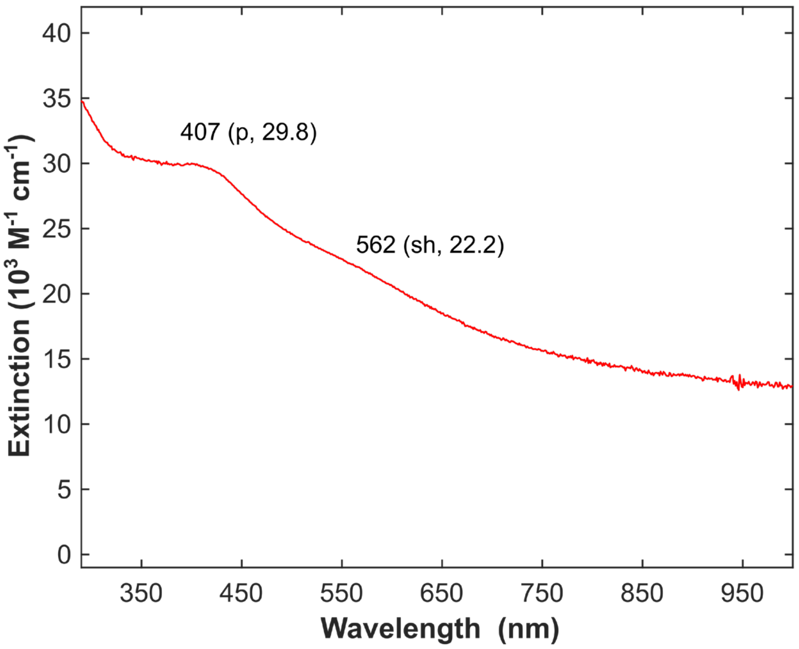

Supplementary Figure 13. UV-vis electronic absorption spectrum of a $1{\cdot}10^{-4}$ M THF solution of $K_6[Fe_{12}S_{12}(DmpS)_6]$ recorded at room temperature.

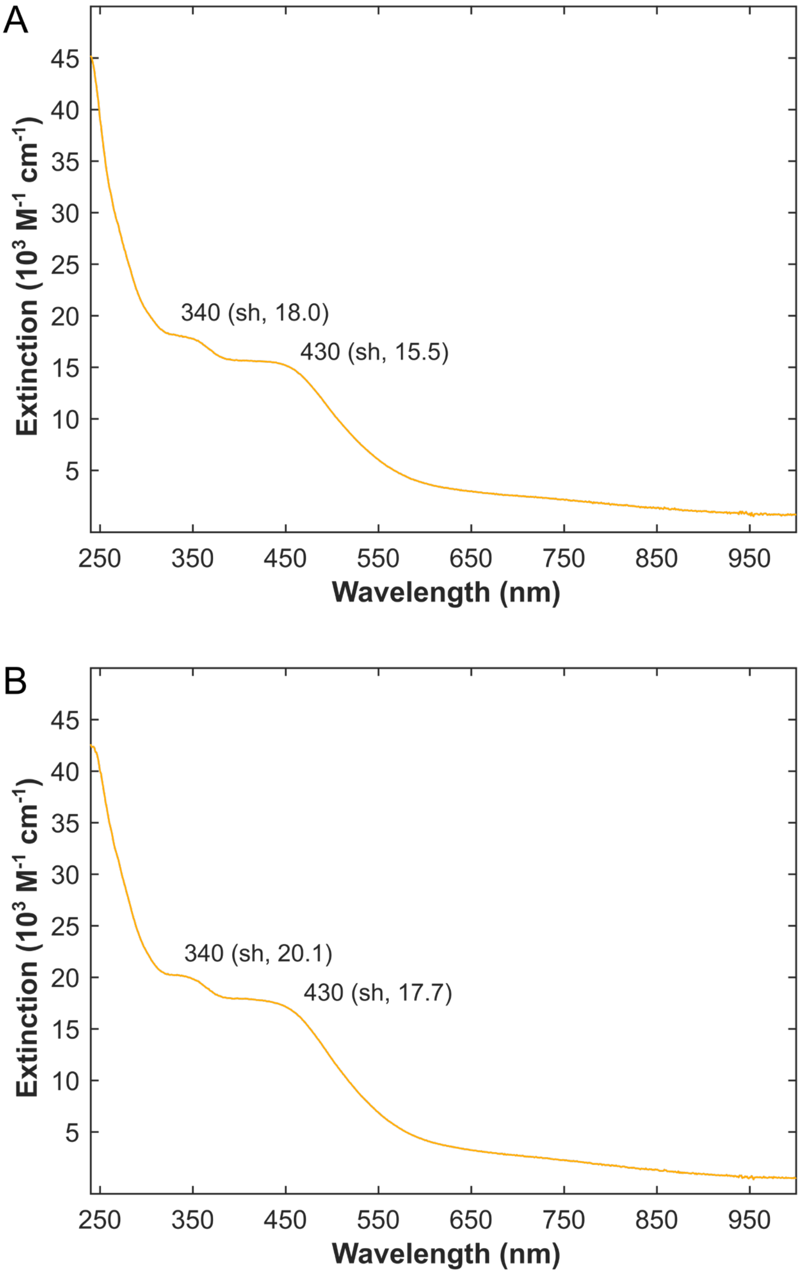

Supplementary Figure 14. UV-vis electronic absorption spectra of $^{[2.2.2]}K[Fe_4S_4(DmpS)_3(Im^*)]$ (A) and $^{[18\text{-}C\text{-}6]}K[Fe_4S_4(DmpS)_3(Im^*)]$ (B) recorded in $1 \cdot 10^{-4}$ M DCM solution at room temperature.

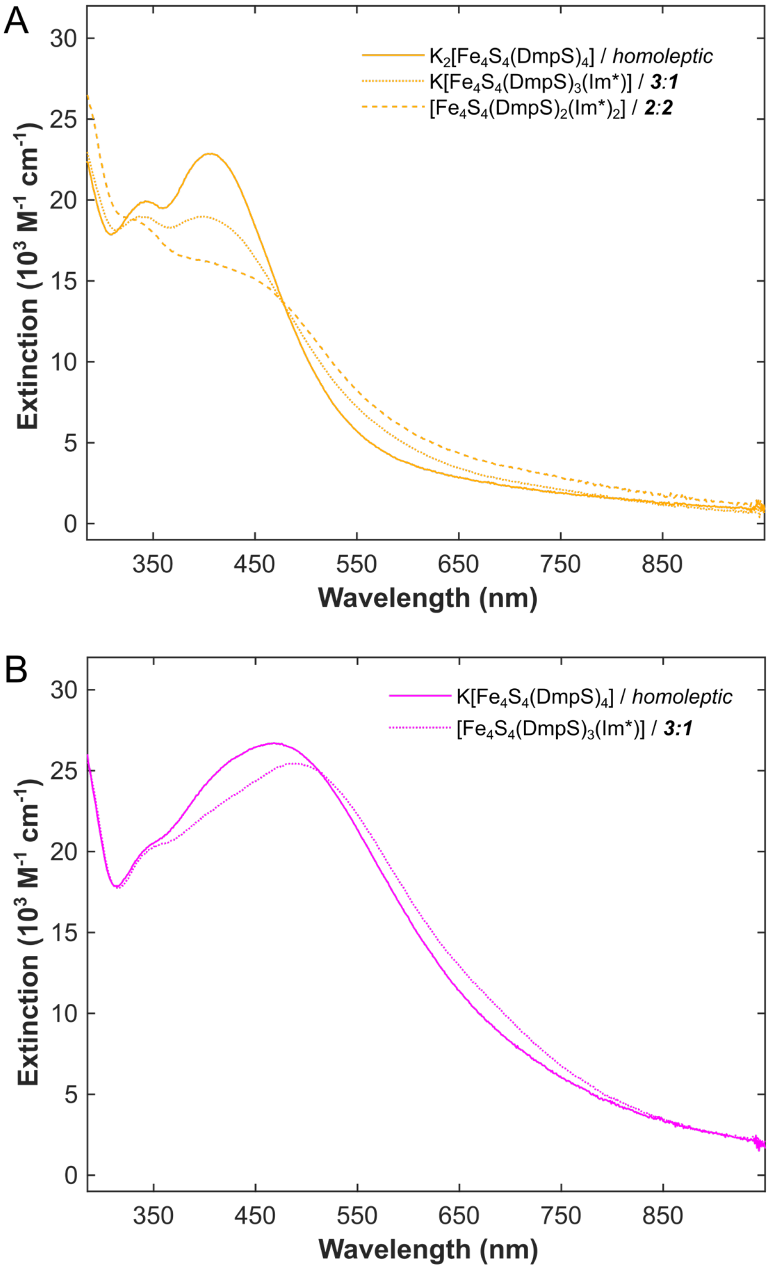

Supplementary Figure 15. (A) UV-vis electronic absorption spectra of $K_2[Fe_4S_4(DmpS)_4]$ (*solid yellow line*), $K[Fe_4S_4(DmpS)_3(Im^*)]$ (*dotted yellow line*) and $[Fe_4S_4(DmpS)_3(Im^*)_2]$ (*dashed yellow line*) recorded in $1{\cdot}10^{-4}$ M toluene solution at room temperature, and, (B) of $K[Fe_4S_4(DmpS)_4]$ (*solid magenta line*) and $[Fe_4S_4(DmpS)_3(Im^*)]$ (*dotted magenta line*) likewise recorded in $1{\cdot}10^{-4}$ M toluene solution at room temperature.

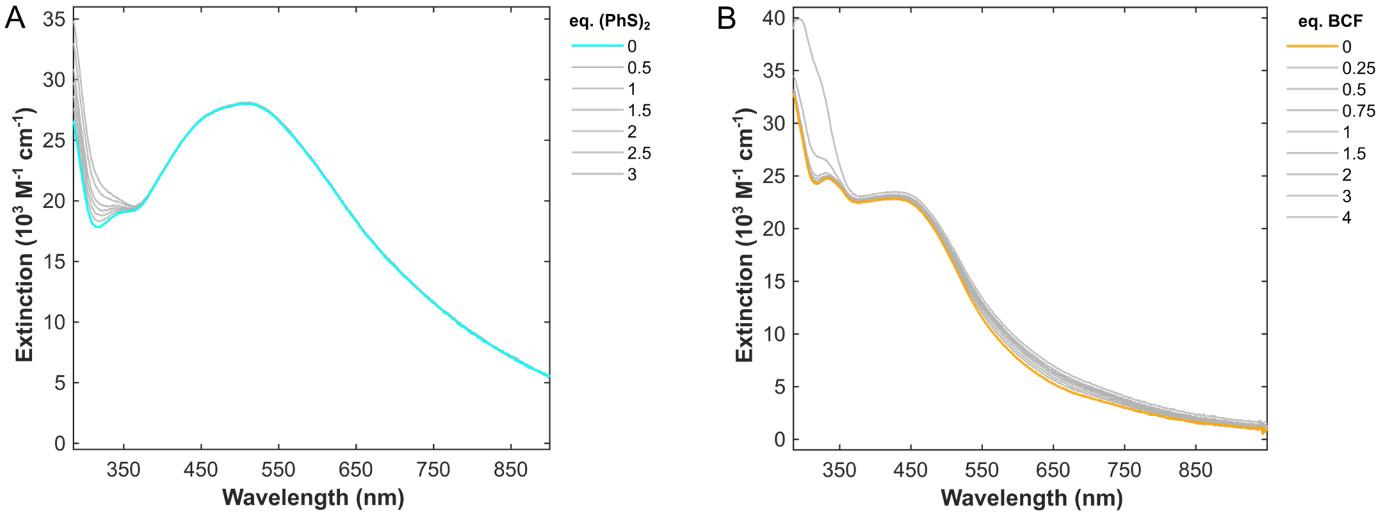

Supplementary Figure 16. (A) UV-vis electronic absorption spectrum of a $1 \cdot 10^{-4}$ M toluene solution of [Fe_4S_4(DmpS)$_4$] and its evolution upon titration with stoichiometric amounts of (PhS)$_2$ (*grey lines*). (B) UV-vis electronic absorption spectrum of a $1 \cdot 10^{-4}$ M toluene solution of [Fe_4S_4(DmpS)$_2$(Im*)$_2$] and its evolution upon titration with stoichiometric amounts of B(C_6F_5)$_3$ (*grey lines*).

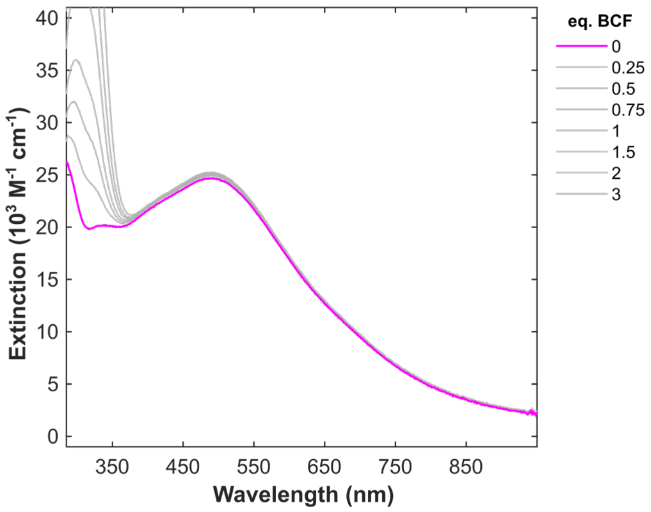

Supplementary Figure 17. UV-vis electronic absorption spectrum of a $1 \cdot 10^{-4}$ M toluene solution of [Fe_4S_4(DmpS)$_3$(Im*)] and its evolution upon titration with stoichiometric amounts of B(C_6F_5)$_3$ (*grey lines*).

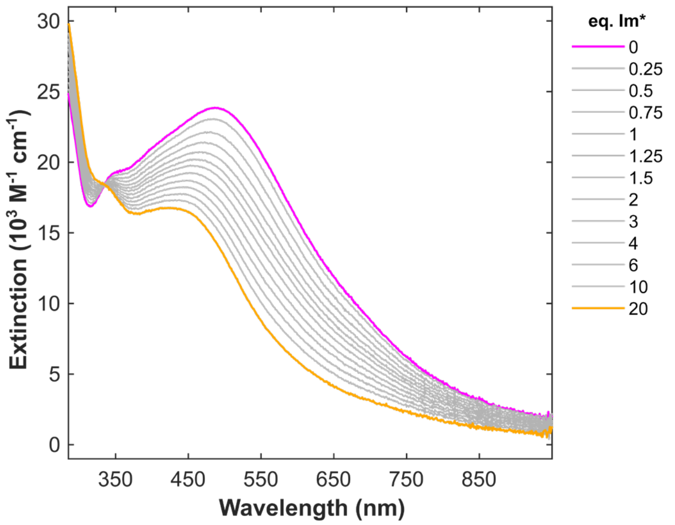

Supplementary Figure 18. UV-vis electronic absorption spectrum of a $1 \cdot 10^{-4}$ M toluene solution of $[Fe_4S_4(DmpS)_3(Im^*)]$ and its evolution upon titration with additional equivalents of Im*.

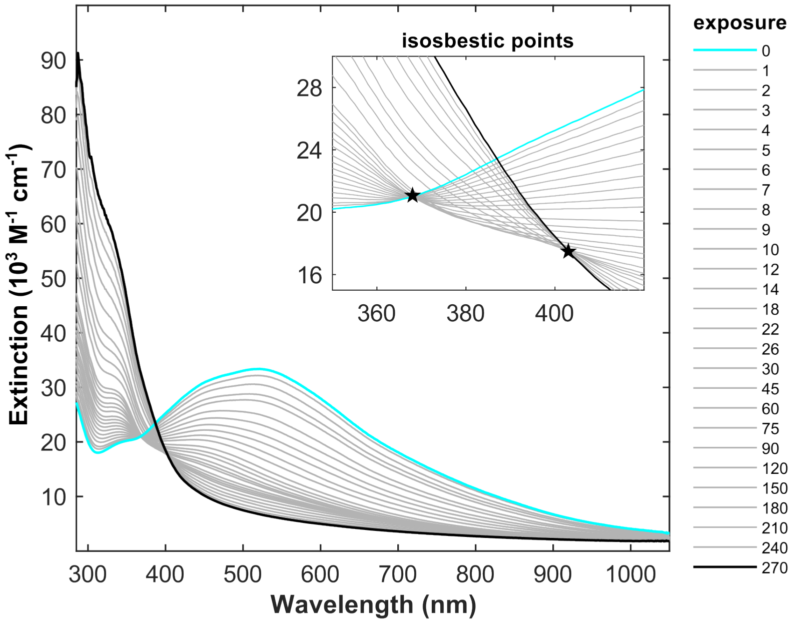

Supplementary Figure 19. UV-vis electronic absorption spectrum of a $1 \cdot 10^{-4}$ M toluene solution of $[Fe_4S_4(DmpS)_4]$ and its evolution upon irradiation with UV light. Exposure times are given in minutes and two isosbestic points are illustrated in the *inset* and marked by *black stars*.

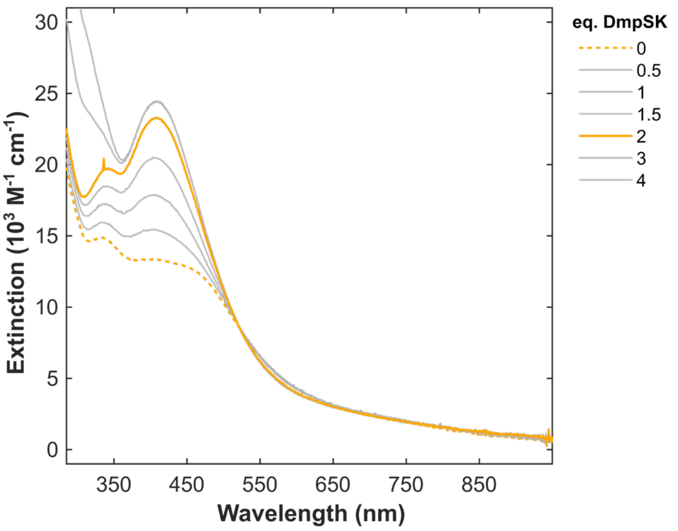

Supplementary Figure 20. UV-vis electronic absorption spectrum of a 1·10^{-4} M toluene solution of [$Fe_4S_4(DmpS)_2(Im^*)_2$] and its evolution upon titration with additional equivalents of DmpSK.

^{1}H and ^{13}C NMR Spectroscopy

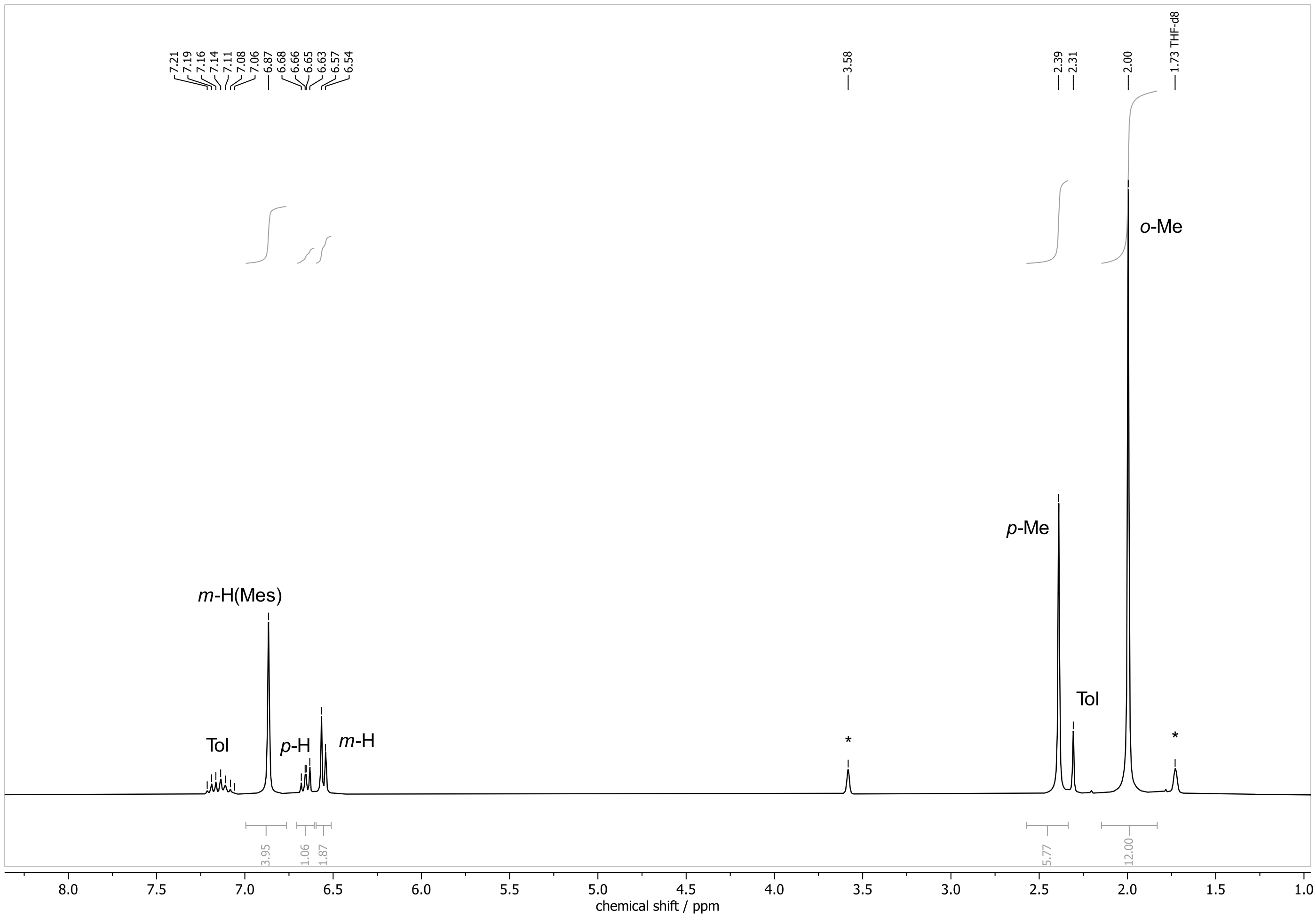

Supplementary Figure 21. 300 MHz ^{1}H NMR spectrum of DmpSK recorded at room temperature in THF-*d*8 (*).

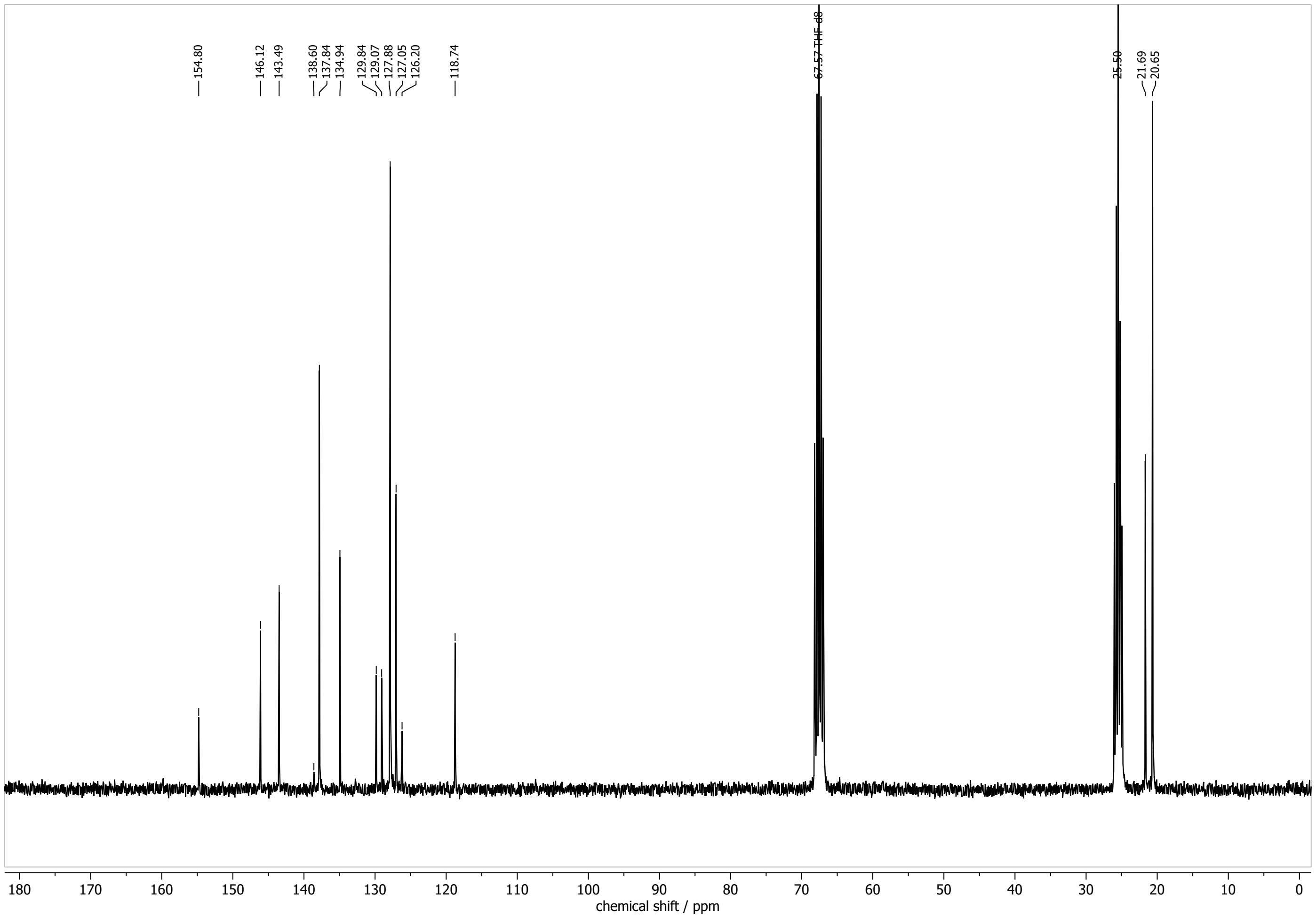

Supplementary Figure 22. 75 MHz ^{13}C NMR spectrum of DmpSK recorded at room temperature in THF-*d*8.

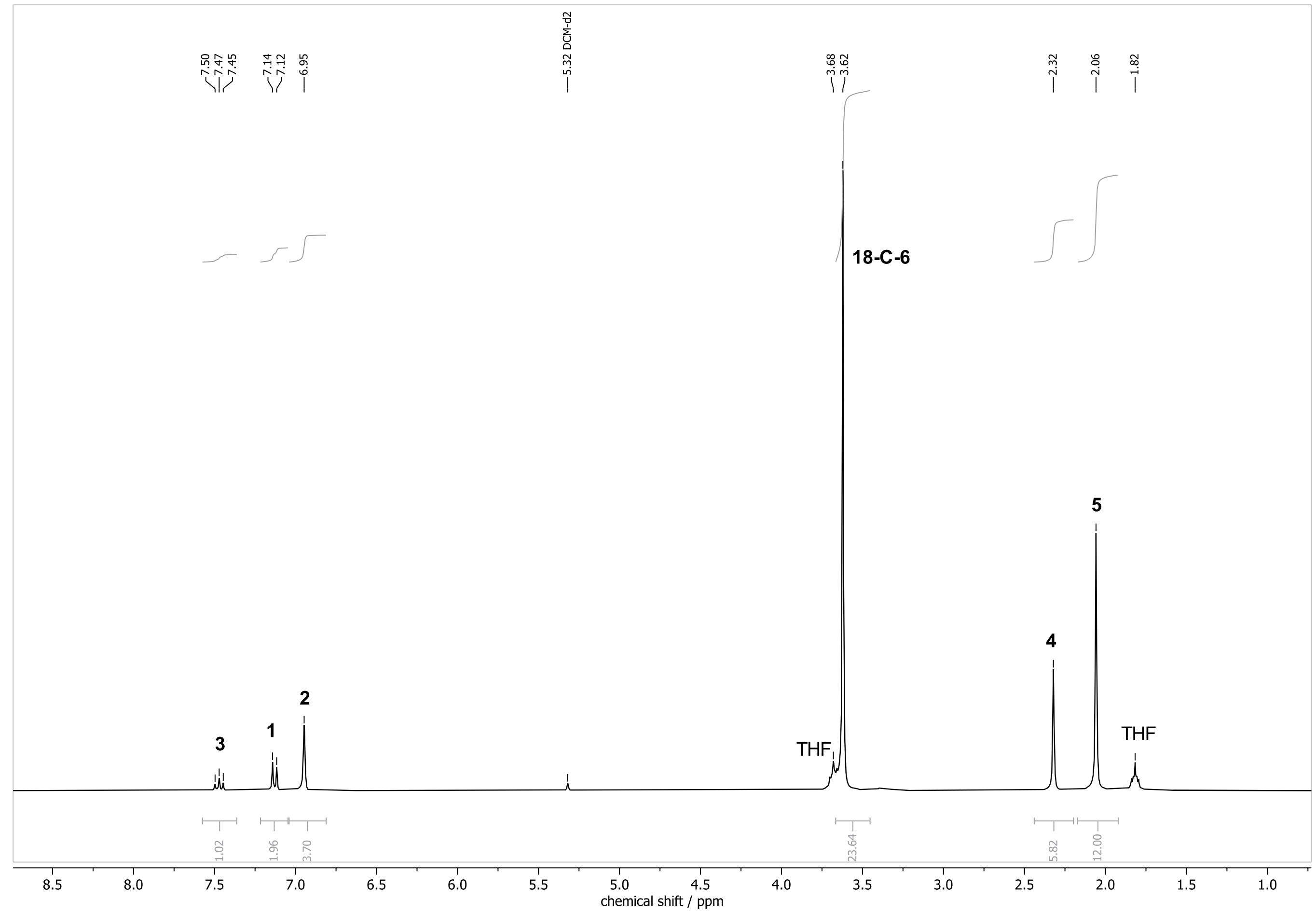

Supplementary Figure 23. 300 MHz ^{1}H NMR spectrum of DmpS$^{[18\text{-}C\text{-}6]}$K recorded at room temperature in CD_2Cl_2.

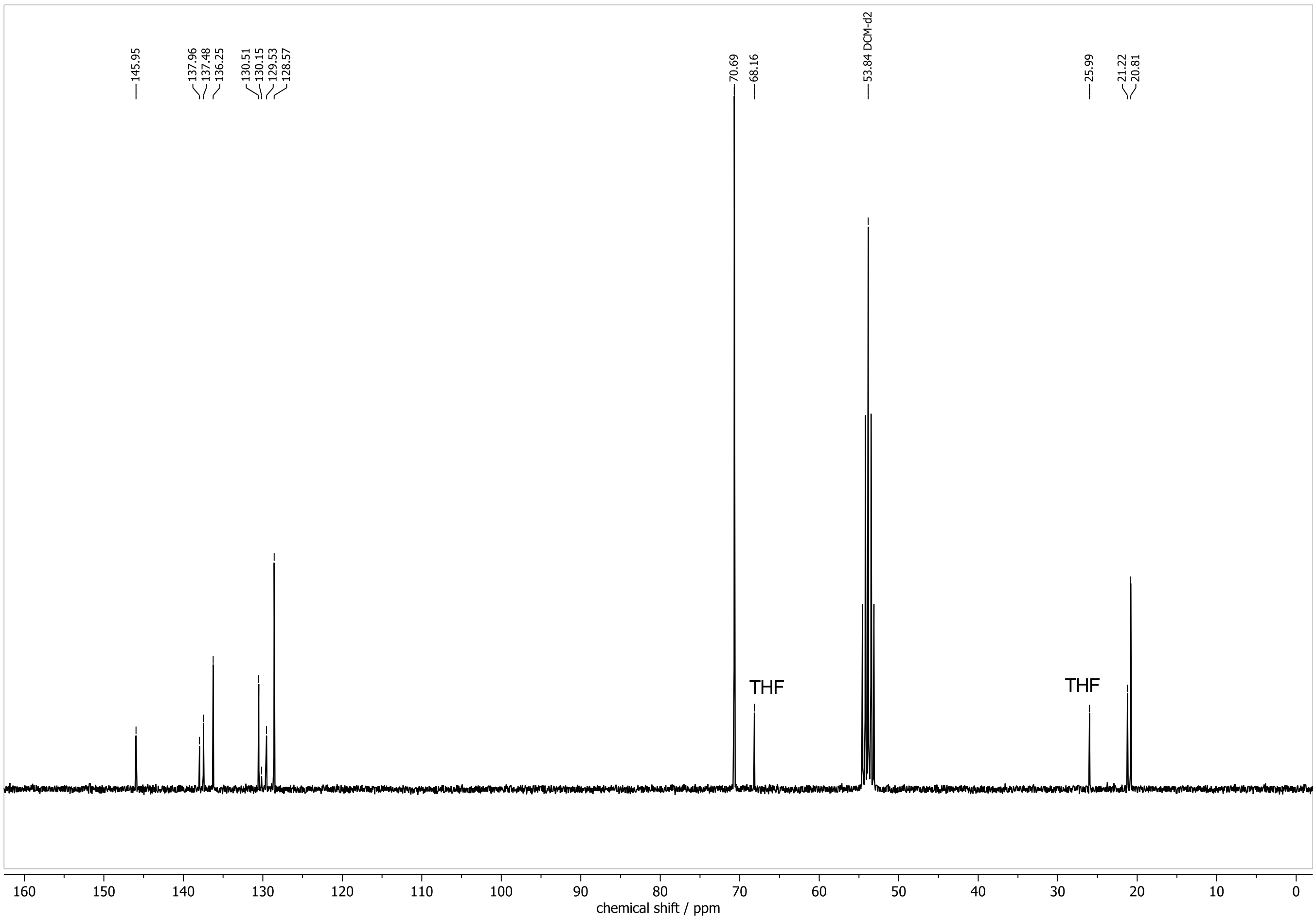

Supplementary Figure 24. 75 MHz ^{13}C NMR spectrum of DmpS[18-C-6]K recorded at room temperature in CD_2Cl_2.

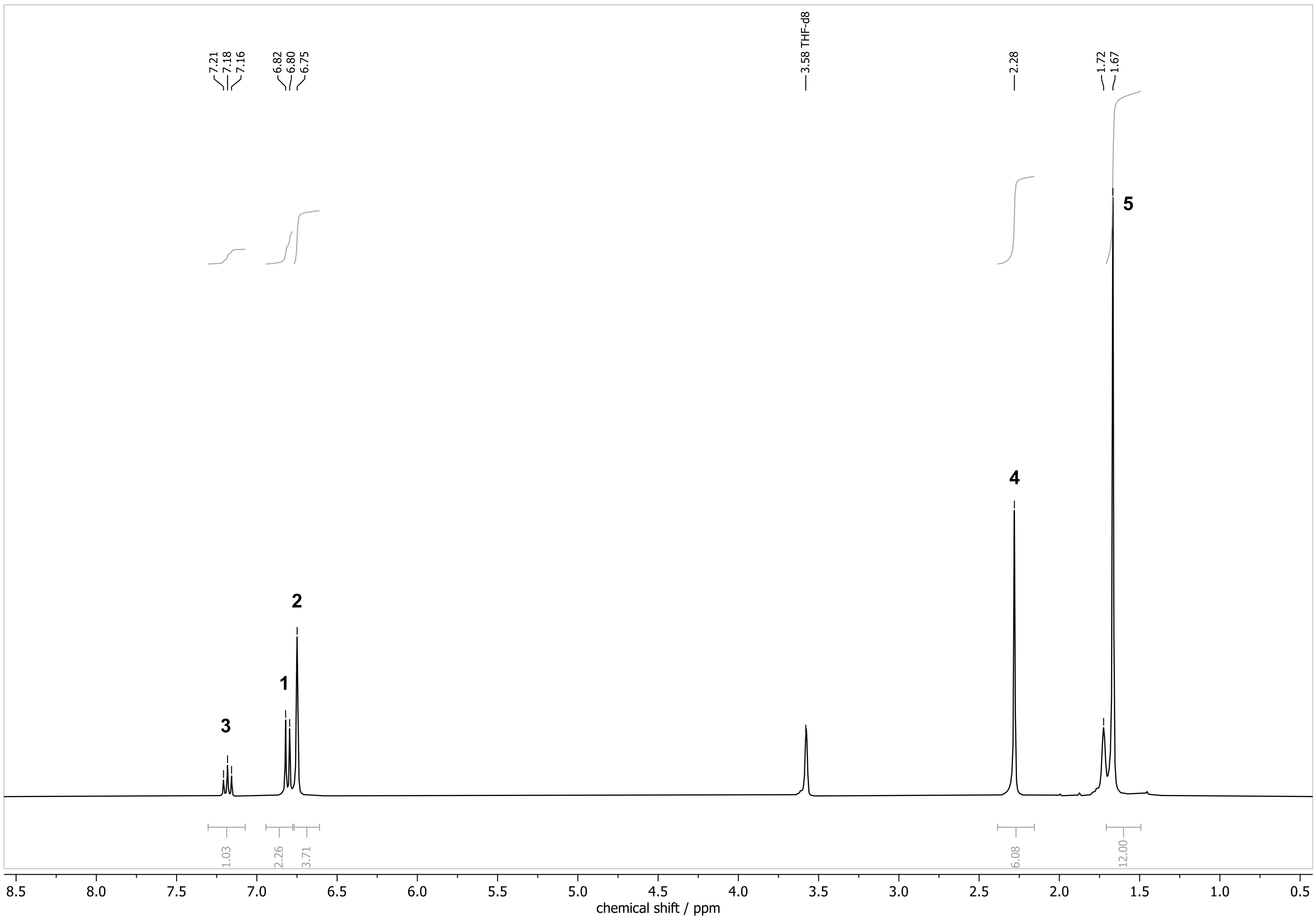

Supplementary Figure 25. 300 MHz ^{1}H NMR spectrum of $(DmpS)_2$ recorded at room temperature in THF-*d*8.

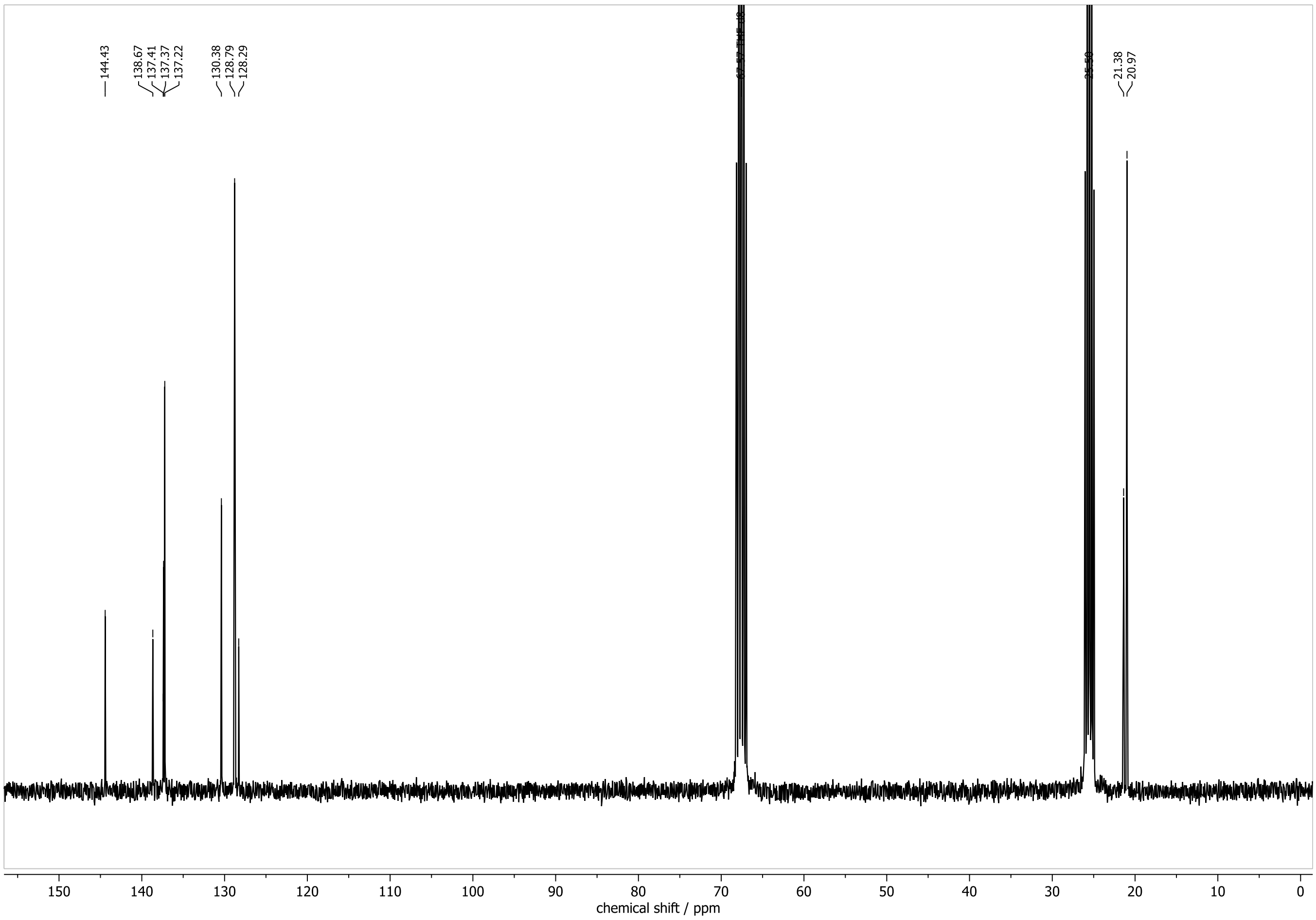

Supplementary Figure 26. 75 MHz ^{13}C NMR spectrum of $(DmpS)_2$ recorded at room temperature in THF-*d*8.

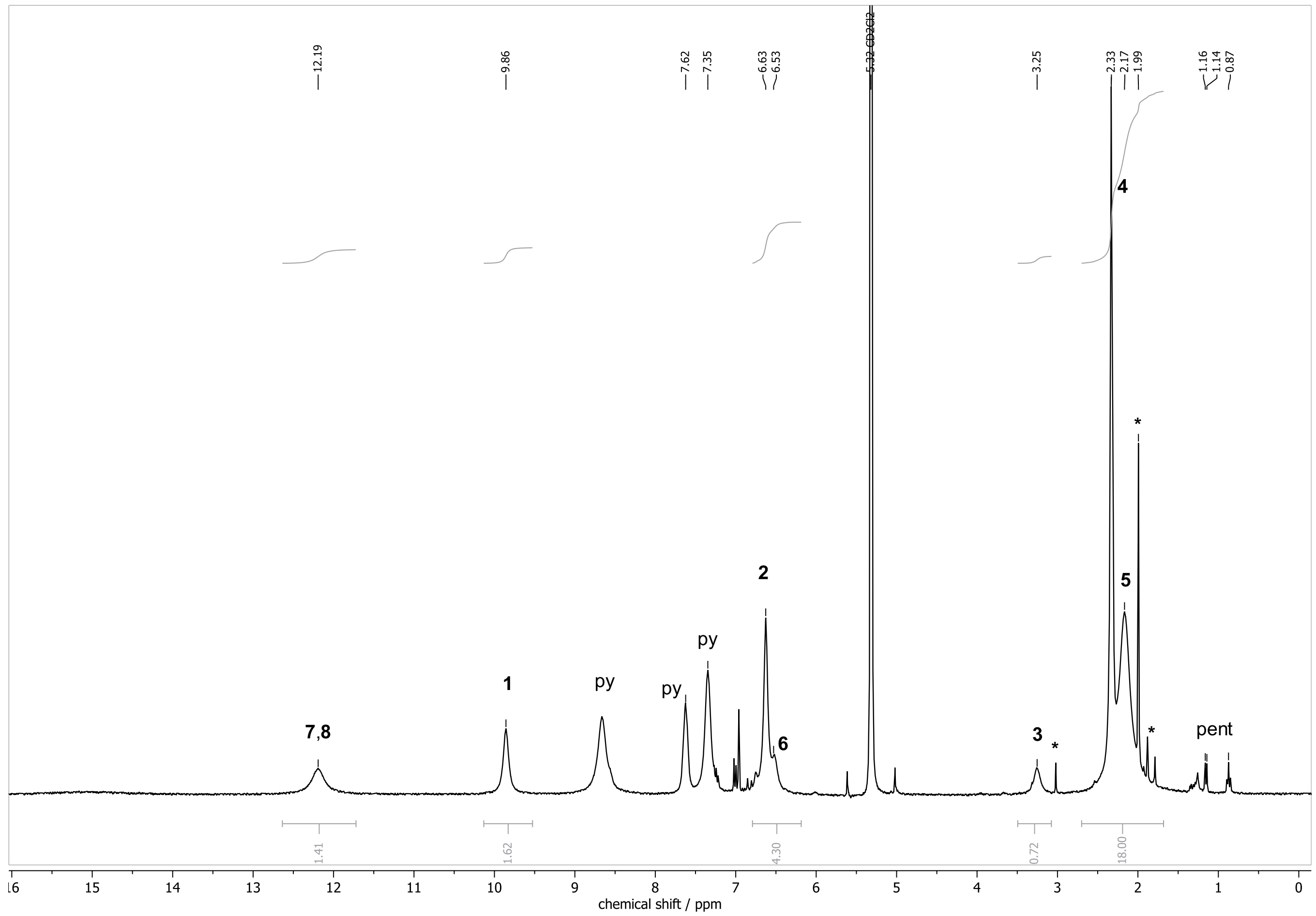

Supplementary Figure 27. 300 MHz ^{1}H NMR spectrum of $[Fe_2S_2(DmpS)_2(py)_2]$ recorded at room temperature in CD_2Cl_2.

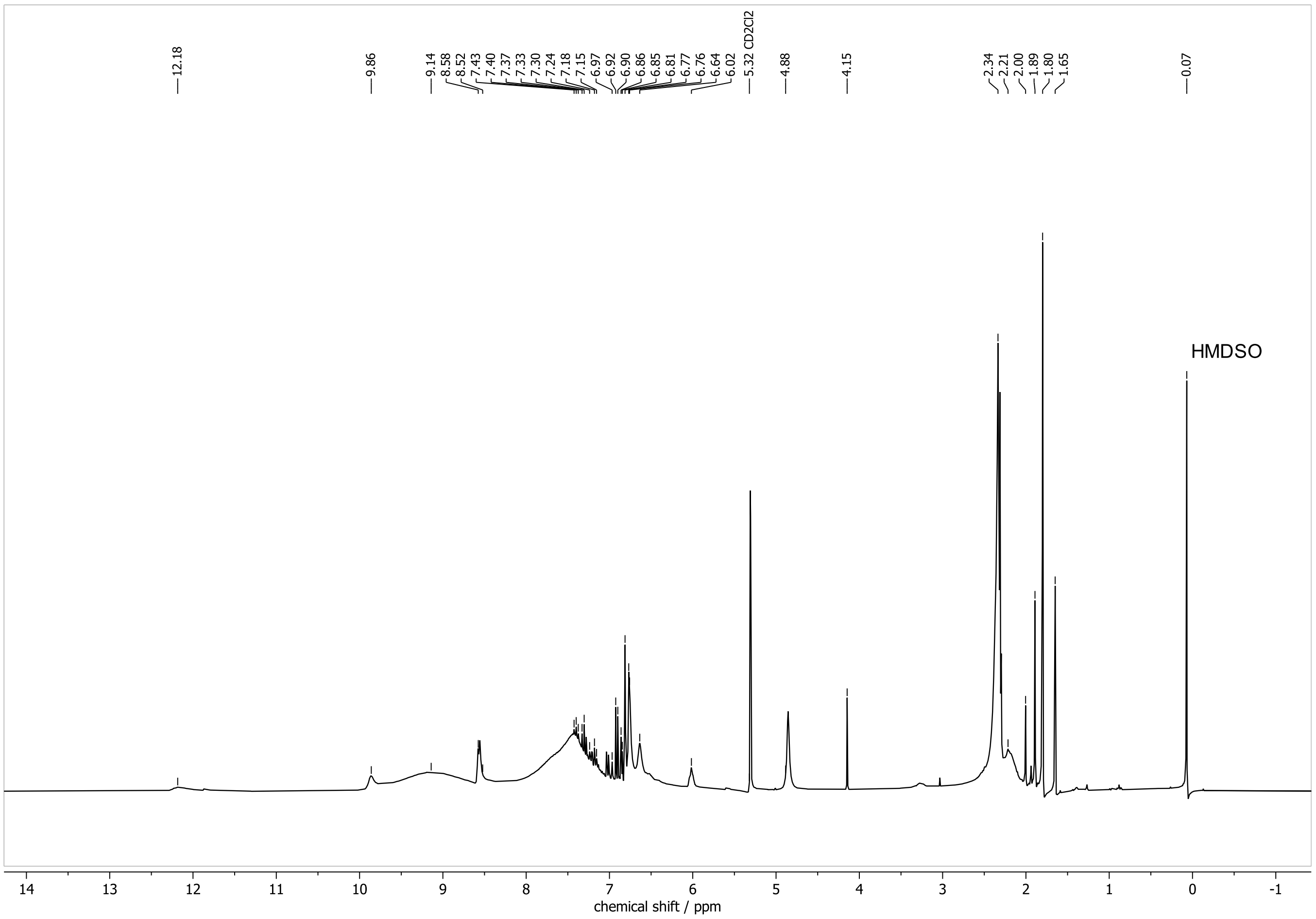

Supplementary Figure 28. 300 MHz ^{1}H NMR spectrum of $[Fe_4S_4(DmpS)_4]$ in presence of 20 equivalents of pyridine recorded at room temperature in CD_2Cl_2.

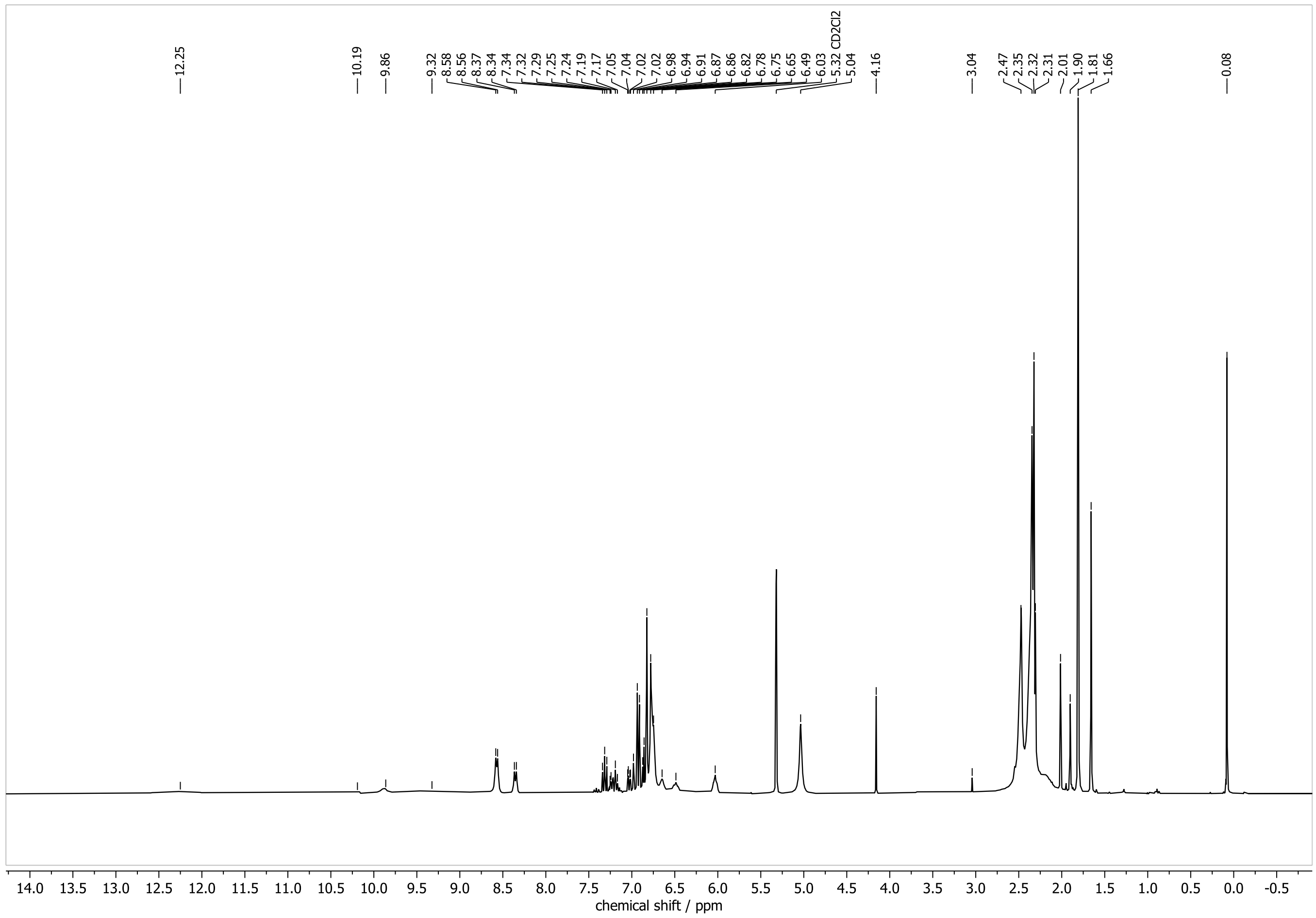

Supplementary Figure 29. 300 MHz ^{1}H NMR spectrum of $[Fe_4S_4(DmpS)_4]$ in presence of 4 equivalents of pyridine recorded at room temperature in CD_2Cl_2.

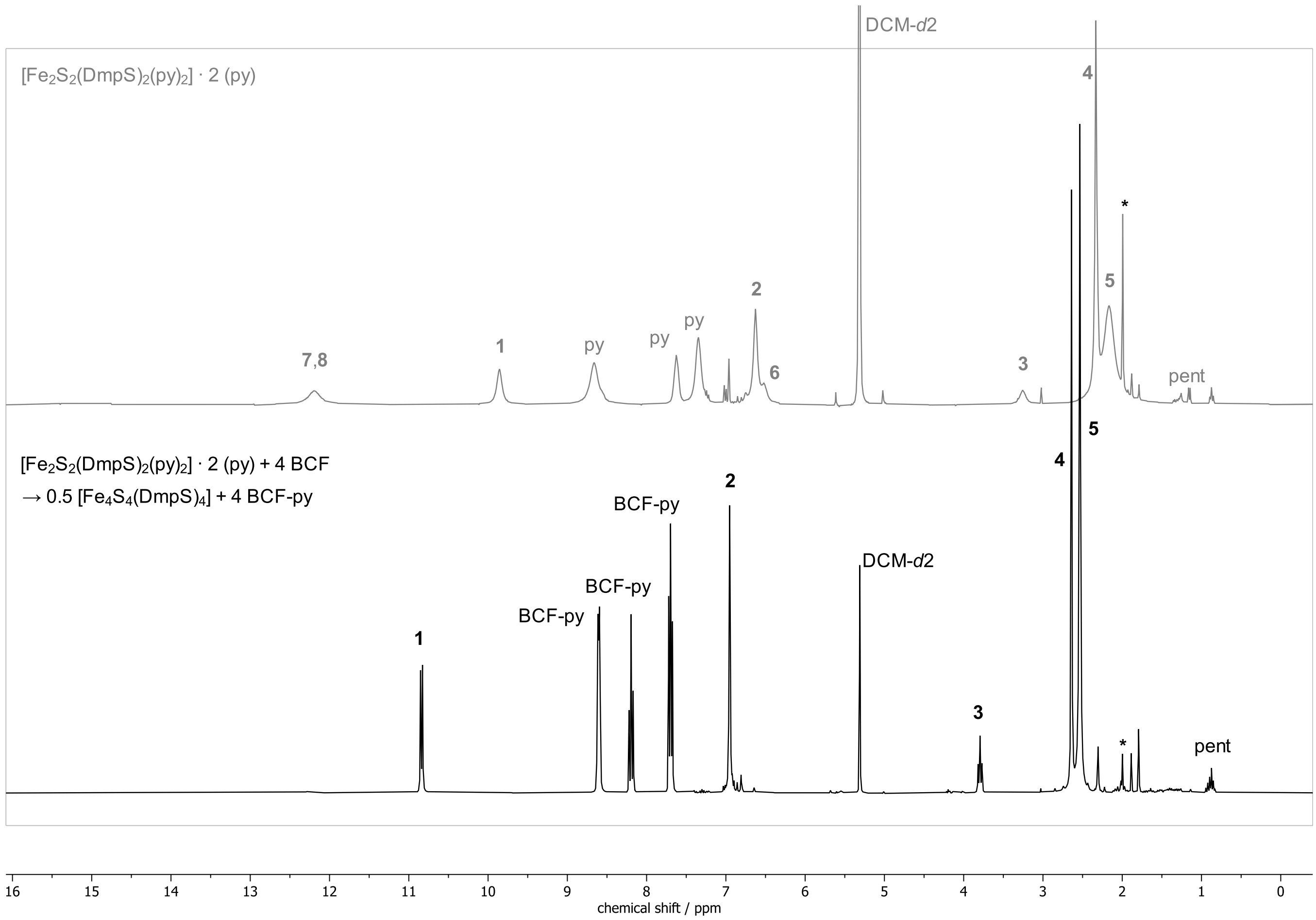

Supplementary Figure 30. (Top) 300 MHz ^{1}H NMR spectrum of $[Fe_2S_2(DmpS)_2(py)_2]$ recorded at room temperature in CD_2Cl_2 (*grey*). (Bottom) Spectrum recorded after addition of 4 equivalents of $B(C_6F_5)_3$, evidencing formation of py·$B(C_6F_5)_3$ as well as $[Fe_4S_4(DmpS)_4]$ (*black*).

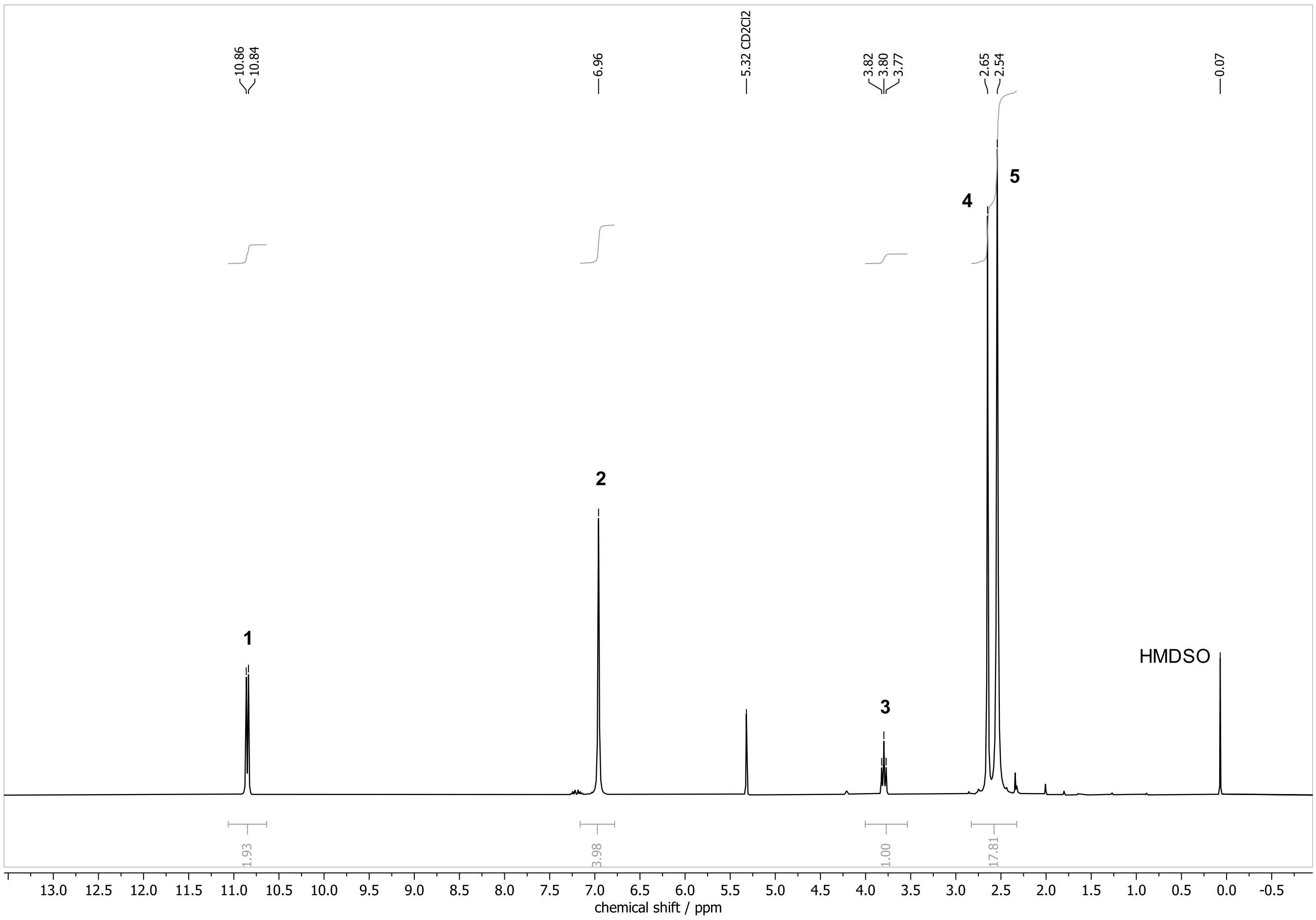

Supplementary Figure 31. 300 MHz ^{1}H NMR spectrum of a neat sample of [Fe_4S_4(DmpS)$_4$] recorded at room temperature in CD_2Cl_2. The signals are identical to the ones observed in Supplementary Fig. 30 for the *in-situ* generation of the all-ferric cubane from [Fe_2S_2(DmpS)$_2$(py)$_2$]

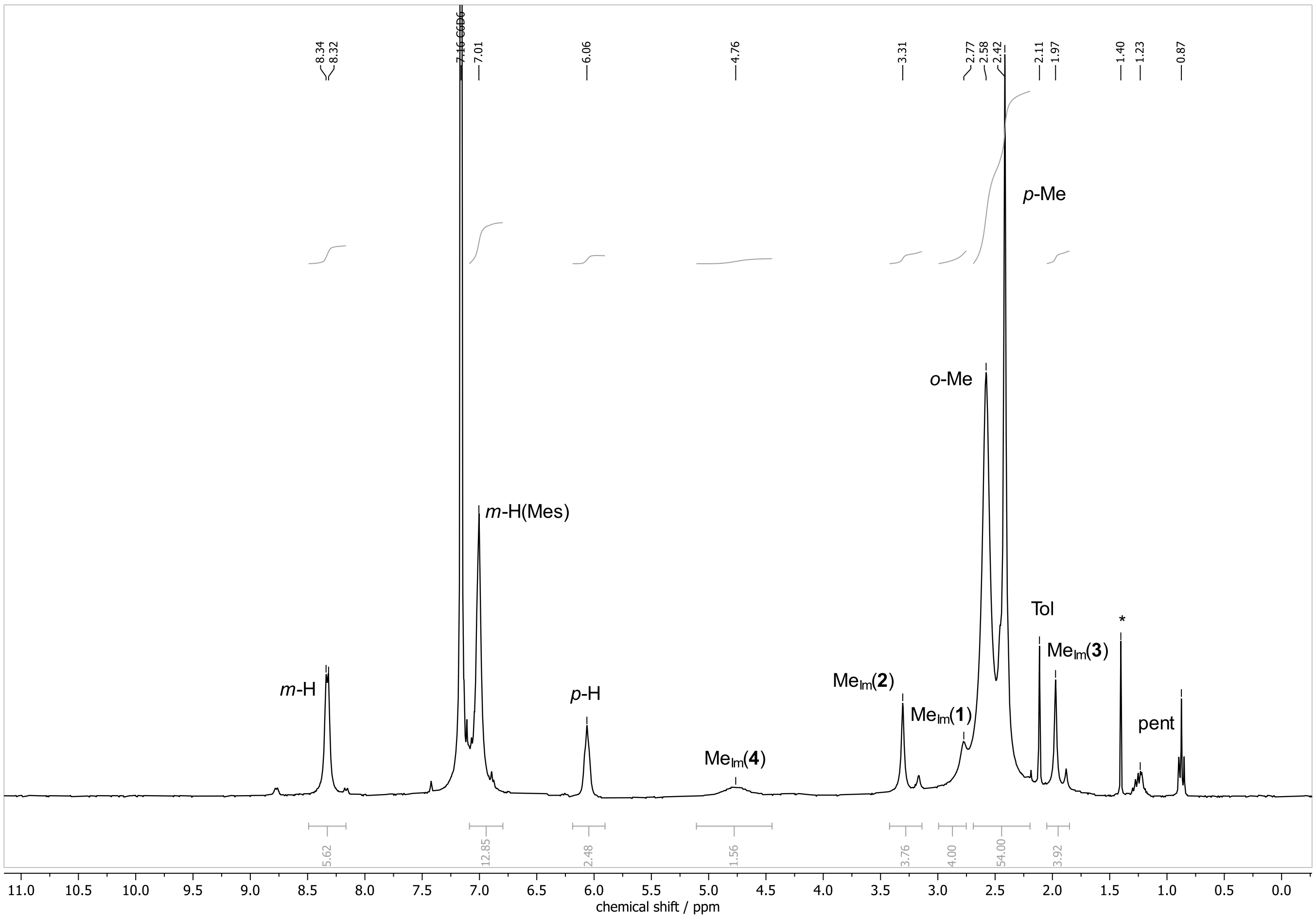

Supplementary Figure 32. 300 MHz ^{1}H NMR spectrum of $K[Fe_4S_4(DmpS)_3(Im^*)]$ recorded at room temperature in C_6D_6.

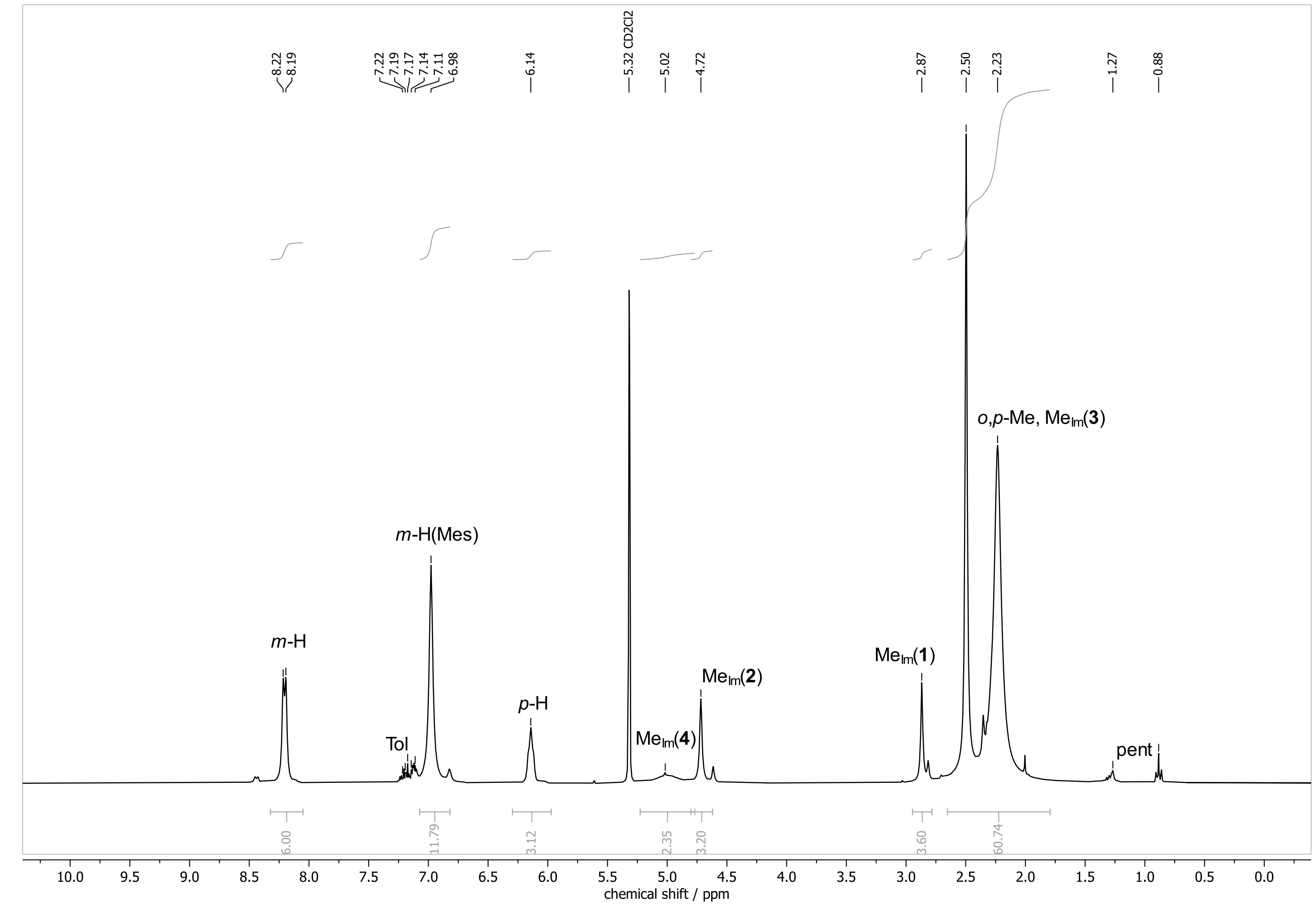

Supplementary Figure 33. 300 MHz ^{1}H NMR spectrum of K[$Fe_4S_4(DmpS)_3(Im^*)$] recorded at room temperature in CD_2Cl_2.

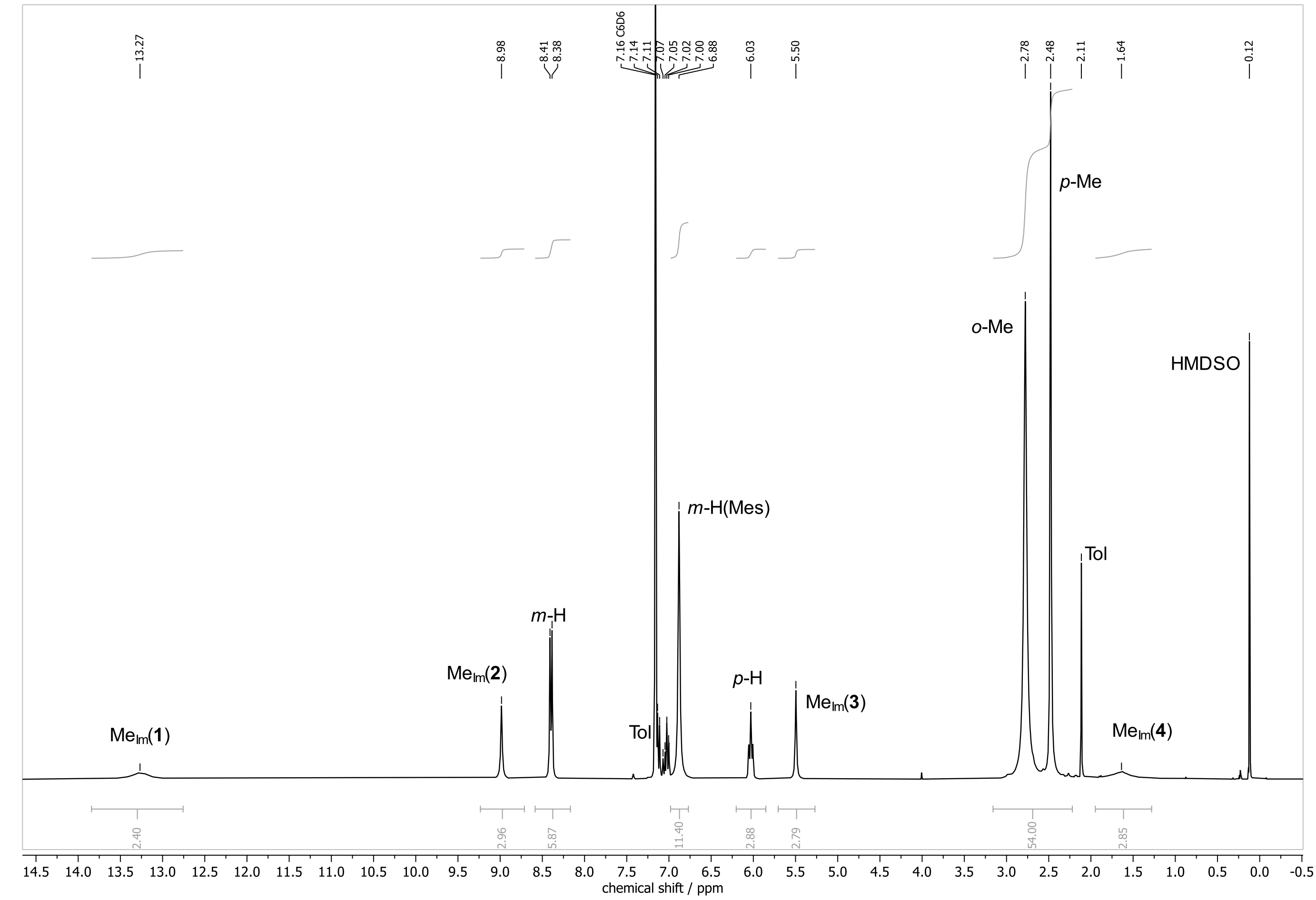

Supplementary Figure 34. 300 MHz ^{1}H NMR spectrum of [Fe_4S_4(DmpS)$_3$(Im*)] recorded at room temperature in C_6D_6.

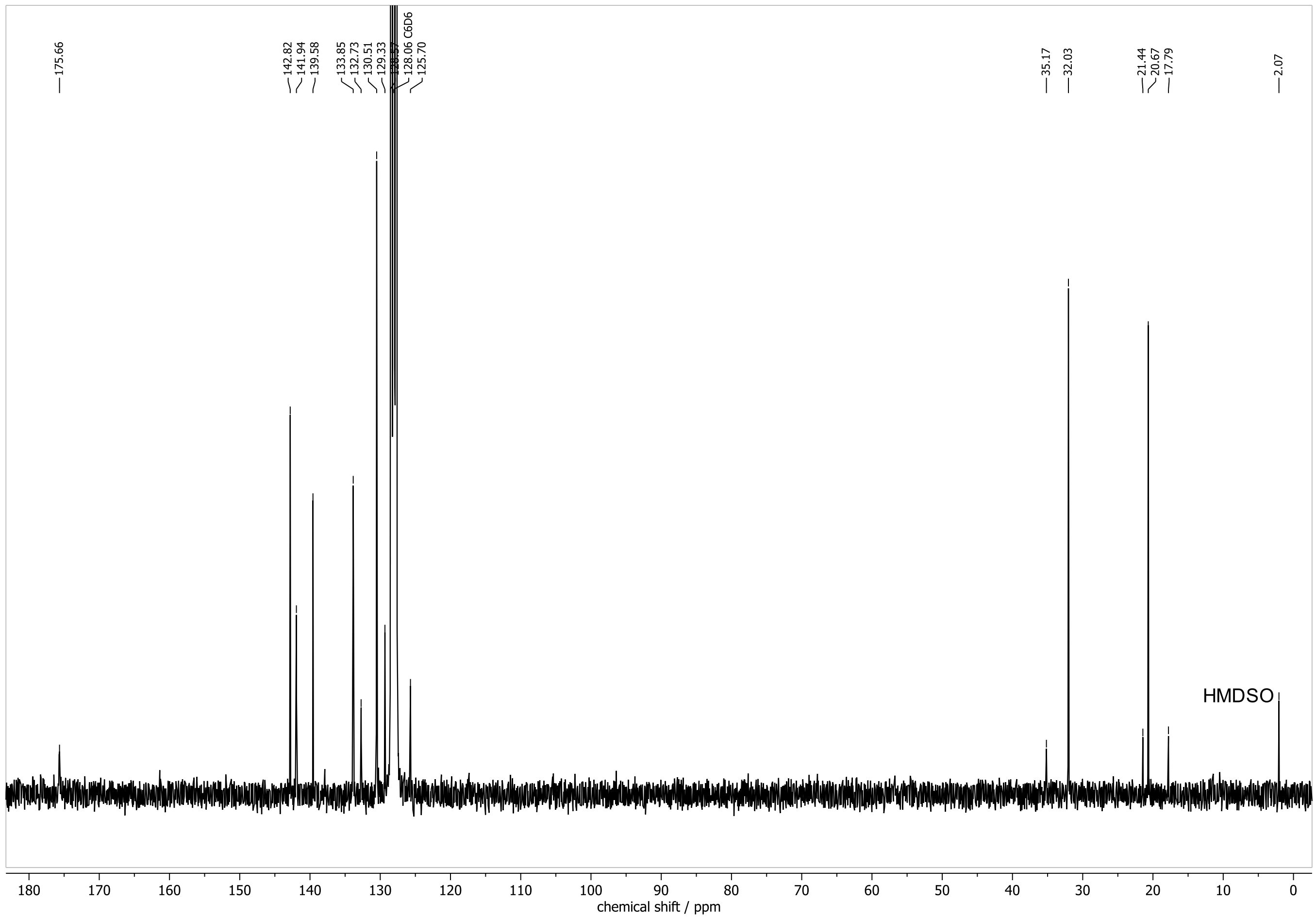

Supplementary Figure 35. 75 MHz ^{13}C NMR spectrum of $[Fe_4S_4(DmpS)_3(Im^*)]$ recorded at room temperature in C_6D_6.

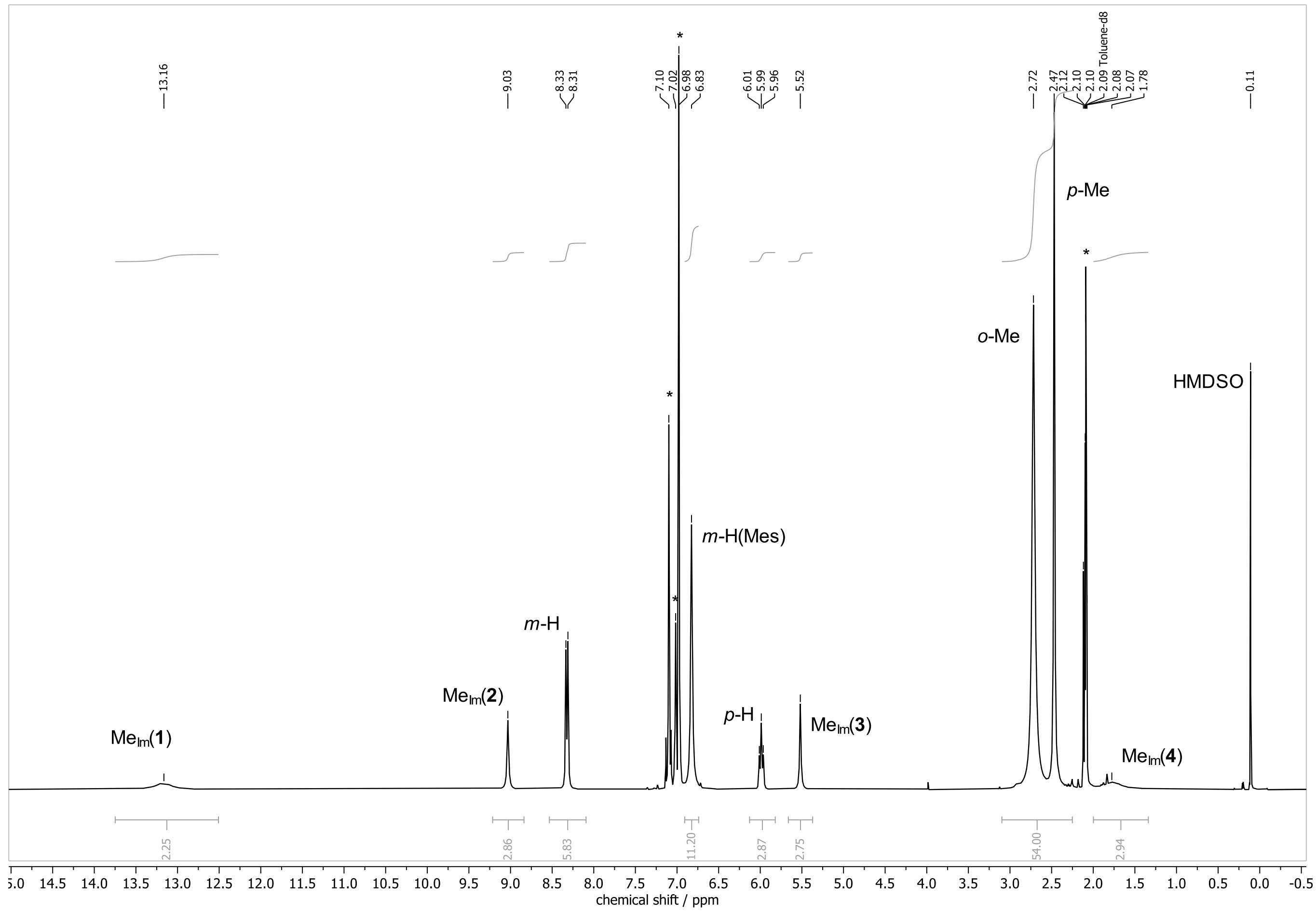

Supplementary Figure 36. 300 MHz ^{1}H NMR spectrum of [$Fe_4S_4(DmpS)_3(Im^*)$] recorded at room temperature in toluene-*d*8 (*).

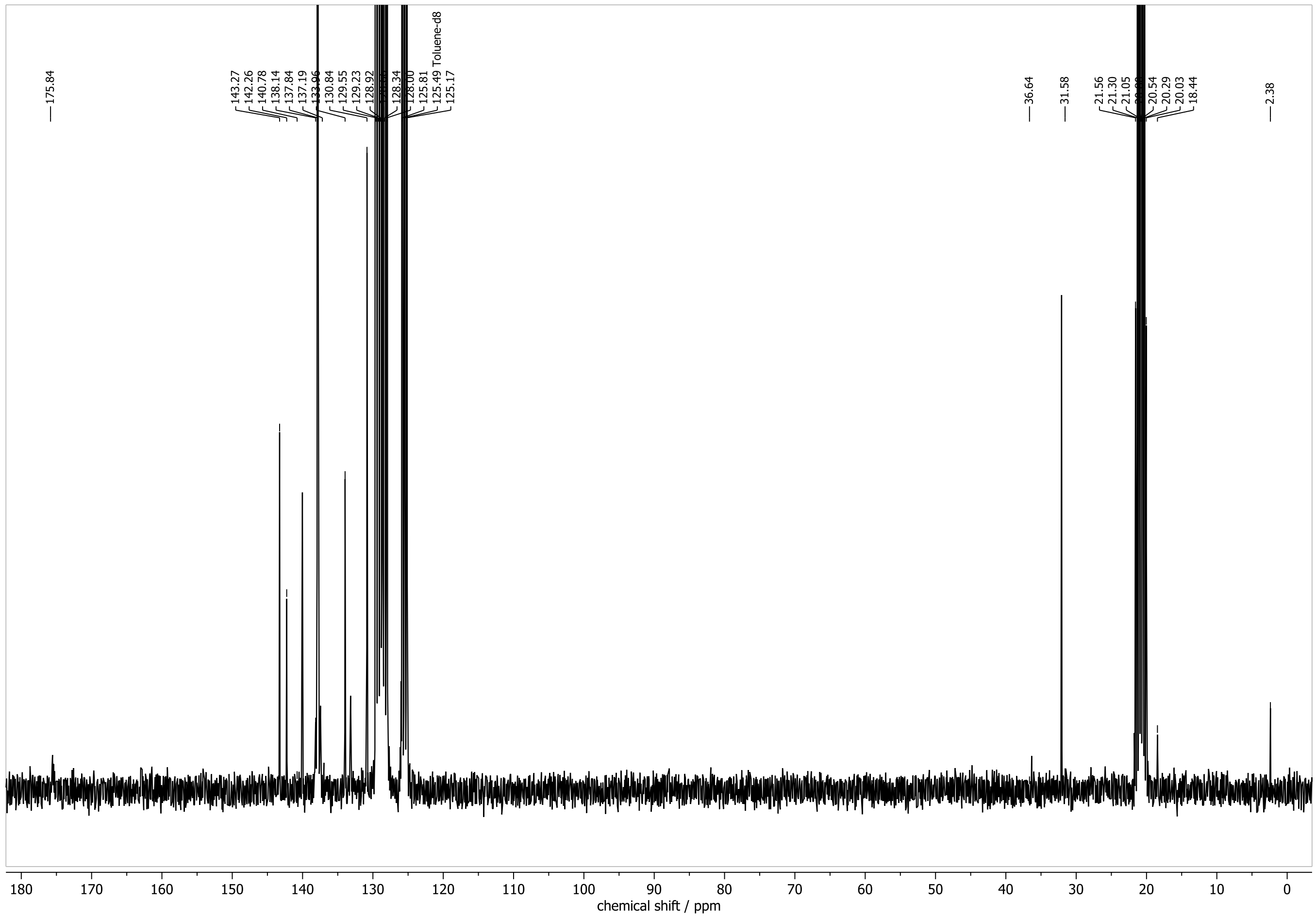

Supplementary Figure 37. 75 MHz ^{13}C NMR spectrum of [Fe_4S_4(DmpS)$_3$(Im*)] recorded at room temperature in toluene-*d*8.

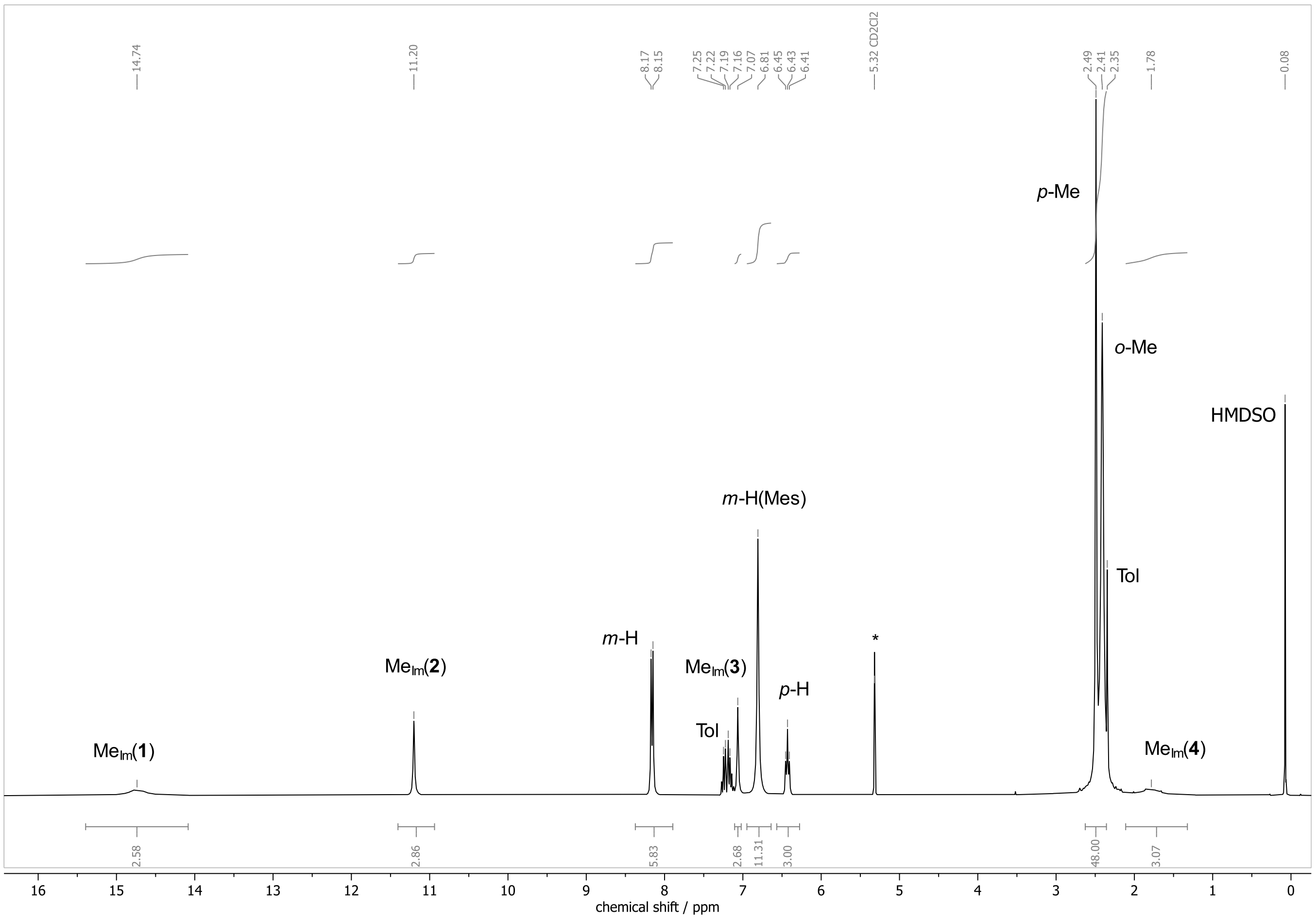

Supplementary Figure 38. 300 MHz ^{1}H NMR spectrum of [$Fe_4S_4(DmpS)_3(Im^*)$] recorded at room temperature in CD_2Cl_2.

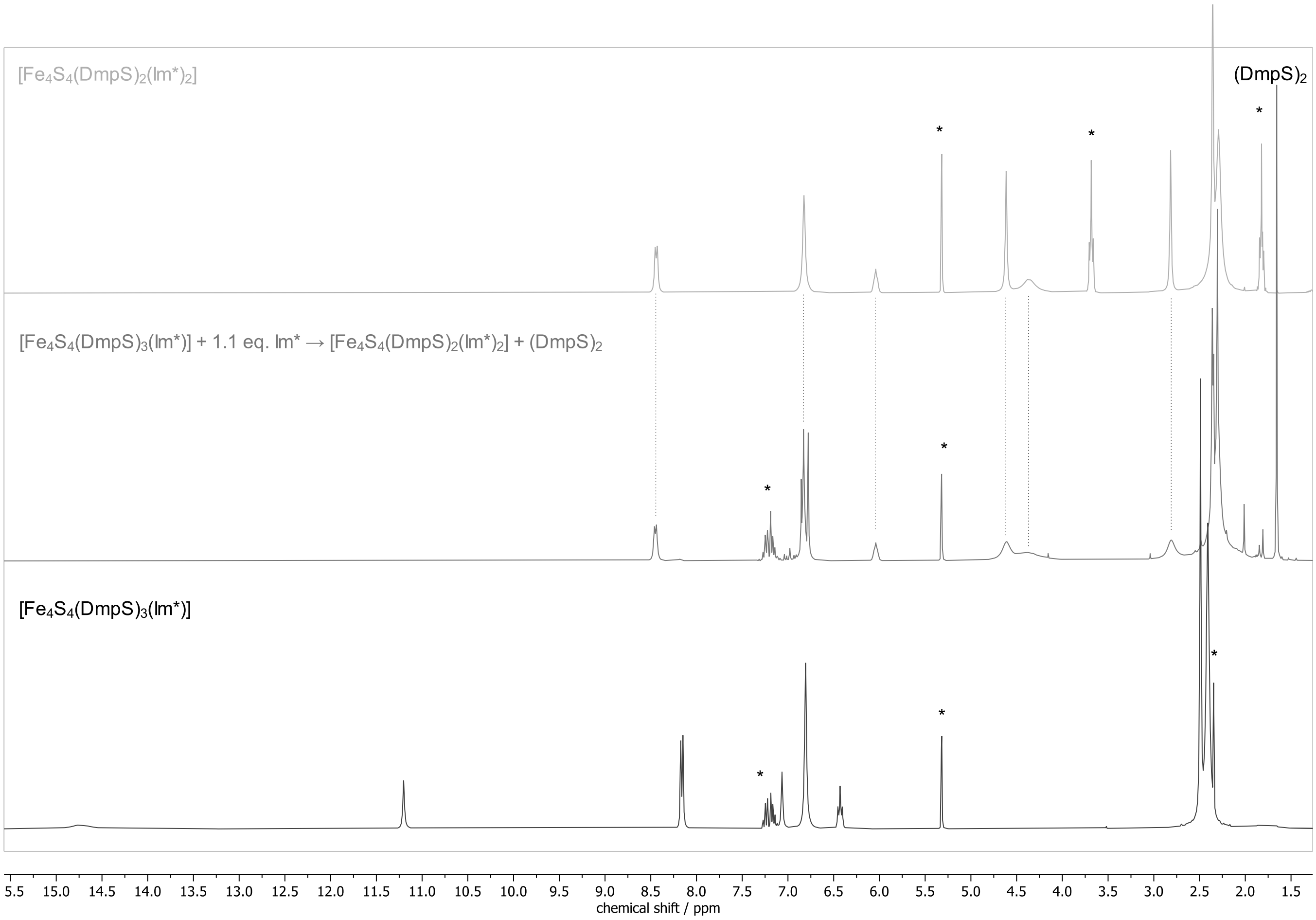

Supplementary Figure 39. 300 MHz ^{1}H NMR spectra of $[Fe_4S_4(DmpS)_3(Im^*)]$ (*bottom*), $[Fe_4S_4(DmpS)_2(Im^*)_2]$ (*top*) as well as $[Fe_4S_4(DmpS)_3(Im^*)]$ in presence of 1.1 equiv. of Im* (*middle*) recorded at room temperature in CD_2Cl_2. Solvent residual signals (from the deuterated solvent as well as co-crystallized solvent) are marked with asterisks (*).

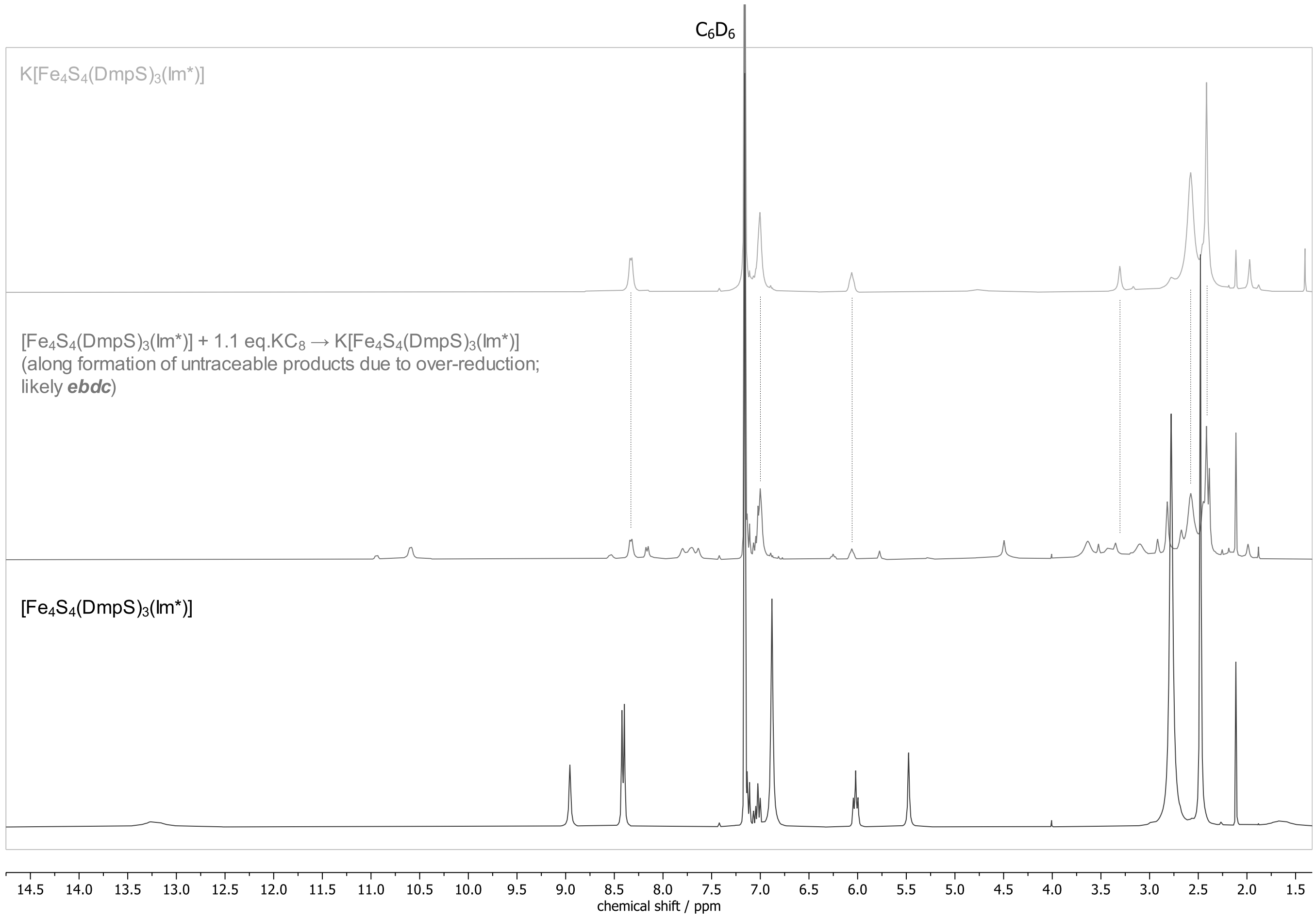

Supplementary Figure 40. 300 MHz ^{1}H NMR spectra of $[Fe_4S_4(DmpS)_3(Im^*)]$ (*bottom*), $K[Fe_4S_4(DmpS)_3(Im^*)]$ (*top*) as well as $[Fe_4S_4(DmpS)_3(Im^*)]$ in presence of 1.1 equiv. of KC_8 (*middle*) recorded at room temperature in C_6D_6.

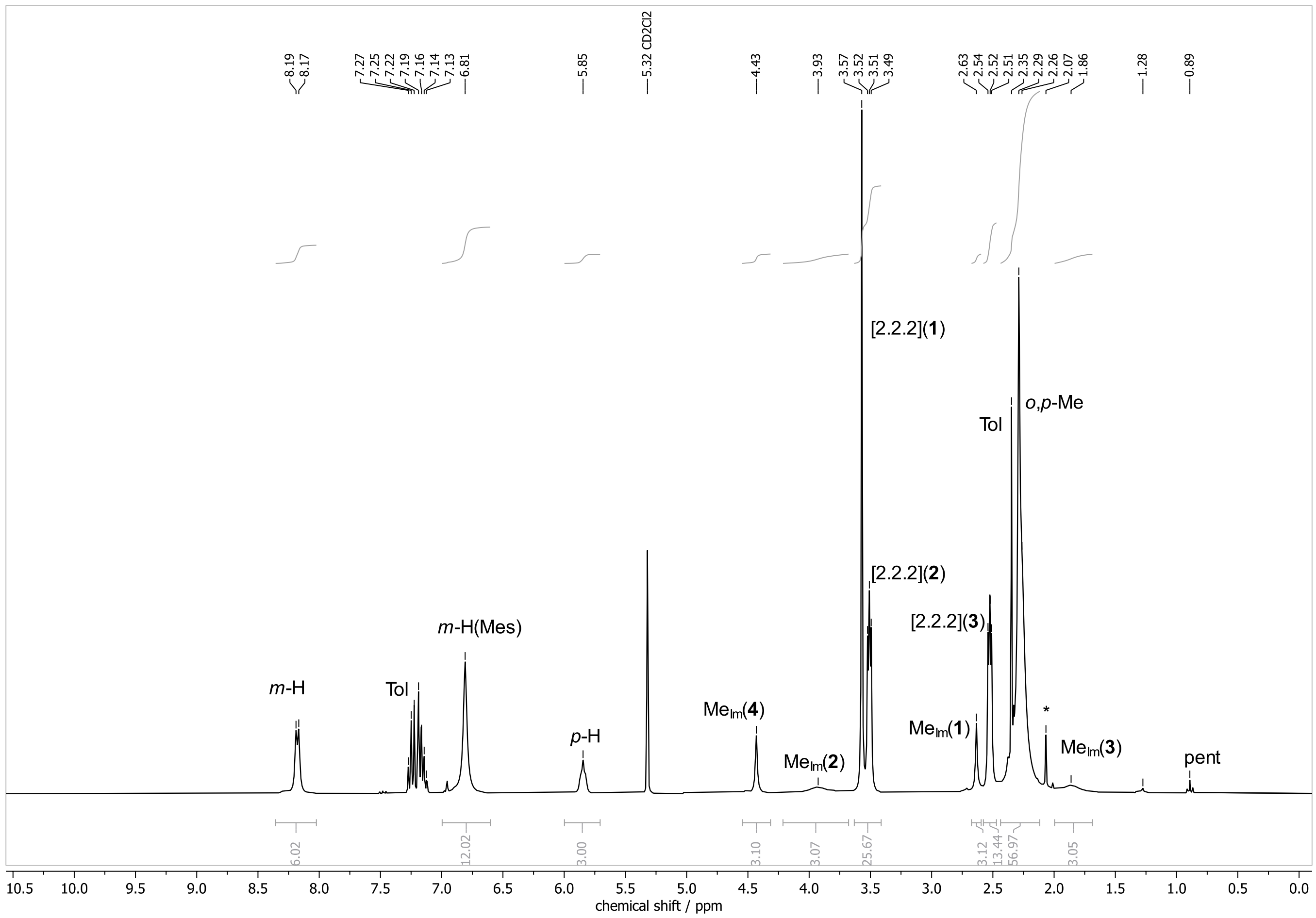

Supplementary Figure 41. 300 MHz ^{1}H NMR spectrum of $^{[2.2.2]}K[Fe_4S_4(DmpS)_3(Im^*)]$ recorded at room temperature in CD_2Cl_2.

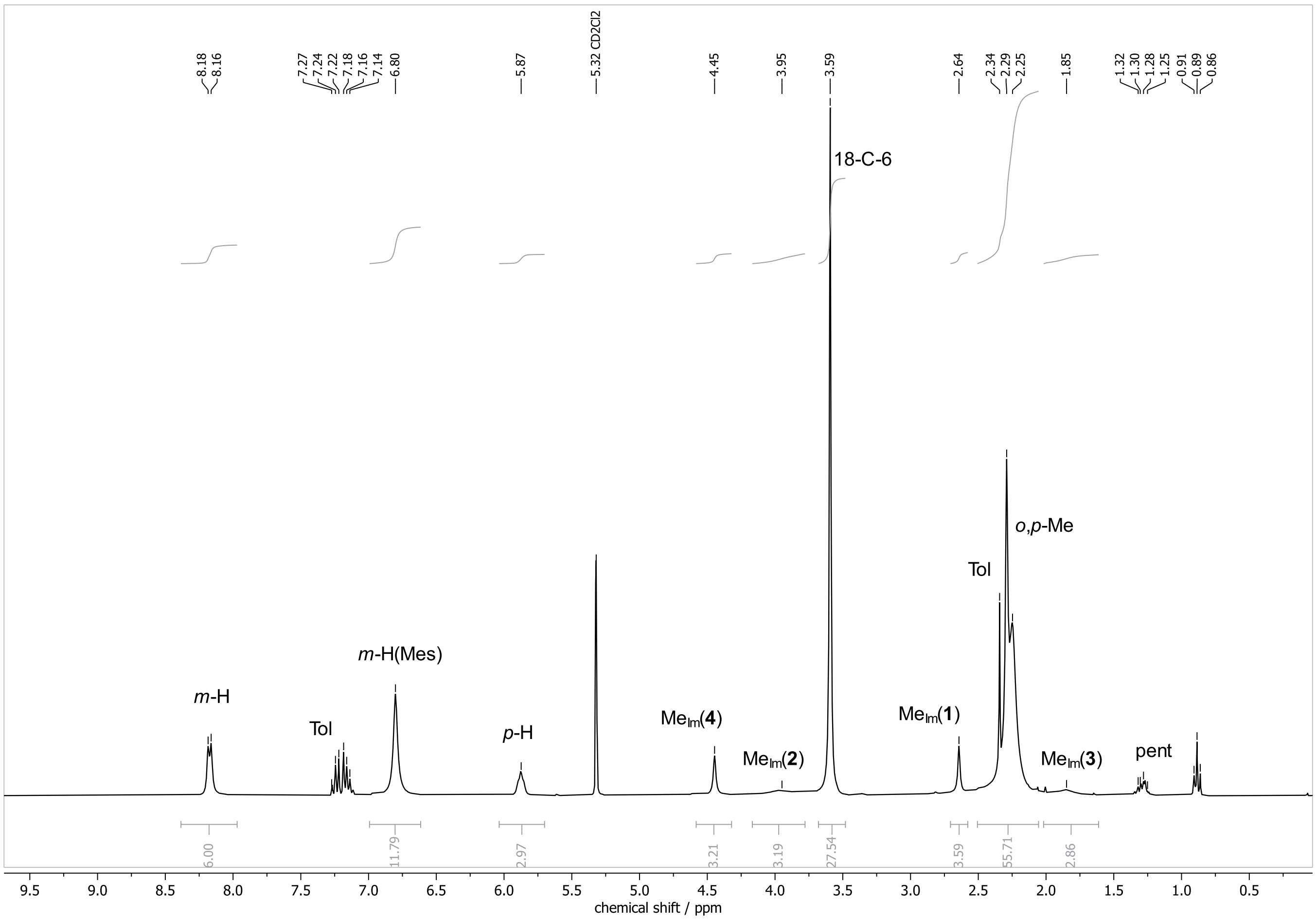

Supplementary Figure 42. 300 MHz ^{1}H NMR spectrum of $^{[18\text{-}C\text{-}6]}K[Fe_4S_4(DmpS)_3(Im^*)]$ recorded at room temperature in CD_2Cl_2.

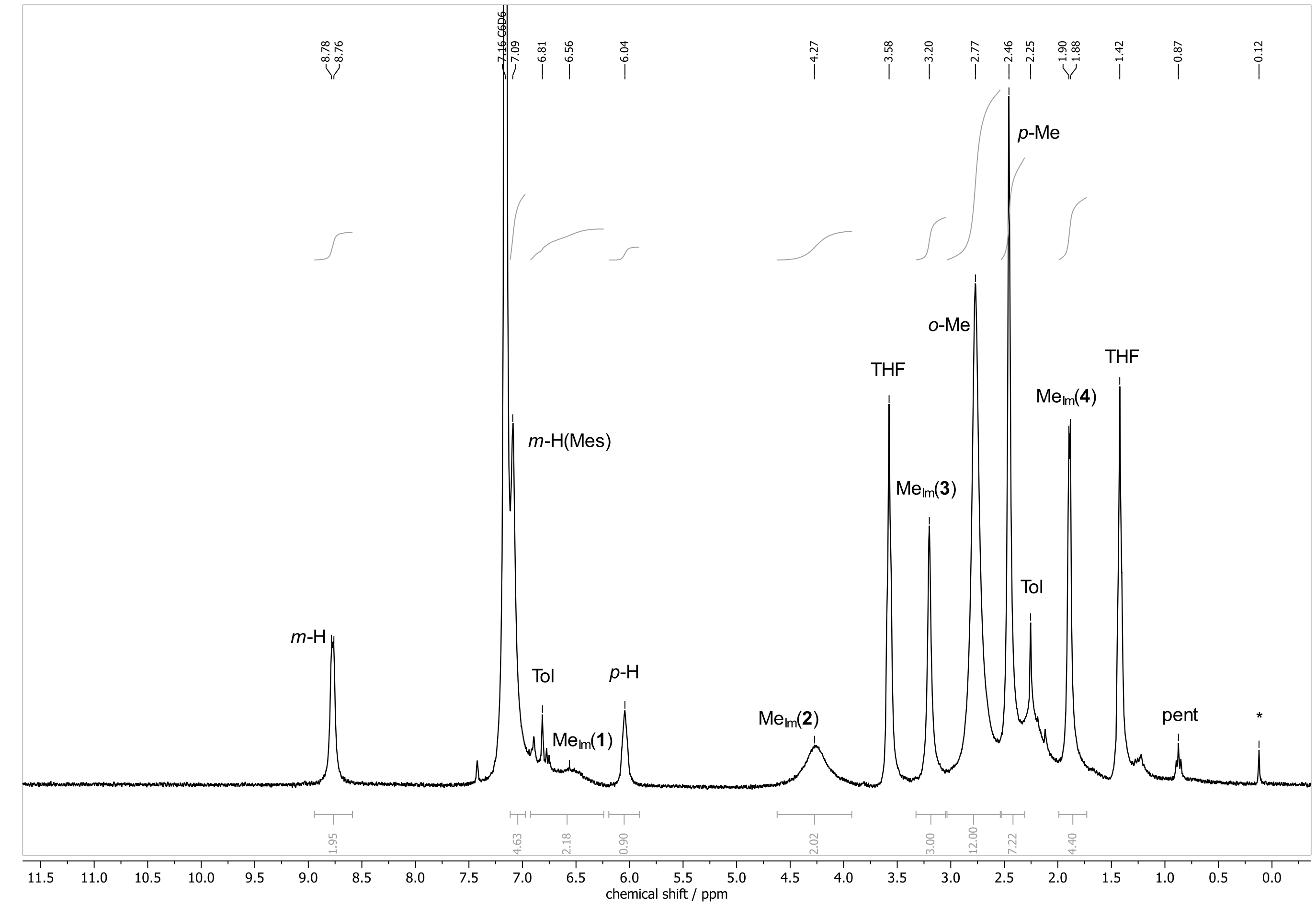

Supplementary Figure 43. 300 MHz ^{1}H NMR spectrum of $[Fe_4S_4(DmpS)_2(Im^*)_2]$ recorded at room temperature in C_6D_6.

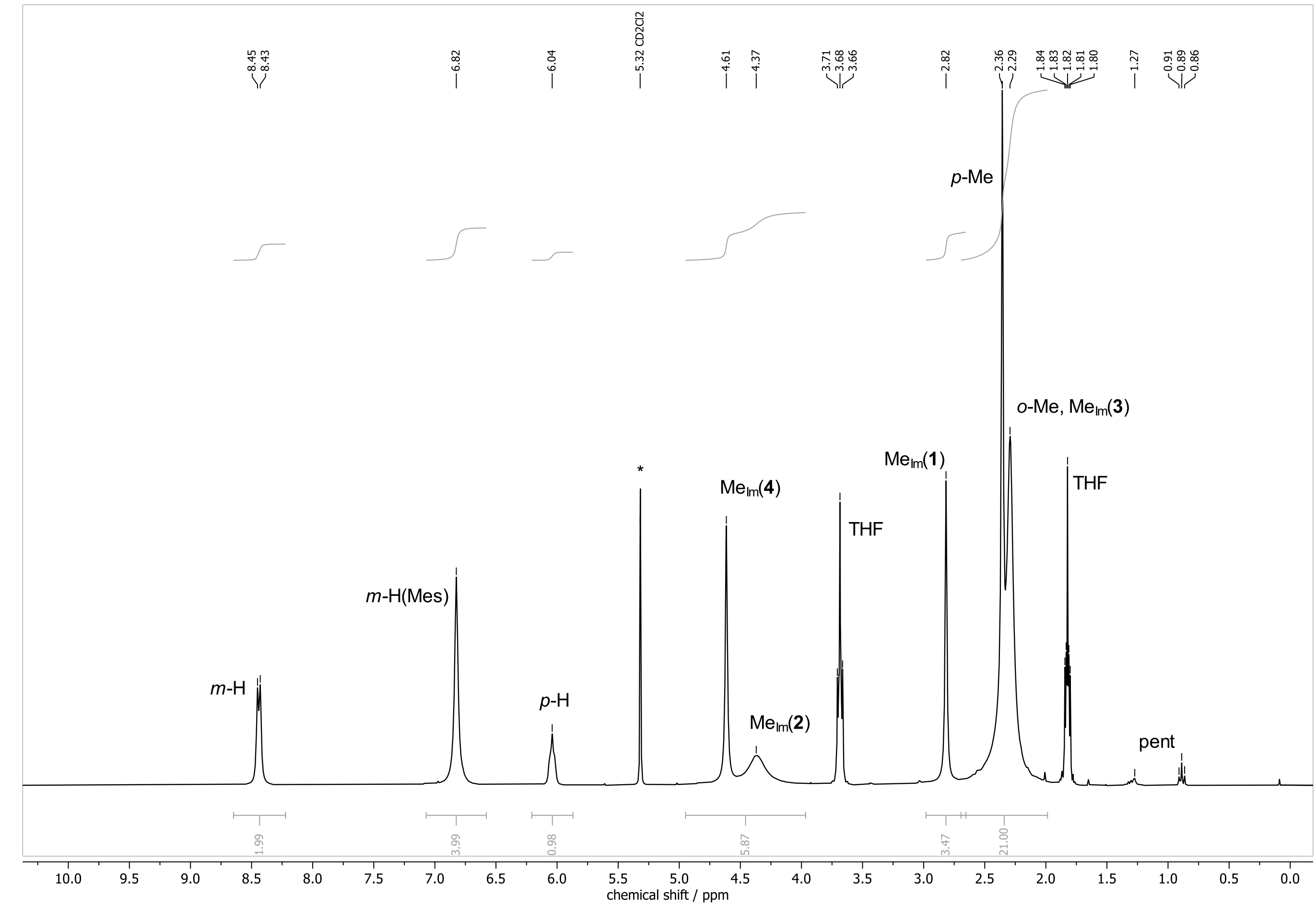

Supplementary Figure 44. 300 MHz ^{1}H NMR spectrum of $[Fe_4S_4(DmpS)_2(Im^*)_2]$ recorded at room temperature in CD_2Cl_2 (*).

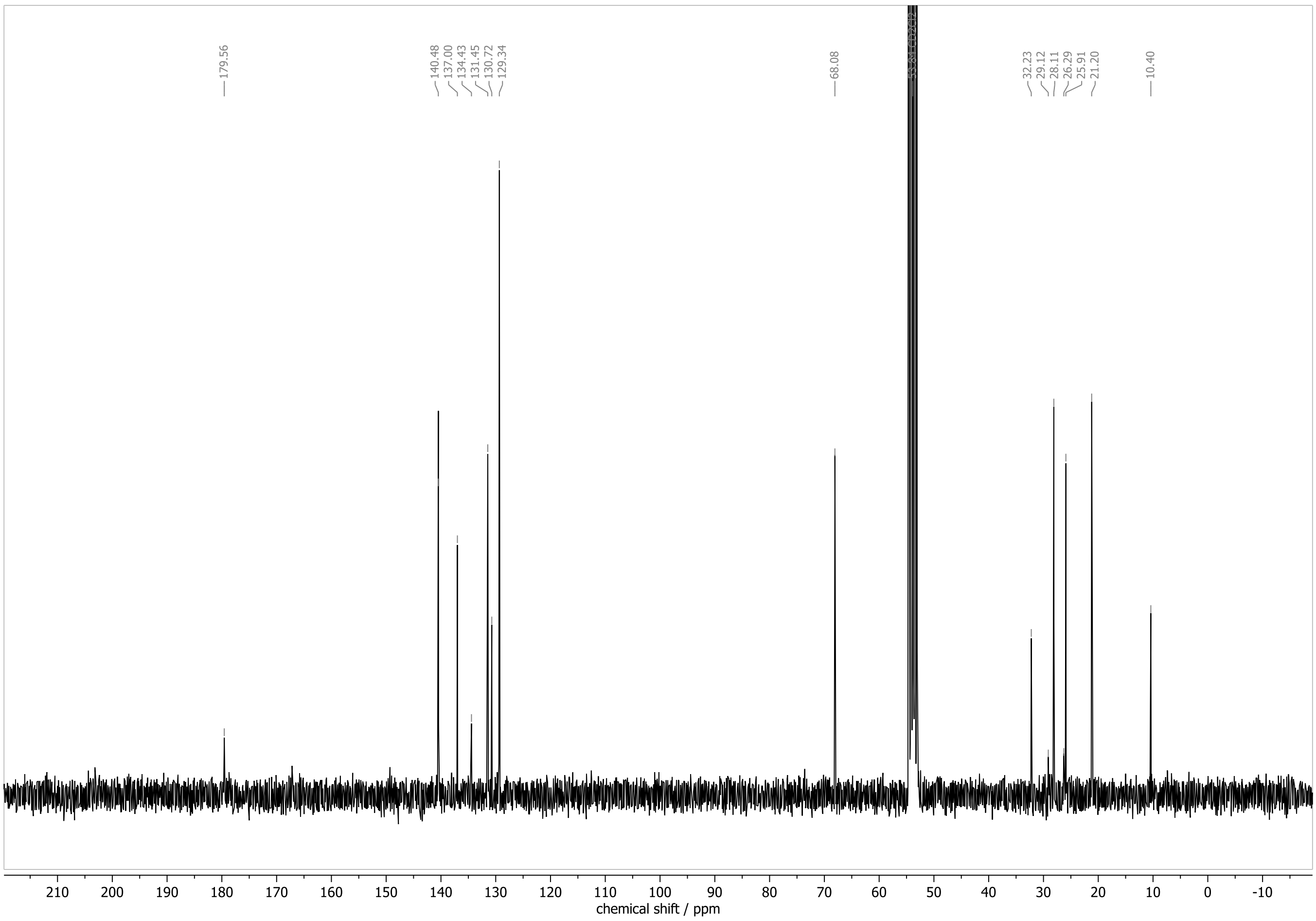

Supplementary Figure 45. 75 MHz ^{13}C NMR spectrum of [$Fe_4S_4(DmpS)_2(Im^*)_2$] recorded at room temperature in CD_2Cl_2 (*).

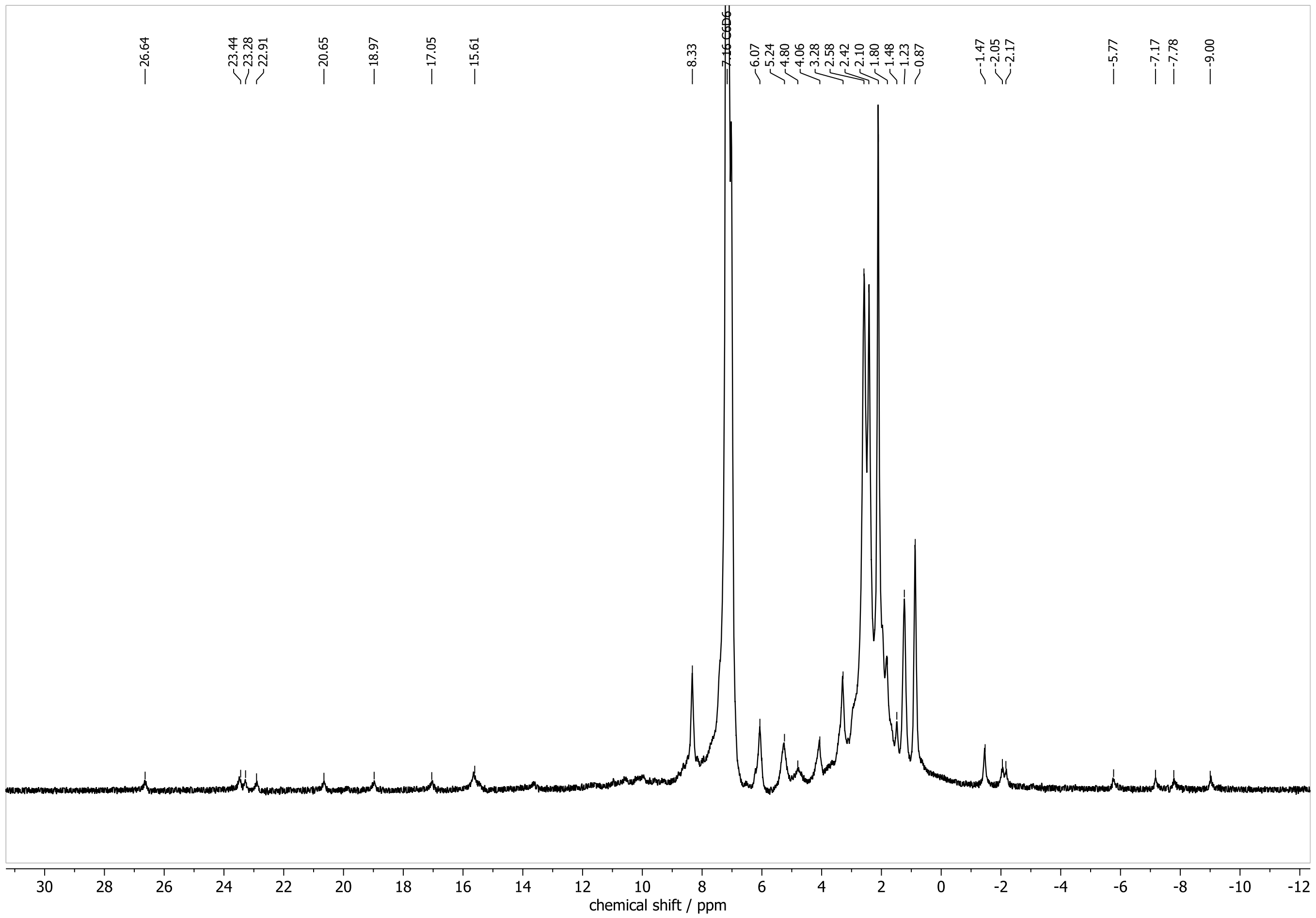

Supplementary Figure 46. 300 MHz ^{1}H NMR spectrum of $K_4[Fe_8S_8(DmpS)_6]$ recorded at room temperature in C_6D_6.

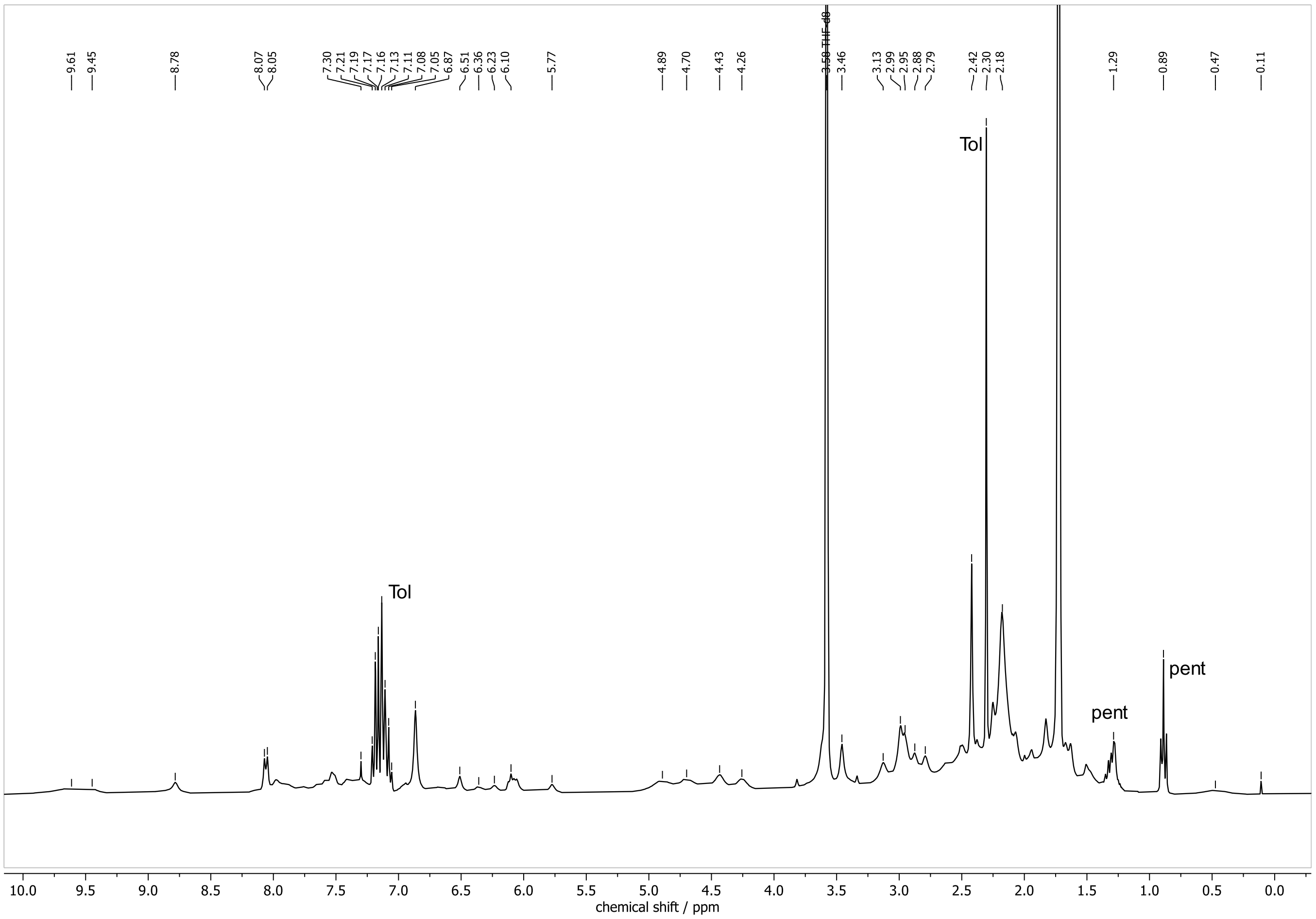

Supplementary Figure 47. Diamagnetic region of the 300 MHz ^{1}H NMR spectrum of $K_4[Fe_8S_8(DmpS)_6]$ recorded at room temperature in THF-*d*8.

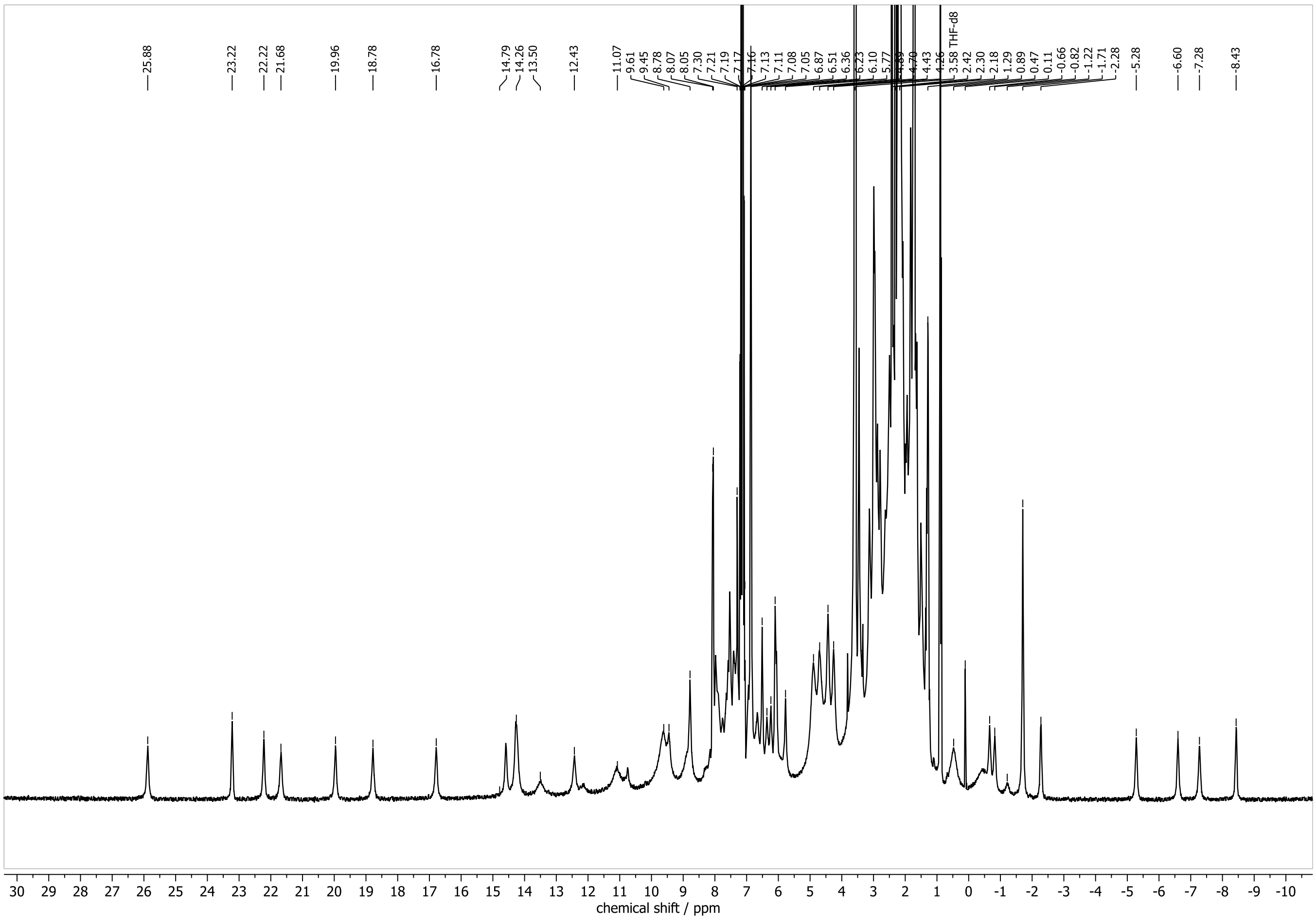

Supplementary Figure 48. Paramagnetic region of the 300 MHz ^{1}H NMR spectrum of $K_4[Fe_8S_8(DmpS)_6]$ recorded at room temperature in THF-*d*8.

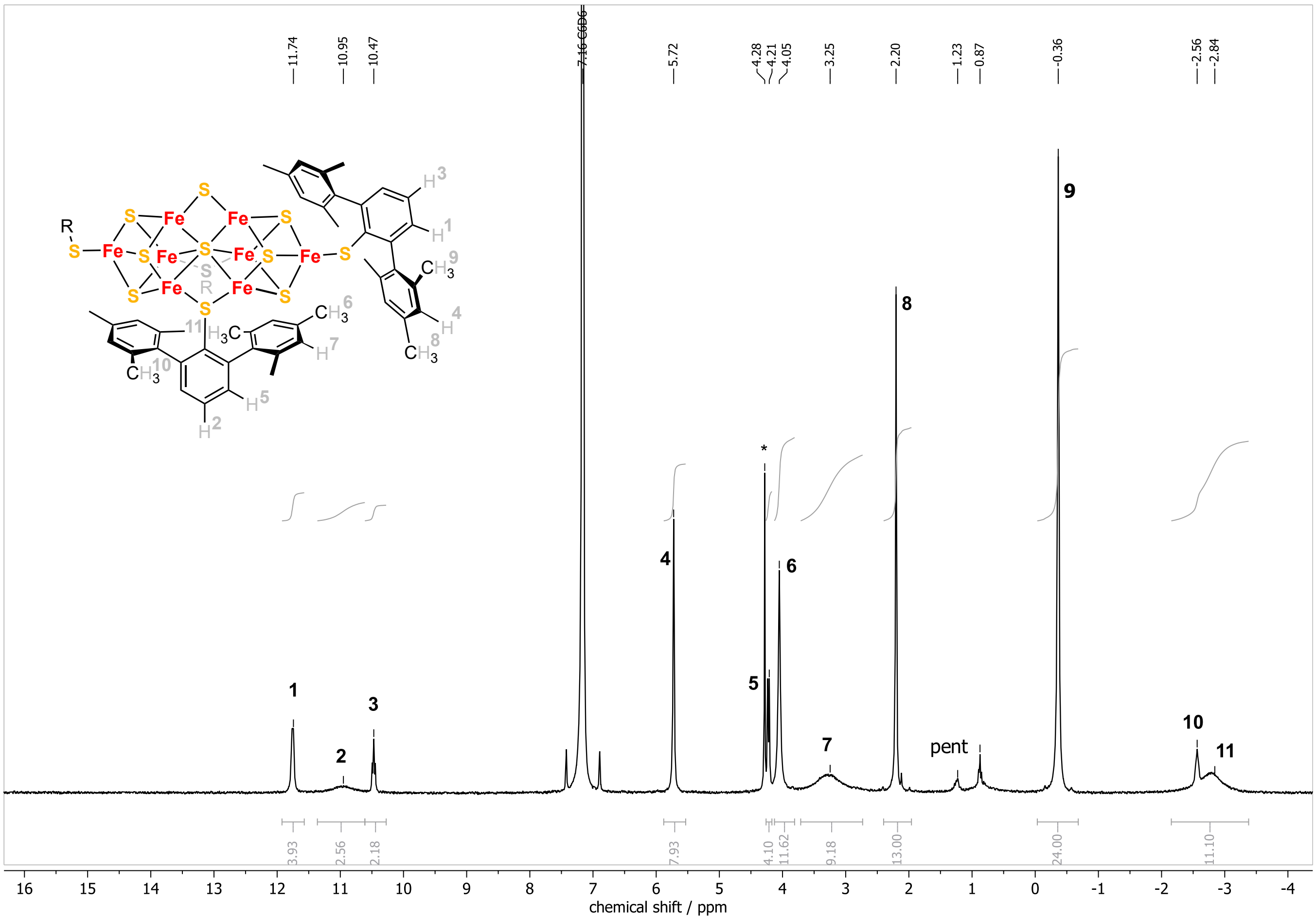

Supplementary Figure 49. 300 MHz ^1H NMR spectrum of [$Fe_8S_8(DmpS)_4$] recorded at room temperature in C_6D_6. An assignment of the peaks is proposed within the inset scheme. Some residual CH_2Cl_2 is marked by an asterisk (*).

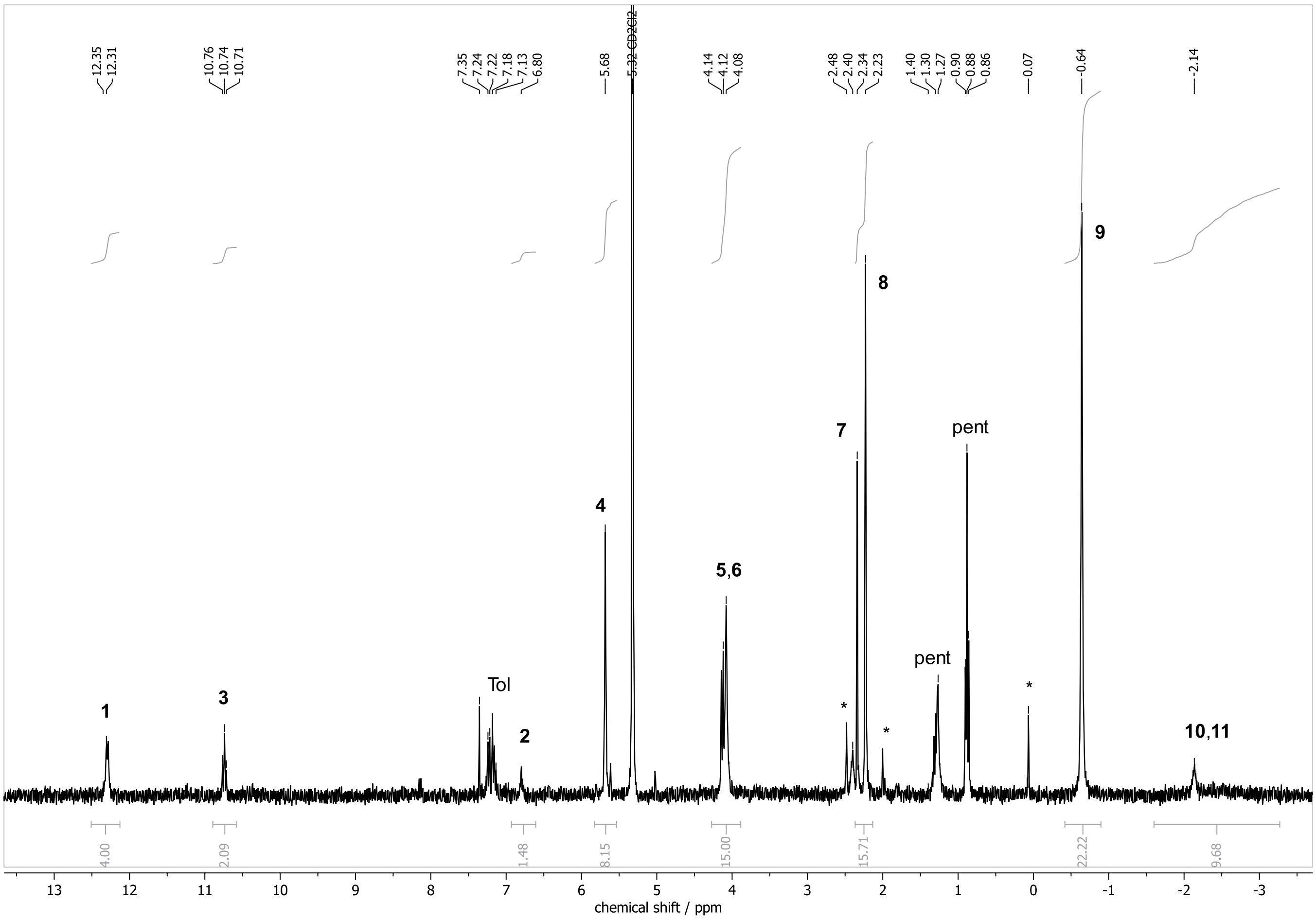

Supplementary Figure 50. 300 MHz ^{1}H NMR spectrum of $[Fe_8S_8(DmpS)_4]$ recorded at room temperature in CD_2Cl_2. Some unknown impurities are marked by an asterisk (*).

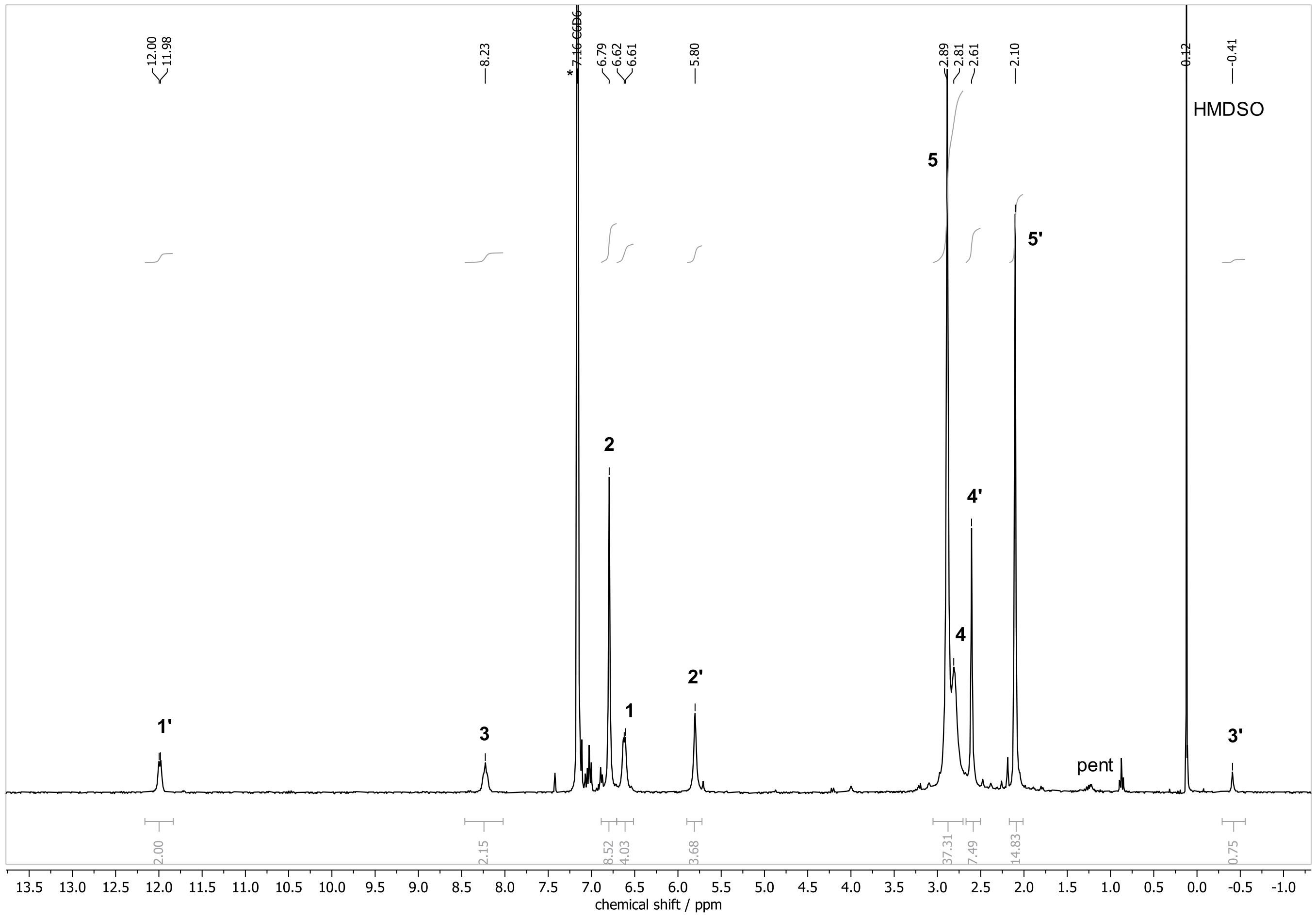

Supplementary Figure 51. 300 MHz ^{1}H NMR spectrum of $[Fe_8S_8(DmpS)_6]$ recorded at room temperature in C_6D_6.

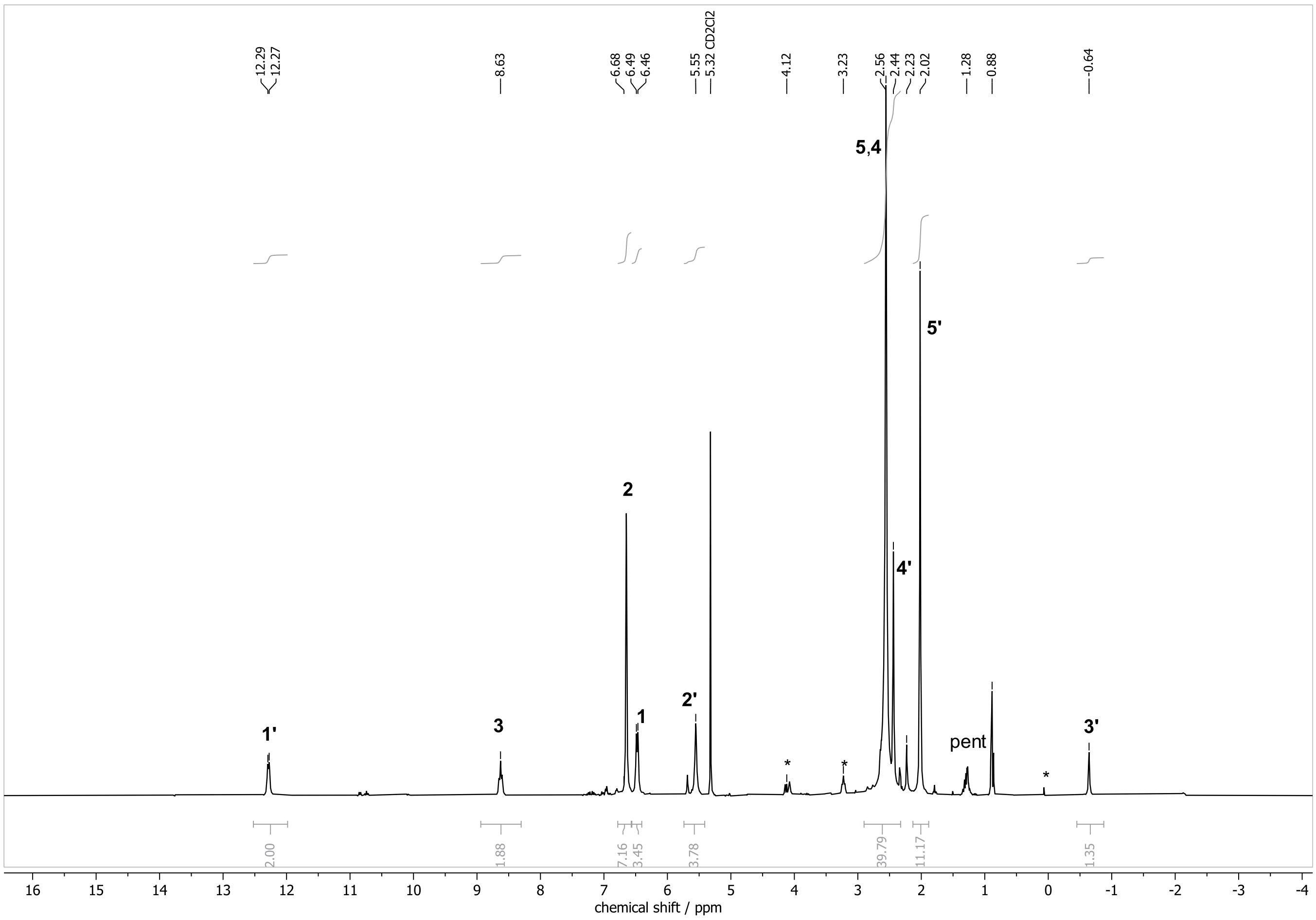

Supplementary Figure 52. 300 MHz ^{1}H NMR spectrum of $[Fe_8S_8(DmpS)_6]$ recorded at room temperature in CD_2Cl_2.

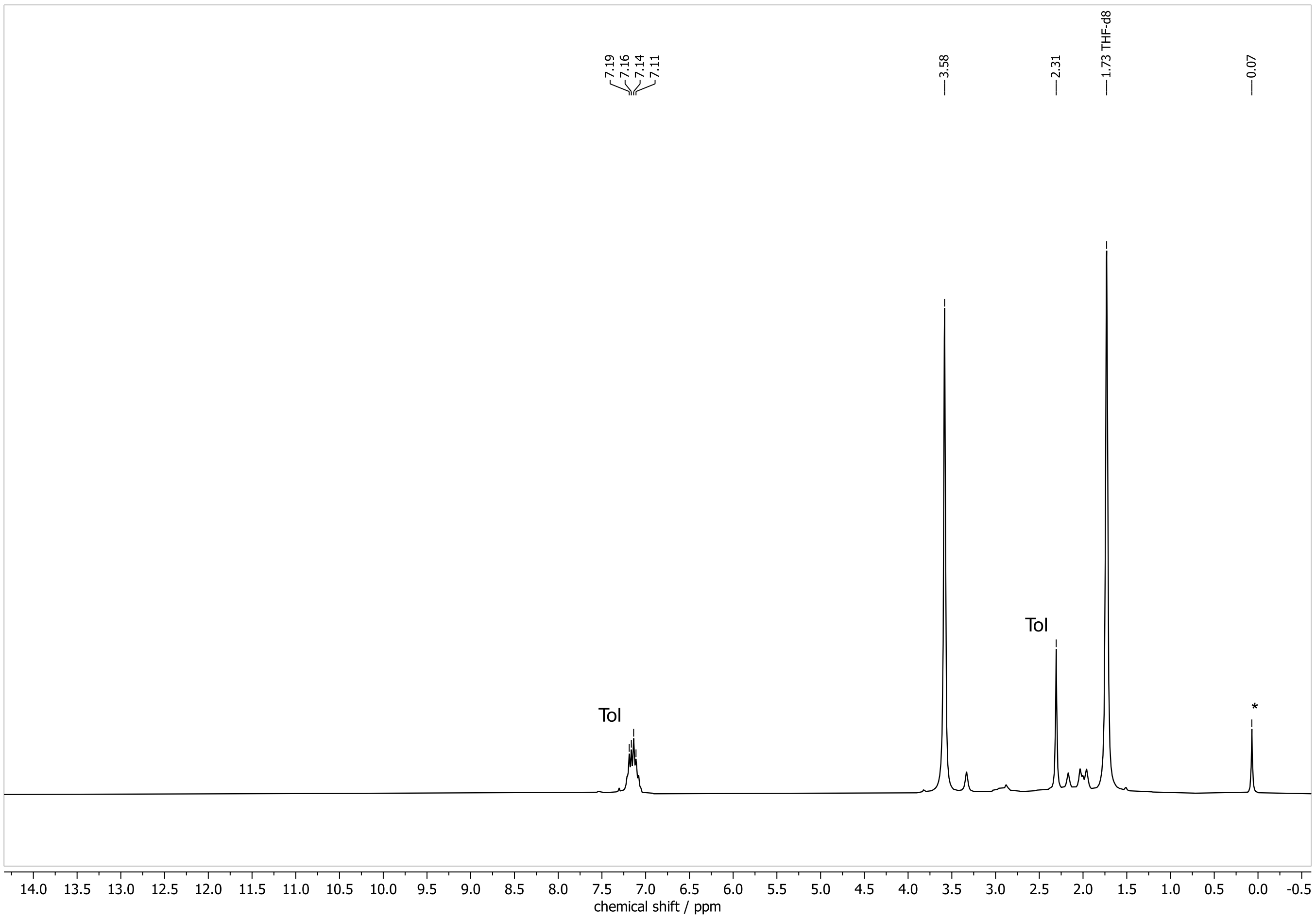

Supplementary Figure 53. 300 MHz ^1H NMR spectrum of $K_6[Fe_{12}S_{12}(DmpS)_6]$ recorded at room temperature in C_6D_6. The compound seems to be NMR silent.

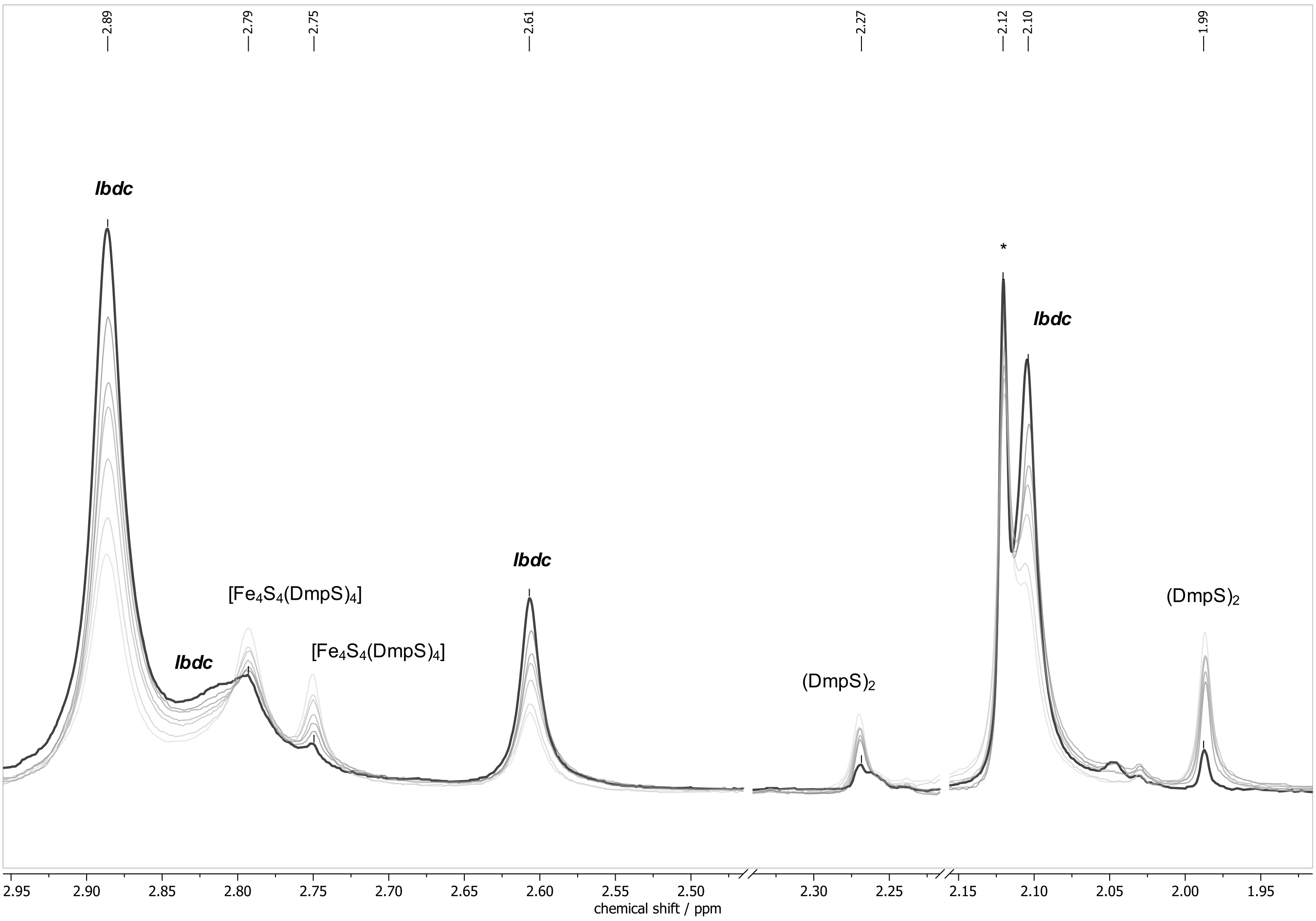

Supplementary Figure 54. Aliphatic region of the 300 MHz ^{1}H NMR spectra of ***lbdc*** recorded at room temperature in C_6D_6 over the course of 10 days (black to grey lines).

Supplementary Note 5. Supplementary Figs. 54 and 55 show the monitoring of a dilute (4 mg in 600 µL) benzene solution of ***lbdc*** *via* ^{1}H NMR over the course of 10 days. During this time, the slow formation of both ***ildc*** as well as [Fe_4S_4(DmpS)$_4$] could be observed (Supplementary Fig. 56). Logically, also the disproportionation of 2 ***lbdc*** to form 1 ***ildc*** and 2 all-ferric canonical cubanes would be a feasible mechanism for the formation of ***ildc*** and, likely, it occurs. However, this mechanism is not supported by the observation of the formation of (DmpS)$_2$ (Supplementary Figs. 54 and 25). Furthermore, if the disproportionation would indeed be the dominant mechanism, the equivalents of formed [Fe_4S_4(DmpS)$_4$] should supersede those of ***ildc***; This is however not the case here.

Therefore, we conclude that the reductive elimination of disulfide from ***lbdc*** is indeed most likely the dominant mechanism for formation of ***ildc***, even though it does compete to some degree with disproportionation, and perhaps even other mechanisms, which are not observable in ^{1}H NMR.

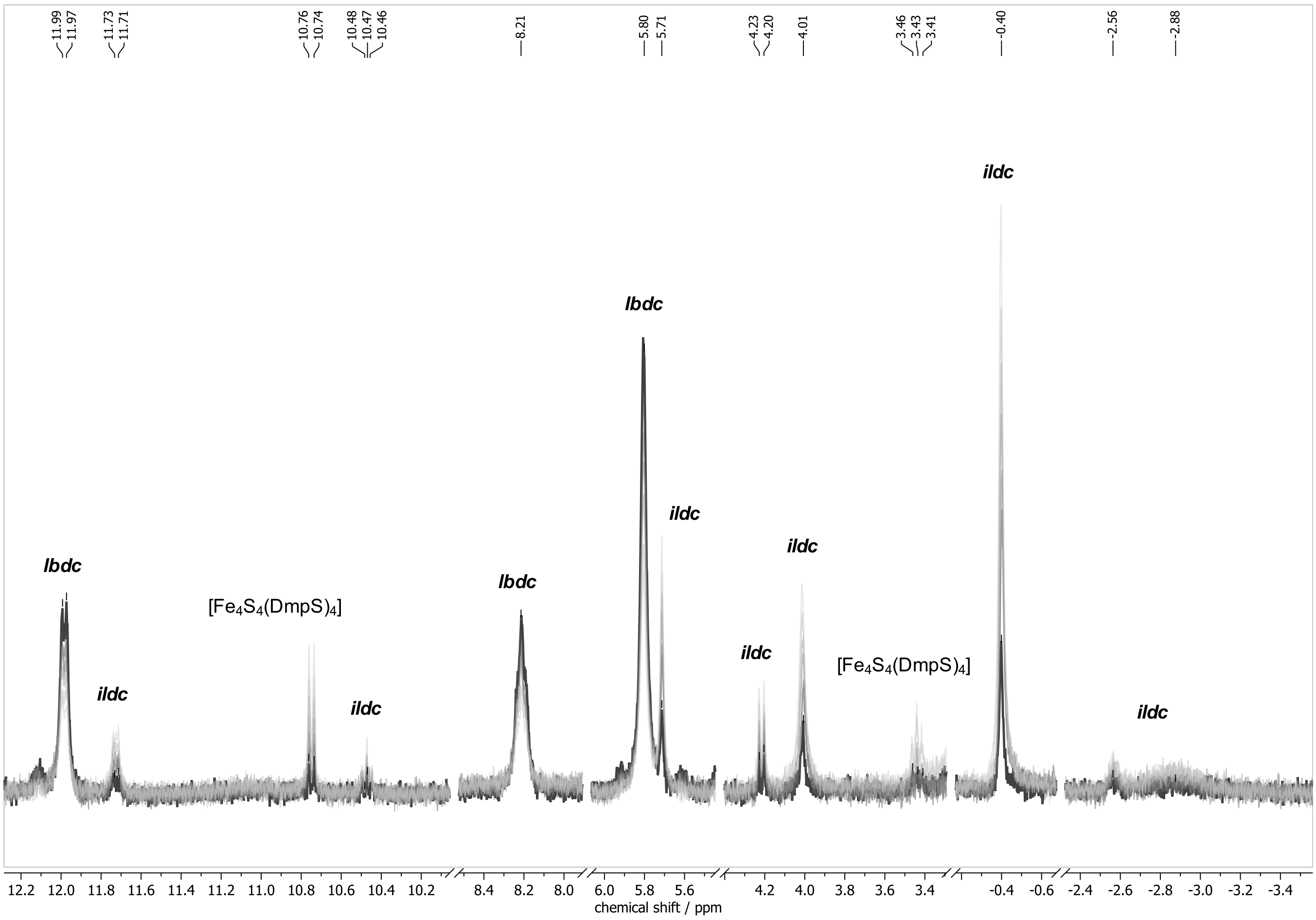

Supplementary Figure 55. Selection of peaks and their time evolution in the 300 MHz ^{1}H NMR spectra of ***lbdc*** recorded at room temperature in C_6D_6 over the course of 10 days (black to grey lines). Refer to Supplementary Note 5 for a short discussion statement.

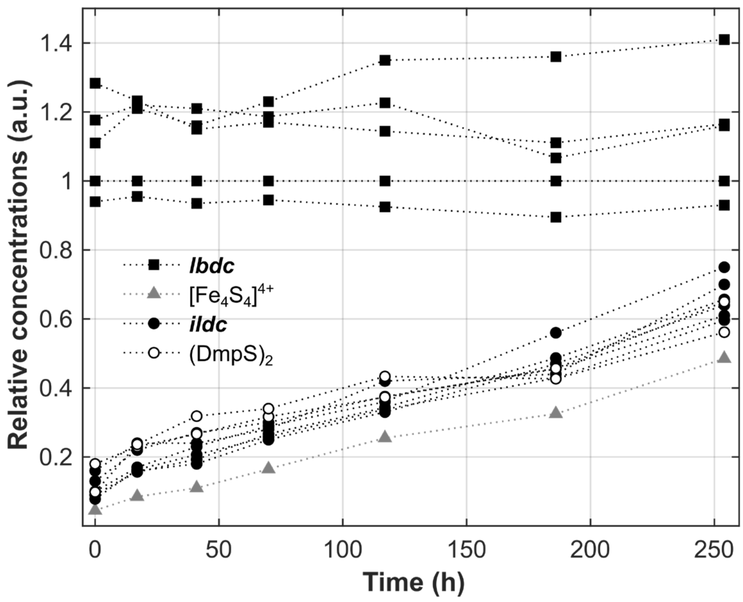

Supplementary Figure 56. Time-evolution of the intensities (concentrations) of selected peaks observable in the ^{1}H NMR spectra shown in Supplementary Figs. 54 and 55. One of the peaks of ***lbdc*** was normalized to 1, and all the other peaks are referenced to it.

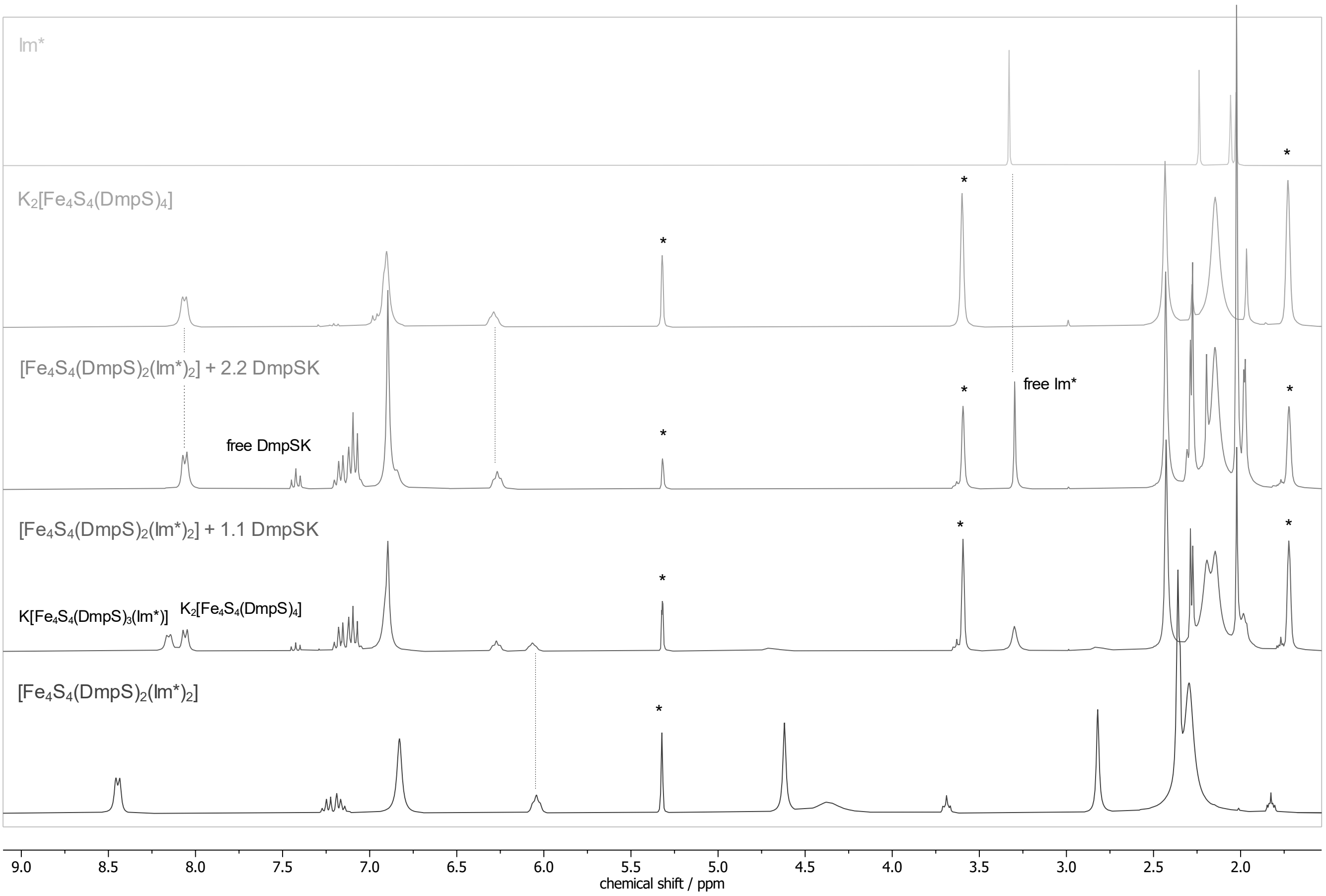

Supplementary Figure 57. Relevant NMR spectra for the conversion of $[Fe_4S_4(DmpS)_2(Im^*)_2]$ to $K_2[Fe_4S_4(DmpS)_4]$. All spectra were recorded at 300 MHz and at room temperature. Solvent residual signals of CD_2Cl_2 and THF-*d*8 are marked by an asterisk (*).

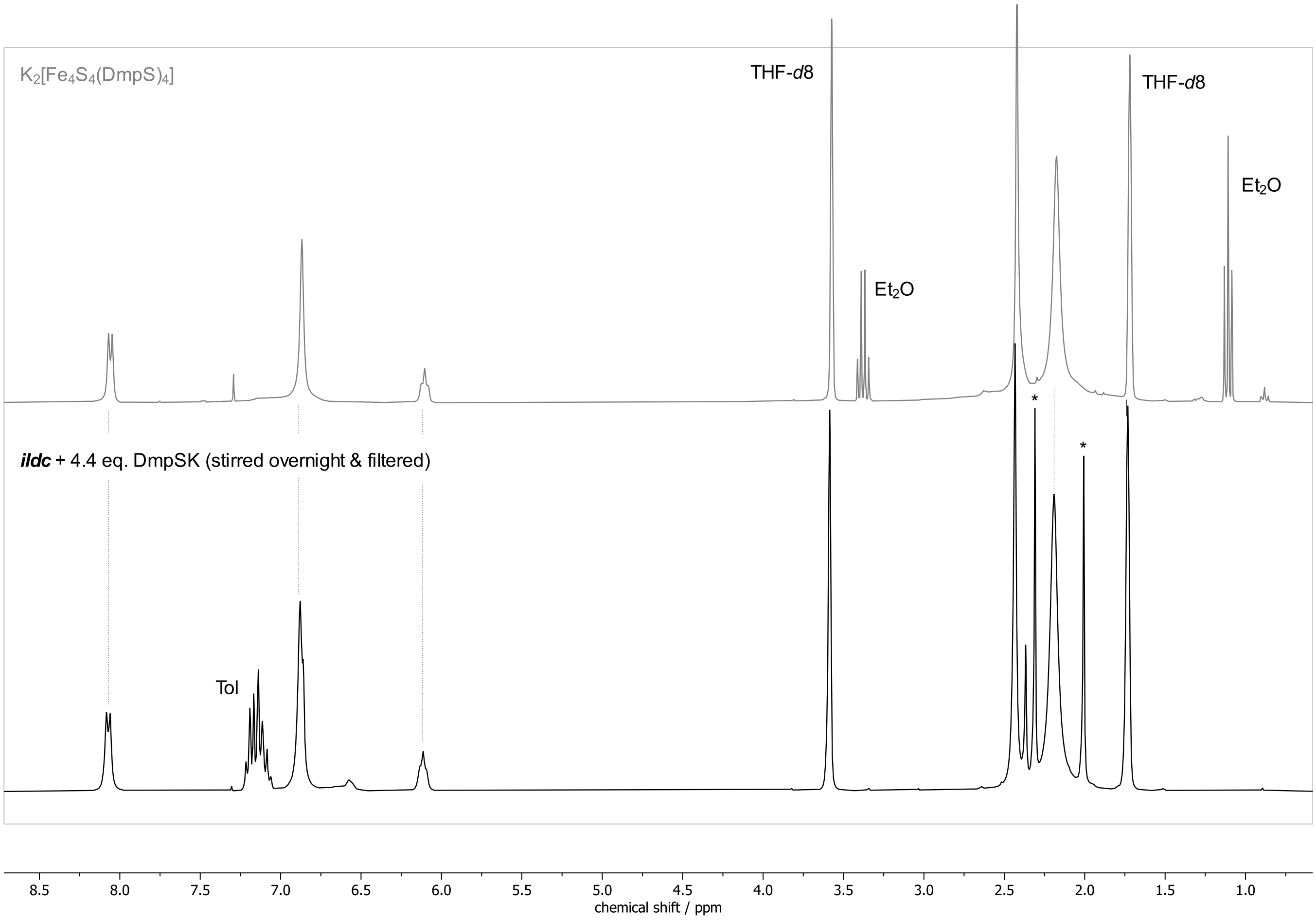

Supplementary Figure 58. Relevant NMR spectra for the conversion of ***ildc*** to $K_2[Fe_4S_4(DmpS)_4]$. All spectra were recorded at 300 MHz and at room temperature. Residual solvent (from the crystal lattice) was present in the samples and was annotated indicated accordingly. Signals corresponding to excess DmpSK are marked by an asterisk (*).

Cyclic Voltammetry

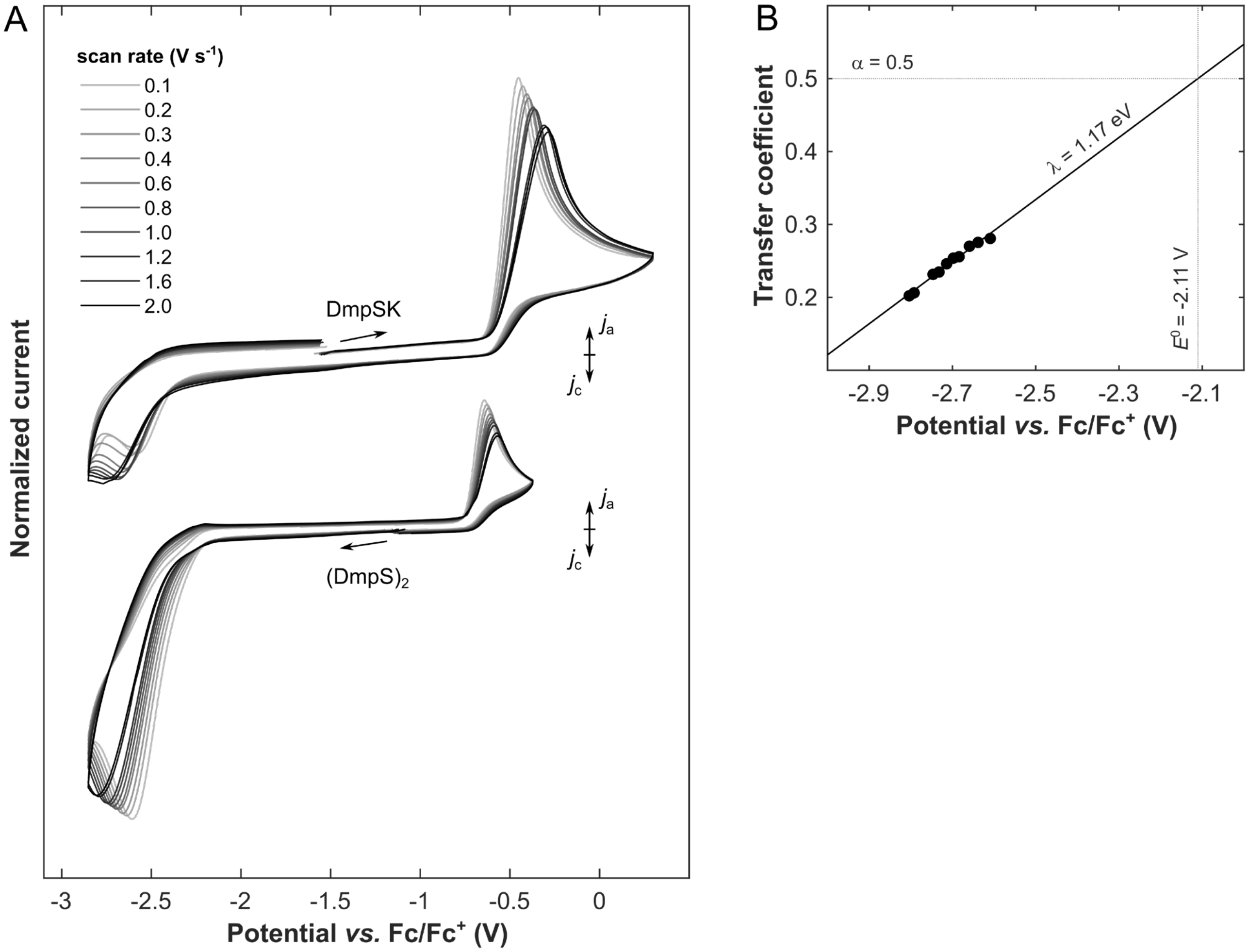

Supplementary Figure 59. (A) Cyclic voltammograms of 2mM solutions of DmpSK (top) and $(DmpS)_2$ (bottom) recorded in a 0.1 M $[^nBu_4N][PF_6]$ solution in THF as supporting electrolyte. Voltammograms recorded at varying scan rates are shown on shades of *grey* and a *black arrow* marks the starting point and direction of the first scan. The currents were normalized by dividing by the square-root of the frequency. (B) Reductive peak potentials of $(DmpS)_2$ at varying scan rates plotted versus the transfer coefficient *α*. *Dotted grey lines* mark the potential at which *α*=0.5, and thus, $E=E_{1/2}$. For further details, refer to Supplementary Note 6 (*vide infra*).

Supplementary Note 6. We determined the transfer coefficient at each scan rate by the equation summarized below, as reported by Nocera an co-workers in their recent publication:[33]

$$\alpha = \frac{1.857}{(E_{p/2} - E_p)} \frac{RT}{F} .$$

Here, $E_{p/2}$ refers to the potential at the mid-peak height, and E_p is the potential at the peak. The standard potential (E^0) is the potential at which $\alpha = 0.5$. Thus, E^0 of the thiolate is obtained from the extrapolation of the linear relationship between α and E_p to $\alpha = 0.5$, as shown in Supplementary Fig. 59B.

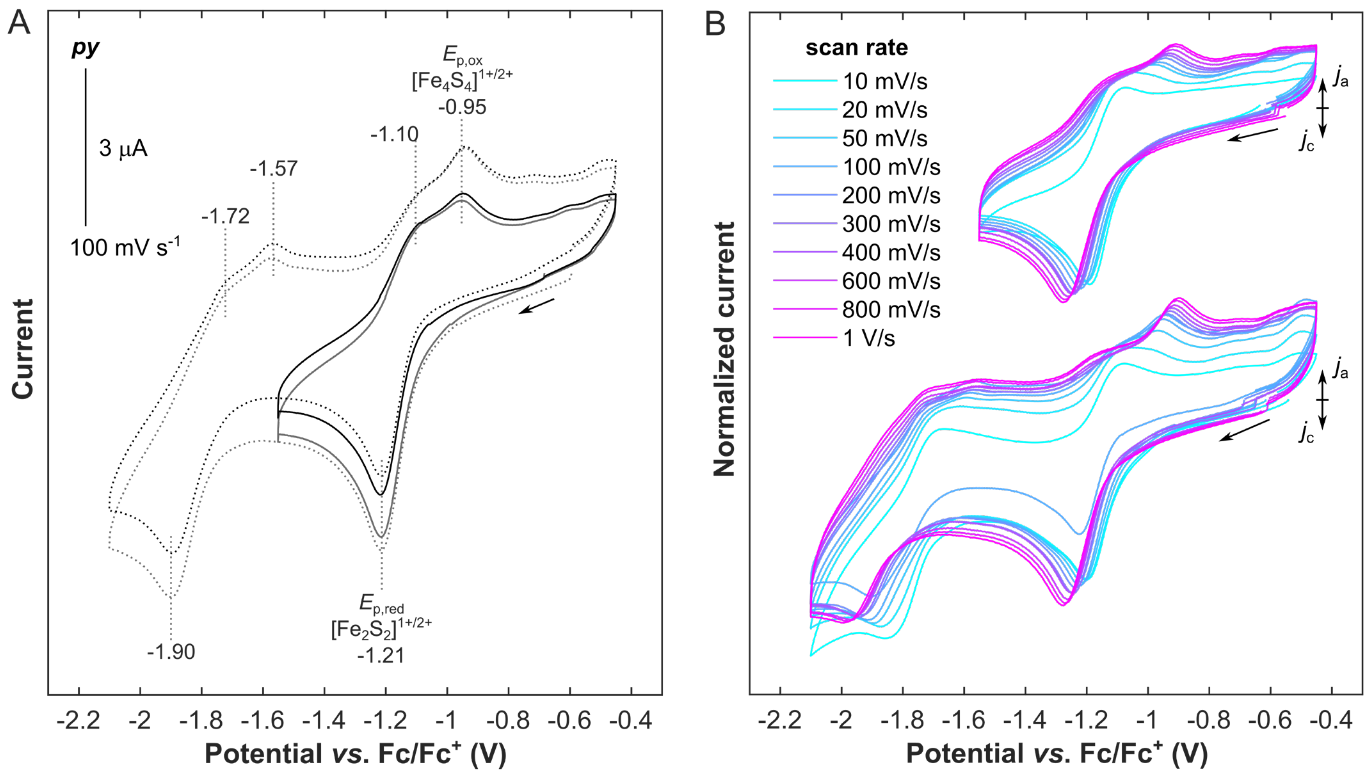

Supplementary Figure 60. (A) Cyclic voltammogam of a 2 mM solution of $[Fe_2S_2(DmpS)_2(py)_2]$ in 0.1 M $[^nBu_4N][PF_6]$ in 1,2-difluorobenzene. Subsequent scans are shown in shades of *grey* and a *black arrow* marks the starting point and direction of the first scan. *Dotted lines* show scans over a wider potential range. (B) Scan rate dependence of the voltammogram, recorded over two potential ranges. A *black arrow* marks the starting point of the scans. For each scan rate, only the first scan is shown.

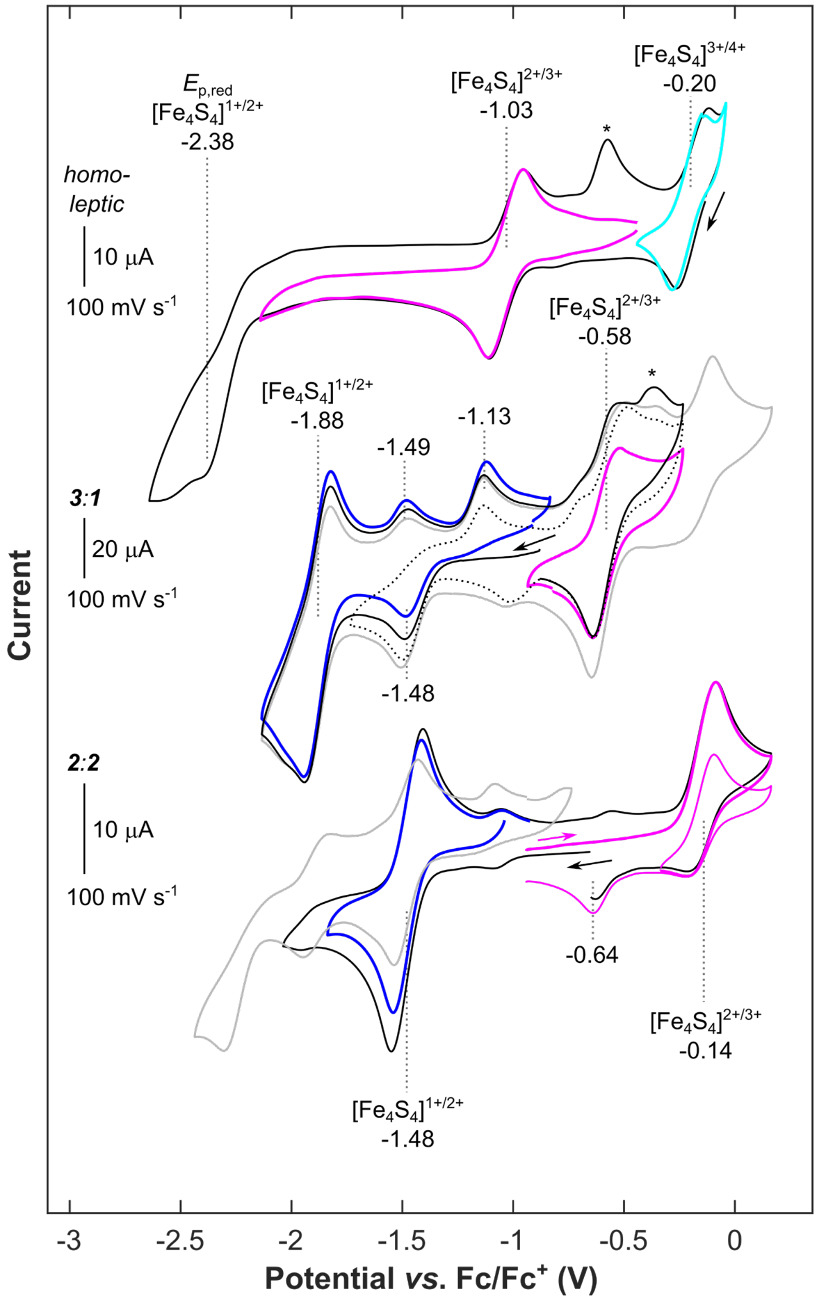

Supplementary Figure 61. Cyclic voltammograms of 2mM solutions of $[Fe_4S_4(DmpS)_4]$ (*top*), $K[Fe_4S_4(DmpS)_3(Im^*)]$ (*middle*) and $[Fe_4S_4(DmpS)_2(Im^*)_2]$ (*bottom*) recorded in a 0.1 M solution of $[^{n}Bu_4N][PF_6]$ in 1,2-diflourobenzene with a frequency of 100 mV s^{-1}. Subsequent scans and scans over different potential ranges are shown in shades of *grey* colour or as *dotted* lines. The starting points of the voltammograms are marked by arrows, and all redox potentials are given *vs*. the ferrocene/ferrocenium redox couple (Fc/Fc$^+$). The main redox events for all compounds are marked as coloured traces based on the redox couple: $[Fe_4S_4]^{3+/4+}$, *cyan*; $[Fe_4S_4]^{2+/3+}$, *magenta*; $[Fe_4S_4]^{1+/2+}$, *blue*.

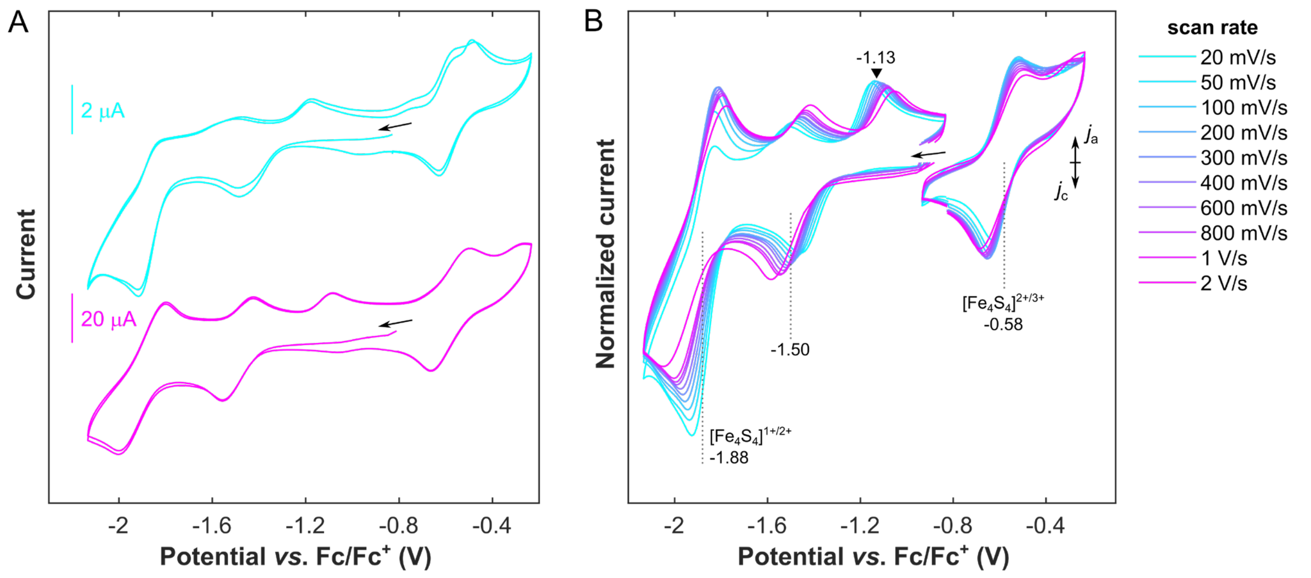

Supplementary Figure 62. (A) Cyclic voltammograms of K[Fe_4S_4(DmpS)$_3$(Im*)] (2 mM in 1,2-difluorobenzene) recorded in 0.1 M [^{n}Bu$_4$N][PF_6] solution in 1,2-difluorobenzene. *Cyan*: Cyclic voltammogram recorded with a scan rate of 20 mV s^{-1}. *Magenta*: Cyclic voltammogram recorded with a scan rate of 2 V s^{-1}. Two subsequent scans are shown and a *black arrow* marks the starting point of the first scan. (B) Scan rate dependance of the oxidative and reductive redox events in the cyclic voltammogram of K[Fe_4S_4(DmpS)$_3$(Im*)]. The current has been normalized by dividing through the square-root of the scan rate.

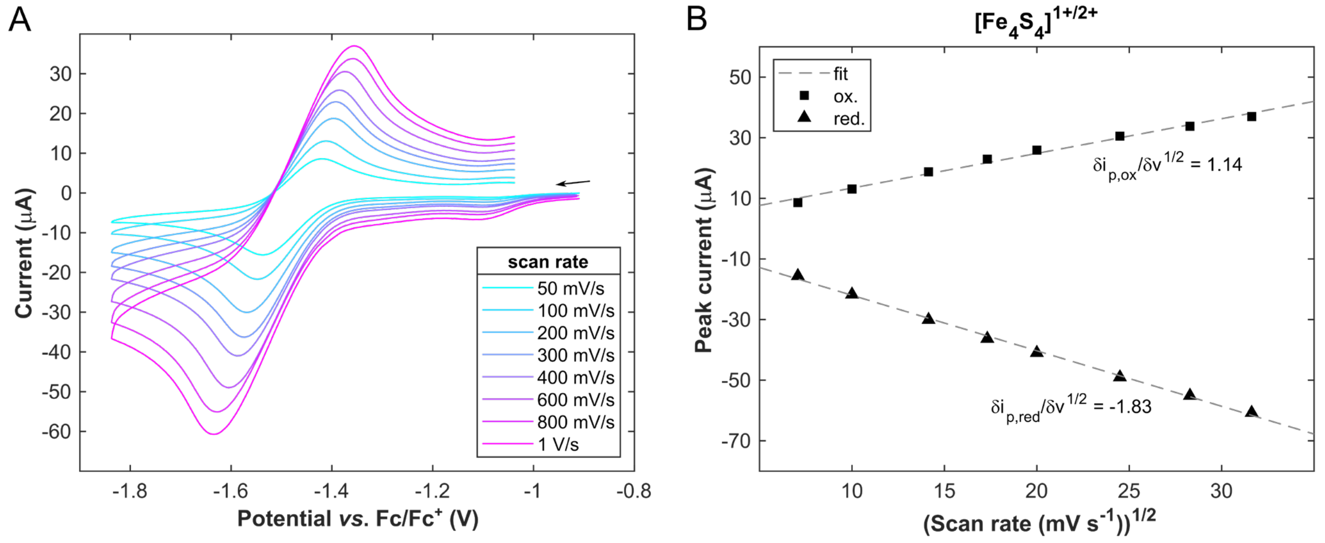

Supplementary Figure 63. (A) Cyclic voltammogram of the [Fe_4S_4(DmpS)$_2$(Im*)$_2$]$^{0/1-}$ redox couple recorded at varying scan rates. A *black arrow* marks the starting point of each scan. (B) Peak current, I_p, plotted against the square root of the scan rate, v, for the voltammograms shown in (A).

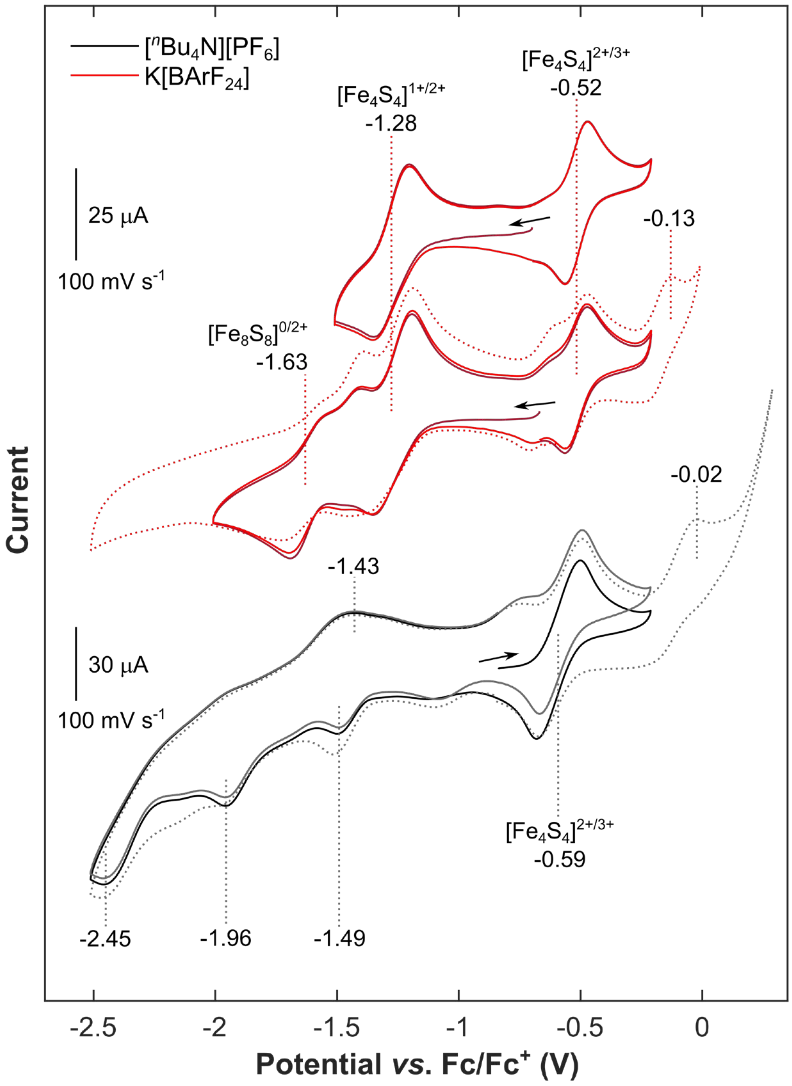

Supplementary Figure 64. Cyclic voltammograms of K[Fe_4S_4(DmpS)$_3$(Im*)] (2 mM in THF) recorded in [^{n}Bu$_4$N][PF$_6$] (*black*) and K[BArF$_{24}$] (*red*) electrolytes (0.1 M in THF), respectively. *Black arrows* mark the direction and starting points of the first scan. Subsequent scans are shown in shades of grey/red, respectively, and *dotted lines* are scans over a wider potential range.

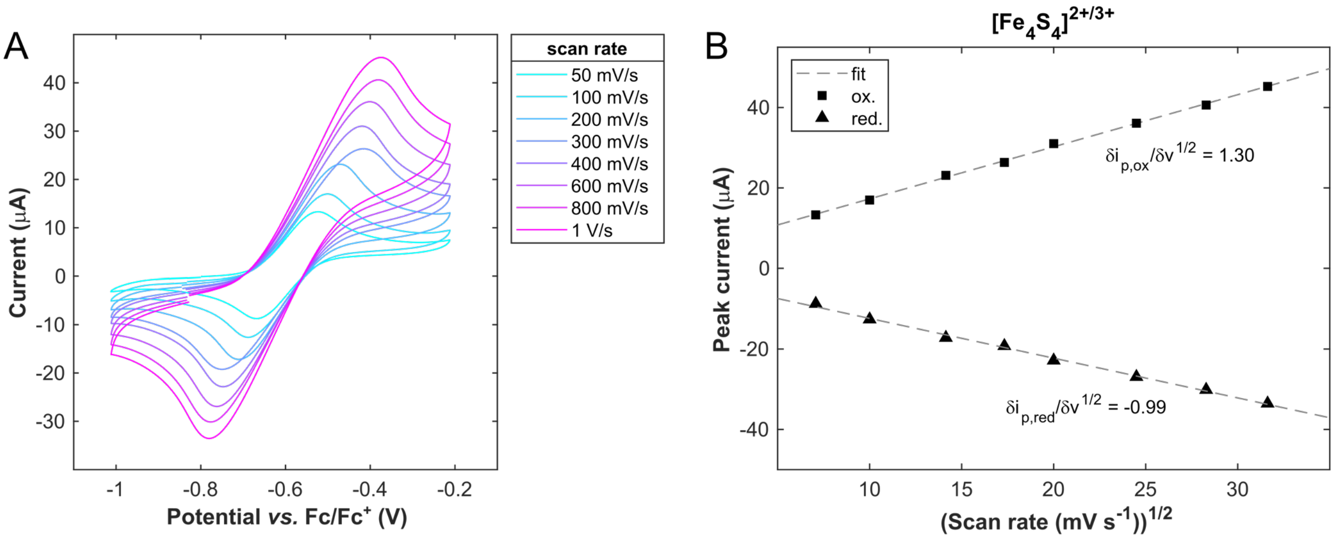

Supplementary Figure 65. (A) Cyclic voltammogram of the $[Fe_4S_4(DmpS)_3(Im^*)]^{0/1-}$ redox couple recorded at varying scan rates in 0.1 M $[^nBu_4N][PF_6]$ solution in THF. A *black arrow* marks the starting point of each scan. (B) Peak current, I_p, plotted against the square root of the scan rate, v, for the voltammograms shown in (A).

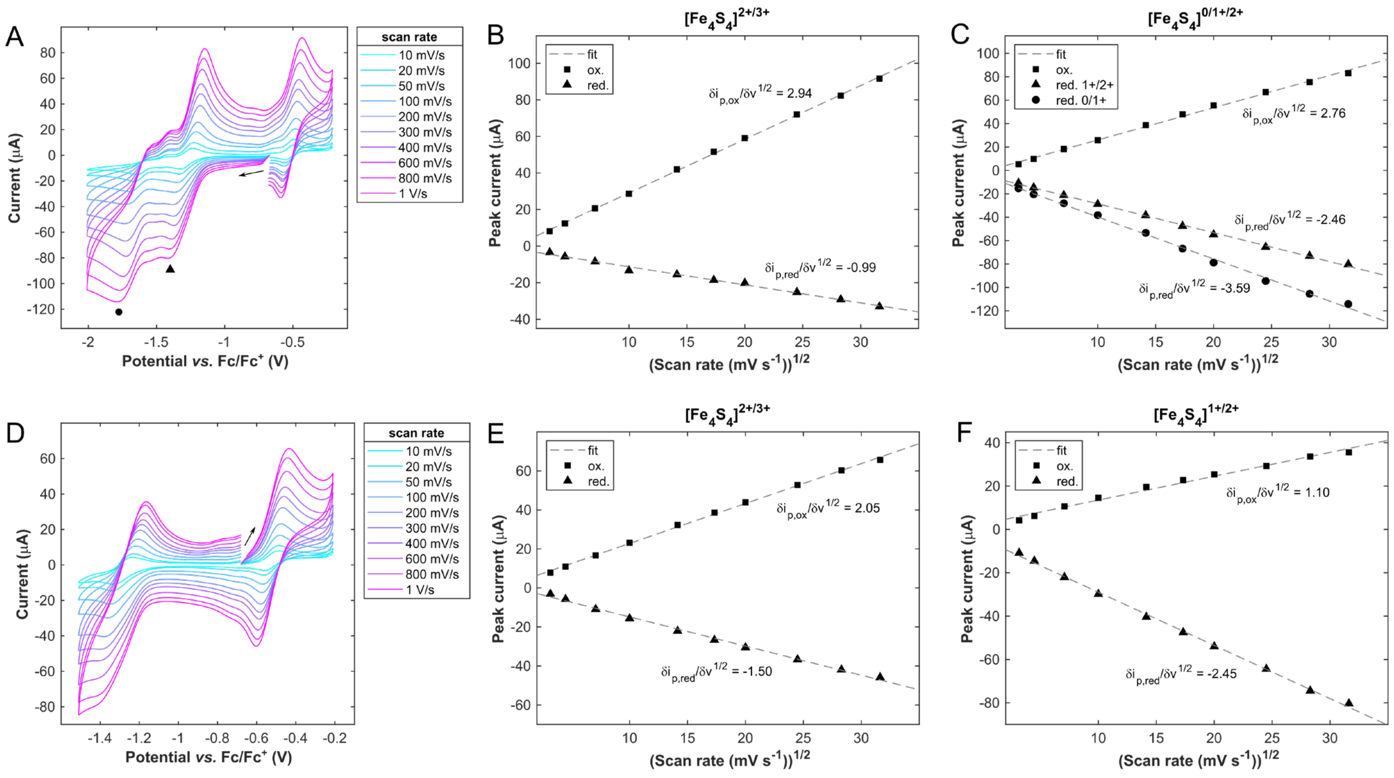

Supplementary Figure 66. (A) Scan rate dependance of the cyclic voltammogram of $[Fe_4S_4(DmpS)_3(Im^*)]$ recorded in 0.1 M $K[BArF_{24}]$ solution in THF. A *black arrow* marks the starting point of each scan. (B,C) Peak current, I_p, plotted against the square root of the scan rate, v, for the redox events in the voltammogram shown in (A). (D) Scan rate dependance of the cyclic voltammogram of $[Fe_4S_4(DmpS)_3(Im^*)]$ recorded in 0.1 M $K[BArF_{24}]$ solution in THF over a narrower potential range. A *black arrow* marks the starting point of each scan. (E,F) Peak current, I_p, plotted against the square root of the scan rate, v, for the two redox events in the voltammogram shown in (D)

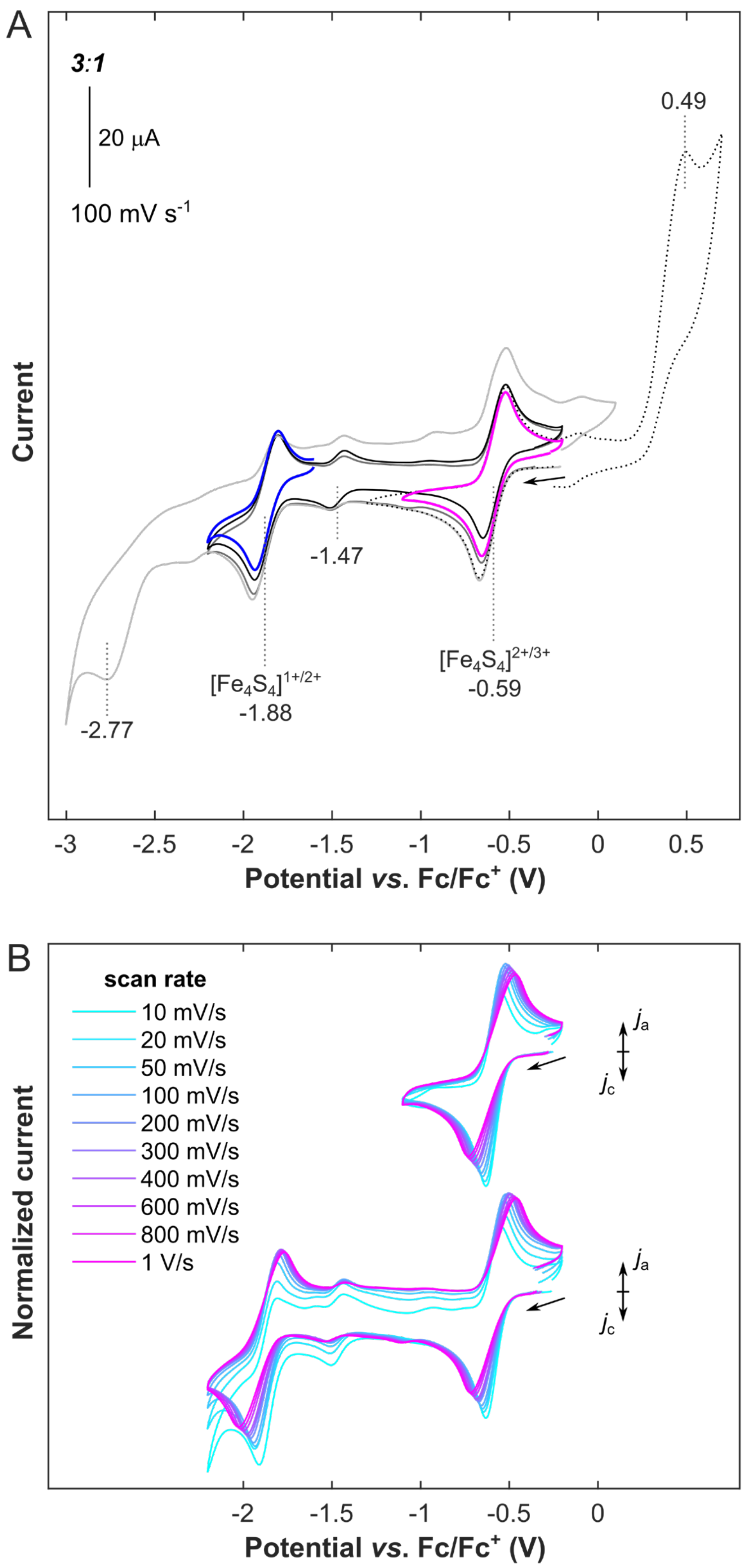

Supplementary Figure 67. (A) Cyclic voltammogram of a 2 mM *o*-DFB solution of $[Fe_4S_4(DmpS)_3(Im^*)]$ recorded in 0.1 M $[^nBu_4N][PF_6]$ as supporting electrolyte salt. Subsequent scans are shown in shades of *grey* and a *black arrow* marks the starting point and direction for all scans. Scans over a wider potential range are shown as *light grey* and *dotted lines*, respectively. The two redox events associated with the $[Fe_4S_4]^{1+/2+}$ and $[Fe_4S_4]^{2+/3+}$ redox couples are highlighted separately in *blue* and *magenta*, respectively. (B) Scan rate dependence of the voltammogram shown in panel (A).

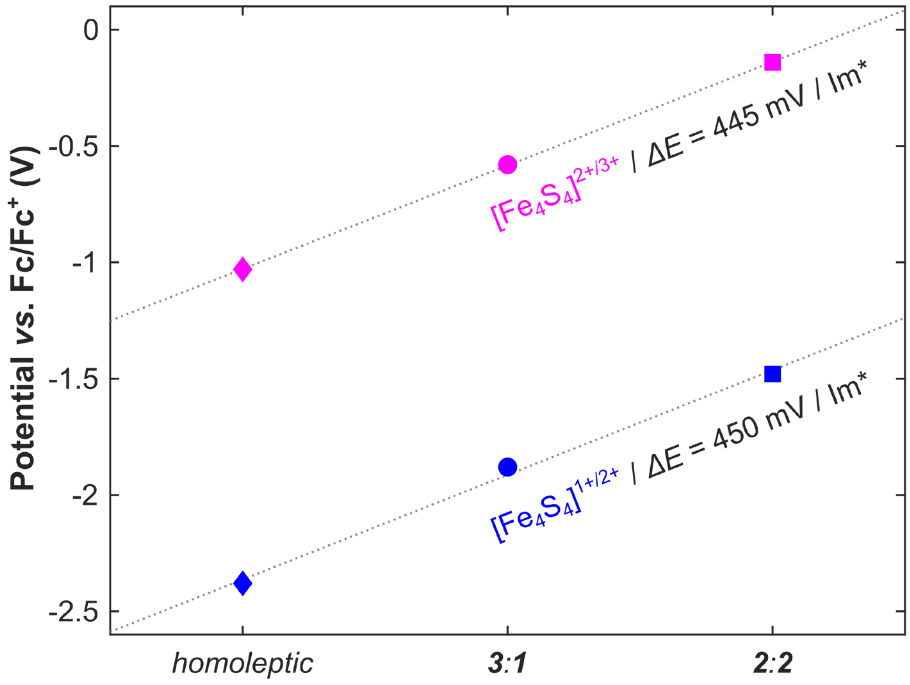

Supplementary Figure 68. Correlation of the number of Im* ligands on the Fe_4S_4 cubane with the complex's redox potential in the $[Fe_4S_4]^{1+/2+}$ (*blue*) and $[Fe_4S_4]^{2+/3+}$ (*magenta*) oxidation states *vs*. Fc/Fc^+: $[Fe_4S_4(DmpS)_4]$ (*homoleptic*; 0 Im*; *diamonds*), $[Fe_4S_4(DmpS)_3(Im^*)]$ (***3:1***; 1 Im*; *dots*) and $[Fe_4S_4(DmpS)_2(Im^*)_2]$ (***2:2***; 2 Im*; *squares*).

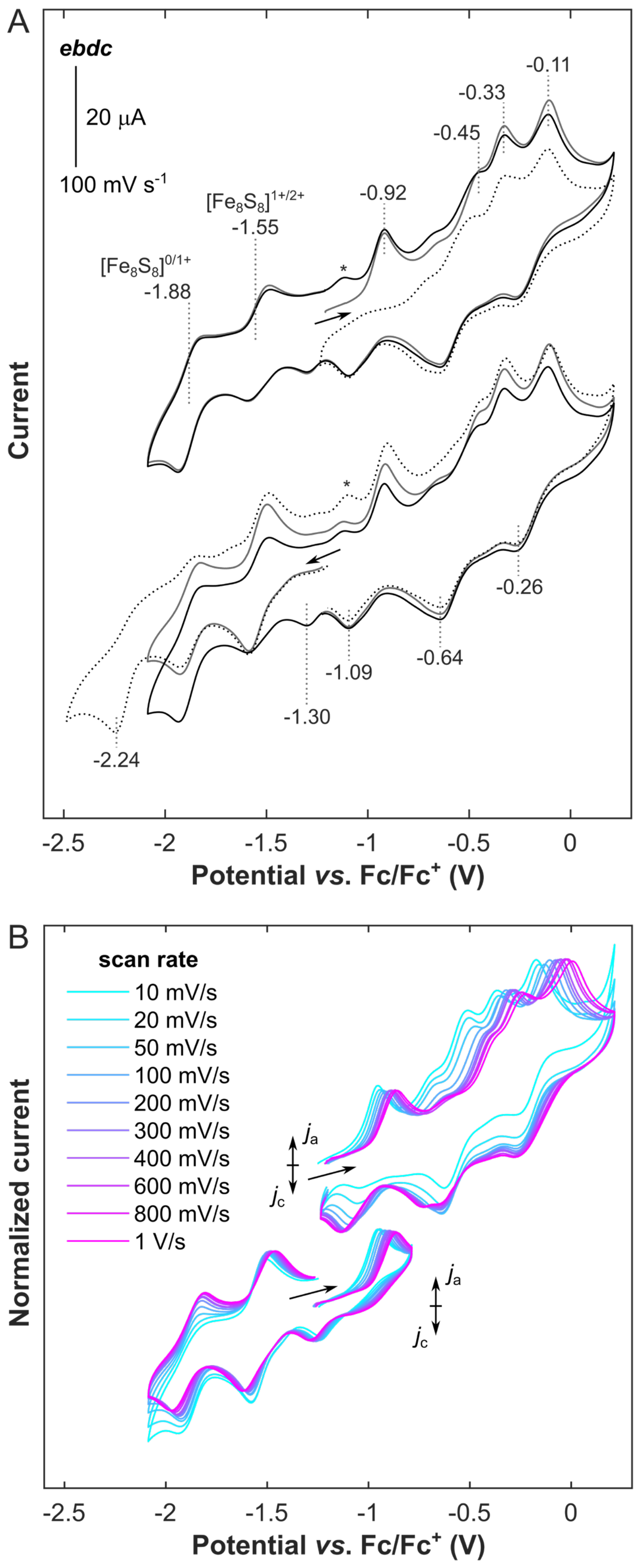

Supplementary Figure 69. (A) Cyclic voltammogram of a 2 mM *o*-DFB solution of $K_4[Fe_8S_8(DmpS)_6]$ (***ebdc***) recorded in 0.1 M $[^nBu_4N][PF_6]$ as supporting electrolyte salt. Subsequent scans are shown in shades of *grey* and a *black arrow* marks the starting point and direction for the first scan. The top traces show the voltammogram, if started scanning anodically, and the bottom traces if started scanning cathodically. Dotted lines show the second scans over narrower/wider potential ranges, respectively. The feature marked by an asterisk (*) only appears after scanning cathodically. (B) Scan rate dependence of the voltammogram shown in panel (A), for the anodic (top) and cathodic (bottom) currents, respectively.

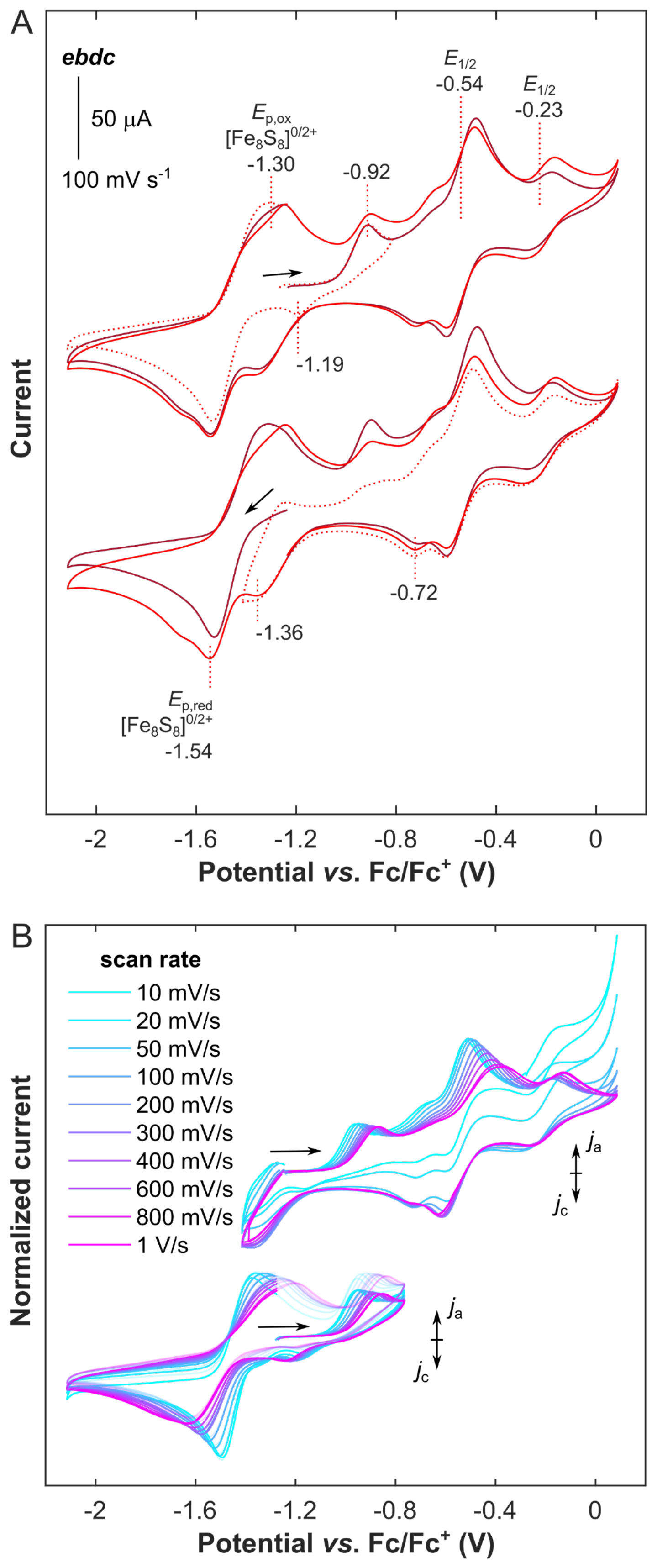

Supplementary Figure 70. (A) Cyclic voltammogram of a 2 mM THF solution of $K_4[Fe_8S_8(DmpS)_6]$ (***ebdc***) recorded in 0.1 M $K[BArF_{24}]$ as supporting electrolyte salt. Subsequent scans are shown in shades of *red* and a *black arrow* marks the starting point and direction for the first scan. The top traces show the voltammogram, if started scanning anodically, and the bottom traces if started scanning cathodically. Dotted lines show the second scans over narrower/wider potential ranges, respectively. (B) Scan rate dependence of the voltammogram shown in panel (A), for the anodic (top) and cathodic (bottom) currents, respectively.

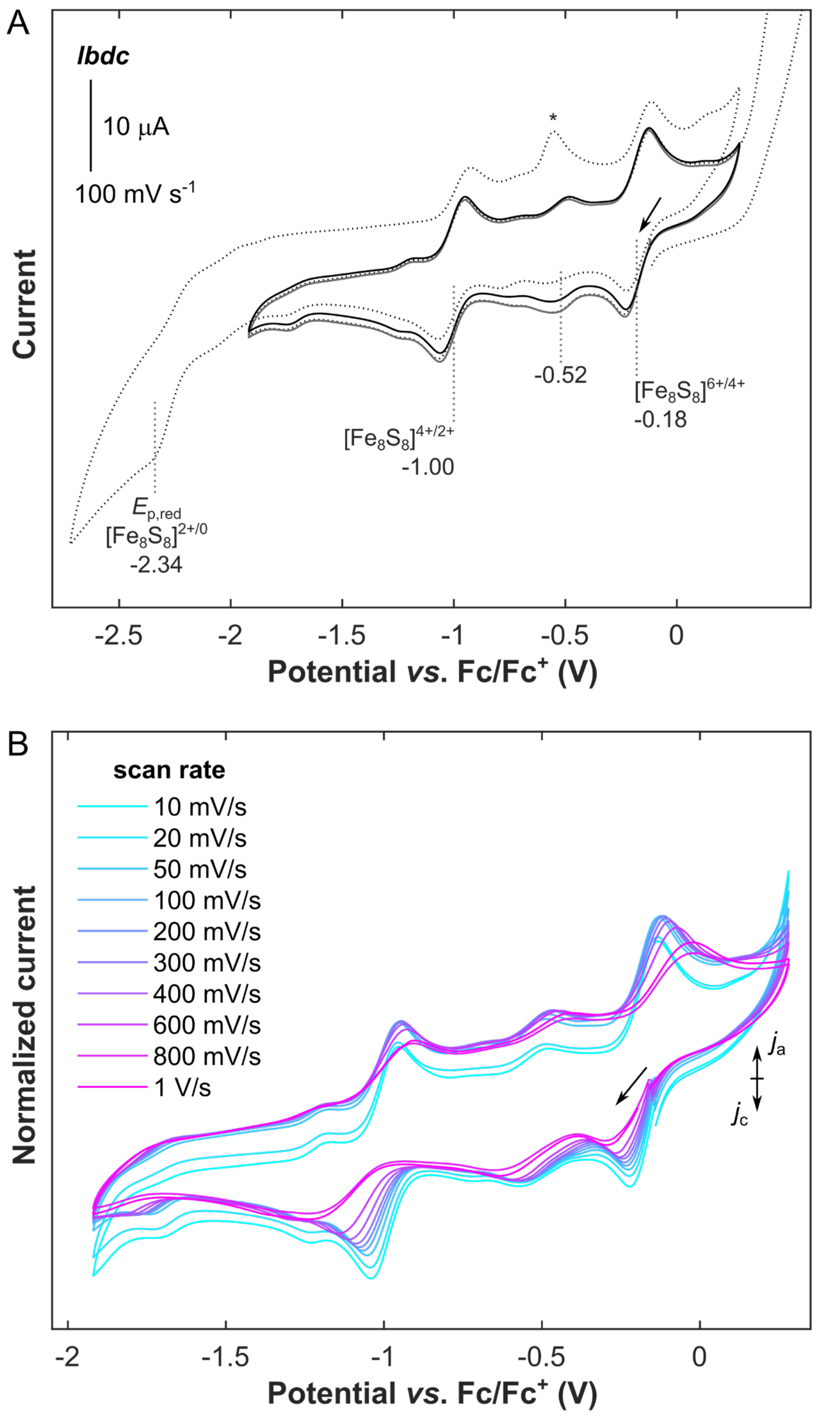

Supplementary Figure 71. (A) Cyclic voltammogram of a 2 mM *o*-DFB solution of $[Fe_8S_8(DmpS)_6]$ (***lbdc***) recorded in 0.1 M $[^nBu_4N][PF_6]$ as supporting electrolyte salt. Subsequent scans are shown in shades of *grey* and a *black arrow* marks the starting point and direction for the first scan. Dotted lines show scans over wider potential ranges. The feature marked by an asterisk (*) only appears after traversing the irreversible cathodic current response at –2.34 V. (B) Scan rate dependence of the voltammogram shown in panel (A).

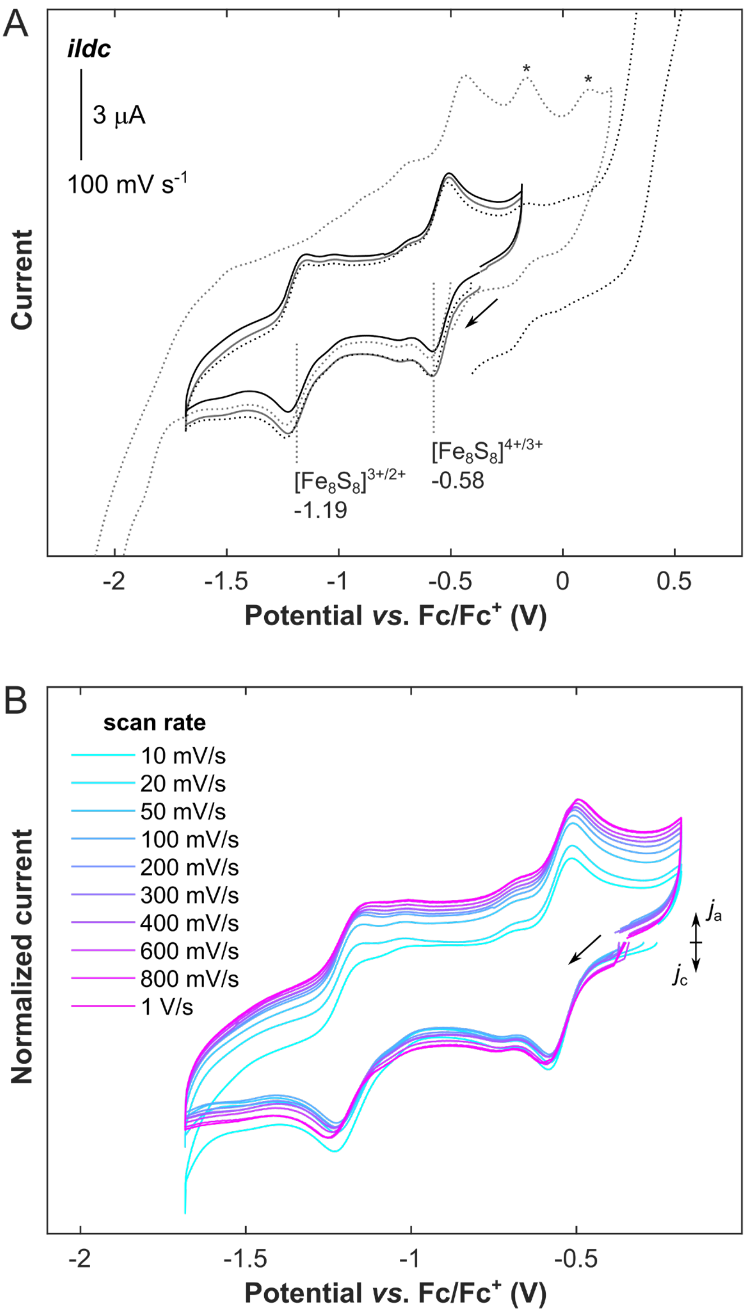

Supplementary Figure 72. (A) Cyclic voltammogram of a 1 mM (or saturated) DCM solution of $[Fe_8S_8(DmpS)_4]$ (***ildc***) recorded in 0.1 M $[^nBu_4N][PF_6]$ as supporting electrolyte salt. Subsequent scans are shown in shades of *grey* and a *black arrow* marks the starting point and direction for the first scan. Dotted lines show scans over wider potential ranges. The features marked by an asterisk (*) only appear after traversing the irreversible cathodic current response at approximately –1.9 V. (B) Scan rate dependence of the voltammogram shown in panel (A).

Crystallographic Details

Black, block-shaped single crystals of $[Fe_2S_2(DmpS)_2(py)_2]\cdot(py)_2$ were grown by layering a pyridine solution with pentane at room temperature. Large, black, block-shaped single-crystals of $K_4[Fe_8S_8(DmpS)_6]\cdot3.5(C_7H_8)$ were grown by vapor diffusion of pentane into a dilute toluene solution of the complex at room temperature. Small black block-shaped single-crystals of $[Fe_8S_8(DmpS)_6]\cdot3(C_7H_8)$ were grown by vapor diffusion of pentane into a very concentrated toluene solution of the complex at room temperature. Black block-shaped single-crystals of $[Fe_8S_8(DmpS)_4]$ were grown by layering a dilute toluene solution of ***Ibdc*** with pentane upon prolonged standing in an undisturbed location. Very small black block-shaped single-crystals of $[Fe_4S_4(DmpS)_2(Im)_2]\cdot K[Fe_4S_4(DmpS)_3(Im)]\cdot2(C_7H_8)$ were grown by vapor diffusion of pentane into a toluene solution of $K[Fe_4S_4(DmpS)_4]$ and a stoichiometric amount of Im at room temperature. The same mixture produced single-crystals of $K_2[Fe_4S_4(DmpS)_4]\cdot3(C_7H_8)$.[2] Large black block-shaped single crystals of $[Fe_4S_4(DmpS)_3(Im)]\cdot0.5[Fe(Im)_6]\cdot3(C_7H_8)$ were grown by vapor diffusion of pentane into a toluene solution of $[Fe_4S_4(DmpS)_4]$ containing excess Im. Black block-shaped crystals of $[Fe_{10}S_{10}(DmpS)_6(Im)_2]\cdot3(C_5H_{12})$ were grown by layering a toluene solution of $[Fe_4S_4(DmpS)_4]$ in presence of 0.9 equiv. of Im with pentane at room temperature. Large black rhombohedral single-cystals of $[Fe_4S_4(DmpS)_4]\cdot0.5[Fe(MeCN)_6]\cdot4(MeCN)$ were grown by prolonged standing of a MeCN solution of $[Fe_4S_4(DmpS)_4]$ layered with toluene. Single crystals of $[Fe_4S_4(DmpS)_3(THF)_3]$ were grown by layering a THF solution of $[Fe_4S_4(DmpS)_4]$ (or ***Ibdc***) with copious amounts of HMDSO at room temperature. Large pale yellow and colourless single-crystals of $(DmpS)_2$ and DmpSSSDmp were isolated from the same mixture, respectively. Large colourless block-shaped single-crystals and black block-shaped single-crystals of $Im^*\cdot B(C_6F_5)_3$ and $[Fe_{24}S_{24}(DmpS)_{10}]\cdot8(C_7H_8)$, respectively, were grown by vapor diffusion of pentane into a toluene solution of $[Fe_4S_4(DmpS)_2(Im^*)_2]$ in presence of 2 equiv. of $B(C_6F_5)_3$. Black irregularly shaped single-crystals of $K_6[Fe_{12}S_{12}(DmpS)_6]\cdot2(C_5H_{12})\cdot3(C_7H_8)$ were grown vapor diffusion of pentane into a dilute toluene solution of the compound at room temperature. Black, irregularly shaped single-crystals of $[Fe_8S_7(DmpS)_3Cl_2]$ and yellow plate-shaped single-crystals of $[Fe(DmpS)Cl]_2$ were grown upon prolonged standing of a DCM solution of $[Fe_4S_4(DmpS)_4]$ layered with an equivalent amount of pentane at room temperature. Black, needle-shaped single-crystals of $[Fe_4S_4(DmpS)_3(Im^*)]\cdot(C_7H_8)_{0.5}\cdot(C_6H_{18}Si_2O)_{0.5}$ were grown by layering a concentrated toluene solution with a generous amount of HMDSO at room temperature. Irregular, prism-shaped single crystals of $K[Fe_4S_4(DmpS)_3(Im^*)]$ were grown upon prolonged undisturbed standing of a toluene/pentane (*ca.* 1:1) solution of the compound at ambient temperature. Large elongated block shaped single crystals of $[Fe_4S_4(DmpS)_2(Im^*)_2]\cdot(C_4H_8O)$ were grown from a THF solution layered with pentane at room temperature. Aggregates of small irregularly-shaped single crystals of $^{[18\text{-}C\text{-}6]}K[Fe_4S_4(DmpS)_3(Im^*)]\cdot2.57(C_7H_8)\cdot1.43(C_5H_{12})$ were grown by layering a toluene solution with pentane at room temperature. Colourless, block-shaped single crystals of $DmpSK\cdot(C_4H_8O)_{0.4}$ were grown by prolonged standing of a THF/pentane (1:1) solution of the compound (10 mg in 3 mL) at –35°C. Colourless, block-shaped single crystals of $DmpS^{[18\text{-}C\text{-}6]}K\cdot(C_4H_8O)$ were grown by prolonged standing of a saturated THF/diethyl ether (2:1) solution of the compound at –35°C. Light yellow block-shaped single crystals of $(DmpS)_2$ were grown from a THF solution of the compound layered with pentane at –35°C.

The suitable crystals were embedded in perfluoropolyalkylether oil inside an argon-filled glovebox equipped with a microscope. Subsequently, they were transferred from a sealed vessel under argon into the cold nitrogen gas stream of the diffractometer as fast as possible. Intensity data of all investigated compounds, except that of ***Ibdc***, were collected at 100 K. Data for ***Ibdc*** was instead collected at 180 K, because the crystals underwent a phase transition at lower *T*, leading to shattering of the crystals in the cold gas stream. Data for $[Fe_2S_2(DmpS)_2(py)_2]\cdot(py)_2$, $[Fe_4S_4(DmpS)_4]\cdot0.5[Fe(MeCN)_6]\cdot4(MeCN)$, $K[Fe_4S_4(DmpS)_3(Im^*)]$ and $[Fe_8S_7(DmpS)_3Cl_2]$ were collected on a Bruker Venture D8 diffractometer equipped with a Bruker PhotonII detector using Mo-K_α radiation. Data for the remainder of the compounds were collected on a Rigaku XtaLAB Synergy-

S diffractometer with a HyPix-6000HE detector using Cu-K_α, or Mo-K_α radiation, respectively, as summarized in Supplementary Tables 1-7 and 9.

After data collection, structures were solved by intrinsic phasing (SHELXT) and refined by full-matrix least squares procedures on F^2 using SHELXL in the Olex2 program suite.[34-37] All non-hydrogen atoms were refined with anisotropic displacement parameters. The hydrogen atoms were placed in positions of optimized geometry. Crystallographic data, data collection and structure refinement details for the crystal structure determinations are given in Supplementary Tables 1-7 and 9. Representations of the molecular structures are shown in Supplementary Figs. 73-96.
When necessary, fragments such as disordered solvent molecules were refined using appropriate parts and restraints according to Kratzert's fragment database—as implemented in Olex2 (*vide infra*).[38,39]

No structures resulted in Level A IUCR checkcif alerts. All Level B alerts were addressed directly within the correspondings checkcif and .cif files, which are provided as Supplemental Materials and are also deposited in the CSD database under the accession numbers 2389108-2389127 and 2389327-2389329 as indicated in the Tables below. The Level B alert "PLAT910_ALERT_3_B" (for missing reflections at lower angles) is re-occurring for multiple structures. Typically we observed this alert for structures which were measured using Mo K_α radiation, especially on the Bruker Venture D8 diffractometer. Because the missing reflections are limited to those at lower angles, which are often affected by the beamstop, they do not impact the quality of the structural data. Instead, the higher-angle reflections are sufficient for a reliable structure determination.

Crystallographer’s remarks and tables

The asymmetric unit in $[Fe_4S_4(DmpS)_3(Im^*)]\cdot(C_7H_8)_{0.5}\cdot(C_6H_{18}Si_2O)_{0.5}$ contained solvent molecules of toluene and HMDSO in the same solvent-accessible void. Thus, the two molecules were refined in parts, and using appropriate restraints from Kratzert’s fragment database as implemented in Olex2.[38,39] The final occupancies of the two molecules refined to 0.49:0.25.

The asymmetric unit of $^{[18\text{-}C\text{-}6]}K[Fe_4S_4(DmpS)_3(Im^*)]\cdot 2.57(C_7H_8)\cdot 1.43(C_5H_{12})$ contained several disordered solvent molecules, which were refined in parts, using appropriate restraints as implemented in Kratzert’s fragment database in Olex2. One of the two [18-C-6](K) fragments was likewise severely disordered, and modelled appropriately. The two fragments refined to an occupancy ratio of 0.56:0.44.

The asymmetric unit of $K_4[Fe_8S_8(DmpS)_6]\cdot 3.5(C_7H_8)$ contained three disordered toluene molecules, which were refined in parts using appropriate constraints and restraints. The fourth toluene molecule resides on the symmetry plane, and was thus fixed to an occupancy of 0.5.

The asymmetric unit of $[Fe_8S_8(DmpS)_6]\cdot 3(C_7H_8)$ contains three molecules of toluene, one of which was disordered. In the final refinement, parts, constraints and restraints were appropriately assigned, yielding and occupancy ratio of 0.41:0.59.

The asymmetric unit of $[Fe_8S_8(DmpS)_4]$ contains solvent accessible voids of 105 $Å^3$. However, there is no significant residual electron density present in this void.
When crystallized from CH_2Cl_2, this void, located on the symmetry plane, is occupied by a disordered solvent molecule (50:50 occupancy ratio), which can be refined using appropriate parts (0 and -2, respectively).

The asymmetric unit of $[Fe_4S_4(DmpS)_3(Im)]\cdot 0.5[Fe(Im)_6]\cdot 3(C_7H_8)$ contains 1.5 disordered toluene molecules, the half-fragment of which is located on the symmetry plane. Accordingly, they were modelled using appropriate restraints and constraints and refined in parts. The occupancy ratio of the disordered complete molecule refined to 0.24:0.76.

The asymmetric unit of $[Fe_{10}S_{10}(DmpS)_6(Im)_2]\cdot 3(C_5H_{12})$ contains three pentane molecules, one of which was disordered more severely than the others. All molecules were refined using constrains and restraints as implemented in the fragment database and the severely disordered one was refined in parts, yielding the final occupancy ratio of 0.27:0.73.

The asymmetric unit of $[Fe_4S_4(DmpS)_4]\cdot 0.5[Fe(MeCN)_6]\cdot 4(MeCN)$ contains four molecules of solvent, two of which were disordered. The corresponding fragments were refined in parts, finally yielding occupancy ratios of 0.38:0.62 and 0.24:0.76, respectively.

The asymmetric unit of $[Fe_4S_4(DmpS)_3(THF)_3]$ contains two thirds of two individual molecules. One of the two thirds possesses disordered THF ligands, which were refined in parts using appropriate restraints. The final occupancy ratio amounted to 0.44:0.56.

The crystal of DmpSSSDmp under investigation was twinned, with volume-fractions of 0.30:0.70 for the individual components. Accordingly, a hklf5-refinement was performed.

The asymmetric unit of $[Fe_{24}S_{24}(DmpS)_{10}]\cdot 8(C_7H_8)$ contained six voids filled with solvent molecules. By the electron density it became evident that four of these voids were filled with fractional solvent molecules. Fixing the occupancy of these to a value of 0.5 gave a reasonable fit to the data, which is why this value was used for the final refinement.

The asymmetric unit of $K_6[Fe_{12}S_{12}(DmpS)_6]$ contained a well-resolved molecule of pentane, which was modelled and refined using appropriate constrains and restraints from the fragment database. Still, large voids with significant diffuse electron density remained. Therefore, a solvent mask was calculated and 684 electrons were found in a volume of 336 Å^3 in 1 void per unit cell. This is consistent with the presence of 1/3 C_7H_8 per asymmetric unit which account for 600 electrons per unit cell.

The crystal of $[Fe_8S_7(DmpS)_3Cl_2]$ under investigation was twinned by inversion, and refined using the appropriate twin law, yielding volume fractions of 0.23:0.77. Furthermore, one of the two Fe atoms in its asymmetric unit is disordered, and was refined in parts, resulting in an occupancy ratio of 0.13:0.87. This aligns with the notion that one of the six Fe-atoms in the molecule, which are related by symmetry is crystallographically unique.

The asymmetric unit of $DmpSK\cdot(C_4H_8O)_{0.4}$ contained a disordered molecule of THF, which was refined using appropriate restraints and a free occupancy, converging at a value of 0.39.

The asymmetric unit of $DmpS^{[18\text{-}C\text{-}6]}K\cdot(C_4H_8O)$ contained a disordered molecule of THF, which was refined in parts, using appropriate restraints.

The asymmetric unit of $Im^*\cdot B(C_6F_5)_3$ contains disordered poorly resolved solvent molecules. These were modeled using the fragment database as a pentane and toluene molecule with fractional occupancies of 0.25 and 0.20, respectively.

Supplementary Table 1. Crystallographic data, data collection and refinement details for $[Fe_2S_2(DmpS)_2(py)_2]$, ***ebdc*** and ***lbdc***.

**parameter**	$[Fe_2S_2(DmpS)_2(py)_2]\cdot 2(py)$	$K_4[Fe_8S_8(DmpS)_6]\cdot 3.5(C_7H_8)$	$[Fe_8S_8(DmpS)_6]\cdot 3(C_7H_8)$
empiric formula	$C_{68}H_{70}S_4Fe_2N_4$	$C_{168.5}H_{178}Fe_8K_4S_{14}$	$C_{165}H_{174}Fe_8S_{14}$
M [g mol^{-1}]	1183.22	3255.14	3052.67
crystal size [mm]	0.38 × 0.18 × 0.088	0.57 × 0.12 × 0.11	0.17 × 0.09 × 0.06
temperature	100.0 K	100.0 K	180.0 K
radiation type	Mo K_α	Mo K_α	Cu K_α
crystal system	*monoclinic*	*triclinic*	*monoclinic*
space group (no.)	$P2_1/c$ (14)	P-1 (2)	$P2_1/c$(14)
a [Å]	8.8107(4)	18.6528(2)	19.3317(2)
b [Å]	14.7323(7)	19.9210(2)	52.3232(5)
c [Å]	24.0369(10)	22.5715(2)	15.75500(10)
α [°]	90	84.4270(10)	90
β [°]	98.758(2)	84.3200(10)	102.6140(10)
γ [°]	90	72.8260(10)	90
V [Å^3]	3083.7(2)	7953.51(14)	15551.5(2)
Z, *Z'*	2, 0.5	2, 1	4, 1
μ [mm^{-1}]	0.649	1.047	7.936
F(000)	1244.0	3394.0	6384.0
abs. corr.	SADABS	SCALE3 ABSPACK	SCALE3 ABSPACK
T_{min}; T_{max}	0.6665; 0.7456	0.7662; 1.0000	0.8256; 1.0000
2θ-range [°]	4.404 to 55.07	4.352 to 57.4	4.684 to 160.838
coll. refl.	91570	163697	209531
indep. refl.	7105 with R_{int}=0.0630 R_σ=0.0254	41013 with R_{int}=0.0517 R_σ=0.0456	33502 with R_{int}=0.0991 R_σ=0.0447
restraints/param.	0/358	792/1790	670/1644
wR_2	0.0715	0.0958	0.1591
$R_1(F_0 \geq 4.0\sigma(F))$	0.0316	0.0373	0.0615
GooF F^2	1.022	1.033	1.052
$\Delta\rho$ (max; min) [e Å^{-3}]	0.34; –0.26	1.95; –1.10	0.87; –0.63
CSD number	2389114	2389123	2389116
identifier	lg_c96_6_mono	lg-c260_auto	mlw026_3_auto

Supplementary Table 2. Crystallographic data, data collection and refinement details for ***ildc***, $[Fe_4S_4(DmpS)_2(Im)_2]$, and $K[Fe_4S_4(DmpS)_3(Im)]$.

**parameter**	$[Fe_8S_8(DmpS)_4]$	$[Fe_8S_8(DmpS)_4]\cdot CH_2Cl_2$	$[Fe_4S_4(DmpS)_2(Im)_2]$ $\cdot K[Fe_4S_4(DmpS)_3(Im)]\cdot 2(C_7H_8)$
empiric formula	$C_{96}H_{100}S_{12}Fe_8$	$C_{97}H_{102}Fe_8S_{12}Cl_2$	$C_{146}H_{159}Fe_8KN_6S_{13}$
M [g mol^{-1}]	2085.27	2170.20	2900.46
crystal size [mm]	0.19 × 0.08 × 0.04	0.04 × 0.04 × 0.02	0.18 × 0.06 × 0.05
temperature	100.0 K	100.0 K	100.0 K
radiation type	Cu K_α	Cu K_α	Cu K_α
crystal system	*monoclinic*	*monoclinic*	*triclinic*
space group (no.)	$P2_1/m$ (11)	$P2_1/m$ (11)	P-1 (2)
a [Å]	10.9661(2)	11.00887(10)	19.83630(11)
b [Å]	36.5202(3)	35.5994(3)	28.06014(13)
c [Å]	12.6263(2)	12.63142(13)	28.29498(17)
α [°]	90	90	101.7806(5)
β [°]	114.269(2)	106.0703(10)	109.5407(5)
γ [°]	90	90	93.6107(4)
V [Å^3]	4609.76(13)	4756.92(8)	14381.70(14)
Z, *Z'*	2, 0.5	2, 0.5	8, 4
μ [mm^{-1}]	12.683	12.820	8.685
F(000)	2152.0	2236.0	6048.0
abs. corr.	SCALE3 ABSPACK	SCALE3 ABSPACK	SCALE3 ABSPACK
T_{min}; T_{max}	0.2639; 1.0000	0.5947; 1.0000	0.6449; 1.0000
2θ-range [°]	4.84 to 160.81	7.284 to 160.51	4.11 to 161.364
coll. refl.	59989	48290	376654
indep. refl.	10125 with R_{int}=0.0485 R_σ=0.0321	10395 with R_{int}=0.0466 R_σ=0.0320	62067 with R_{int}=0.0878 R_σ=0.0558
restraints/param.	0/547	0/569	136/3167
wR_2	0.1162	0.1110	0.1250
$R_1(F_0 \geq 4.0\sigma(F))$	0.0429	0.0438	0.0490
GooF F^2	1.058	1.040	1.067
$\Delta\rho$ (max; min) [e Å^{-3}]	0.70; –0.72	0.86; –0.98	1.10; –0.82
CSD number	2389115	2389110	2389111
identifier	lg-c319_1_auto	lg96_9_auto	lg-c278_5_autored

Supplementary Table 3. Crystallographic data, data collection and refinement details for $[Fe_4S_4(DmpS)_3(Im)]\cdot 0.5[Fe(Im)_6]$, $[Fe_{10}S_{10}(DmpS)_6(Im)_2]$, and $[Fe_4S_4(DmpS)_4]\cdot 0.5[Fe(MeCN)_6]$.

**parameter**	$[Fe_4S_4(DmpS)_3(Im)]$ $\cdot 0.5[Fe(Im)_6]\cdot 3(C_7H_8)$	$[Fe_{10}S_{10}(DmpS)_6(Im)_2]$ $\cdot 3(C_5H_{12})$	$[Fe_4S_4(DmpS)_4]$ $\cdot 0.5[Fe(MeCN)_6]\cdot 4(MeCN)$
empiric formula	$C_{98.5}H_{111}Fe_{4.5}N_8S_7$	$C_{167}H_{198}Fe_{10}N_4S_{16}$	$C_{110}H_{121}Fe_{4.5}N_7S_8$
M [g mol^{-1}]	1882.69	3332.74	2048.94
crystal size [mm]	0.71 × 0.09 × 0.09	0.88 × 0.17 × 0.11	0.71 × 0.66 × 0.54
temperature	100.0 K	100.0 K	100.0 K
radiation type	Mo K_α	Mo K_α	Mo K_α
crystal system	*triclinic*	*monoclinic*	*triclinic*
space group (no.)	P-1 (2)	$P2_1/c$ (14)	P-1 (2)
a [Å]	14.6425(3)	16.4049(3)	15.7289(8)
b [Å]	15.1253(3)	26.9113(7)	16.3152(8)
c [Å]	22.7773(4)	36.8410(9)	23.2698(11)
α [°]	105.8443(16)	90	84.285(2)
β [°]	91.2887(15)	90.2000(10)	83.982(2)
γ [°]	99.8180(15)	90	64.443(2)
V [Å^3]	4769.11(16)	16264.5(6)	5347.7(5)
Z, *Z*'	2, 1	4, 1	2, 1
μ [mm^{-1}]	0.872	1.120	0.802
F(000)	1974.0	6976.0	2150.0
abs. corr.	SCALE3 ABSPACK	SCALE3 ABSPACK	SADABS
T_{min}; T_{max}	0.4003; 1.0000	0.4994; 1.0000	0.6125; 0.7455
2θ-range [°]	4.336 to 65.226	4.412 to 54.206	4.352 to 54.374
coll. refl.	112364	126888	173797
indep. refl.	29007 with R_{int}=0.0587 R_σ=0.0561	35154 with R_{int}=0.0376 R_σ=0.0354	23636 with R_{int}=0.0469 R_σ=0.0270
restraints/param.	272/1110	188/1836	26/1228
wR_2	0.1110	0.0865	0.0911
$R_1(F_0 \geq 4.0\sigma(F))$	0.0494	0.0347	0.0414
GooF F^2	1.016	1.027	1.072
$\Delta\rho$ (max; min) [e Å^{-3}]	0.83; –0.75	1.67; –0.95	1.30; –0.50
CSD number	2389112	2389122	2389124
identifier	hs013_1_auto	lg-c249_2_mo_auto	mo_lg_c96_3_0m

Supplementary Table 4. Crystallographic data, data collection and refinement details for $[Fe_4S_4(DmpS)_3(THF)_3]$, DmpSSSDmp, and Im*·$C(C_6F_5)_3$.

**parameter**	$[Fe_4S_4(DmpS)_3(THF)_3]$	DmpSSSDmp	Im*·$C(C_6F_5)_3$
empiric formula	$C_{168}H_{198}Fe_8O_6S_{14}$	$C_{24}H_{25}S_{1.5}$	$C_{32}H_{20}BF_{15}N_2$
M [g mol^{-1}]	3208.89	361.53	728.31
crystal size [mm]	0.17 × 0.15 × 0.07	0.3 × 0.12 × 0.09	0.72 × 0.65 × 0.5
temperature	100.0 K	100.0 K	100.0 K
radiation type	Cu K_α	Cu K_α	Mo K_α
crystal system	*trigonal*	*monoclinic*	*monoclinic*
space group (no.)	R3c (161)	C2/m (12)	C2/c (15)
a [Å]	22.03025(16)	9.8923(2)	25.2693(16)
b [Å]	22.03025(16)	22.8864(5)	13.1755(7)
c [Å]	57.5916(6)	8.7506(2)	20.8647(12)
α [°]	90	90	90
β [°]	90	98.135(2)	125.852(4)
γ [°]	120	90	90
V [Å^3]	24206.3(4)	1961.19(7)	5630.4(6)
Z, *Z'*	6, 1	4, 0.5	8, 1
μ [mm^{-1}]	7.700	1.964	0.170
F(000)	10116.0	772.0	2928.0
abs. corr.	SCALE3 ABSPACK	SCALE3 ABSPACK	SADABS
T_{min}; T_{max}	0.7813; 1.0000	0.8888; 1.0000	0.6118; 0.7472
2θ-range [°]	5.556 to 160.906	7.726 to 160.63	3.676 to 56.564
coll. refl.	44787	21753	104513
indep. refl.	10214 with R_{int}=0.0453 R_σ=0.0432	21753 with R_{int}=0.050 R_σ=0.0466	6990 with R_{int}=0.0646 R_σ=0.0204
restraints/param.	1/593	0/127	0/392
wR_2	0.0837	0.1454	0.2439
$R_1(F_0 \geq 4.0\sigma(F))$	0.0334	0.0544	0.0915
GooF F^2	1.061	1.072	1.175
$\Delta\rho$ (max; min) [e Å^{-3}]	0.52; –0.46	0.41; –0.36	0.65; –0.97
CSD number	2389127	2389125	2389120
identifier	hs_14_3_auto	hs_14_2_twin1_hklf4	mo_lg_c298_2

Supplementary Table 5. Crystallographic data, data collection and refinement details for $[Fe_{24}S_{24}(DmpS)_{10}]$, $K_6[Fe_{12}S_{12}(DmpS)_6]$ and $[Fe_4S_4(DmpS)_3(Im^*)]$.

**parameter**	$[Fe_{24}S_{24}(DmpS)_{10}]\cdot 8(C_7H_8)$	$K_6[Fe_{12}S_{12}(DmpS)_6]$	$[Fe_4S_4(DmpS)_3(Im^*)]\cdot(C_7H_8)_{0.5}\cdot(C_6H_{18}Si_2O)_{0.5}$
empiric formula	$C_{296}H_{314}Fe_{24}S_{34}$	$C_{77}H_{87}Fe_6K_3S_9$	$C_{79}H_{87}Fe_4N_2S_7$, $0.255(C_6H_{18}OSi_2)$, $0.495(C_7H_8)$
M [g mol^{-1}]	6301.89	1753.40	1599.34
crystal size [mm]	0.31 × 0.18 × 0.18	0.41 × 0.22 × 0.11	0.49 × 0.07 × 0.06
temperature	100.0 K	100.0 K	100 K
radiation type	Mo K_{α}	Cu K_{α}	Cu K_{α}
crystal system	*triclinic*	*trigonal*	*monoclinic*
space group (no.)	P-1 (2)	R-3c (167)	$P2_1/c$ (14)
a [Å]	18.9603(13)	21.5876(6)	16.36400(10)
b [Å]	19.1739(14)	21.5876(6)	15.22200(10)
c [Å]	23.8708(18)	64.7637(15)	32.0588(2)
α [°]	100.929(3)	90	90
β [°]	111.951(3)	90	92.3730(10)
γ [°]	107.079(3)	120	90
V [Å^3]	7242.1(9)	26137.9(16)	7978.77(9)
Z, *Z'*	1, 0.5	12, 0.16	4, 1
μ [mm^{-1}]	1.456	11.382	7.838
F(000)	3258.0	10872.0	3355.0
abs. corr.	SADABS	SCALE3 ABSPACK	SCALE3 ABSPACK
T_{min}; T_{max}	0.6129; 0.7454	0.31826; 1.0000	0.2476; 1.0000
2θ-range [°]	3.824 to 53.324	8.192 to 160.412	5.406 to 161.678
coll. refl.	316609	43952	104990
indep. refl.	30206 with R_{int}=0.0558 R_{σ}=0.0298	6316 with R_{int}=0.0687 R_{σ}=0.0292	17284 with R_{int}=0.0474 R_{σ}=0.0325
restraints/param.	764/1684	61/301	301/1004
wR_2	0.1550	0.2369	0.0832
$R_1(F_0 \geq 4.0\sigma(F))$	0.0758	0.0834	0.0317
GooF F^2	1.093	1.051	1.072
$\Delta\rho$ (max; min) [e Å^{-3}]	2.34; –1.12	1.33; –0.52	0.57; –0.39
CSD number	2389119	2389109	2389126
identifier	mo_lg_c298_1_0m	lg-c293_6_cu_auto	lg-c312_2_auto

Supplementary Table 6. Crystallographic data, data collection and refinement details for K[Fe_4S_4(DmpS)$_3$(Im*)], [18-C-6]K[Fe_4S_4(DmpS)$_3$(Im*)] and K[Fe_4S_4(DmpS)$_2$(Im*)$_2$].

**parameter**	K[Fe_4S_4(DmpS)$_3$(Im*)]	[18-C-6]K[Fe_4S_4(DmpS)$_3$(Im*)] ·2.57(C_7H_8)·1.43(C_5H_{12})	[Fe_4S_4(DmpS)$_2$(Im*)$_2$] ·(C_4H_8O)
empiric formula	$C_{79}H_{87}Fe_4KN_2S_7$	$C_{79}H_{87}Fe_4N_2S_7$, 2.57(C_7H_8), 1.43(C_5H_{12})	$C_{62}H_{74}Fe_4N_4S_6$, C_4H_8O
M [g mol^{-1}]	1551.43	1984.79	1363.12
crystal size [mm]	0.51 × 0.27 × 0.09	0.39 × 0.11 × 0.1	0.61 × 0.15 × 0.06
temperature	100 K	100 K	100 K
radiation type	Mo K_α	Cu K_α	Cu K_α
crystal system	*monoclinic*	*monoclinic*	*orthorhombic*
space group (no.)	$P2_1/c$ (14)	$P2_1/n$ (14)	$P2_12_12$ (18)
a [Å]	13.3595(6)	16.30310(10)	21.1396(2)
b [Å]	45.203(2)	46.5522(4)	17.65280(10)
c [Å]	13.4598(7)	26.8420(2)	8.85770(10)
α [°]	90	90	90
β [°]	114.185(2)	94.9840(10)	90
γ [°]	90	90	90
V [Å^3]	7414.8(6)	20294.6(3)	3305.46(5)
Z, *Z'*	4, 1	8, 2	2, 0.5
μ [mm^{-1}]	1.065	6.613	9.003
F(000)	3240.0	8379.0	1428.0
abs. corr.	SADABS	SCALE3 ABSPACK	SCALE3 ABSPACK
T_{min}; T_{max}	0.6429; 0.7465	0.7140; 1.0000	0.1707; 1.0000
2θ-range [°]	3.728 to 66.524	5.034 to 136.496	6.524 to 160.758
coll. refl.	308948	255261	23447
indep. refl.	28503 with R_{int}=0.0502 R_σ=0.0276	37128 with R_{int}=0.0754 R_σ=0.0363	6976 with R_{int}=0.0464 R_σ=0.0394
restraints/param.	0/860	1668/2065	75/398
wR_2	0.1920	0.2656	0.0804
R_1(F_0≥4.0σ(*F*))	0.0806	0.0968	0.0330
GooF F^2	1.230	1.071	1.051
$\Delta\rho$ (max; min) [e Å^{-3}]	1.71; –0.87	1.15; –0.84	0.61; –0.42
CSD number	2389118	2389117	2389113
identifier	mono	mlw008_d_1_auto	lg-c277_1_auto

Supplementary Table 7. Crystallographic data, data collection and refinement details for $[Fe_8S_7(DmpS)_3Cl_2]$ and $[Fe(DmpS)Cl]_2$.

**parameter**	$[Fe_8S_7(DmpS)_3Cl_2]$	$[Fe(DmpS)Cl]_2$
empiric formula	$C_{72}H_{75}S_{10}Cl_2Fe_8$	$C_{48}H_{50}S_2Fe_2Cl_2$
M [g mol^{-1}]	1778.62	873.60
crystal size [mm]	0.26 × 0.12 × 0.12	0.22 × 0.13 × 0.11
temperature	100.0 K	100.0 K
radiation type	Mo K_α	Cu K_α
crystal system	*hexagonal*	*triclinic*
space group (no.)	$P6_3/m$ (176)	P-1 (2)
a [Å]	13.2003(4)	9.9892(5)
b [Å]	13.2003(4)	11.3747(5)
c [Å]	23.9400(8)	11.4445(5)
α [°]	90	60.738(5)
β [°]	90	75.742(4)
γ [°]	120	66.071(4)
V [Å^3]	3612.6(3)	1035.05(10)
Z, *Z*'	2, 0.16	1, 0.5
μ [mm^{-1}]	1.964	7.997
F(000)	1818.0	456.0
abs. corr.	SADABS	SCALE3 ABSPACK
T_{min}; T_{max}	0.6711; 0.7456	0.8687; 1.0000
2θ-range [°]	4.928 to 55.808	8.874 to 160.758
coll. refl.	163990	14484
indep. refl.	2955 with R_{int}=0.1123 R_σ=0.0202	4392 with R_{int}=0.0376 R_σ=0.0383
restraints/param.	0/152	0/250
wR_2	0.0766	0.1057
$R_1(F_0 \geq 4.0\sigma(F))$	0.0365	0.0404
GooF F^2	1.168	1.118
$\Delta\rho$ (max; min) [e Å^{-3}]	1.19; –0.62	0.45; –0.78
CSD number	2389121	2389108
identifier	lg_c103_4_hexa	lg_c103_7_auto

Supplementary Table 8. Selected Structural parameters of selected Fe_4S_4 complexes introduced in this work (*vide supra*).

**parameter**	$[Fe_4S_4(DmpS)_3(Im^*)]$	$K[Fe_4S_4(DmpS)_3(Im^*)]$	$[Fe_4S_4(DmpS)_2(Im^*)_2]$
Fe-S av. (Å)	2.269(2)	2.280(3)	2.286(1)
Fe-SR av. (Å)	2.219(3)	2.253(5)	2.255(1)
Fe-N av. (Å)	1.997(2)	2.033(4)	2.018(3)
Fe-Fe av. (Å)	2.743(2)	2.745(4)	2.712(1)
$V(Fe_4)$ (Å^3)	2.43	2.43	2.34
$V(S_4)$ (Å^3)	5.34	5.46	5.52

Supplementary Table 9. Crystallographic data, data collection and refinement details for DmpSK·$(C_4H_8O)_{0.4}$, DmpS[18-C-6]K·(C_4H_8O) and $(DmpS)_2$.

parameter	DmpSK·$(C_4H_8O)_{0.4}$	DmpS[18-C-6]K·(C_4H_8O)	$(DmpS)_2$
empiric formula	$C_{24}H_{25}KS$, 0.396(C_4H_8O)	$C_{36}H_{48}KO_6S$, C_4H_8O	$C_{48}H_{50}S_2$
M [g mol^{-1}]	413.15	720.01	691.00
crystal size [mm]	0.19 × 0.04 × 0.03	0.55 × 0.11 × 0.08	0.23 × 0.18 × 0.1
temperature	100 K	100 K	100 K
radiation type	Cu K_α	Cu K_α	Cu K_α
crystal system	*tetragonal*	*monoclinic*	*monoclinic*
space group (no.)	$I4_1/acd$ (142)	$P2_1/n$ (14)	$P2_1/n$ (14)
a [Å]	22.38180(10)	8.36222(5)	15.57610(10)
b [Å]	22.38180(10)	20.10566(16)	15.20230(10)
c [Å]	36.6919(3)	23.82038(16)	16.21400(10)
α [°]	90	90	90
β [°]	90	99.4077(6)	93.6580(10)
γ [°]	90	90	90
V [Å^3]	18380.6(2)	3951.00(5)	3831.53(4)
Z, *Z'*	32, 1	4, 1	4, 1
μ [mm^{-1}]	2.926	2.038	1.492
F(000)	7035.0	1548.0	1480.0
abs. corr.	SCALE3 ABSPACK	SCALE3 ABSPACK	SCALE3 ABSPACK
T_{min}; T_{max}	0.7907; 1.0000	0.8203; 1.0000	0.4735; 1.0000
2θ-range [°]	7.9 to 161.03	5.784 to 160.626	7.63 to 160.548
coll. refl.	46895	56000	53936
indep. refl.	5006 with R_{int}=0.0386 R_σ=0.0234	8537 with R_{int}=0.0414 R_σ=0.0297	8311 with R_{int}=0.0425 R_σ=0.0283
restraints/param.	20/259	150/464	0/463
wR_2	0.1431	0.1589	0.0996
$R_1(F_0 \geq 4.0\sigma(F))$	0.0537	0.0569	0.0374
GooF F^2	1.079	1.056	1.055
$\Delta\rho$ (max; min) [e Å^{-3}]	1.21; –0.39	0.91; –0.72	0.29; –0.35
CSD number	2389327	2389329	2389328
identifier	lg-l3_closed_auto	lg-l22_1_cu_auto	lg-l24_1_auto

BVS analysis of ***ildc***

Supplementary Table 10. Summary of the bond-valence-sum (BVS) analysis[40] of ***ildc***. The parameters were taken from [41], and the analysis accordingly carried out as described in [42].

Fe	S	r / Å	assumed oxidation state of Fe						state
			**+II**			**+III**			
			bond valence $s = e^{(R_0-R)/B}$	sum $\Sigma(s)$	difference $\lvert d\rvert = \lvert\Sigma(s) - 2\rvert$	bond valence $s = e^{(R_0-R)/B}$	sum $\Sigma(s)$	difference $\lvert d\rvert = \lvert\Sigma(s) - 3\rvert$	
1	2	2.165	0.8975	2.9203	0.9203	0.9577	3.1160	0.1160	**+III**
	4	2.245	0.7230			0.7715			
	3	2.265	0.6850			0.7309			
	1	2.305	0.6148			0.6560			
2	4	2.268	0.6794	2.7592	0.7592	0.7250	2.9441	0.0559	**+III**
	3	2.274	0.6685			0.7133			
	5	2.282	0.6542			0.6981			
	8	2.228	0.7570			0.8077			
3	3	2.289	0.6420	2.2282	0.2282	0.6850	2.3776	0.6224	**+II**
	1	2.505	0.3581			0.3821			
	5	2.297	0.6282			0.6703			
	6	2.314	0.6000			0.6402			
4	4	2.313	0.6016	2.0889	0.0889	0.6420	2.2289	0.7711	**+II**
	1	2.523	0.3411			0.3639			
	5	2.312	0.6033			0.6437			
	7	2.351	0.5429			0.5793			

Structure images

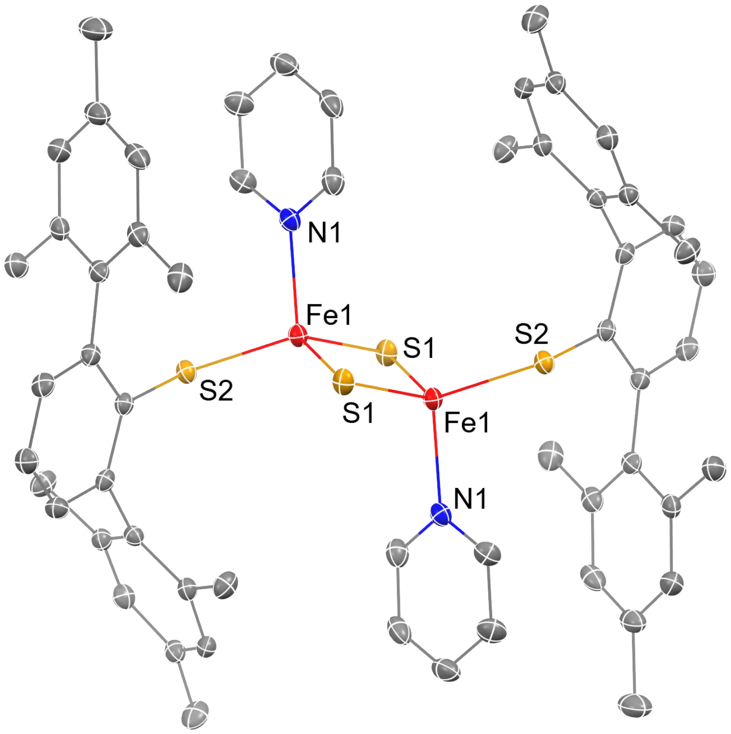

Supplementary Figure 73. Solid-state molecular structure of $[Fe_2S_2(DmpS)_2(py)_2]$ in crystals of $[Fe_2S_2(DmpS)_2(py)_2]\cdot 2(py)$. Displacement ellipsoids are shown at 50% probability. Hydrogen atoms as well as co-crystallized solvent molecules are omitted for clarity.

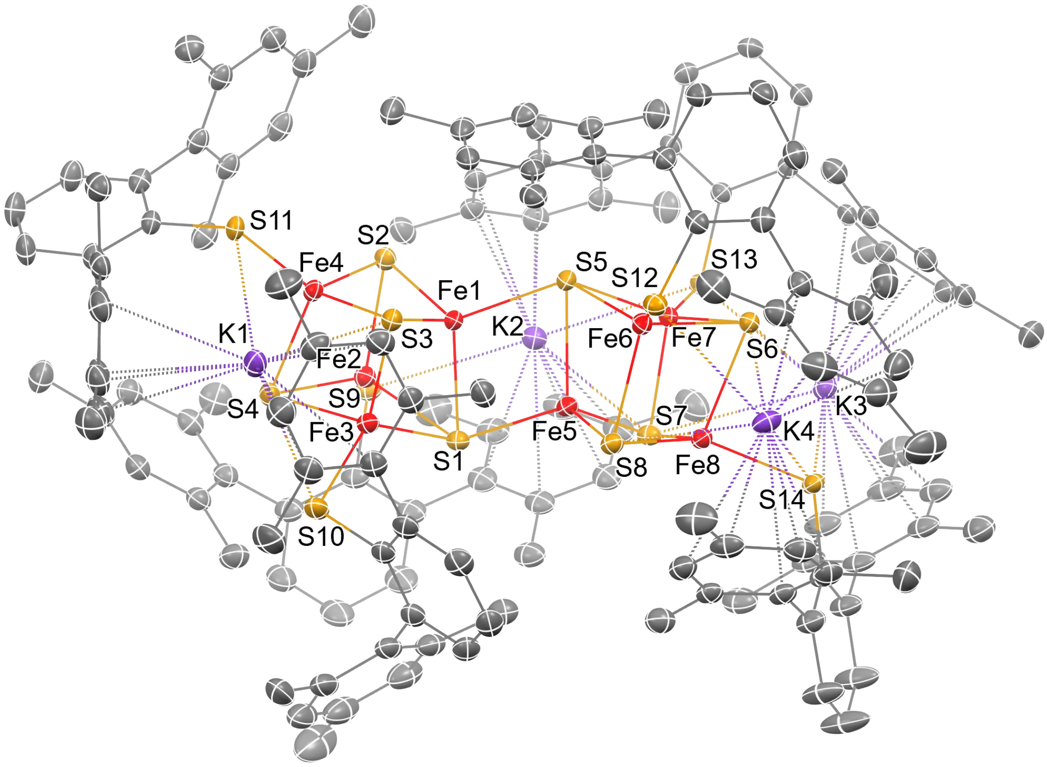

Supplementary Figure 74. Solid-state molecular structure of $K_4[Fe_8S_8(DmpS)_6]$ in crystals of $K_4[Fe_8S_8(DmpS)_6]\cdot 3.5(C_7H_8)$. Displacement ellipsoids are shown at 50% probability. Hydrogen atoms as well as co-crystallized solvent molecules are omitted for clarity.

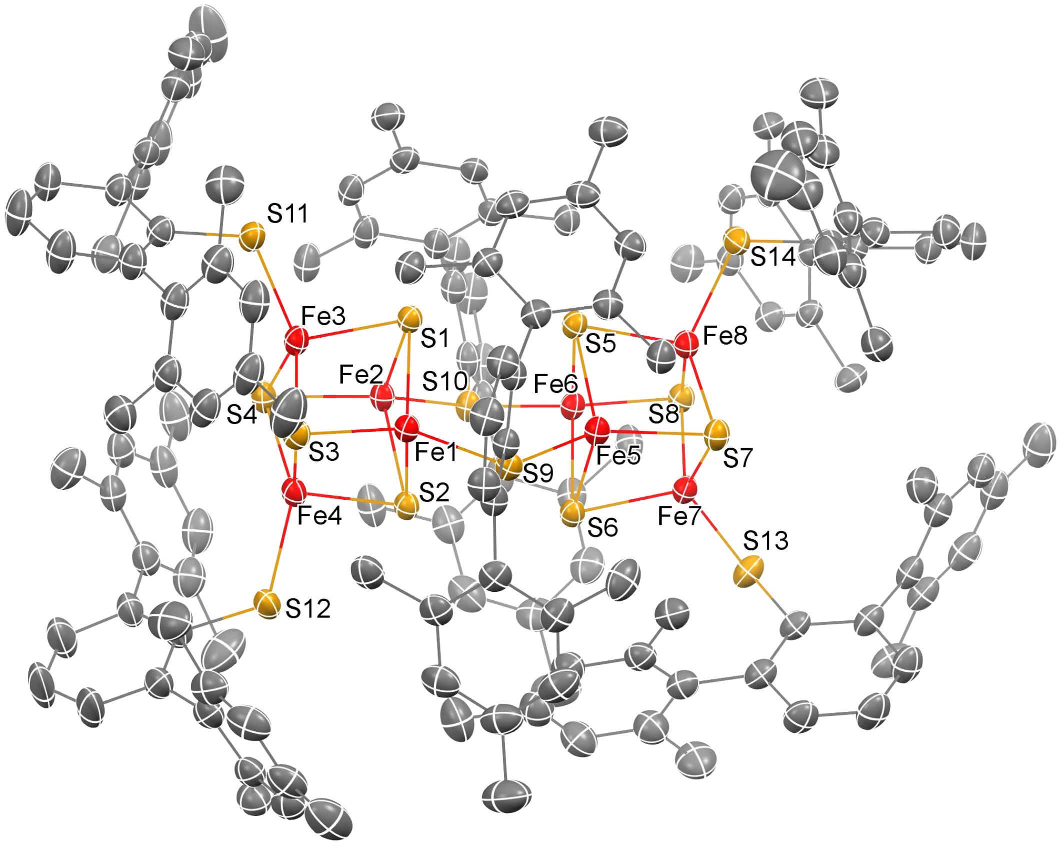

Supplementary Figure 75. Solid-state molecular structure of $[Fe_8S_8(DmpS)_6]$ in crystals of $K_4[Fe_8S_8(DmpS)_6]\cdot3(C_7H_8)$. Displacement ellipsoids are shown at 30% probability. Hydrogen atoms as well as co-crystallized solvent molecules are omitted for clarity.

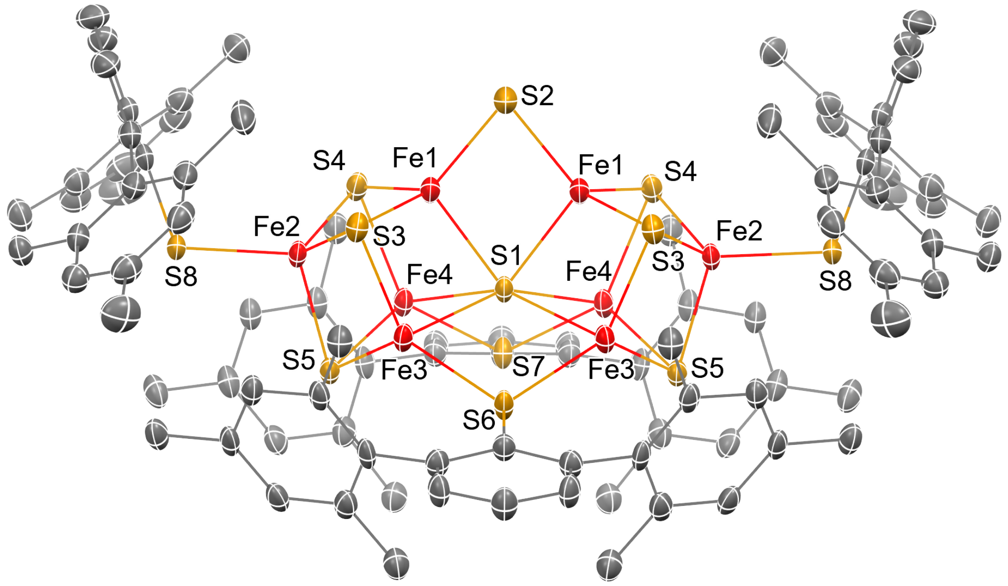

Supplementary Figure 76. Solid-state molecular structure of $[Fe_8S_8(DmpS)_4]$ in crystals of $[Fe_8S_8(DmpS)_4]$. Displacement ellipsoids are shown at 50% probability. Hydrogen atoms are omitted for clarity.

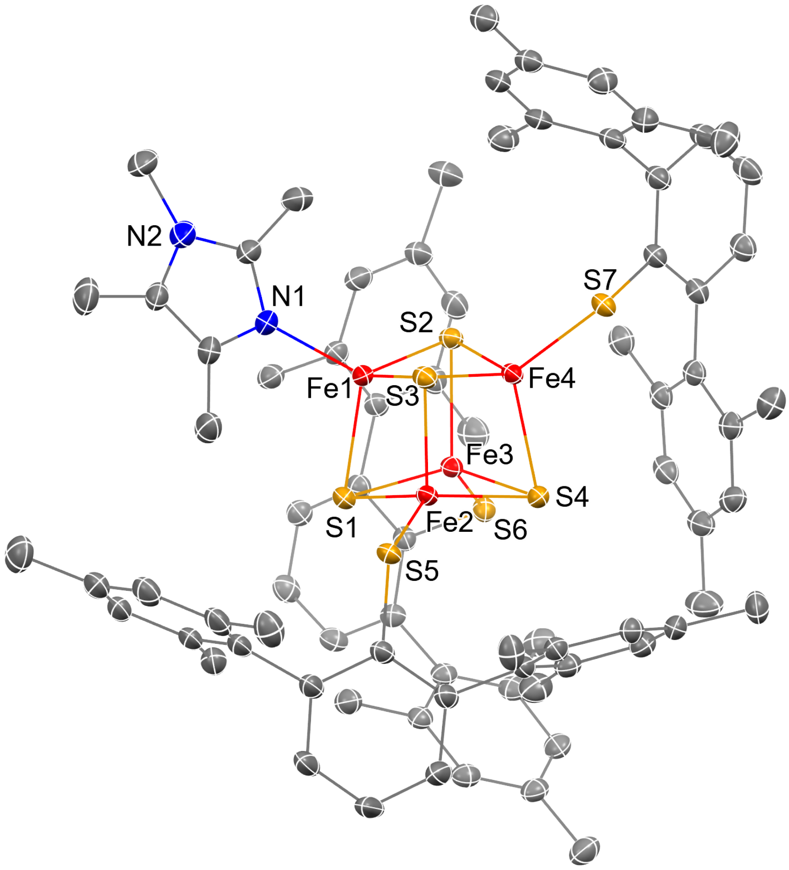

Supplementary Figure 77. Solid-state molecular structure of $[Fe_4S_4(DmpS)_3(Im^*)]$ in crystals of $[Fe_4S_4(DmpS)_3(Im^*)]\cdot(C_7H_8)_{0.5}\cdot(C_6H_{18}Si_2O)_{0.25}$. Thermal displacement ellipsoids are shown at 50% probability. Hydrogen atoms and co-crystallized solvent molecules were omitted for clarity.

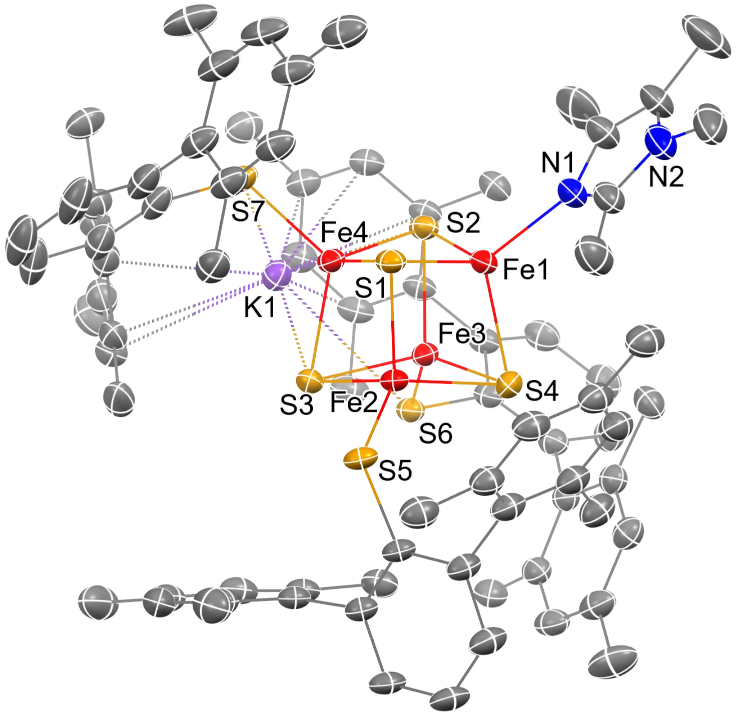

Supplementary Figure 78. Solid-state molecular structure of $K[Fe_4S_4(DmpS)_3(Im^*)]$. Thermal displacement ellipsoids are shown at 50% probability. Hydrogen atoms were omitted for clarity.

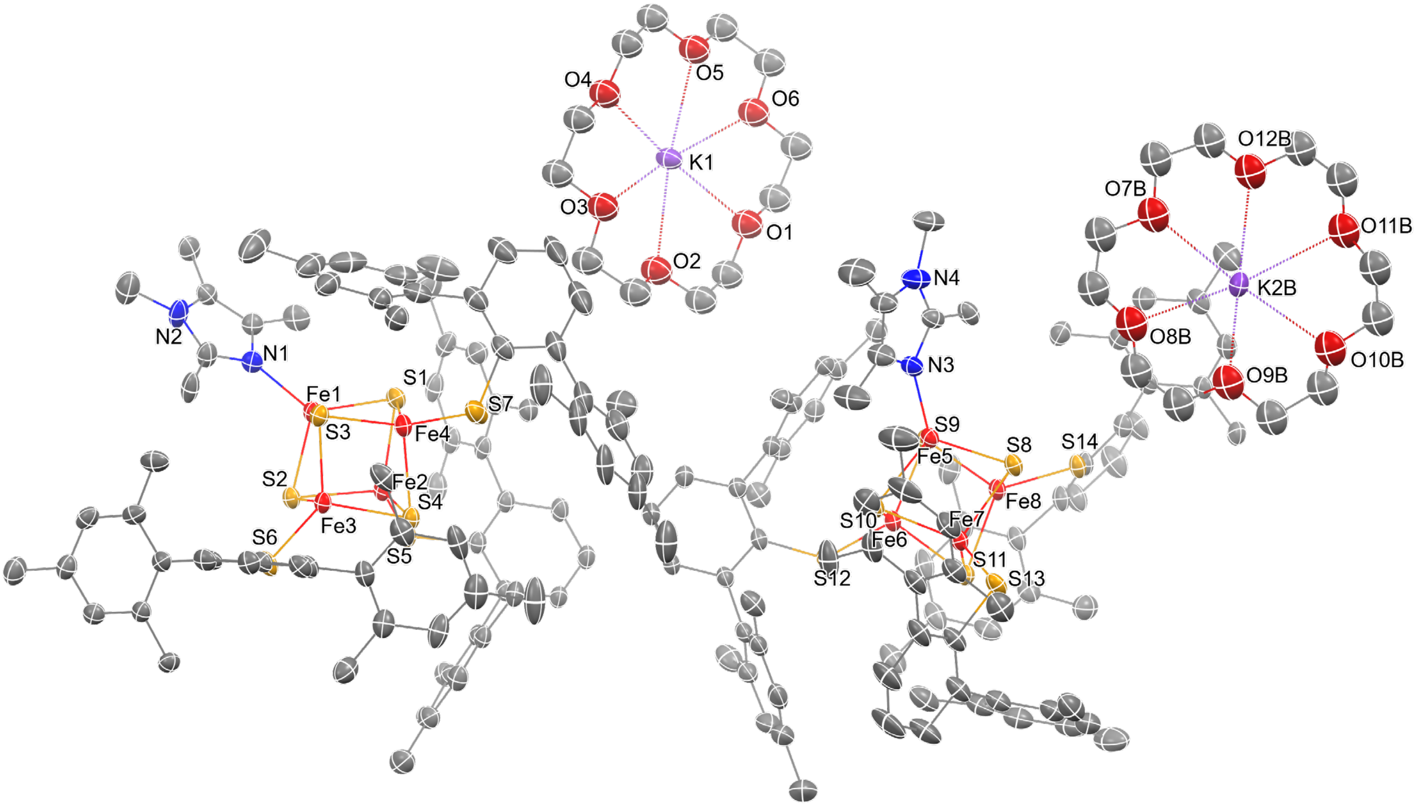

Supplementary Figure 79. Solid-state molecular structure of [18-C-6]$K[Fe_4S_4(DmpS)_3(Im^*)]$ in crystals of 2·[18-C-6]$K[Fe_4S_4(DmpS)_3(Im^*)]$·$2.57(C_7H_8)$·$1.43(C_5H_{12})$. Thermal displacement ellipsoids are shown at 30% probability. Hydrogen atoms, co-crystallized solvent molecules and disorder on one of the [18-C-6]K^+ units were omitted for clarity.

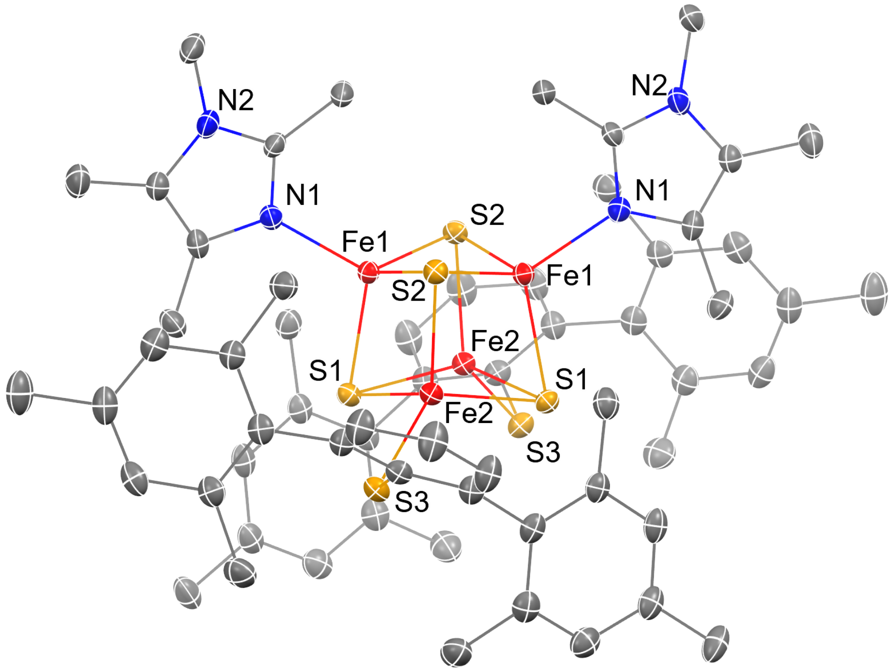

Supplementary Figure 80. Solid-state molecular structure of $[Fe_4S_4(DmpS)_2(Im^*)_2]$ in crystals of $[Fe_4S_4(DmpS)_3(Im^*)]$·(C_4H_8O). Thermal displacement ellipsoids are shown at 50% probability. Hydrogen atoms and co-crystallized solvent molecules were omitted for clarity.

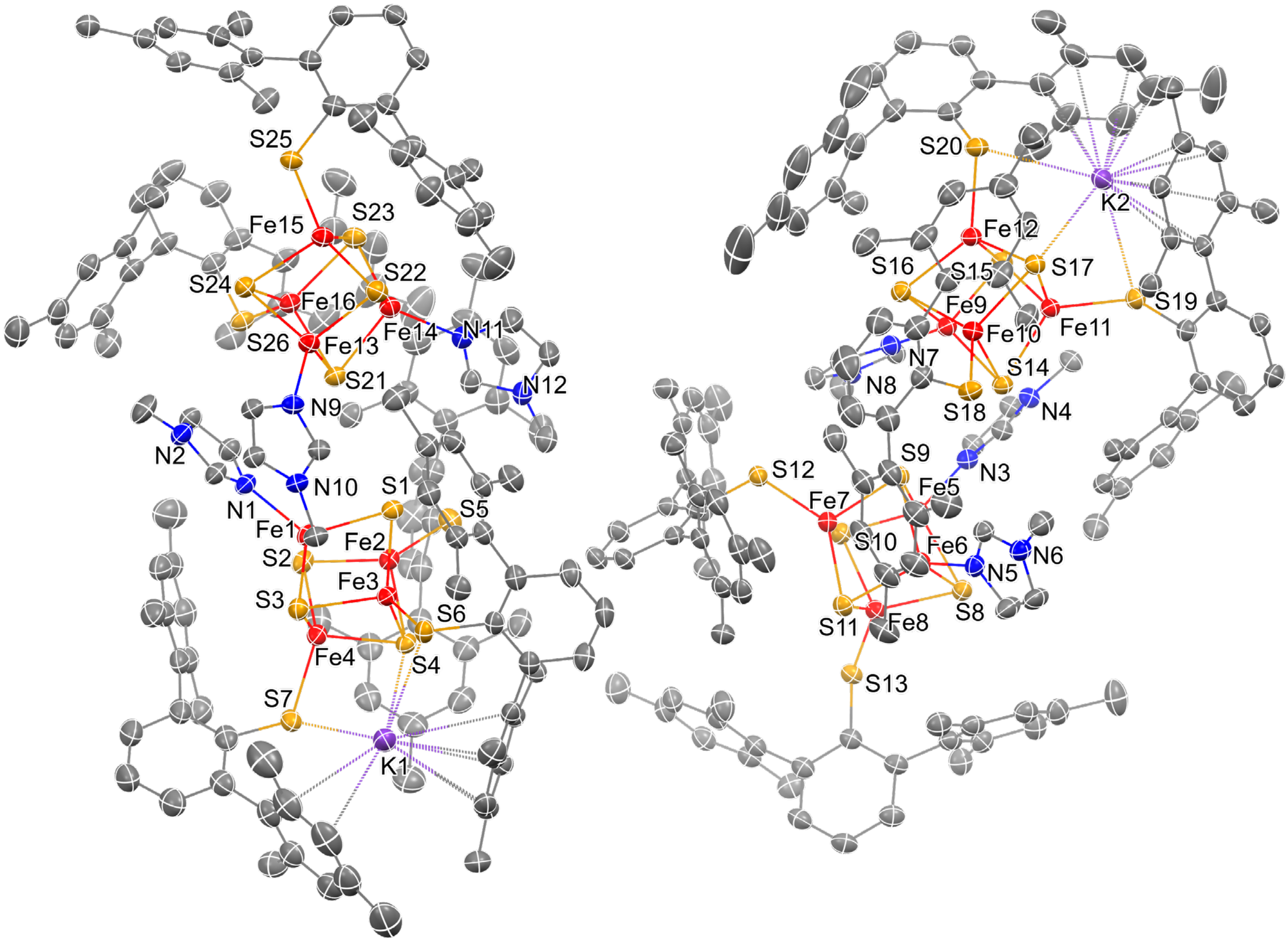

Supplementary Figure 81. Solid-state molecular structures of [$Fe_4S_4(DmpS)_2(Im)_2$] and K[$Fe_4S_4(DmpS)_3(Im)$] in crystals of [$Fe_4S_4(DmpS)_2(Im)_2$]·K[$Fe_4S_4(DmpS)_3(Im)$]·2(C_7H_8). All complexes found in the asymmetric unit are shown. Displacement ellipsoids are displayed at 50% probability; Hydrogen atoms and co-crystallized solvent molecules are omitted for clarity.

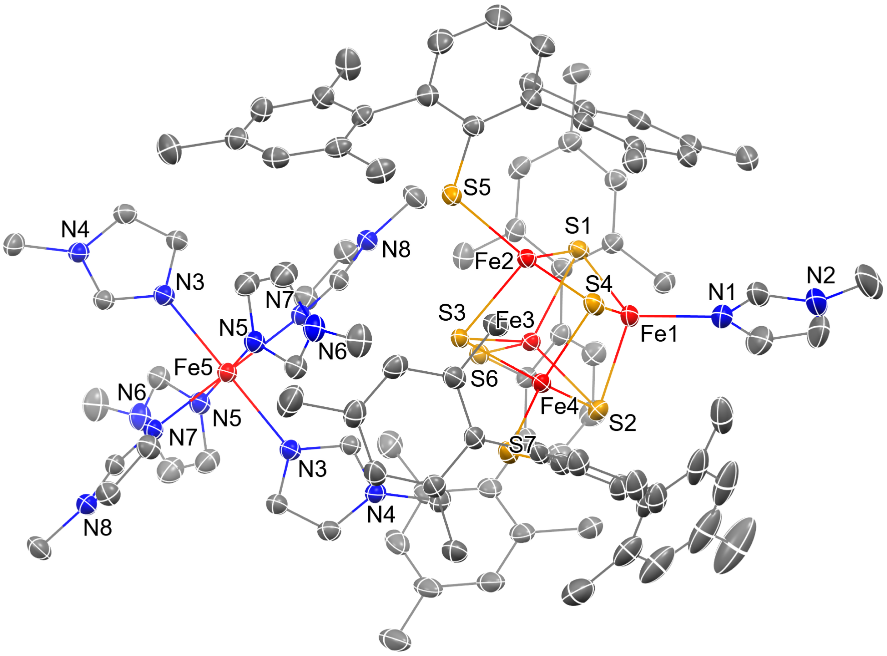

Supplementary Figure 82. Solid-state molecular structure of [$Fe(Im)_6$]$^{2+}$ and [$Fe_4S_4(DmpS)_3(Im)$]$^{-}$ in crystals of [$Fe_4S_4(DmpS)_3(Im)$]·0.5[$Fe(Im)_6$]·3(C_7H_8). Displacement ellipsoids are shown at 50% probability. Hydrogen atoms as well as co-crystallized solvent molecules are omitted for clarity.

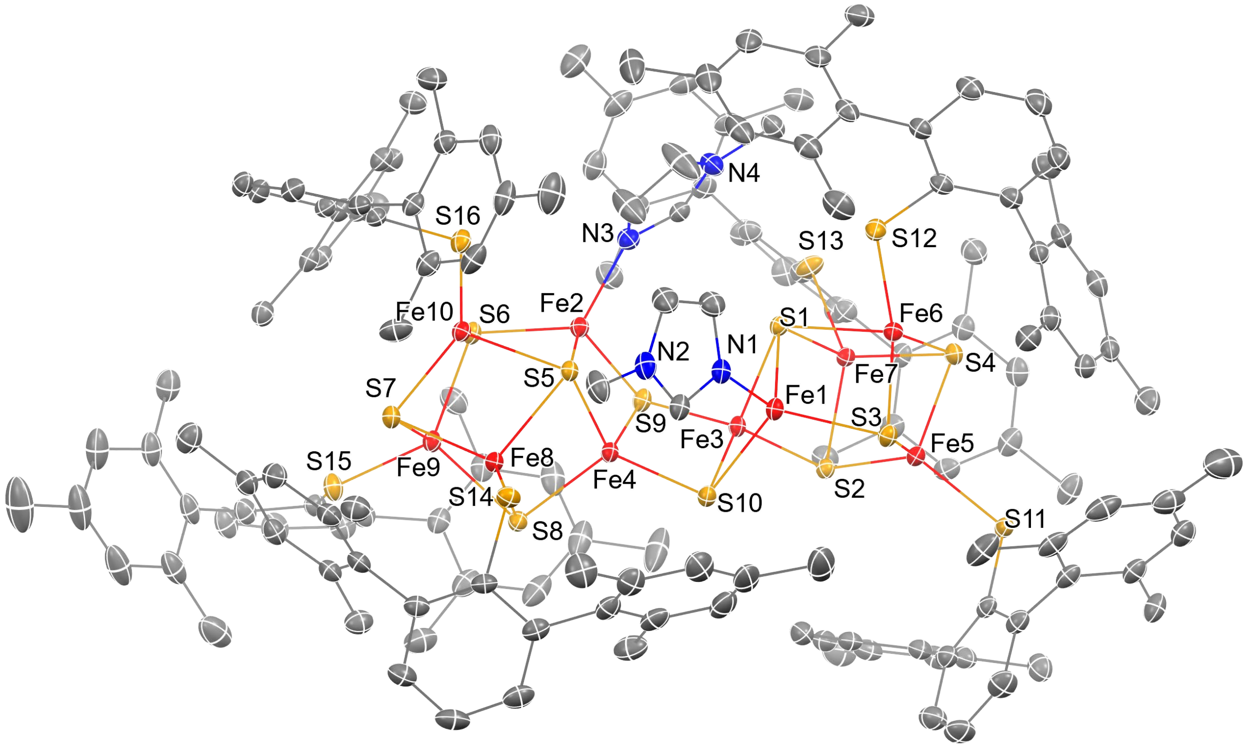

Supplementary Figure 83. Solid-state molecular structure of $[Fe_{10}S_{10}(DmpS)_6(Im)_2]$ in crystals of $[Fe_{10}S_{10}(DmpS)_6(Im)_2]\cdot 3(C_5H_{12})$. Displacement ellipsoids are shown at 50% probability. Hydrogen atoms as well as co-crystallized solvent molecules are omitted for clarity.

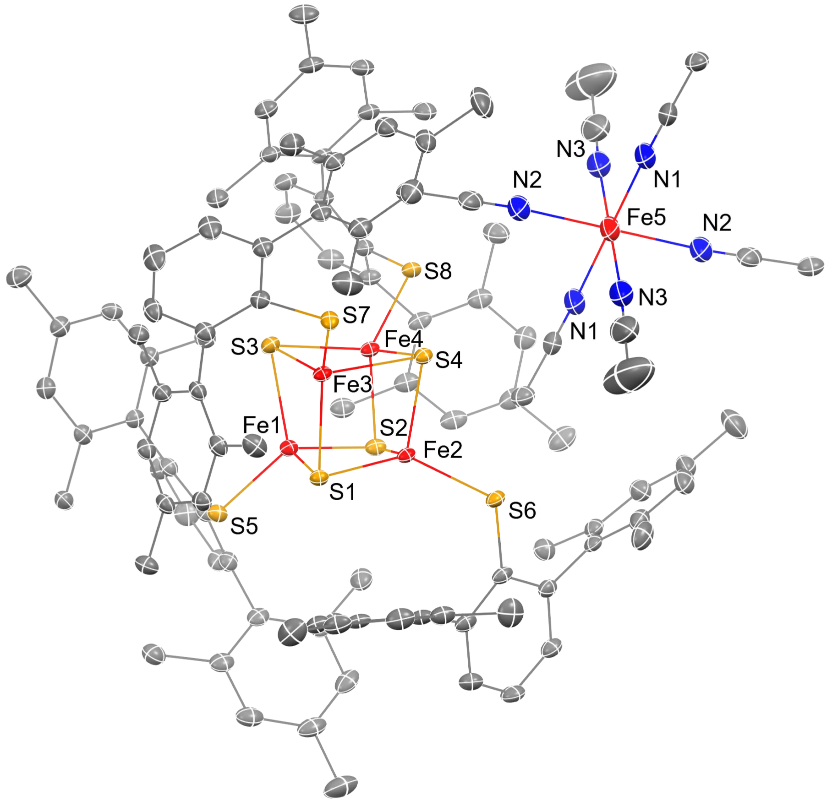

Supplementary Figure 84. Solid-state molecular structure of $[Fe(MeCN)_6]^{2+}$ and $[Fe_4S_4(DmpS)_4]^{-}$ in crystals of $[Fe_4S_4(DmpS)_4]\cdot 0.5[Fe(MeCN)_6]\cdot 4(MeCN)$. Displacement ellipsoids are shown at 50% probability. Hydrogen atoms as well as co-crystallized solvent molecules are omitted for clarity.

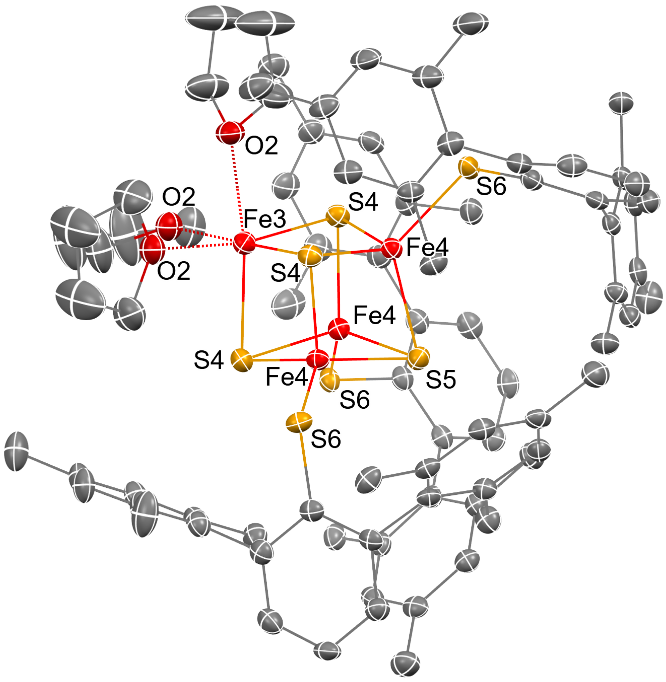

Supplementary Figure 85. Solid-state molecular structure of $[Fe_4S_4(DmpS)_3(THF)_3]$ in crystals of $[Fe_4S_4(DmpS)_3(THF)_3]$. Displacement ellipsoids are shown at 50% probability. Hydrogen atoms as well as co-crystallized solvent molecules are omitted for clarity. The structure is congruent with the reported one for this compound.[26]

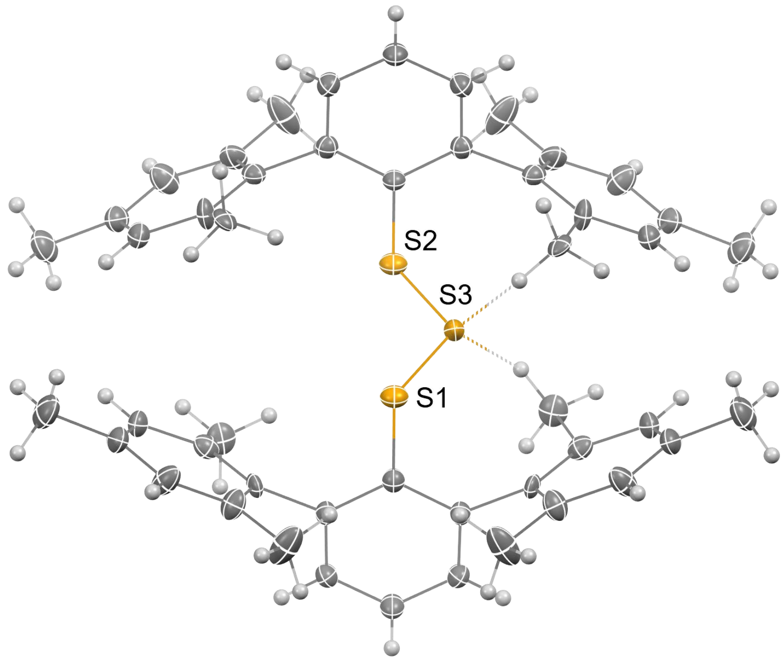

Supplementary Figure 86. Solid-state molecular structure of DmpSSSDmp in crystals of DmpSSSDmp. Displacement ellipsoids are shown at 50% probability. Hydrogen atoms as well as co-crystallized solvent molecules are omitted for clarity.

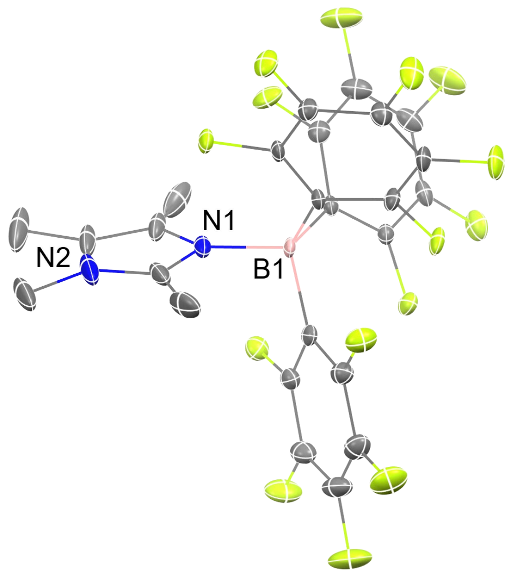

Supplementary Figure 87. Solid-state molecular structure of Im*·$B(C_6F_5)_3$ in crystals of Im*·$B(C_6F_5)_3$. Displacement ellipsoids are shown at 50% probability. Hydrogen atoms as well as co-crystallized solvent molecules are omitted for clarity.

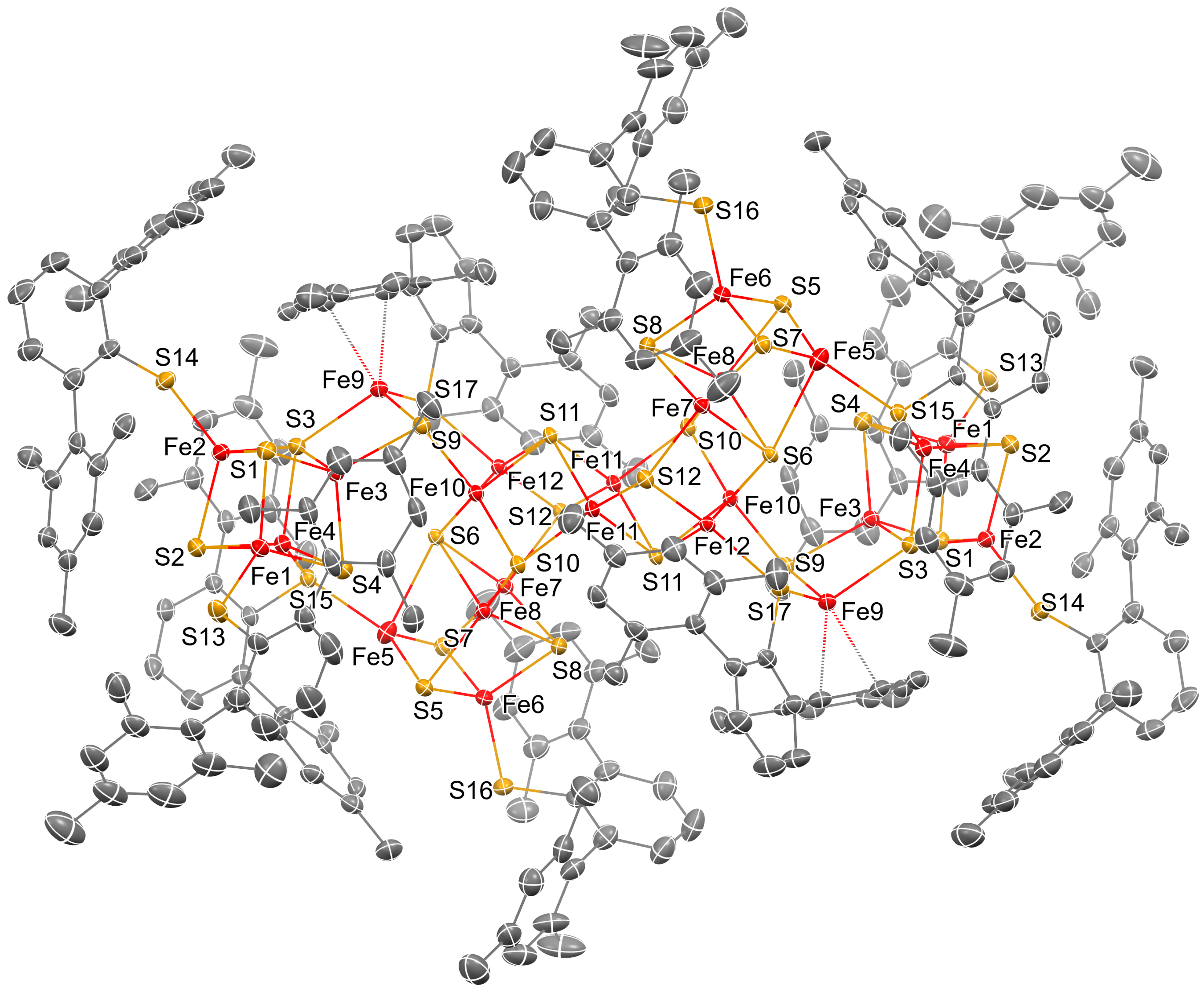

Supplementary Figure 88. Solid-state molecular structure of $[Fe_{24}S_{24}(DmpS)_{10}]$ in crystals of $[Fe_{24}S_{24}(DmpS)_{10}]\cdot 8(C_7H_8)$. Displacement ellipsoids are shown at 50% probability. Hydrogen atoms as well as co-crystallized solvent molecules are omitted for clarity.

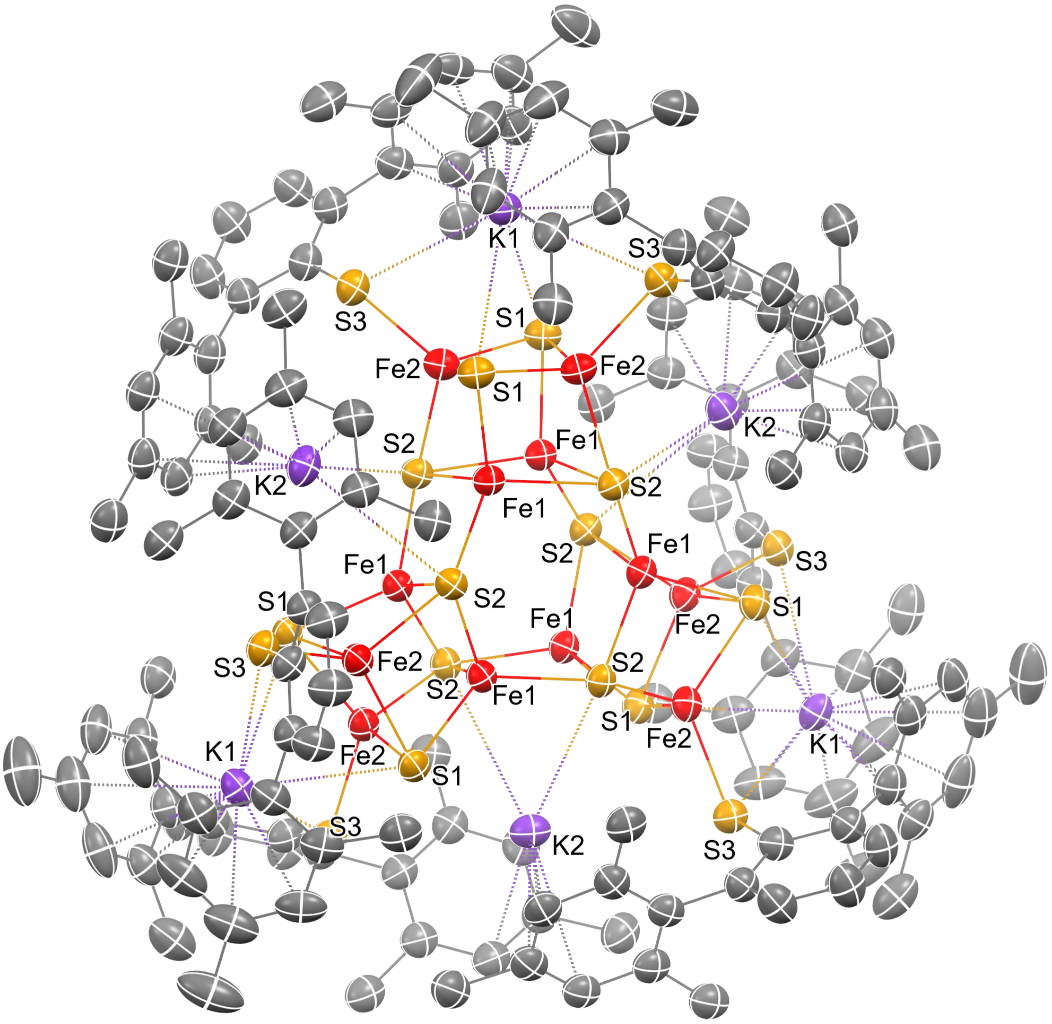

Supplementary Figure 89. Solid-state molecular structure of $K_6[Fe_{12}S_{12}(DmpS)_6]$ in crystals of $K_6[Fe_{12}S_{12}(DmpS)_6]\cdot 2(C_5H_{12})\cdot 3(C_7H_8)$. Displacement ellipsoids are shown at 30% probability. Hydrogen atoms as well as co-crystallized solvent molecules are omitted for clarity.

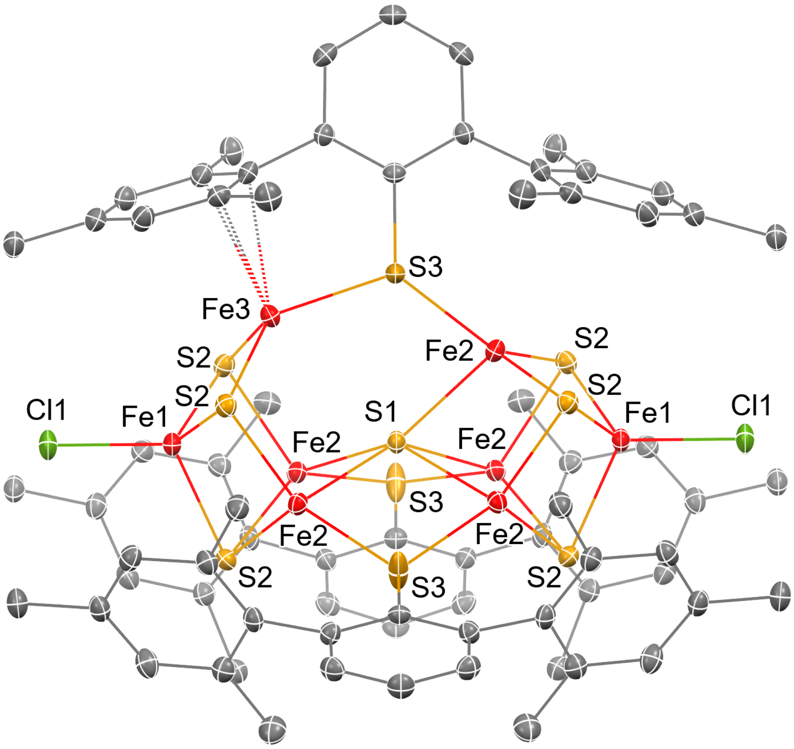

Supplementary Figure 90. Solid-state molecular structure of $[Fe_8S_7(DmpS)_3Cl_2]$ in crystals of $[Fe_8S_7(DmpS)_3Cl_2]$. Displacement ellipsoids are shown at 50% probability. Hydrogen atoms are omitted for clarity.

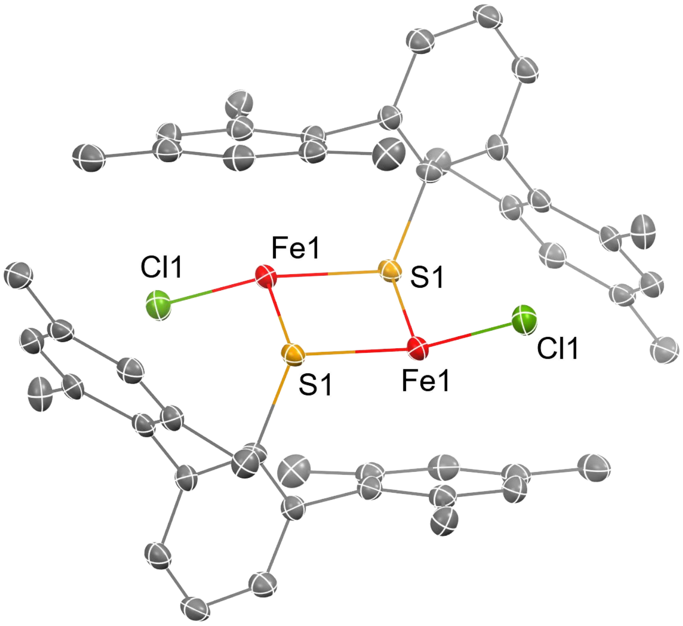

Supplementary Figure 91. Solid-state molecular structure of $[Fe(DmpS)Cl]_2$ in crystals of $[Fe(DmpS)Cl]_2$. Displacement ellipsoids are shown at 50% probability. Hydrogen atoms are omitted for clarity.

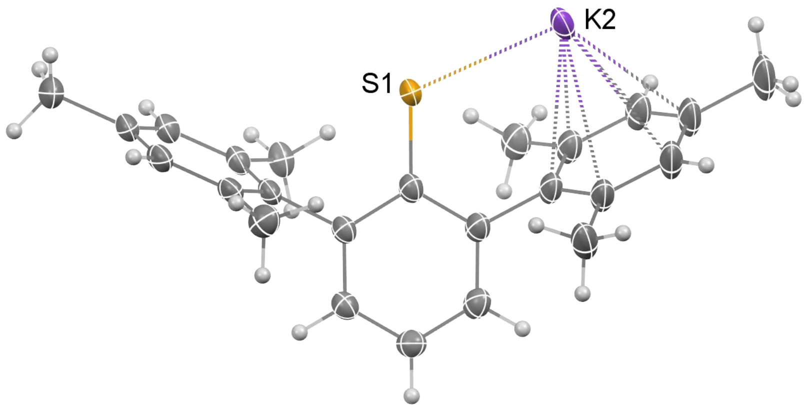

Supplementary Figure 92. Solid-state molecular structure of DmpSK in crystals of DmpSK·$(C_4H_8O)_{0.4}$. Thermal displacement ellipsoids are shown at 50% probability and co-crystallized solvent molecules were omitted for clarity.

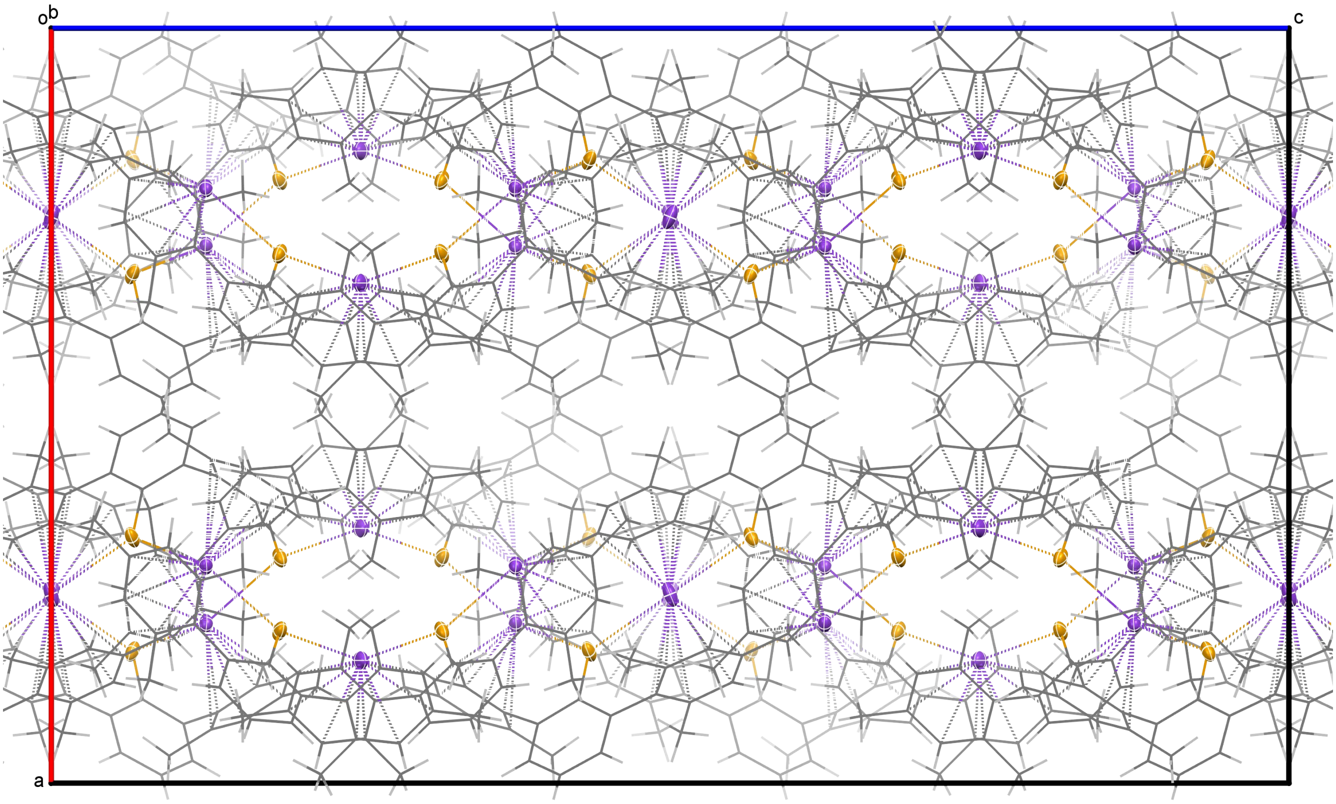

Supplementary Figure 93. Unit cell of DmpSK·$(C_4H_8O)_{0.4}$ showing its polymeric structure. The view is oriented to be along axis *b*. For clarity, displacement ellipsoids are only shown for K (*purple*) and S atoms (*yellow*).

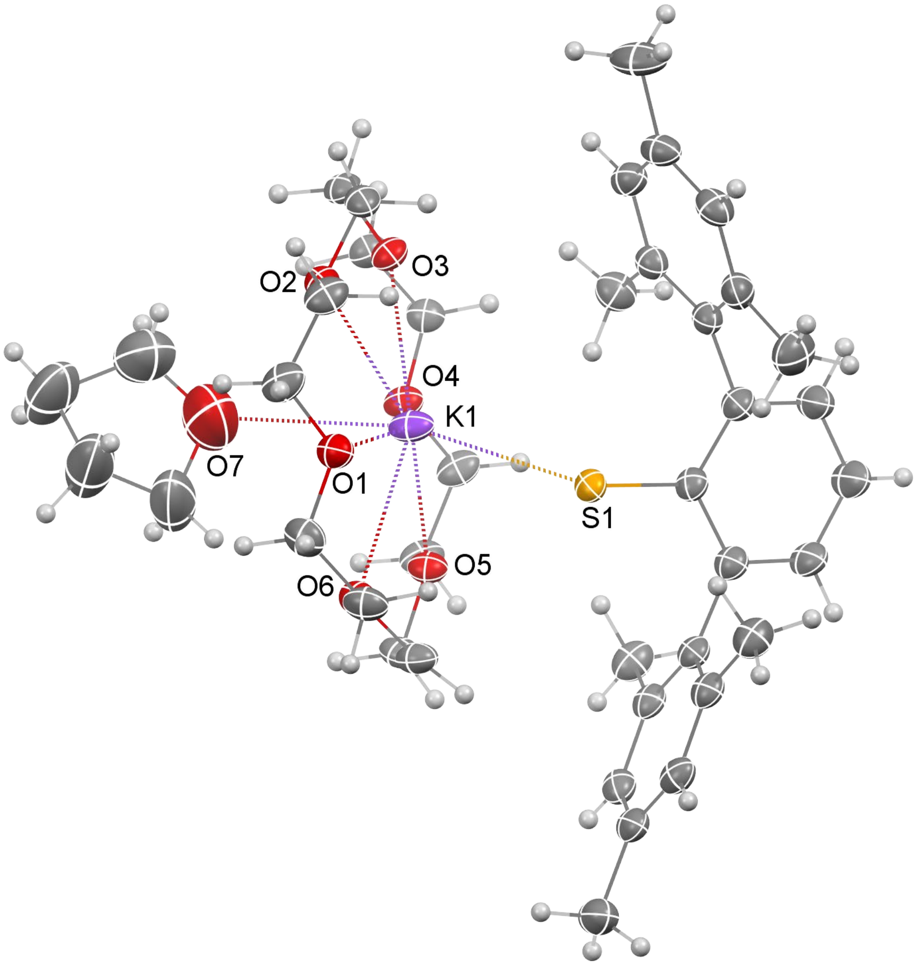

Supplementary Figure 94. Solid-state molecular structure of DmpS[18-C-6]K in crystals of DmpS[18-C-6]K·(C_4H_8O). Thermal displacement ellipsoids are shown at 50% probability and disorder on the solvent molecule were omitted for clarity.

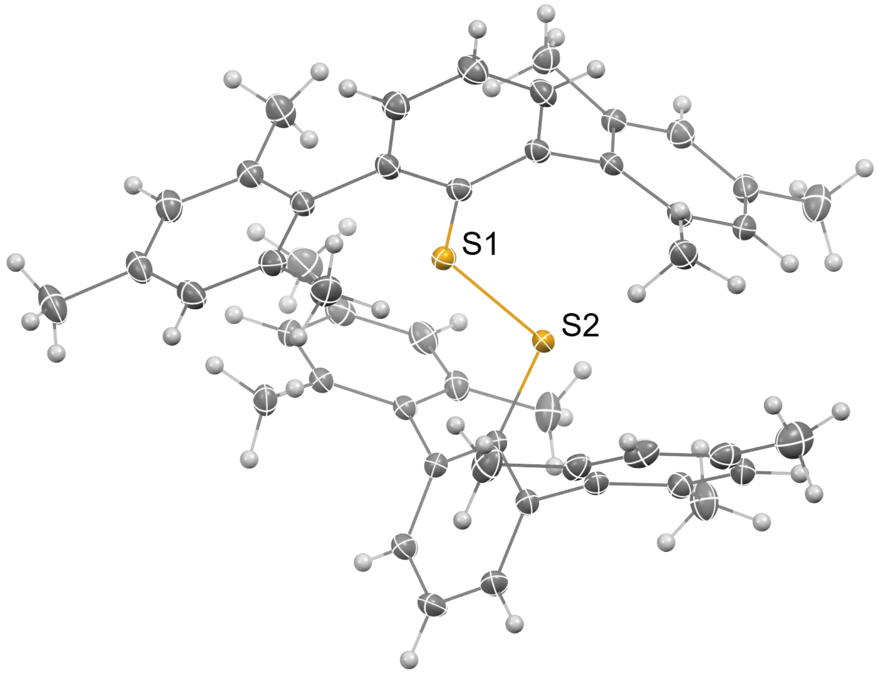

Supplementary Figure 95. Solid-state molecular structure of $(DmpS)_2$. Thermal displacement ellipsoids are shown at 50% probability.

Structural figures for supporting discussion

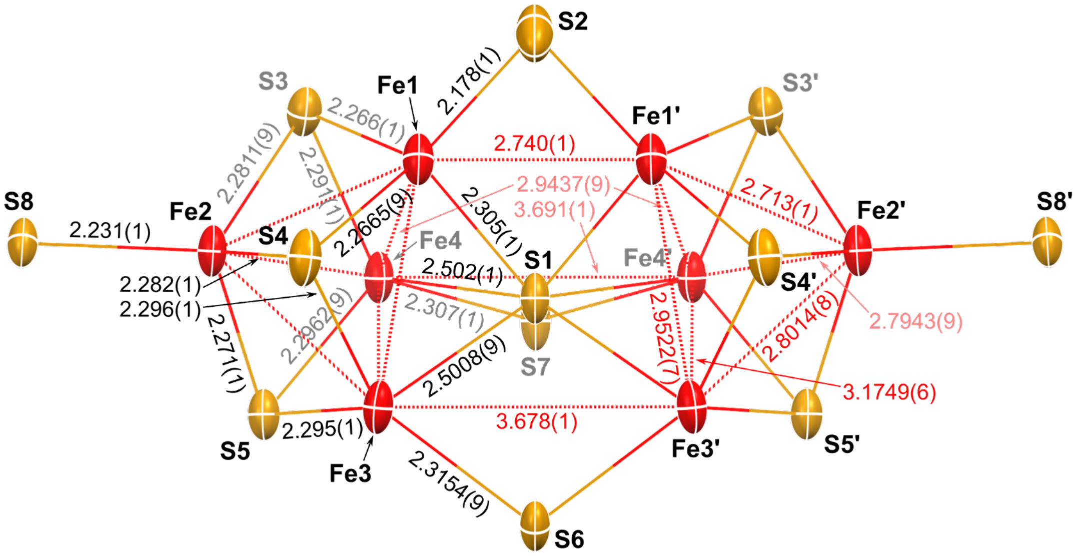

Supplementary Figure 96. (A) Solid-state molecular structure of the FeS core of [Fe_8S_8(DmpS)$_4$] with displacement ellipsoids shown at 50% probability. Selected bond lengths/atomic distances are given in Å.

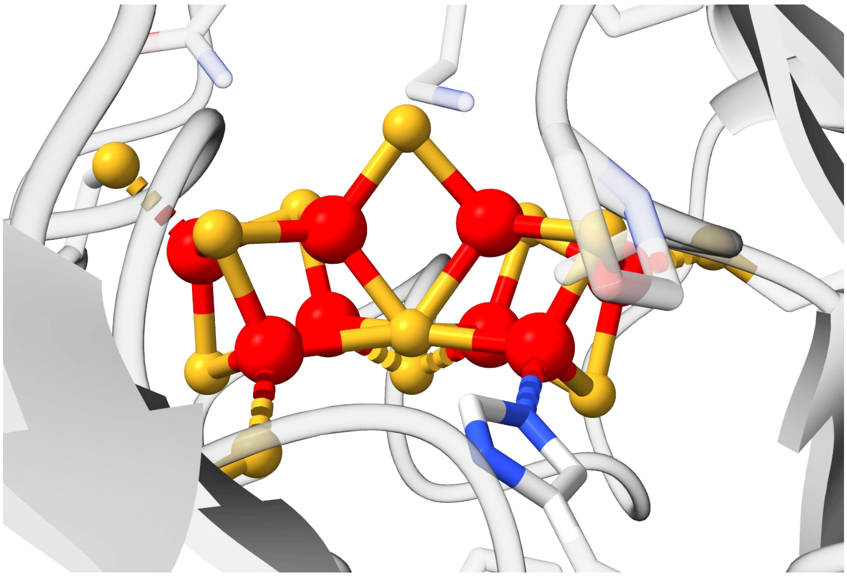

Supplementary Figure 97. Close-up view of the K-cluster in the crystal structure refinement of *Methanobacterium thermoautotrophicum* NifB with a full complement of FeS cofactors, as proposed by the group of Nicolet (PDB code 7BI7).[43] The view of the cofactor given here is opposite to the one shown in Fig. 1D of the main text. This image was generated using the ChimeraX program suite.[44,45]

Supplementary ^{57}Fe Mössbauer Spectra and Simulations

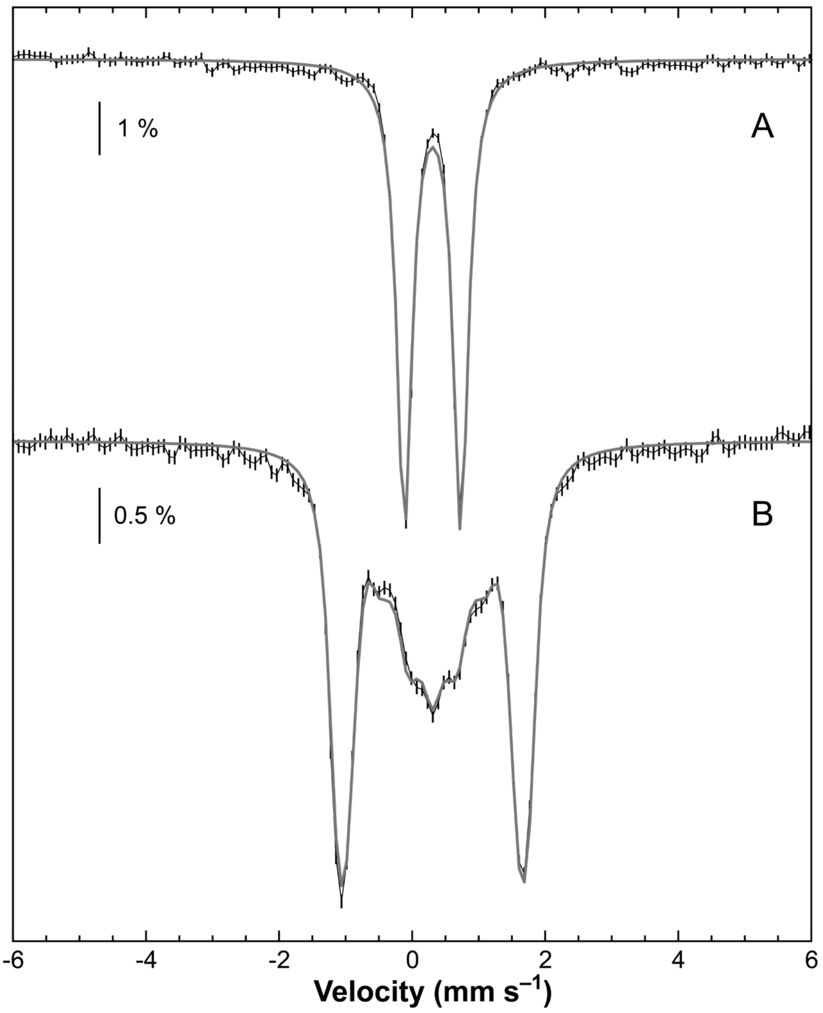

Supplementary Figure 98. Experimental 5.3 K Mössbauer spectra recorded on a powder sample of [$Fe_2S_2(DmpS)_2(py)_2$] using a 0.06 (A) and 7 T (B) external magnetic field applied parallel to the γ-beam. Simulated spectra are overlaid as *grey solid lines*. They were obtained assuming a unique diamagnetic iron site. The following nuclear parameters were determined: δ = 0.31 mm s^{-1}, ΔE_Q = 0.85 mm s^{-1}, η = 1.0, Γ_{fwhm} = 0.27 mm s^{-1} (A) and Γ_{fwhm} = 0.28 mm s^{-1} (B).

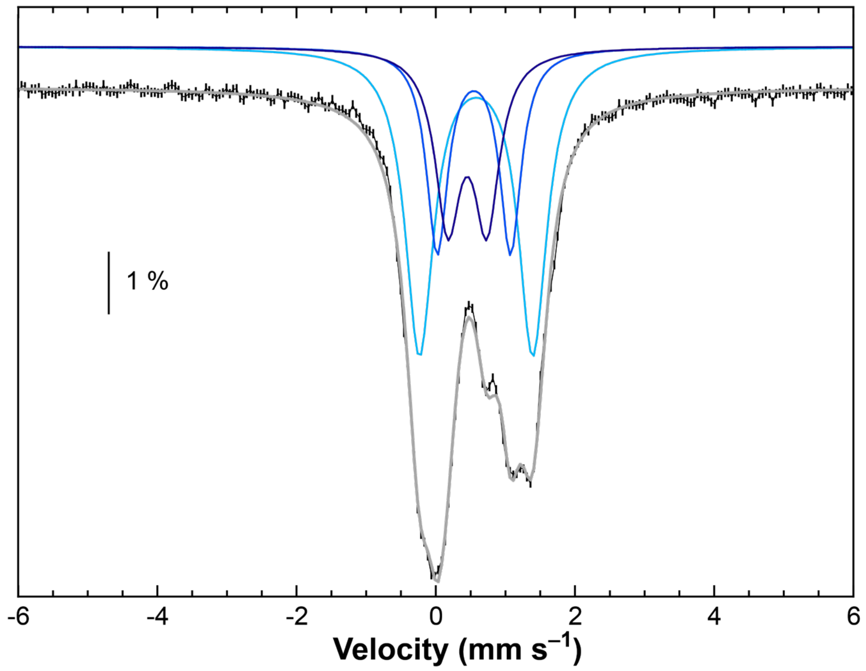

Supplementary Figure 99. Experimental (*black vertical bars*) and simulated (*grey solid line*) zero-field spectrum of a toluene solution sample of $K_4[^{57}Fe_8S_8(DmpS)_6]$ recorded at 82 K. Owing to the three resolved lines evidenced above 0.5 mm s^{-1}, simulations were performed considering three iron sites in a 2:1:1 ratio. One satisfying result is overlaid as *solid grey line* and contributions are displayed above as *solid color traces*. The obtained parameters are: Doublet 1 (*light blue*): δ = 0.58 mm s^{-1}, ΔE_Q = 1.63 mm s^{-1}, Γ_{fwhm} = 0.49 mm s^{-1}; Doublet 2 (*medium blue*): δ = 0.55 mm s^{-1}, ΔE_Q = 1.05 mm s^{-1}, Γ_{fwhm} = 0.37 mm s^{-1};. Doublet 3 (*dark blue*): δ = 0.45 mm s^{-1}, ΔE_Q = 0.56 mm s^{-1}, Γ_{fwhm} = 0.43 mm s^{-1}. The average isomer shift is 0.54 mm s^{-1} is in full consistency with the value obtained on the powder sample (0.53 mm s^{-1}).

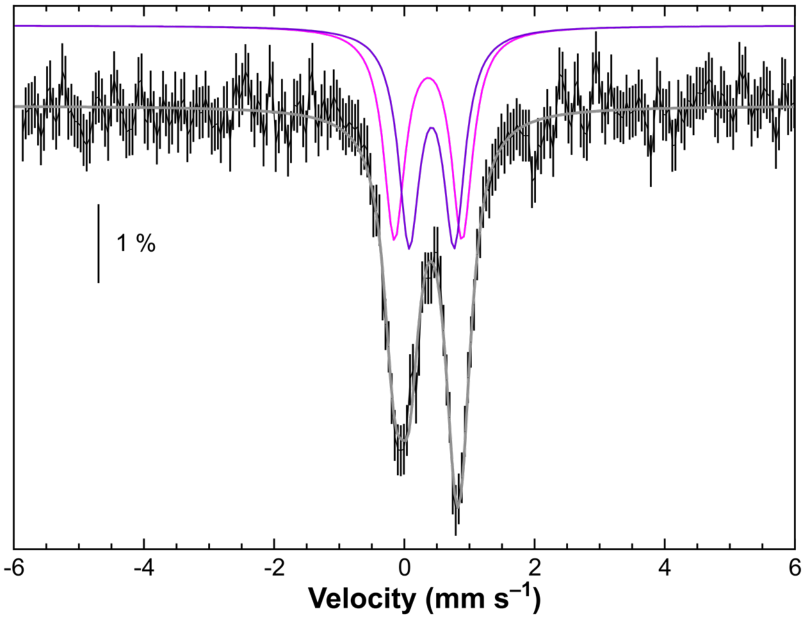

Supplementary Figure 100. Experimental 80 K Mössbauer spectrum (*vertical bars*) recorded on a powder sample of [Fe_8S_8(DmpS)$_6$] using a 0.06 T external magnetic field applied parallel to the γ-beam. The simulated spectrum is overlaid as a *grey solid line* (same as in Fig. 5C). The two 1:1 nested doublets are drawn as solid thin *violet* and *magenta lines* above the spectrum. The following nuclear parameters were determined: Doublet 1 (*magenta*): δ = 0.37 mm s^{-1}, ΔE_Q = 1.04 mm s^{-1}; Doublet 2 (*violet*): δ = 0.42 mm s^{-1}, ΔE_Q = 0.69 mm s^{-1}; Common linewidth: Γ_{fwhm} = 0.39 mm s^{-1}.

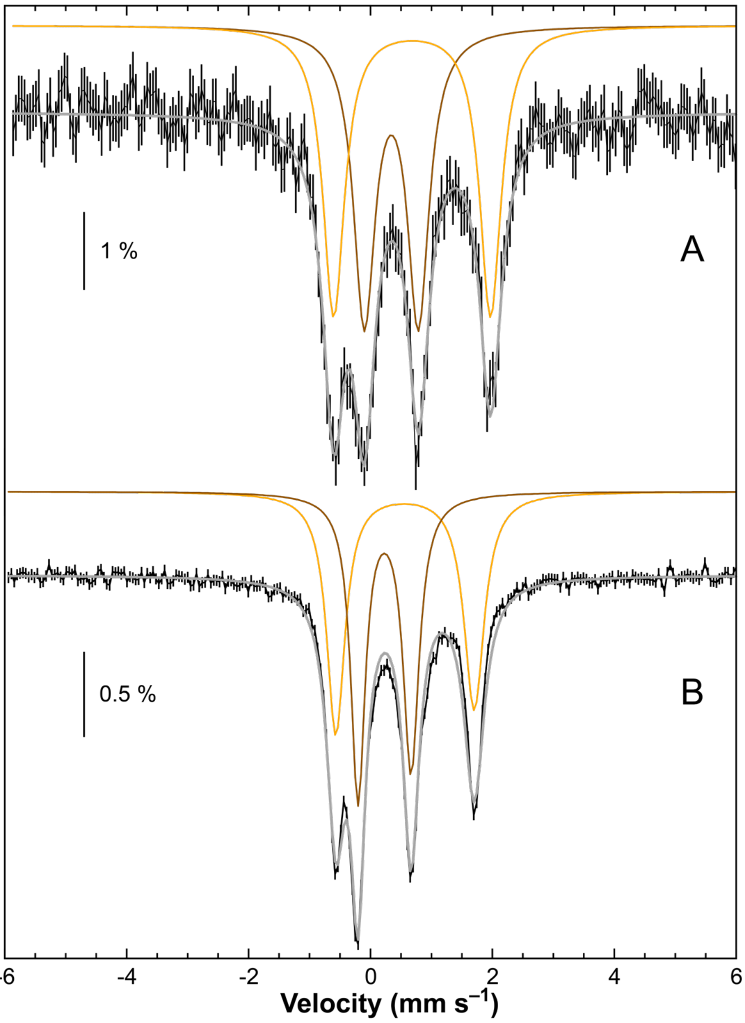

Supplementary Figure 101. Experimental (*black vertical bars*) and simulated (*grey solid lines*) zero-field spectra of a powder sample of [Fe_8S_8(DmpS)$_4$] at 80 K (A; same as in Fig. 5D) and 293 K (B). The contributions in a 1:1 ratio are displayed as colored traces above the spectra (ferrous and ferric sites in *yellow* and *brown*, respectively). Parameters are listed in Supplementary Table 11.

Supplementary Table 11. Nuclear parameters associated to the two equally contributing doublets considered to reproduce the powder spectra of [Fe_8S_8(DmpS)$_4$] displayed in Supplementary Fig. 101. A common linewidth was considered for the 80 K spectrum whereas four different linewidths were considered for the room temperature spectrum.

T (K)	site	valence	δ (mm s^{-1})	ΔE_Q (mm s^{-1})	Γ_{fwhm} (mm s^{-1})	area (%)
80	1	III	0.34	0.89	0.43	51
	2	II	0.68	2.57	0.43	
293	1	III	0.23	0.87	0.29/0.32	51
	2	II	0.57	2.27	0.36/0.40	

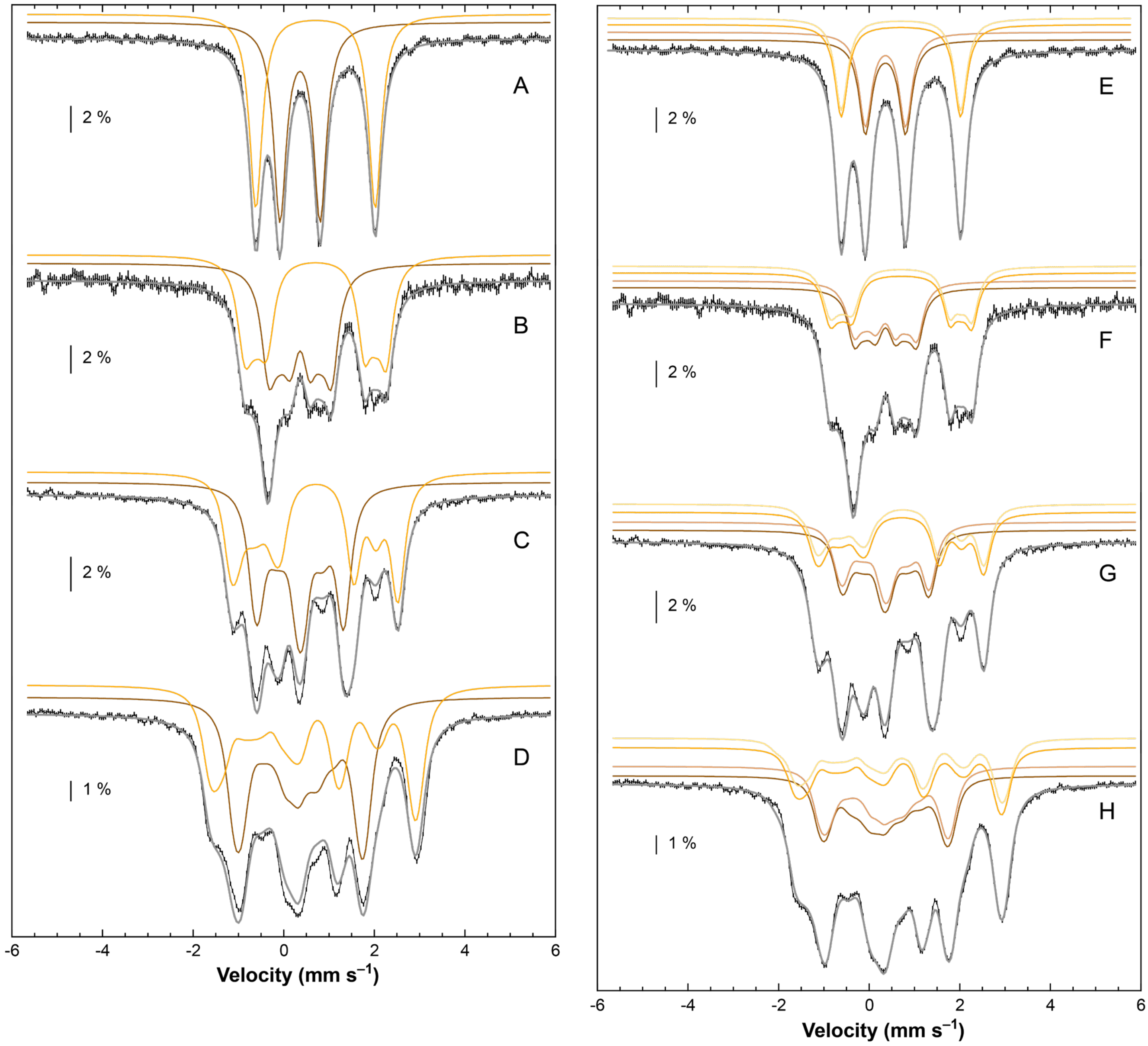

Supplementary Figure 102. Experimental 5.7 K Mössbauer spectrum (*black vertical bars*) recorded on a powder sample of [Fe_8S_8(DmpS)$_4$] using a 0.06 (A,E), 2 (B,F), 4 (C,G) and 7 T (D,H) external magnetic field applied parallel to the γ-beam. The simulated spectrum is overlaid as *thick grey solid lines*. They were obtained considering (A-D) two equally contributing diamagnetic sites, or (E-H) four equally contributing Fe sites with 1/2-electronic spin, respectively, their contributions being displayed above the spectra as *colored thin solid lines*. The isomer shift and quadrupole splitting values were fixed to those determined upon considering two diamagnetic sites (as shown in panel A). Nuclear parameters: (A-D) Site 1 (*brown*): δ = 0.36(1) mm s^{-1}, ΔE_Q = –0.89(3) mm s^{-1}, η = 0.8(1); Site 2 (*yellow*): δ = 0.70(1) mm s^{-1}, ΔE_Q = –2.63(3) mm s^{-1}, η = 0.5(1); Common linewidth: Γ_{fwhm} = 0.34 mm s^{-1} at 0.06, 2 and 4 T, Γ_{fwhm} = 0.36 mm s^{-1} at 7 T. (G-H) Site 1 (*dark brown*): δ = 0.36(1) mm s^{-1}, ΔE_Q = –0.89(3) mm s^{-1}, η = 0.9(1), $A_{iso}/(g_n\mu_n)$ = –24(2) T; Site 2 (*light brown*): δ = 0.36(1) mm s^{-1}, ΔE_Q = –0.89(3) mm s^{-1}, η = 0.8(1), $A_{iso}/(g_n\mu_n)$ = –17(2) T; Site 3 (*yellow*): δ = 0.70(1) mm s^{-1}, ΔE_Q = –2.63(3) mm s^{-1} , η = 0.6(1), $A_{iso}/(g_n\mu_n)$ = 2(2) T; Site 4 (*light yellow*): δ = 0.70(1) mm s^{-1}, ΔE_Q = –2.63(3) mm s^{-1}, η = 0.5(1), $A_{iso}/(g_n\mu_n)$ = 6(3) T; Principal values of the anisotropic exchange interaction (principal axes identical to those of the four EFG tensors): 18(5) / 33(5) / –2(5) cm^{-1}. These values led to J_{iso} = 16(5) cm^{-1} and ZFS parameters of the excited S=1 state D = –14(3) cm^{-1} and E/D = 0.3(1). Linewidth: Γ_{fwhm} = 0.34 mm s^{-1} at 0.06 T, Γ_{fwhm} = 0.32 mm s^{-1} at 2, 4 and 7 T.

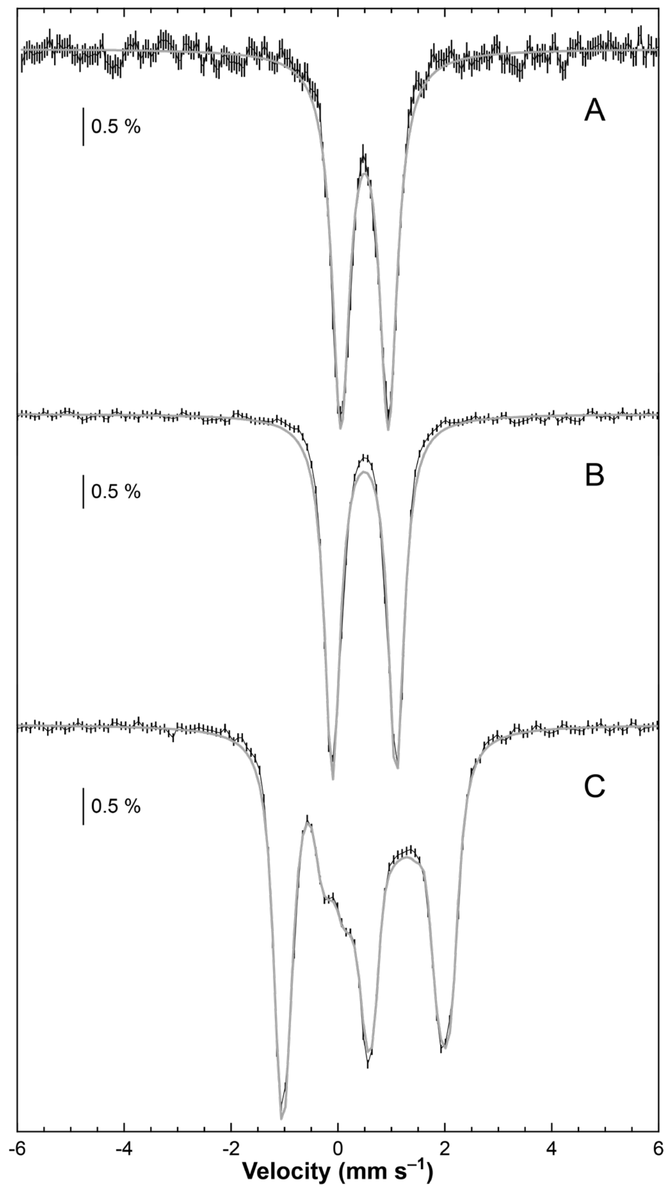

Supplementary Figure 103. Mössbauer powder spectra (*black vertical bars*) of [2.2.2]$K_2[Fe_4S_4(DmpS)_4]$. Recording conditions: Panel A: T=80 K and B=0.06 T; Panel B: T=5.8 K and B=0.06 T; Panel C: T=5.8 K and B=7 T. The external magnetic field, B, was applied parallel to the γ-rays. Simulations are overlaid as *grey solid thick lines*. A single diamagnetic Fe site was assumed. Simulation Parameters are δ = 0.49 (0.50) mm s^{-1}, ΔE_Q = 1.19 (0.89) mm s^{-1}, η = 0.5 (n.d.), Γ_{fwhm} = 0.35 (0.40) mm s^{-1}, and the relative areas amounted to 1.01 (1.02); The values determined at 80 K are given in brackets.

Density Functional Theory Calculations for *ildc*

Density Functional Theory (DFT) calculations on ***ildc*** were carried out using the ORCA v. 6.0.1,[46] program package as implemented on ETH Zürich's Euler cluster.

In a first step, we carried out broken-symmetry[47,48] (BS) geometry optimizations of the complete structure (216 atoms) at the BP86 level of theory,[49,50] using the experimental solid-state geometry determined by single-crystal XRD as starting point. To keep the computational time at a reasonable duration, the def2-SVP basis set was used for C and H atoms, whereas the def2-TZVP was used for the Fe and S atoms,[51,52] while the RI approximation in conjunction with the corresponding def2/J auxiliary basis set was implemented.[53] A dispersion correction was not used, as the latter was shown to negatively influence the geometric parameters of optimized structures for FeS clusters of nuclearity 4 or higher, as well as being detrimental to the results of frequency calculations on these systems.[54] The "FlipSpin" procedure in ORCA converges a high-spin solution, localizes the orbitals and then converges to a broken-symmetry solution. Because of this, and because ***ildc*** has a singlet (S=0) ground state, it is not guaranteed that the exact "input" FlipSpin broken-symmetry solution will be reproduced in the final wavefunction—ORCA localizes electrons after generating the high-spin solution and thus re-distributes them in the broken-symmetry solution.

Accordingly, to capture as much "unique" broken-symmetry solutions as possible, and to avoid computational biases, geometry optimizations with tighter-than-default convergence criteria ("tightopt") were carried out using the total of

$$\binom{8}{4} = 70$$

FlipSpin possibilities as the starting points. The 70 converged simulations are numbered sequentially, BS1-BS70. All Fe sites were assumed to be in their respective high-spin states (4 Fe sites with S=5/2 and 4 sites with S=2; resulting in a total of 36 unpaired electrons in the high-spin solutions). This yielded 12 unique broken-symmetry solutions, many of which are degenerate, and are thus clustered around their respective final single-point energies. The latter are indicated as grey lines in Supplementary Fig. 106A and are referenced against the energetically lowest solution. Exemplarily for each unique spin-topology (highlighted as green markers in Supplementary Fig. 106A), one of the spin-density plots is shown alongside, in panel B of Supplementary Fig. 106. All 70 optimized geometries reproduce the geometrical structure of ***ildc*** fairly (Supplementary Fig. 104). Notably, all solutions were confirmed to be "true" broken-symmetry solutions by inspection/monitoring of the value of $\langle S^2 \rangle$; being non-zero in all cases.

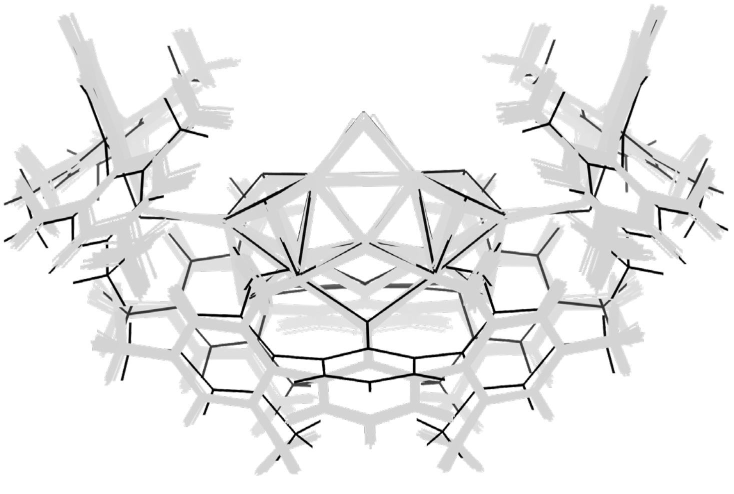

Supplementary Figure 104. Overlay of the solid-state molecular structure of ***ildc*** (*black*) with the 70 DFT optimized geometries (*grey*).

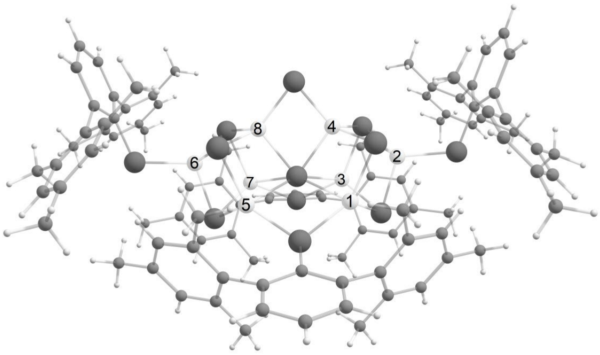

Supplementary Figure 105. Molecular structure of ***ildc*** with the numbering scheme of the Fe atoms, as used throughout the computational analyses.

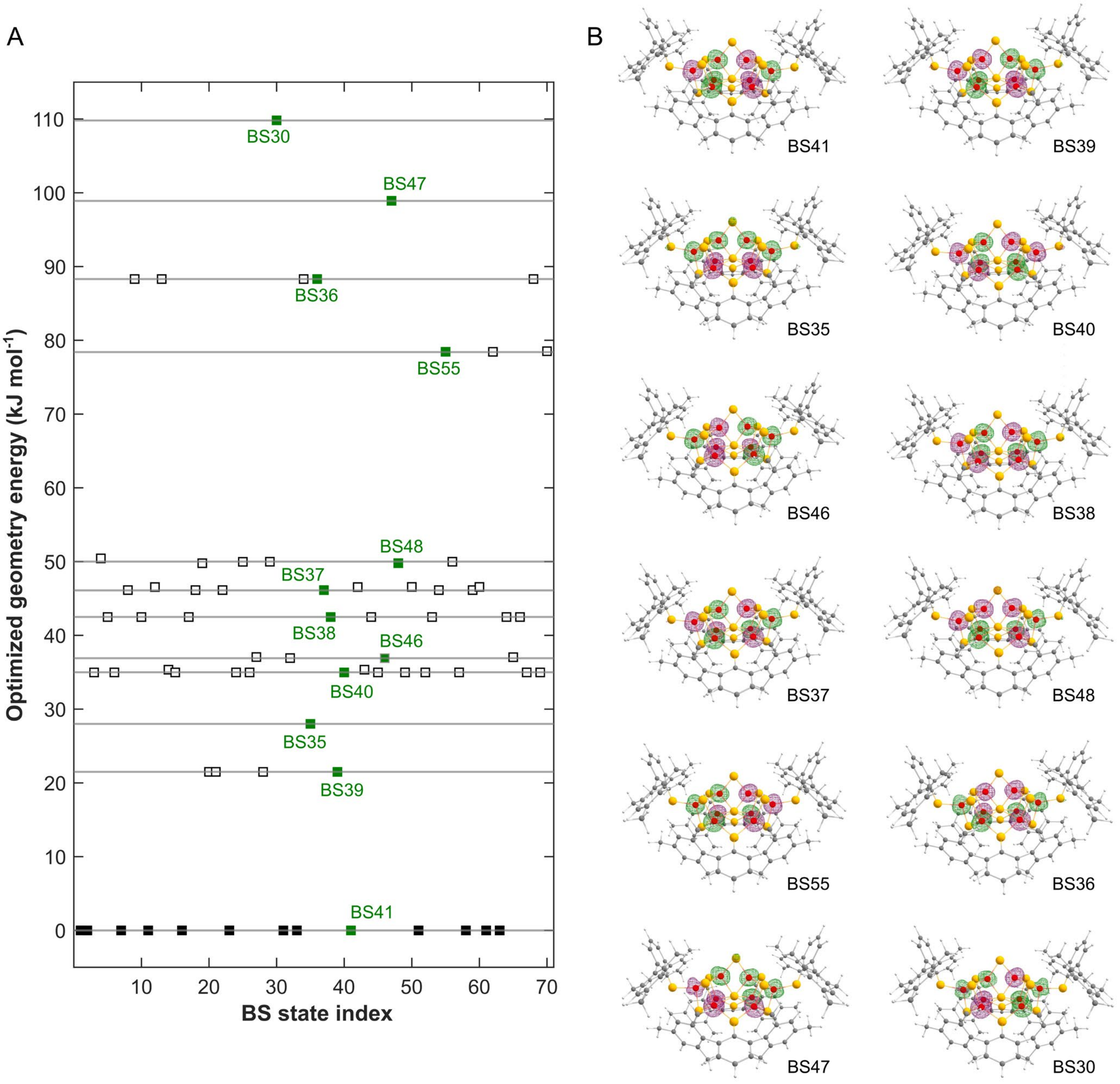

Supplementary Figure 106. (A) Energetic ordering of the 70 converged geometry optimized BS solutions for the spin topology of ***ildc***. The lowest-energy solutions (equivalent) are marked in black, while selected unique BS solutions are marked in green. The respective spin-densities for the 12 identified unique spin topologies are summarized in panel (B).

The optimized geometries were subsequently utilized for single-point calculations of the Fe sites' Mössbauer parameters (specifying *via* the %eprnmr input block: "nuclei = all Fe {fgrad, rho}"). For this, the same level of theory as for the geometry optimization was used, save for the basis set of the Fe atoms, for which the specialized basis set CP(PPP)[55] was chosen. Furthermore, the calculation was performed without the use of auxiliary basis sets ("NORI"), tighter-than-default grid settings were enforced ("DEFGRID3") and the integration accuracy for the Fe atoms was increased in the %methods input-block ("SpecialGridAtoms 26", "SpecialGridIntAcc 7").
While the Mössbauer quadrupole splittings are available directly from the ORCA output, the isomer shifts, δ, were calculated empirically from the electron density at the nucleus (ρ), using the appropriate calibration curve for the employed method according to:

$$\delta = \alpha(\rho - C) + \beta$$

whereby, in our case (BP86 functional combined with the CP(PPP) basis set), α=–0.425, β=7.916 and C=11810.[56]
Inspection of the resulting Mössbauer parameters of the lowest-energy BS solutions evidences that the states, which are (approximately) at the same energy in Supplementary Fig. 106A are in fact identical, because they yield the very same simulated spectroscopic observables. This is illustrated for the example of the lowest-energy BS state (Supplementary Fig. 107):

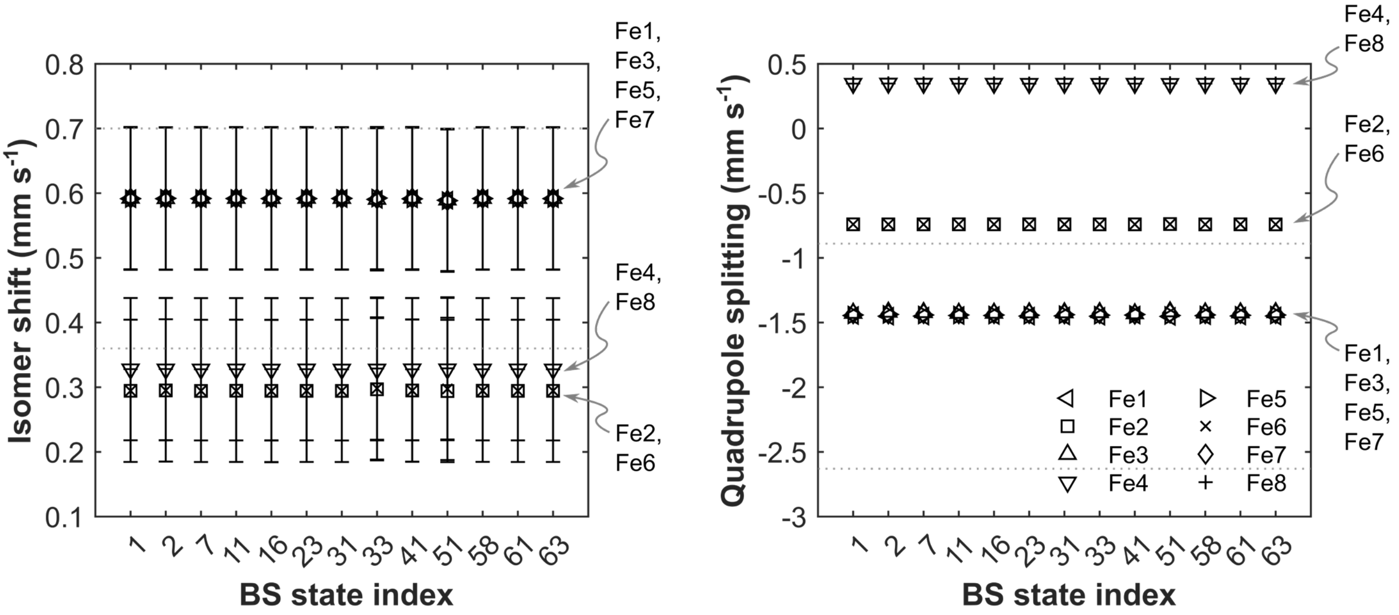

Supplementary Figure 107. Calculated DFT-Mössbauer parameters of ***ildc*** for the lowest-energy converged BS-solutions. The isomer shifts are summarized on the left, while the quadrupole splittings are summarized on the right. The fact that all solutions yield the same parameters illustrates that they are equivalent. *Dotted grey lines* mark the experimental values.

The computed isomer shifts for the 12 unique converged BS solutions are similar in the sense that higher isomers shifts are predicted for Fe atoms 1,3,5 and 7, than for Fe sites 2, 3, 6 and 8 (Supplementary Fig. 108, *top*). Accordingly, the DFT simulations support the discrete localization of Fe(II) and Fe(III) valences in ***ildc***. The associated quadrupole splittings for the 12 unique spin topologies, however differ significantly from one another (Supplementary Fig. 108, *bottom*). Among the different solutions, we were pleased to realize that the energetically lowest one (for example BS41) shows the best qualitative agreement with experiment (Supplementary Table 12): The isomer shifts are within error of the empirical calibration curve (0.11 mm s^{-1}),[56] and the quadrupole splittings show the same large and negative value for Fe1, Fe3, Fe5 and Fe7, while being smaller for the

higher-valent Fe sites with η-values close to 1, rendering the sign of their quadrupole splittings ambiguous.

Supplementary Table 12. Summary of the experimental and calculated ^{57}Fe Mössbauer parameters of the 8 Fe atoms in ***ildc***. Indices are given as described for the DFT-optimized geometry (Supplementary Fig. 105); Those discussed for the solid-state molecular structure in the main text are given in parentheses.

Fe atom	1 (3)	2 (2)	3 (4)	4 (1)	5 (3')	6 (2')	7 (4')	8 (1')
δ_{exp} (mm s^{-1})	0.70	0.36	0.70	0.36	0.70	0.36	0.70	0.36
δ_{DFT} (mm s^{-1})	0.60	0.30	0.60	0.33	0.60	0.30	0.60	0.33
$\Delta E_{Q,exp}$ (mm s^{-1})	–2.63	–0.89	–2.63	–0.89	–2.63	–0.89	–2.63	–0.89
$\Delta E_{Q,DFT}$ (mm s^{-1})	–1.45	–0.74	–1.43	0.34	–1.44	–0.74	–1.43	0.35
η_{exp}	0.5	0.8	0.5	0.8	0.5	0.8	0.5	0.8
η_{DFT}	0.32	0.88	0.31	0.96	0.32	0.88	0.31	0.95

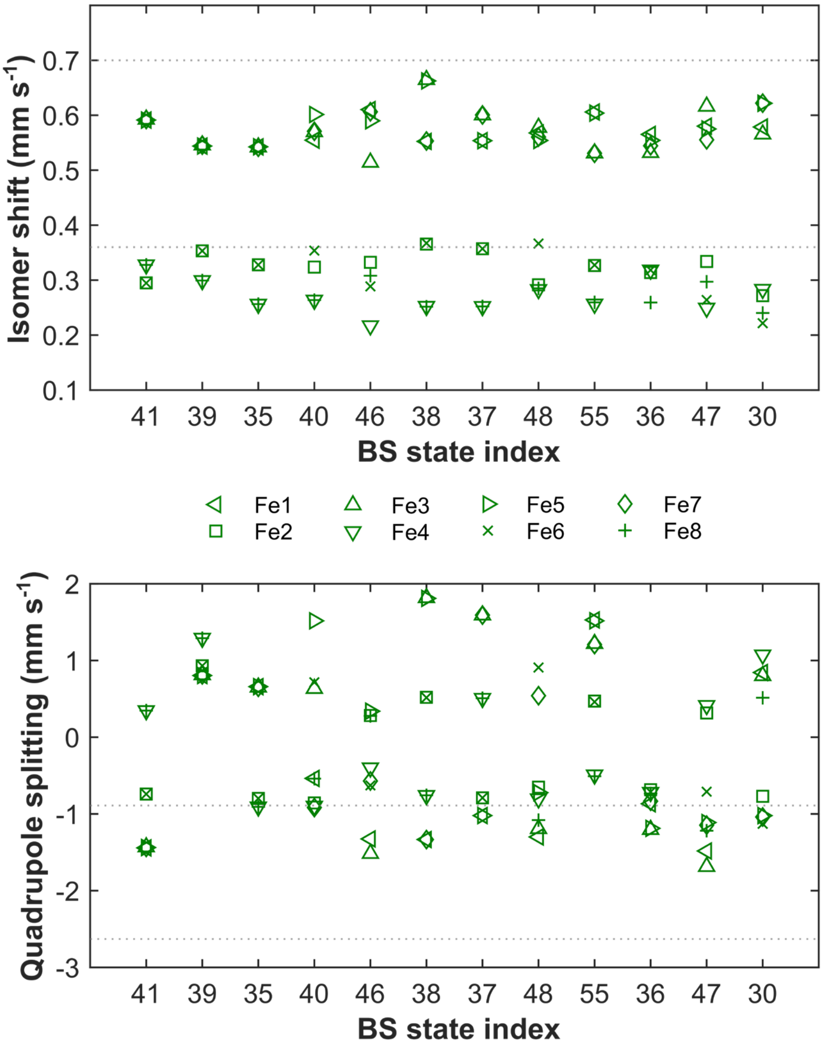

Figure S108. Calculated DFT-Mössbauer parameters of ***ildc*** for the different converged BS-solutions, which are highlighted in *green* in Supplementary Fig. 106A. The isomer shifts are summarized on the top, while the quadrupole splittings are summarized on the bottom. *Dotted grey lines* mark the experimental values.

To gain a minimal qualitative understanding of the electronic and magnetic interactions between the Fe atoms in the cluster, we carried out localization of the Kohn-Sham orbitals of the three lowest-energy BS solutions (BS41, BS39 and BS35) using Knizia's Intrinsic atomic orbital/intrinsic bond orbital (IAO/IBO) method[57] (%loc input block; "LocMet IAOIBO"), as it is popularly done for FeS clusters.[58-61] This method has proven particularly useful in identifying Fe sites engaged in spin-dependent delocalization (SDD; also referred-to as double-exchange):[62] This is possible *via* identification of a singly-occupied locally minority-spin orbital, which is shared between two adjacent Fe sites. In the classical case, *i.e.* if the SDD is completely symmetric and the Fe atoms' valences are indistinguishable, this orbital is localized on the two Fe sites equally and spans the face of the Fe_2S_2 subcluster within which SDD occurs (as shown, for example for the $[Fe_4S_4]^{2+}$ complex in Fig. 6F). SDD can however also occur asymmetrically, causing the itinerant electron to preferentially reside at either one of the two Fe atoms. This is often the case for the hetero-ligated Fe_2S_2 subclusters of site-differentiated Fe_4S_4 complexes and manifests as a difference in the Löwdin populations[63] of the orbitals on the individual Fe atoms.[59-61] Oftentimes, so-called "formal oxidation states" are computed in this manner, but for the purpose of simplicity and to pay respect to the inherent shortcomings of population analyses—they are not real molecular properties (*i.e.* not observables),[64]—we prefer to adhere to the "winner-takes-all" formalism, which is recognized to take hold if the Löwdin population of an orbital on an atom (or a group of equivalent atoms) is ≥70%. In the latter case, the electron(s) in the respective orbital are then entirely assigned to this atom (or group).
Based on this, we provide an initial discussion on the electronic origin of the valence-trapping in ***ildc***. However, it must be emphasized at this point that while broken-symmetry DFT has been demonstrated to provide valuable insights into the electronic structure of FeS complexes on many occasions,[59-61,65,66] it is not ideally suited to evaluate these systems. This is due to the fact that their spin-coupled electronic structures are in reality known not only to be highly multiconfigurational,[67,68] but also coupled to vibronic terms.[69] For the time being, we thus use broken-symmetry DFT to gain an initial understanding of the ***ildc***'s spin-topology and electronic structure—paralleling what is done popularly in the current literature,[59-61,65,66]—but point out that the nature of FeS clusters, such as ***ildc***, warrants the exploration of more specialized approaches as multi-reference theoretical methods become increasingly applicable to larger and more complex systems.

Among the three BS solutions lowest in energy, only BS41 has a propensity for SDD, owing to the presence of ferromagnetically aligned Fe(II) and Fe(III) ions in the Fe_2S_2 subclusters comprised of Fe[1,4]/Fe[3,4] or Fe[5,8]/Fe[7,8], respectively. Indeed, inspection of the IBOs revealed that only BS41 possesses orbitals characteristic for SDD—2 spin-up and 2 spin-down orbitals, representing the 4 delocalizable electrons of the Fe(II) atoms (Fe1, Fe3, Fe5 and Fe8). In BS35 and BS39 delocalization is conceptually impossible because their spin-topology lacks spin-aligned mixed-valence Fe atoms on Fe_2S_2 subclusters (Supplementary Fig. 106B). Notably, although some higher-energy BS solutions, such as BS38 for example, do have spin-topologies pertinent to SDD, inspection of the corresponding IBOs revealed no significant qualitative or quantitative differences regarding electronic delocalization with respect to the scenario we evaluated for BS41 (*vide infra*). All relevant IBOs revealed significantly asymmetric SDD interactions.

Isosurface plots of the delocalized IBOs of BS41 are shown in Supplementary Fig. 109A (and Fig. 6C):

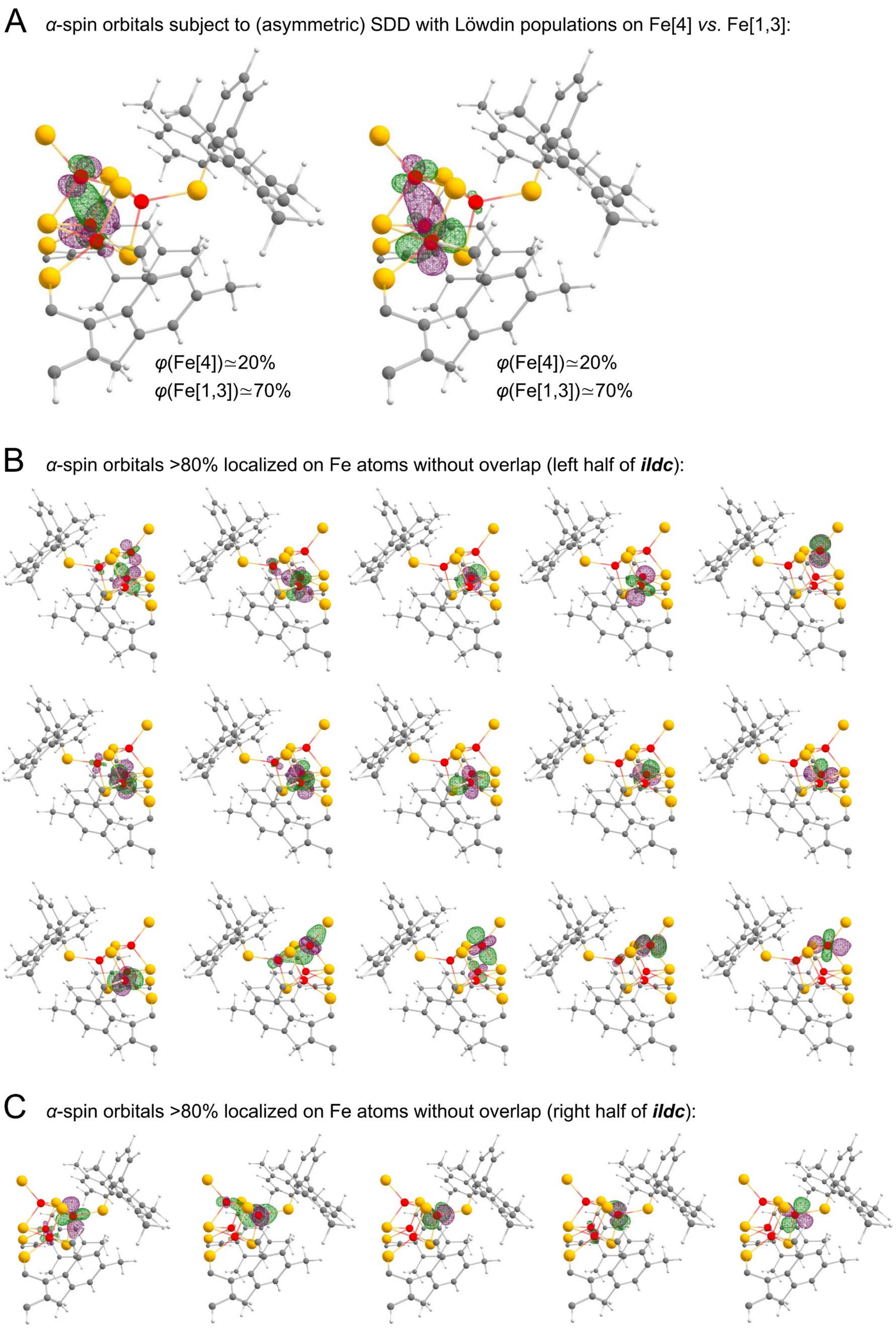

Supplementary Figure 109. (A,B,C) Molecular orbital diagrams (isosurface plots with isodensity cut-off 0.05; *purple/green mesh*) for all (22) *α*-spin metal *d*-electrons in ***ildc***, shown for BS41, which is the lowest-energy BS solution. For the sake of clarity, only one half of the ***ildc*** is represented: the left half in panel (B) and the right half in panel (C). Due to the molecules inherent symmetry, and the spin-symmetry of BS41, the *β*-spin metal *d*-electrons are in qualitatively equivalent orbitals. Panel (A) shows the MOs of interest to the evaluation of SDD. The Löwdin populations of the corresponding Fe atoms (in %) are shown alongside and are rounded to the first digit.

A corresponding Löwdin population analysis of these orbitals indicates that the 4 locally minority spin electrons pertinent to SDD are localized ca. 70-80% on Fe[1,3]/Fe[5,7] *versus* ca. 20% on Fe[4]/Fe[8] (small differences in the Löwdin populations of the α-/β-spin orbitals are observed). This represents a significant asymmetry in the double-exchange interaction, uncharacteristic for all-S ligated Fe sites in FeS clusters. In fact, within the "winner-takes-all" formalism, this scenario assigns the delocalizable electrons to the individual Fe atoms in a way that (*i*) reproduces the valence topology we anticipated based on a BVS analysis of ***ildc***'s solid-state structure (Supplementary Table 10), and (*ii*) is in line with the valences invoked by the computed (and measuered) ^{57}Fe isomer shifts.
Within a chemically intuitive hypothesis, we think that a potential origin of this asymmetry may be the different ligand properties of a μ^3- *vs*. a μ^6-sulfide and a μ^1- *vs*. a μ^2-thiolate ligand: If a sulfide/thiolate bridges more Fe atoms, the covalency of the individual Fe-S bonds is lower, while the sulfide/thiolate's effective donor-strength and charge must be significantly reduced. This local "weakening" of Fe-S bonding interactions is illustrated when comparing the structure of the (delocalized) $[Fe_4S_4]^{2+}$ derivative complex, $[Fe_4S_4(DmpS)_4]^{2-}$, with that of the $[Fe_8S_8]^{4+}$ core of ***ildc*** (Supplementary Figs. 110A,B). Evidently, the Fe_4S_4 subcluster of ***ildc*** shows a significantly "inflated" geometry around the atoms bound to the higher-order bridging ligand ($\mu^6(S^{2-})$), which results in longer Fe-S and Fe-Fe distances compared to those in $[Fe_4S_4]^{2+}$.
This argument can be expanded when considering the geometric structure of the M-cluster (Supplementary Fig. 110C). In contrast to ***ildc***, whose $Fe_2S(\mu^3)S(\mu^6)$ subclusters are valence-trapped, the latter has spin-delocalized $Fe^{2.5}$-pairs as part of $Fe_2S(\mu^3)C(\mu^6)$ and/or $Fe_2S(\mu^3)S(\mu^3)$ subclusters. This has been established spectroscopically as well as computationally.[65,66,70,71] However, the M-cluster's structure, at the difference to that of ***ildc***, shows an Fe_4S_3C subunit, which is significantly contracted, resulting in shorter Fe-S/Fe-C and Fe-Fe distances with respect to $[Fe_4S_4]^{2+}$. In turn, this suggests that at the difference to a μ^6-sulfide, the μ^6-carbide is able to sustain spin-dependent delocalization across the FeS core cluster because the Fe-C(μ^6) bonds are shorter than those between Fe and a S(μ^6) ligand; The latter being a likely consequence of its high charge-to-bridged-atoms ratio.

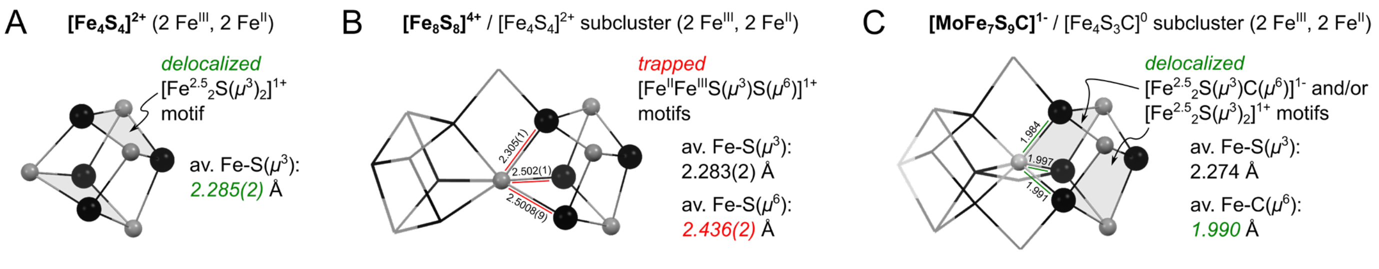

Supplementary Figure 110. Crystal structures of the $[Fe_4S_4]^{2+}$ core in $[Fe_4S_4(DmpS)_4]^{2-}$ (A), one of the two (equivalent) $[Fe_4S_4]^{2+}$ subclusters in ***ildc*** (B), and the $[Fe_4S_3C]^0$ subcluster in the M-cluster of nitrogenase (C; PDB code 3U7Q)[72]. In all structures, a (potential) arrangement of delocalized pairs is indicated by *shaded grey areas*, and the respective average Fe-S(μ^3) bond lengths are given alongside. In panels (B) and (C), the Fe-S(μ^6) and Fe-C(μ^6) distances are highlighted using colored lines, and their average is summarized alongside.

Altogether it must be emphasized that the exact physical origin of valence trapping in ***ildc*** is undoubtedly more complex, encompassing contributions from multiple possible S>0 excited states, m_S=0 excited-state spin topologies and electronic states, as well as vibronic terms. The picture we draw above, for the solution designated "BS41" (which was determined to be the most suitable based on an "unbiased" approach) is simple, and merely underscores the notion that inherent ground-state electronic structure effects contribute to the experimentally observed phenomenon.

Lastly the ^{57}Fe NRVS PVDOS spectrum of ***ildc*** was simulated by using a Numerical frequency calculation ("NumFreq"; an analytical frequency calculation proved unfeasible for ***ildc*** with the computational resources at our disposal). For this, the same level of theory as previously was used, *i.e.* deploying the BP86 functional together with the def2-SVP basis set for C and H and the def2-TZVP basis set for Fe and S. The calculated hessian was analysed using the "orca_vib" program and transformed into the NRVS spectrum using "orca_mapspc". To approximate the experimental lineshapes, the simulated signals were broadened by Lorentzian lines with 14 cm^{-1} width, as done previously in comparable simulations.[11,73] After testing multiple scaling schemes, an empirical scaling factor, $F(E)$, linearly dependent on the energy was applied to the theoretical spectrum:

$$F(E) = (2.6587 \times 10^{-5})E + 1$$

This corresponds to (rather small) a scaling factor of 1.011 for the energetically highest feature at 420 cm^{-1}.

We considered the spectra obtained for the three lowest-energy BS solutions (BS41, BS39 and BS35), and were pleased to find that BS41 most closely reproduced the experimentally observed features (Supplementary Figs. 111 and 112). The corresponding normal modes were visualized and evaluated directly from the ORCA output file in Chemcraft.[74] Animated visualizations of selected modes, which were discussed in the main text are appended as .gif files in the online *Supporting Materials*, wherein the vibrational displacement was scaled by a factor of 2 for clarity. The corresponding modes are marked with asterisks in the "stick" representation of the DFT-PVDOS simulation below:

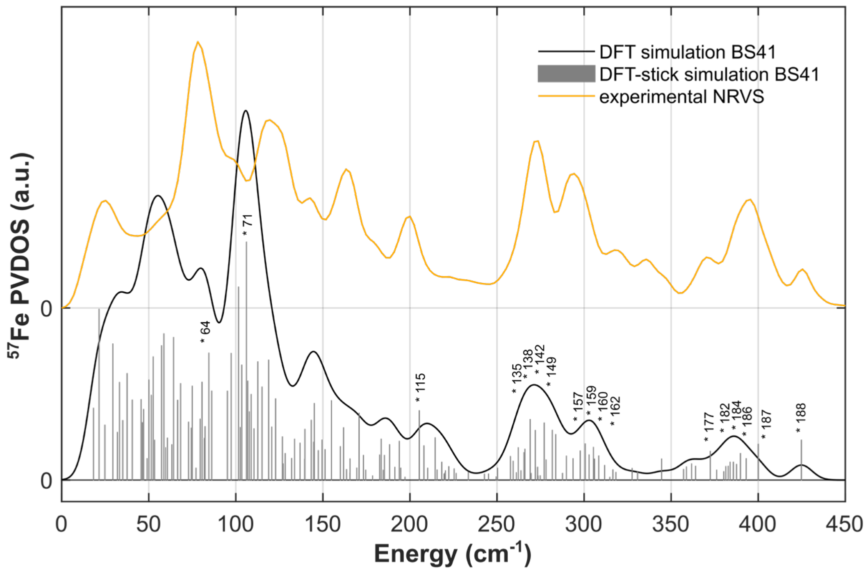

Supplementary Figure 111. Experimental (*yellow trace*) and DFT calculated (*black trace*) ^{57}Fe NRVS PVDOS spectrum of ***ildc*** for solution BS41. The individual normal modes contributing to the observed PVDOS are shown as *grey sticks*. The modes discussed in the main text and visualized in the appended .gif files are marked by their number with asterisks (*).

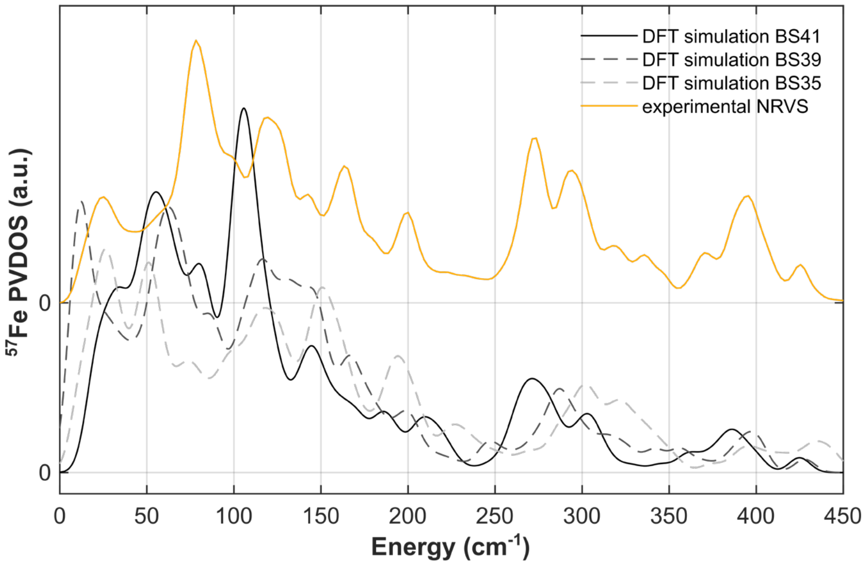

Supplementary Figure 112. Experimental (*yellow trace*) and DFT calculated ^{57}Fe NRVS PVDOS spectra of ***ildc*** for solutiona BS41 (*solid black trace*), BS39 (*dark grey dashed trace*) and BS35 (*light grey dashed trace*).

Finally, geometry optimizations and calculations of Mössbauer parameters in complete analogy to those described above, for ***ildc***, were carried out for $[Fe_4S_4(DmpS)_4]^{2-}$. The comparative results of these calculations are summarized in panels D-F of Fig. 6 in the main text.

References

1 Grunwald, L. *et al.* A complete biomimetic iron-sulfur cubane redox series. *Proceedings of the National Academy of Sciences* **119**, e2122677119, doi:10.1073/pnas.2122677119 (2022).

2 Grunwald, L., Inoue, M., Carril, P. C., Wörle, M. & Mougel, V. Gated electron transfers at synthetic iron-sulfur cubanes. *Chem* **10**, 365-387, doi:10.1016/j.chempr.2023.09.023 (2024).

3 Ohki, Y., Ikagawa, Y. & Tatsumi, K. Synthesis of New [8Fe-7S] Clusters: A Topological Link between the Core Structures of P-Cluster, FeMo-co, and FeFe-co of Nitrogenases. *Journal of the American Chemical Society* **129**, 10457-10465, doi:10.1021/ja072256b (2007).

4 Ellison, J. J., Ruhlandt-Senge, K. & Power, P. P. Synthesis and Characterization of Thiolato Complexes with Two-Coordinate Iron(II). *Angewandte Chemie International Edition in English* **33**, 1178-1180, doi:10.1002/anie.199411781 (1994).

5 Heintz, R. A., Smith, J. A., Szalay, P. S., Weisgerber, A. & Dunbar, K. R. in *Inorganic Syntheses Inorganic Syntheses* (ed Dimitri Coucouvanis) 75-121 (2002).

6 Jolly, W. L., Carey, N. A. D. & Clark, H. C. in *Inorganic Syntheses Inorganic Syntheses* (ed William L. Jolly) 120-122 (1968).

7 Barras, J.-P. *et al.* Synthesis and reactivity of the pentamethylcyclopentadienyl iron acetyl complex [(η^5-C_5Me_5)Fe(CO)(PPh_3)COMe]. *Journal of Organometallic Chemistry* **461**, 157-165, doi:10.1016/0022-328X(93)83287-6 (1993).

8 Carboni, M. *et al.* Biologically Relevant Heterodinuclear Iron–Manganese Complexes. *Inorganic Chemistry* **51**, 10447-10460, doi:10.1021/ic301725z (2012).

9 Wang, H., Braun, A., Cramer, S. P., Gee, L. B. & Yoda, Y. Nuclear Resonance Vibrational Spectroscopy: A Modern Tool to Pinpoint Site-Specific Cooperative Processes. *Crystals* **11**, 909, doi:10.3390/cryst11080909 (2021).

10 Guo, Y. *et al.* Characterization of the Fe Site in Iron−Sulfur Cluster-Free Hydrogenase (Hmd) and of a Model Compound via Nuclear Resonance Vibrational Spectroscopy (NRVS). *Inorganic Chemistry* **47**, 3969-3977, doi:10.1021/ic701251j (2008).

11 Kamali, S. *et al.* Observation of the Fe-CN and Fe-CO Vibrations in the Active Site of [NiFe] Hydrogenase by Nuclear Resonance Vibrational Spectroscopy. *Angewandte Chemie International Edition* **52**, 724-728, doi:10.1002/anie.201204616 (2013).

12 Ogata, H. *et al.* Hydride bridge in [NiFe]-hydrogenase observed by nuclear resonance vibrational spectroscopy. *Nature Communications* **6**, 7890, doi:10.1038/ncomms8890 (2015).

13 Gee, L. B., Wang, H. & Cramer, S. P. in *Bioorganometallic Chemistry* (eds Wolfgang Weigand & Ulf-Peter Apfel) 353-394 (De Gruyter, 2020).

14 Sturhahn, W. CONUSS and PHOENIX: Evaluation of nuclear resonant scattering data. *Hyperfine Interactions* **125**, 149-172, doi:10.1023/A:1012681503686 (2000).

15 Johnson, D. W. & Spence, J. C. H. Determination of the single-scattering probability distribution from plural-scattering data. *Journal of Physics D: Applied Physics* **7**, 771, doi:10.1088/0022-3727/7/6/304 (1974).

16 Wang, H., Yoda, Y., Kamali, S., Zhou, Z.-H. & Cramer, S. P. Real sample temperature: a critical issue in the experiments of nuclear resonant vibrational spectroscopy on biological samples. *Journal of Synchrotron Radiation* **19**, 257-263, doi:10.1107/S0909049512001380 (2012).

17 Mayerle, J. J., Denmark, S. E., DePamphilis, B. V., Ibers, J. A. & Holm, R. H. Synthetic analogs of the active sites of iron-sulfur proteins. XI. Synthesis and properties of complexes containing the iron sulfide (Fe_2S_2) core and the structures of bis[o-xylyl-α,α'-dithiolato-μ-sulfido-ferrate(III)] and bis[p-tolylthiolato-μ-sulfido-ferrate(III)] dianions. *Journal of the American Chemical Society* **97**, 1032-1045, doi:10.1021/ja00838a015 (1975).

18 Ballmann, J., Dechert, S., Demeshko, S. & Meyer, F. Tuning Electronic Properties of Biomimetic [2Fe-2S] Clusters by Ligand Variations. *European Journal of Inorganic Chemistry* **2009**, 3219-3225, doi:10.1002/ejic.200900101 (2009).

19 Baumann, B. *et al.* Structure of *Synechococcus elongatus* [Fe_2S_2] Ferredoxin in Solution. *Biochemistry* **35**, 12831-12841, doi:10.1021/bi961144m (1996).

20 Van, V. *et al.* Iron-sulfur clusters are involved in post-translational arginylation. *Nature Communications* **14**, 458, doi:10.1038/s41467-023-36158-z (2023).

21 Fleischhacker, A. S. *et al.* Characterization of the [2Fe-2S] Cluster of Escherichia coli Transcription Factor IscR. *Biochemistry* **51**, 4453-4462, doi:10.1021/bi3003204 (2012).

22 Golinelli-Cohen, M.-P. *et al.* Redox Control of the Human Iron-Sulfur Repair Protein MitoNEET Activity via Its Iron-Sulfur Cluster. *Journal of Biological Chemistry* **291**, 7583-7593, doi:10.1074/jbc.M115.711218 (2016).

23 Stegmaier, K. *et al.* Apd1 and Aim32 Are Prototypes of Bishistidinyl-Coordinated Non-Rieske [2Fe–2S] Proteins. *Journal of the American Chemical Society* **141**, 5753-5765, doi:10.1021/jacs.8b13274 (2019).

24 Tanifuji, K., Tajima, S., Ohki, Y. & Tatsumi, K. Interconversion between [Fe_4S_4] and [Fe_2S_2] Clusters Bearing Amide Ligands. *Inorganic Chemistry* **55**, 4512-4518, doi:10.1021/acs.inorgchem.6b00352 (2016).

25 Skeel, B. A. & Suess, D. L. M. Exploiting Molecular Symmetry to Quantitatively Map the Excited-State Landscape of Iron–Sulfur Clusters. *Journal of the American Chemical Society* **145**, 10376-10395, doi:10.1021/jacs.3c02412 (2023).

26 Ohki, Y. *et al.* Synthetic analogues of [$Fe_4S_4(Cys)_3(His)$] in hydrogenases and [$Fe_4S_4(Cys)_4$] in HiPIP derived from all-ferric [$Fe_4S_4\{N(SiMe_3)_2\}_4$]. *Proceedings of the National Academy of Sciences* **108**, 12635-12640, doi:10.1073/pnas.1106472108 (2011).

27 Middleton, P., Dickson, D. P. E., Johnson, C. E. & Rush, J. D. Interpretation of the Mössbauer Spectra of the Four-Iron Ferredoxin from Bacillus stearothermophilus. *European Journal of Biochemistry* **88**, 135-141, doi:10.1111/j.1432-1033.1978.tb12430.x (1978).

28 Lindahl, P. A., Day, E. P., Kent, T. A., Orme-Johnson, W. H. & Münck, E. Mössbauer, EPR, and magnetization studies of the Azotobacter vinelandii Fe protein. Evidence for a $[4Fe\text{-}4S]^{1+}$ cluster with spin S=3/2. *Journal of Biological Chemistry* **260**, 11160-11173, doi:10.1016/S0021-9258(17)39160-3 (1985).

29 Roncaroli, F. *et al.* Cofactor composition and function of a H_2-sensing regulatory hydrogenase as revealed by Mössbauer and EPR spectroscopy. *Chemical Science* **6**, 4495-4507, doi:10.1039/C5SC01560J (2015).

30 Middleton, P., Dickson, D. P. E., Johnson, C. E. & Rush, J. D. Interpretation of the Mössbauer Spectra of the High-Potential Iron Protein from Chromatium. *European Journal of Biochemistry* **104**, 289-296, doi:10.1111/j.1432-1033.1980.tb04427.x (1980).

31 Dilg, A. W. E. *et al.* Comparison and characterization of the $[Fe_4S_4]^{2+/3+}$ centre in the wild-type and C77S mutated HiPIPs from Chromatium vinosum monitored by Mössbauer, ^{57}Fe ENDOR and EPR

spectroscopies. *JBIC Journal of Biological Inorganic Chemistry* **6**, 232-246, doi:10.1007/s007750000191 (2001).

32 Grunwald, L., Abbott, D. F. & Mougel, V. Gauging Iron–Sulfur Cubane Reactivity from Covalency: Trends with Oxidation State. *JACS Au* **4**, 1315-1322, doi:10.1021/jacsau.4c00213 (2024).

33 Zhu, Q., Costentin, C., Stubbe, J. & Nocera, D. G. Disulfide radical anion as a super-reductant in biology and photoredox chemistry. *Chemical Science* **14**, 6876-6881, doi:10.1039/D3SC01867A (2023).

34 Dolomanov, O. V., Bourhis, L. J., Gildea, R. J., Howard, J. A. K. & Puschmann, H. OLEX2: a complete structure solution, refinement and analysis program. *Journal of Applied Crystallography* **42**, 339-341, doi:doi:10.1107/S0021889808042726 (2009).

35 Sheldrick, G. A short history of SHELX. *Acta Crystallographica Section A* **64**, 112-122, doi:10.1107/S0108767307043930 (2008).

36 Sheldrick, G. Crystal structure refinement with SHELXL. *Acta Crystallographica Section C* **71**, 3-8, doi:10.1107/S2053229614024218 (2015).

37 Sheldrick, G. SHELXT - Integrated space-group and crystal-structure determination. *Acta Crystallographica Section A* **71**, 3-8, doi:10.1107/S2053273314026370 (2015).

38 Kratzert, D., Holstein, J. J. & Krossing, I. DSR: enhanced modelling and refinement of disordered structures with SHELXL. *Journal of Applied Crystallography* **48**, 933-938, doi:10.1107/S1600576715005580 (2015).

39 Kratzert, D. & Krossing, I. Recent improvements in DSR. *Journal of Applied Crystallography* **51**, 928-934, doi:10.1107/S1600576718004508 (2018).

40 Brown, I. D. in *The Chemical Bond in Inorganic Chemistry* (ed I. David Brown) (Oxford University Press, 2016).

41 Liu, W. & Thorp, H. H. Bond valence sum analysis of metal-ligand bond lengths in metalloenzymes and model complexes. 2. Refined distances and other enzymes. *Inorganic Chemistry* **32**, 4102-4105, doi:10.1021/ic00071a023 (1993).

42 Brown, I. D. Recent Developments in the Methods and Applications of the Bond Valence Model. *Chemical Reviews* **109**, 6858-6919, doi:10.1021/cr900053k (2009).

43 Jenner, L. P., Cherrier, M. V., Amara, P., Rubio, L. M. & Nicolet, Y. An unexpected P-cluster like intermediate en route to the nitrogenase FeMo-co. *Chemical Science* **12**, 5269-5274, doi:10.1039/D1SC00289A (2021).

44 Goddard, T. D. *et al.* UCSF ChimeraX: Meeting modern challenges in visualization and analysis. *Protein Science* **27**, 14-25, doi:10.1002/pro.3235 (2018).

45 Pettersen, E. F. *et al.* UCSF ChimeraX: Structure visualization for researchers, educators, and developers. *Protein Science* **30**, 70-82, doi:10.1002/pro.3943 (2021).

46 Neese, F. Software update: The ORCA program system—Version 5.0. *WIREs Computational Molecular Science* **12**, e1606, doi:10.1002/wcms.1606 (2022).

47 Noodleman, L., Case, D. A. & Aizman, A. Broken symmetry analysis of spin coupling in iron-sulfur clusters. *Journal of the American Chemical Society* **110**, 1001-1005, doi:10.1021/ja00212a003 (1988).

48 Neese, F. Definition of corresponding orbitals and the diradical character in broken symmetry DFT calculations on spin coupled systems. *Journal of Physics and Chemistry of Solids* **65**, 781-785, doi:10.1016/j.jpcs.2003.11.015 (2004).

49 Perdew, J. P. Density-functional approximation for the correlation energy of the inhomogeneous electron gas. *Physical Review B* **33**, 8822-8824, doi:10.1103/PhysRevB.33.8822 (1986).

50 Becke, A. D. Density-functional exchange-energy approximation with correct asymptotic behavior. *Physical Review A* **38**, 3098-3100, doi:10.1103/PhysRevA.38.3098 (1988).

51 Weigend, F. Accurate Coulomb-fitting basis sets for H to Rn. *Physical Chemistry Chemical Physics* **8**, 1057-1065, doi:10.1039/B515623H (2006).

52 Weigend, F. & Ahlrichs, R. Balanced basis sets of split valence, triple zeta valence and quadruple zeta valence quality for H to Rn: Design and assessment of accuracy. *Physical Chemistry Chemical Physics* **7**, 3297-3305, doi:10.1039/B508541A (2005).

53 Neese, F. An improvement of the resolution of the identity approximation for the formation of the Coulomb matrix. *Journal of Computational Chemistry* **24**, 1740-1747, doi:10.1002/jcc.10318 (2003).

54 Sandala, G. M., Hopmann, K. H., Ghosh, A. & Noodleman, L. Calibration of DFT Functionals for the Prediction of ^{57}Fe Mössbauer Spectral Parameters in Iron–Nitrosyl and Iron–Sulfur Complexes: Accurate Geometries Prove Essential. *Journal of Chemical Theory and Computation* **7**, 3232-3247, doi:10.1021/ct200187d (2011).

55 Neese, F. Prediction and interpretation of the ^{57}Fe isomer shift in Mössbauer spectra by density functional theory. *Inorganica Chimica Acta* **337**, 181-192, doi:10.1016/S0020-1693(02)01031-9 (2002).

56 Römelt, M., Ye, S. & Neese, F. Calibration of Modern Density Functional Theory Methods for the Prediction of ^{57}Fe Mössbauer Isomer Shifts: Meta-GGA and Double-Hybrid Functionals. *Inorganic Chemistry* **48**, 784-785, doi:10.1021/ic801535v (2009).

57 Knizia, G. Intrinsic Atomic Orbitals: An Unbiased Bridge between Quantum Theory and Chemical Concepts. *Journal of Chemical Theory and Computation* **9**, 4834-4843, doi:10.1021/ct400687b (2013).

58 Bostelaar, T. M., Brown, A. C., Sridharan, A. & Suess, D. L. M. A general method for metallocluster site-differentiation. *Nature Synthesis* **2**, 740-748, doi:10.1038/s44160-023-00286-7 (2023).

59 Brown, A. C., Thompson, N. B. & Suess, D. L. M. Activation of Strong π–Acids at $[Fe_4S_4]^+$ Clusters Enabled by a Noncanonical Electronic Structure. *Journal of the American Chemical Society* **146**, 34080-34091, doi:10.1021/jacs.4c13490 (2024).

60 Brown, A. C., Thompson, N. B. & Suess, D. L. M. Evidence for Low-Valent Electronic Configurations in Iron–Sulfur Clusters. *Journal of the American Chemical Society* **144**, 9066-9073, doi:10.1021/jacs.2c01872 (2022).

61 Sridharan, A., Brown, A. C. & Suess, D. L. M. A Terminal Imido Complex of an Iron–Sulfur Cluster. *Angewandte Chemie International Edition* **60**, 12802-12806, doi:10.1002/anie.202102603 (2021).

62 Girerd, J. J., Papaefthymiou, V., Surerus, K. K. & Munck, E. Double exchange in iron-sulfur clusters and a proposed spin-dependent transfer mechanism. **61**, 805-816, doi:doi:10.1351/pac198961050805 (1989).

63 Szabo, A. & Ostlund, N. S. *Modern Quantum Chemistry: Introduction to Advanced Electronic Structure Theory*. (Dover Publications, 1996).

64 Autschbach, J. Orbitals: Some Fiction and Some Facts. *Journal of Chemical Education* **89**, 1032-1040, doi:10.1021/ed200673w (2012).

65 Benediktsson, B. & Bjornsson, R. QM/MM Study of the Nitrogenase MoFe Protein Resting State: Broken-Symmetry States, Protonation States, and QM Region Convergence in the FeMoco Active Site. *Inorganic Chemistry* **56**, 13417-13429, doi:10.1021/acs.inorgchem.7b02158 (2017).

66 Bjornsson, R., Neese, F. & DeBeer, S. Revisiting the Mössbauer Isomer Shifts of the FeMoco Cluster of Nitrogenase and the Cofactor Charge. *Inorganic Chemistry* **56**, 1470-1477, doi:10.1021/acs.inorgchem.6b02540 (2017).

67 Sharma, S., Sivalingam, K., Neese, F. & Chan, G. K.-L. Low-energy spectrum of iron–sulfur clusters directly from many-particle quantum mechanics. *Nature Chemistry* **6**, 927-933, doi:10.1038/nchem.2041 (2014).

68 Li, Z., Guo, S., Sun, Q. & Chan, G. K.-L. Electronic landscape of the P-cluster of nitrogenase as revealed through many-electron quantum wavefunction simulations. *Nature Chemistry* **11**, 1026-1033, doi:10.1038/s41557-019-0337-3 (2019).

69 Bominaar, E. L., Borshch. Serguei, A. & Girerd, J.-J. Double-Exchange and Vibronic Coupling in Mixed-Valence Systems. Electronic Structure of $[Fe_4S_4]^{3+}$ Clusters in High-Potential Iron Protein and Related Models. *Journal of the American Chemical Society* **116**, 5362-5372, doi:10.1021/ja00091a047 (1994).

70 Yoo, S. J., Angove, H. C., Papaefthymiou, V., Burgess, B. K. & Münck, E. Mössbauer Study of the MoFe Protein of Nitrogenase from Azotobacter vinelandii Using Selective ^{57}Fe Enrichment of the M-Centers. *Journal of the American Chemical Society* **122**, 4926-4936, doi:10.1021/ja000254k (2000).

71 Badding, E. D., Srisantitham, S., Lukoyanov, D. A., Hoffman, B. M. & Suess, D. L. M. Connecting the geometric and electronic structures of the nitrogenase iron–molybdenum cofactor through site-selective 57Fe labelling. *Nature Chemistry* **15**, 658-665, doi:10.1038/s41557-023-01154-9 (2023).

72 Spatzal, T. *et al.* Evidence for Interstitial Carbon in Nitrogenase FeMo Cofactor. *Science* **334**, 940-940, doi:10.1126/science.1214025 (2011).

73 Mitra, D. *et al.* Dynamics of the [4Fe-4S] Cluster in Pyrococcus furiosus D14C Ferredoxin via Nuclear Resonance Vibrational and Resonance Raman Spectroscopies, Force Field Simulations, and Density Functional Theory Calculations. *Biochemistry* **50**, 5220-5235, doi:10.1021/bi200046p (2011).

74 *Chemcraft - graphical software for visualization of quantum chemistry computations. Version 1.8, build 682.*, <https://www.chemcraftprog.com> (2025).
