# Supplementary figures and images for: Stepwise and reversible assembly of [2Fe–2S] rhombs to [8Fe–8S] clusters and their topological interconversions
Source: Nat Chem. 2025 Aug 20;17(10):1586–95. doi: 10.1038/s41557-025-01895-9 (PMC12491067; doi:10.1038/s41557-025-01895-9)

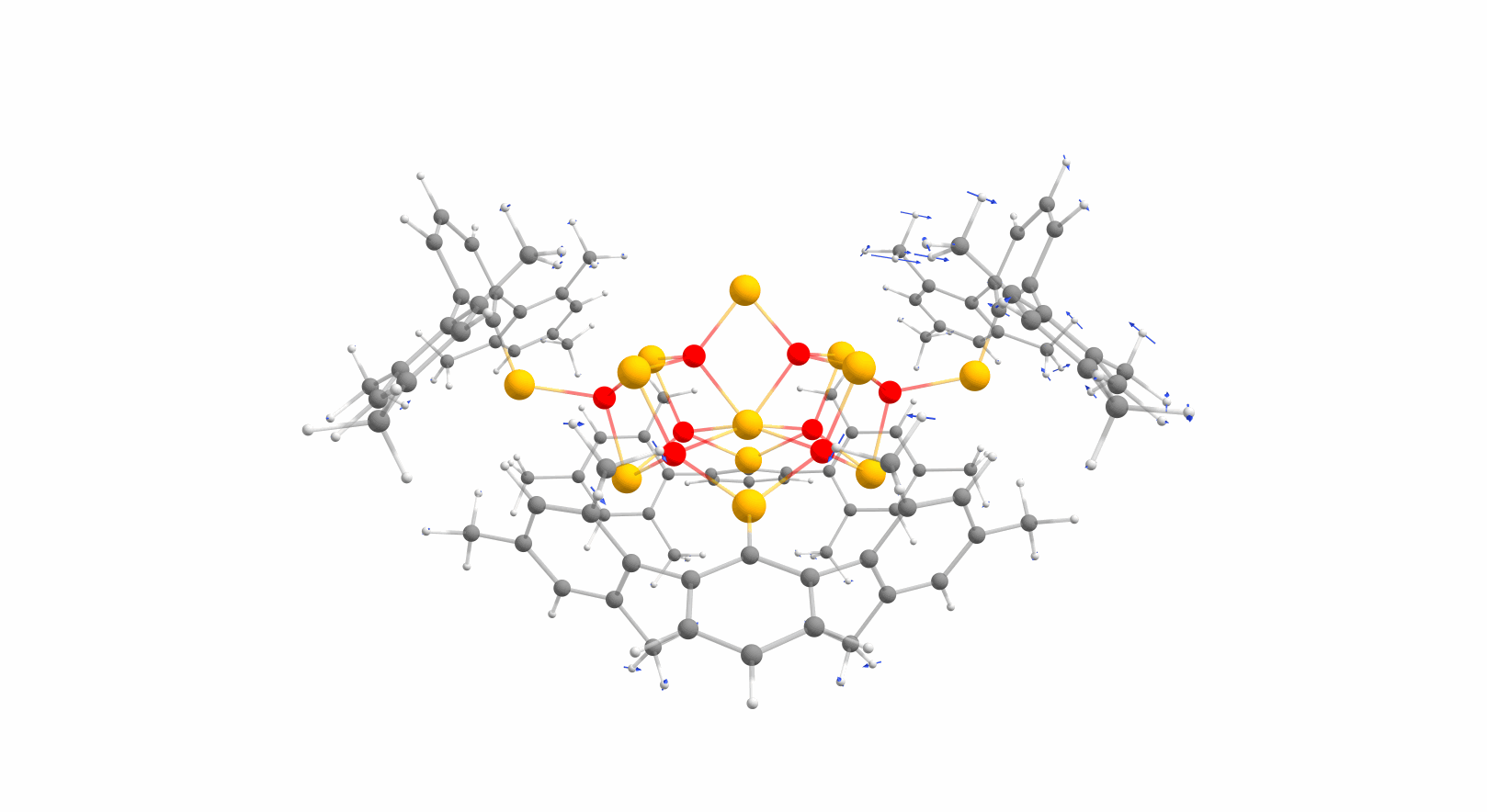

Supplement: Supplementary file 3 — GIF animations of broken-symmetry DFT-calculated normal modes of ildc. [file 41557_2025_1895_MOESM3_ESM.zip › ildc_mode#160_304cm-1.gif]

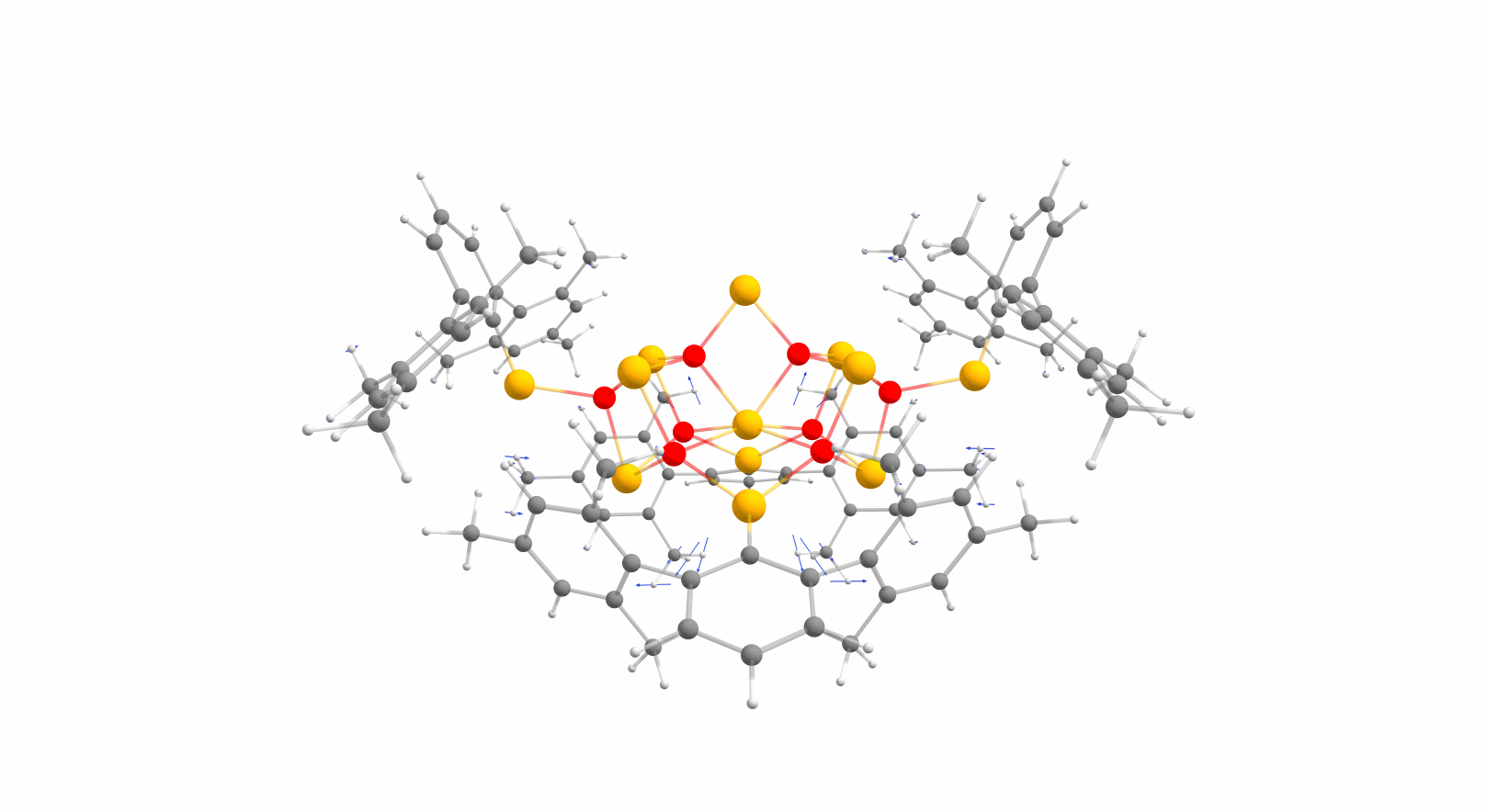

Supplement: Supplementary file 3 — GIF animations of broken-symmetry DFT-calculated normal modes of ildc. [file 41557_2025_1895_MOESM3_ESM.zip › ildc_mode#162_310cm-1.gif]

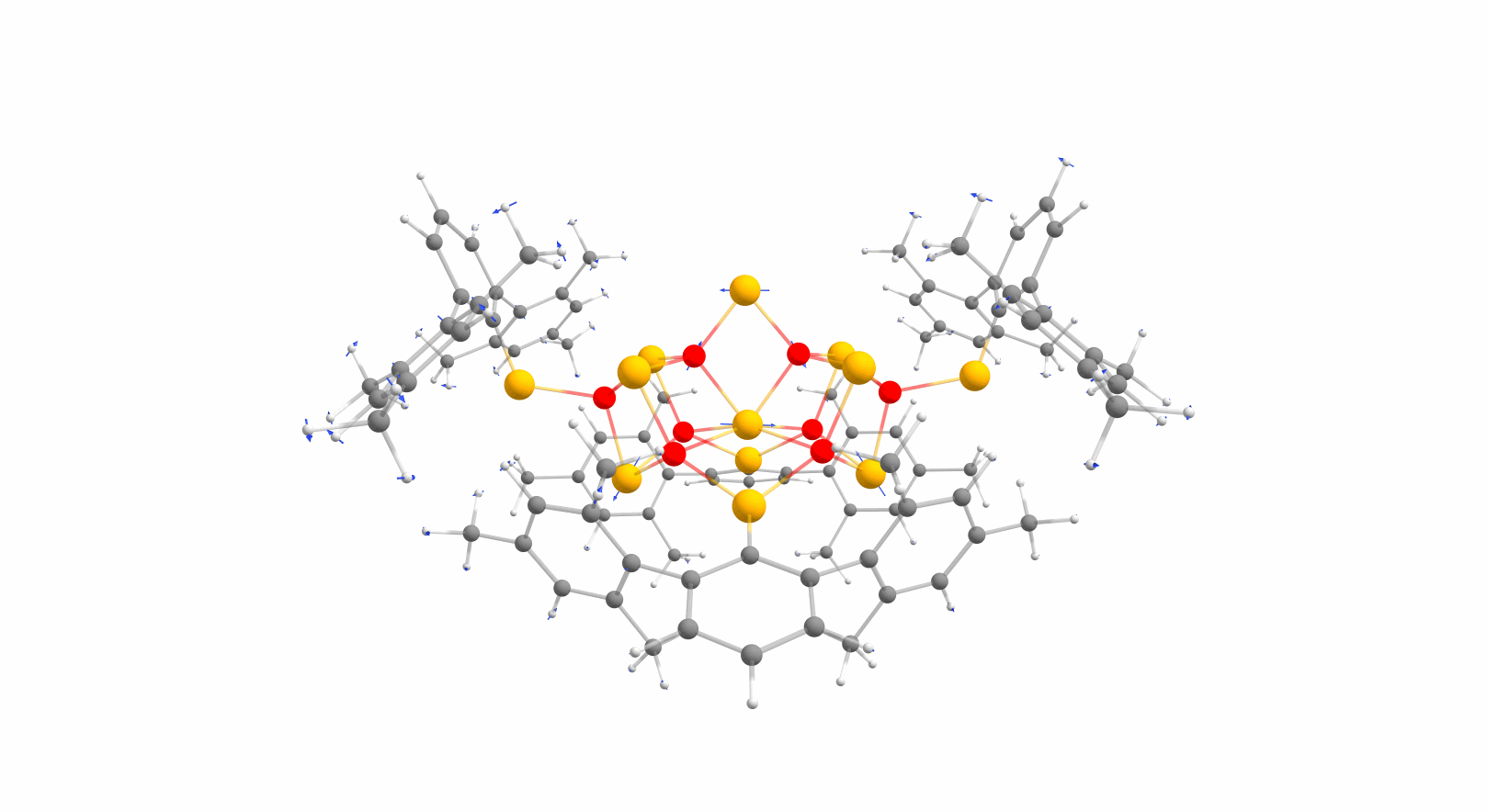

Supplement: Supplementary file 3 — GIF animations of broken-symmetry DFT-calculated normal modes of ildc. [file 41557_2025_1895_MOESM3_ESM.zip › ildc_mode#177_369cm-1.gif]

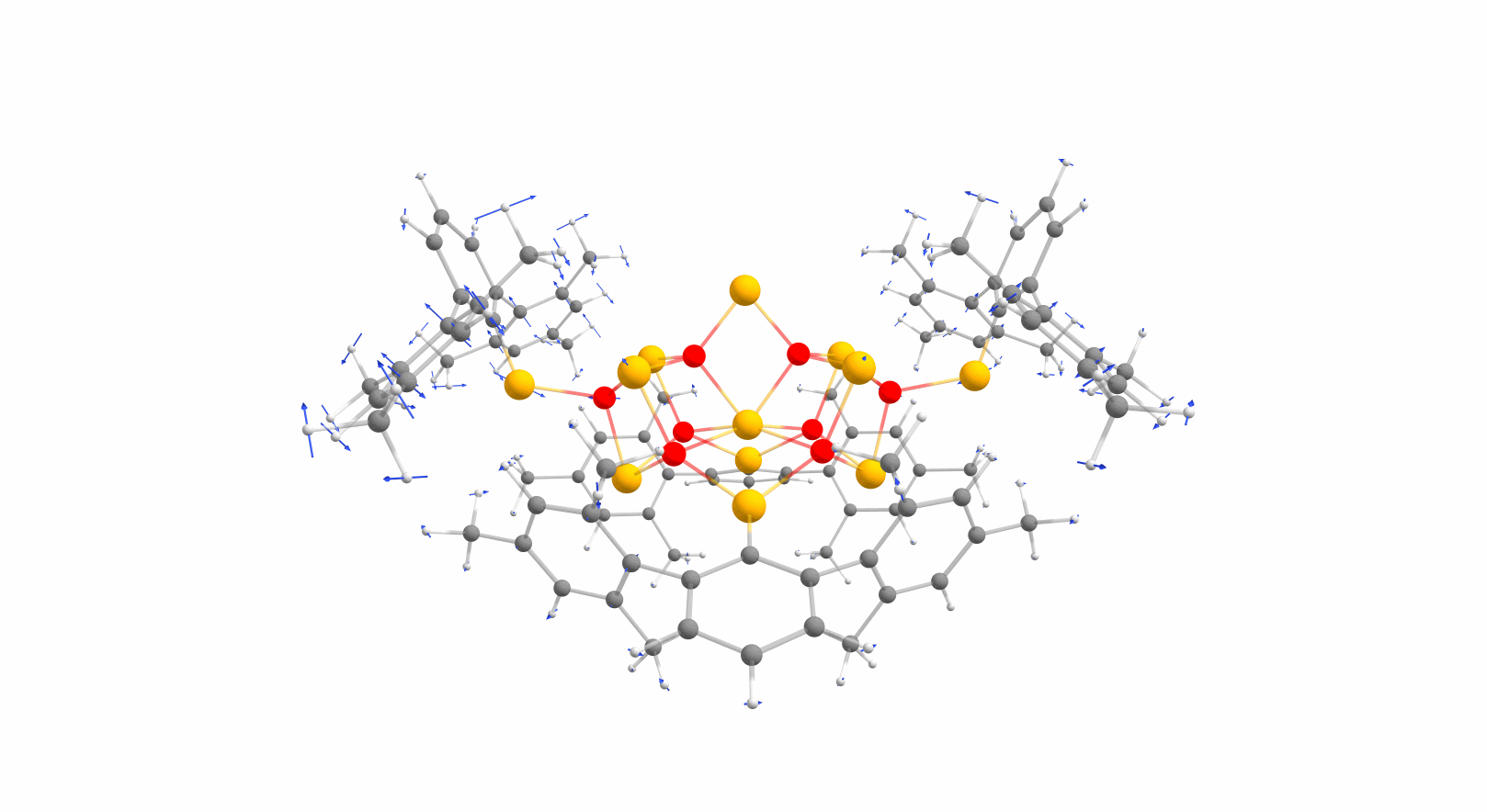

Supplement: Supplementary file 3 — GIF animations of broken-symmetry DFT-calculated normal modes of ildc. [file 41557_2025_1895_MOESM3_ESM.zip › ildc_mode#182_380cm-1.gif]

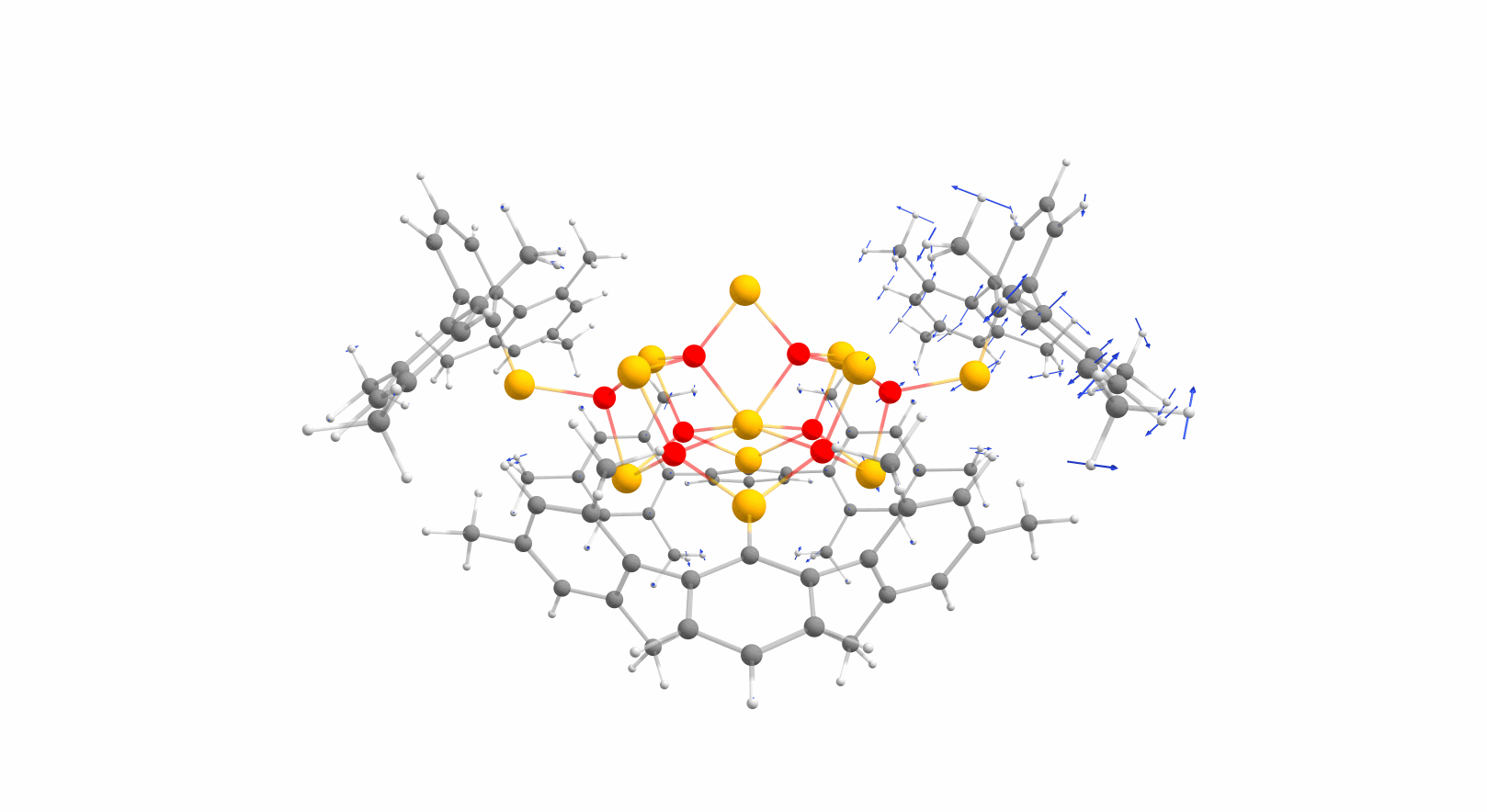

Supplement: Supplementary file 3 — GIF animations of broken-symmetry DFT-calculated normal modes of ildc. [file 41557_2025_1895_MOESM3_ESM.zip › ildc_mode#184_384cm-1.gif]

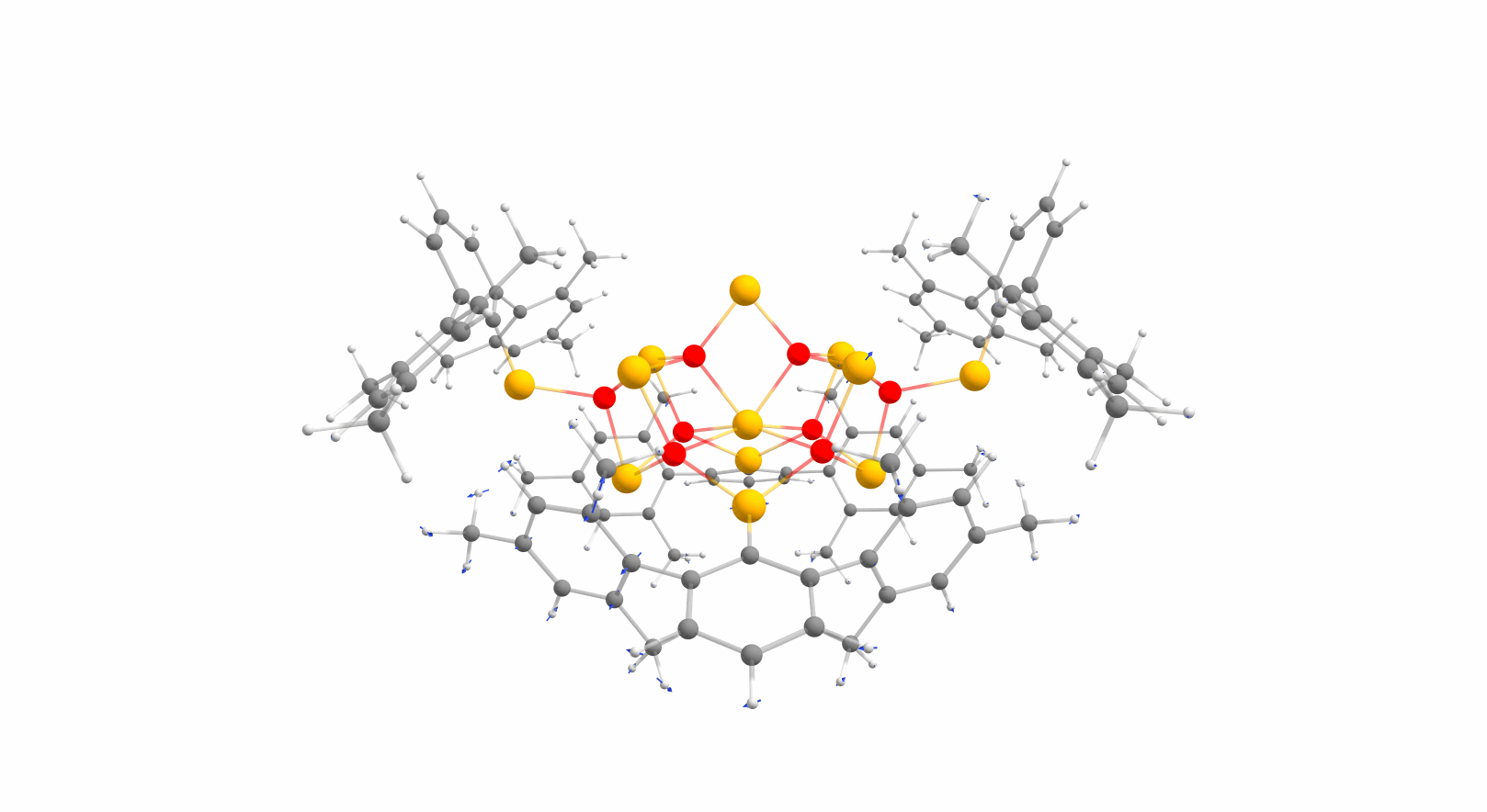

Supplement: Supplementary file 3 — GIF animations of broken-symmetry DFT-calculated normal modes of ildc. [file 41557_2025_1895_MOESM3_ESM.zip › ildc_mode#186_389cm-1.gif]

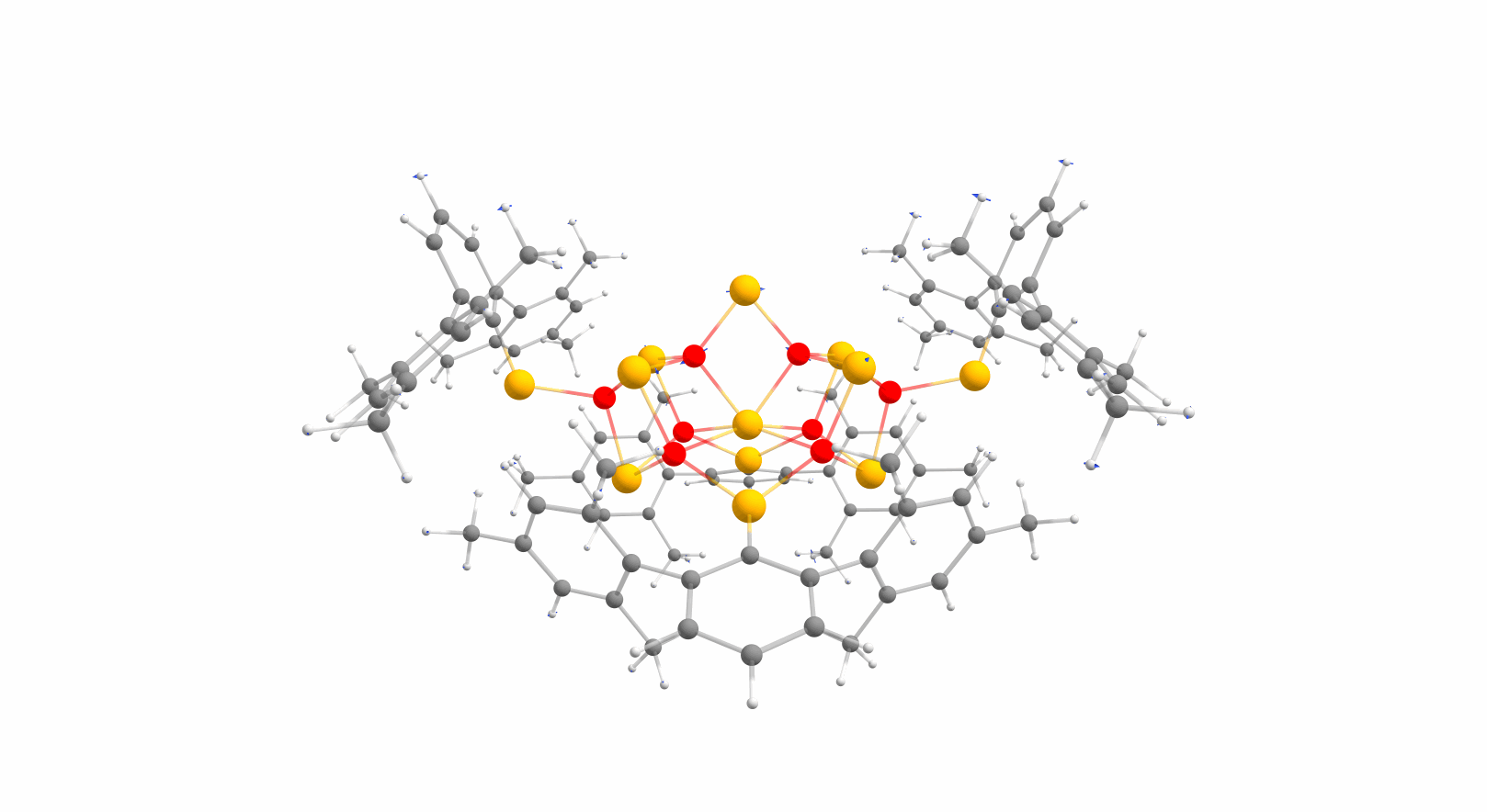

Supplement: Supplementary file 3 — GIF animations of broken-symmetry DFT-calculated normal modes of ildc. [file 41557_2025_1895_MOESM3_ESM.zip › ildc_mode#187_396cm-1.gif]

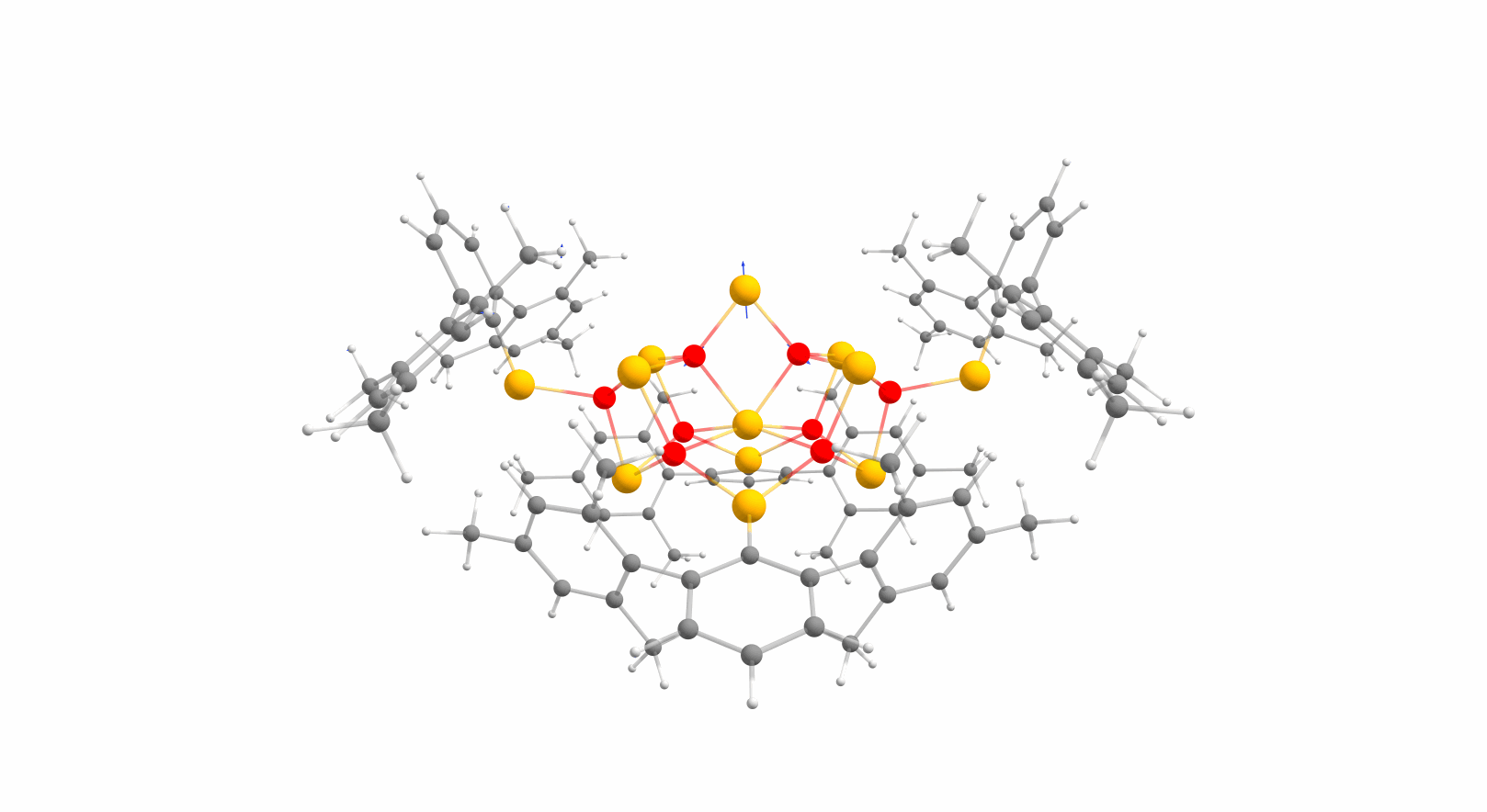

Supplement: Supplementary file 3 — GIF animations of broken-symmetry DFT-calculated normal modes of ildc. [file 41557_2025_1895_MOESM3_ESM.zip › ildc_mode#188_420cm-1.gif]

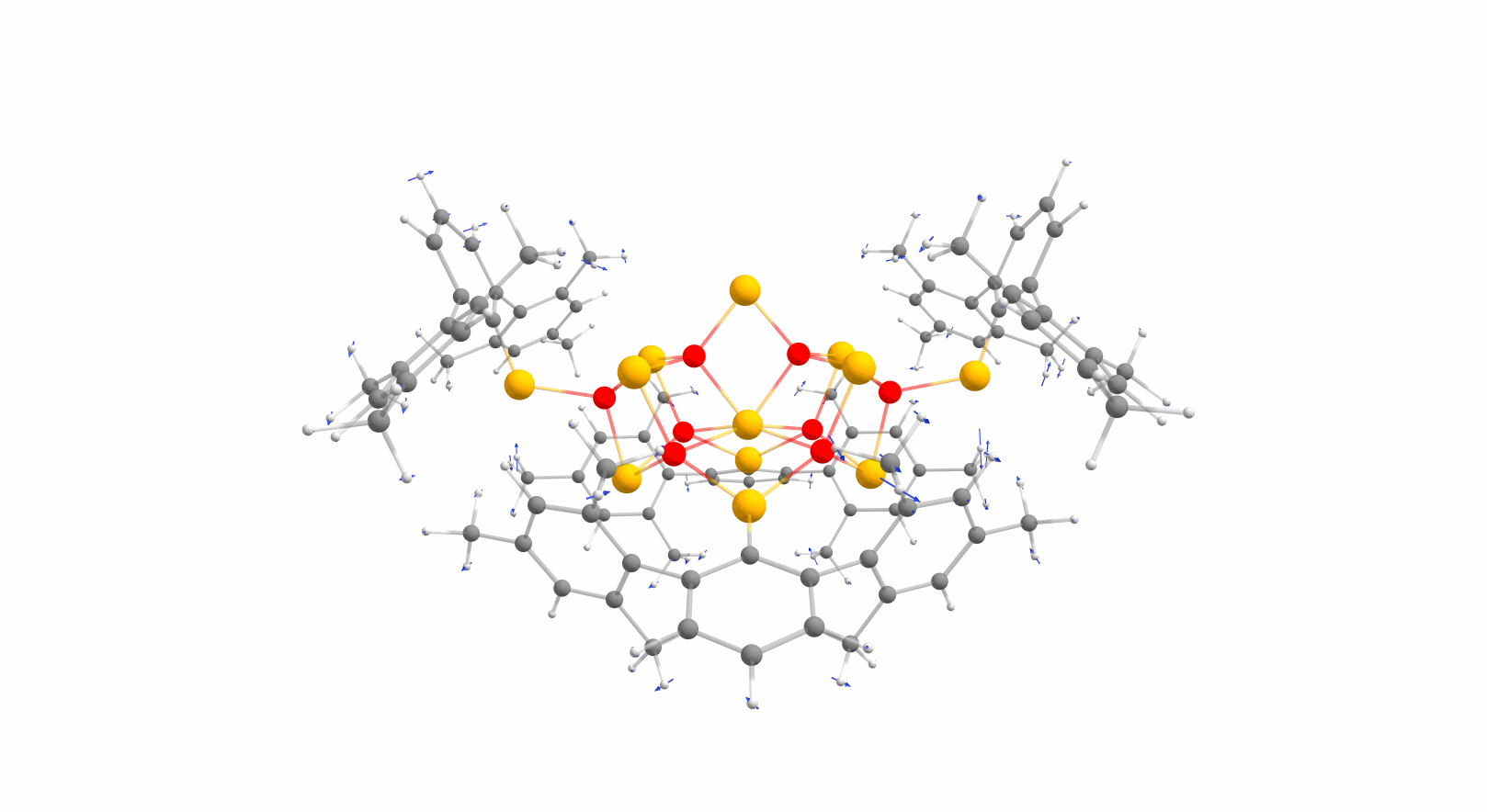

Supplement: Supplementary file 3 — GIF animations of broken-symmetry DFT-calculated normal modes of ildc. [file 41557_2025_1895_MOESM3_ESM.zip › ildc_mode#64_84cm-1.gif]

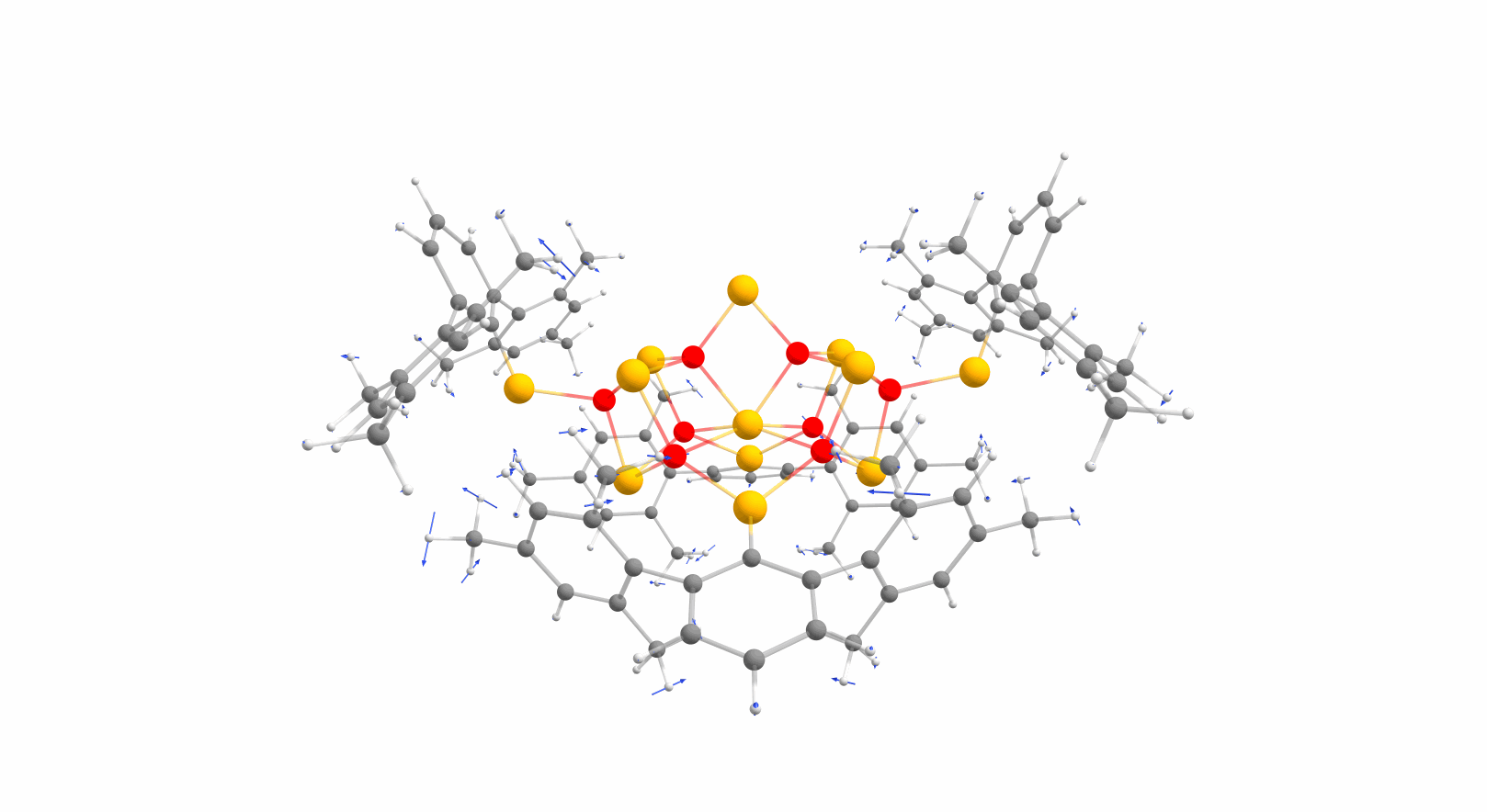

Supplement: Supplementary file 3 — GIF animations of broken-symmetry DFT-calculated normal modes of ildc. [file 41557_2025_1895_MOESM3_ESM.zip › ildc_mode#71_106cm-1.gif]

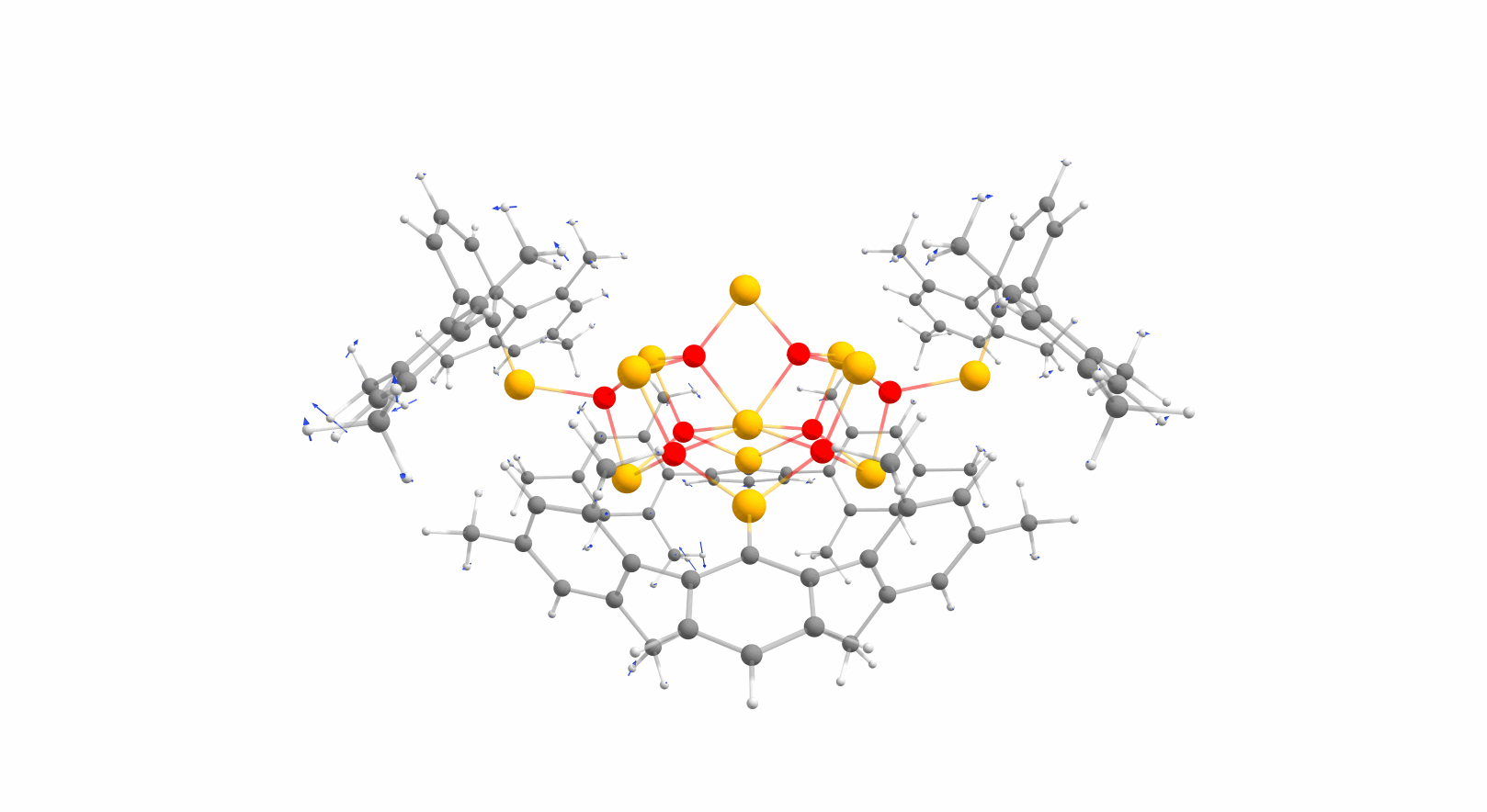

Supplement: Supplementary file 3 — GIF animations of broken-symmetry DFT-calculated normal modes of ildc. [file 41557_2025_1895_MOESM3_ESM.zip › ildc_mode#115_204cm-1.gif]

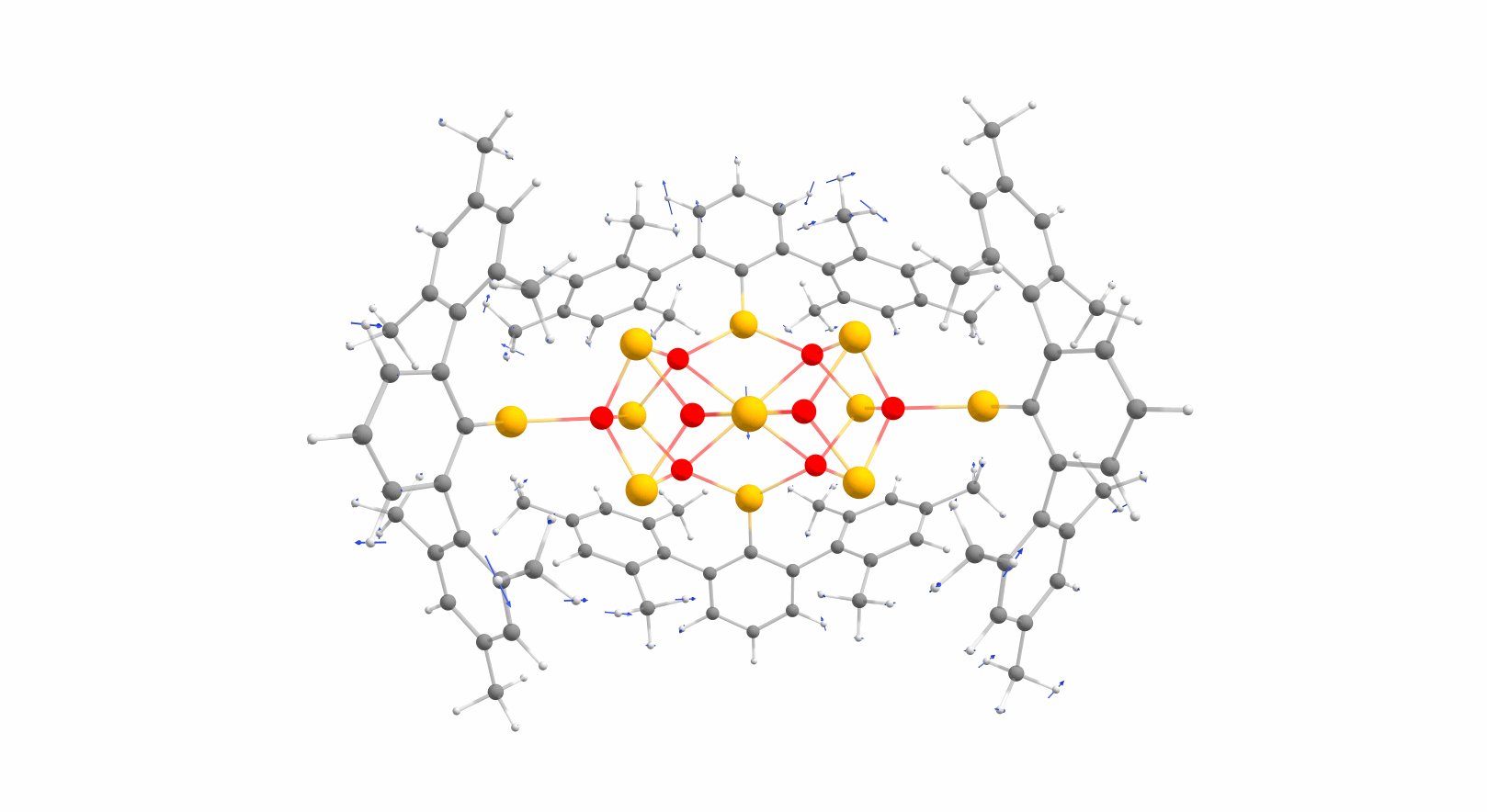

Supplement: Supplementary file 3 — GIF animations of broken-symmetry DFT-calculated normal modes of ildc. [file 41557_2025_1895_MOESM3_ESM.zip › ildc_mode#135_256cm-1.gif]

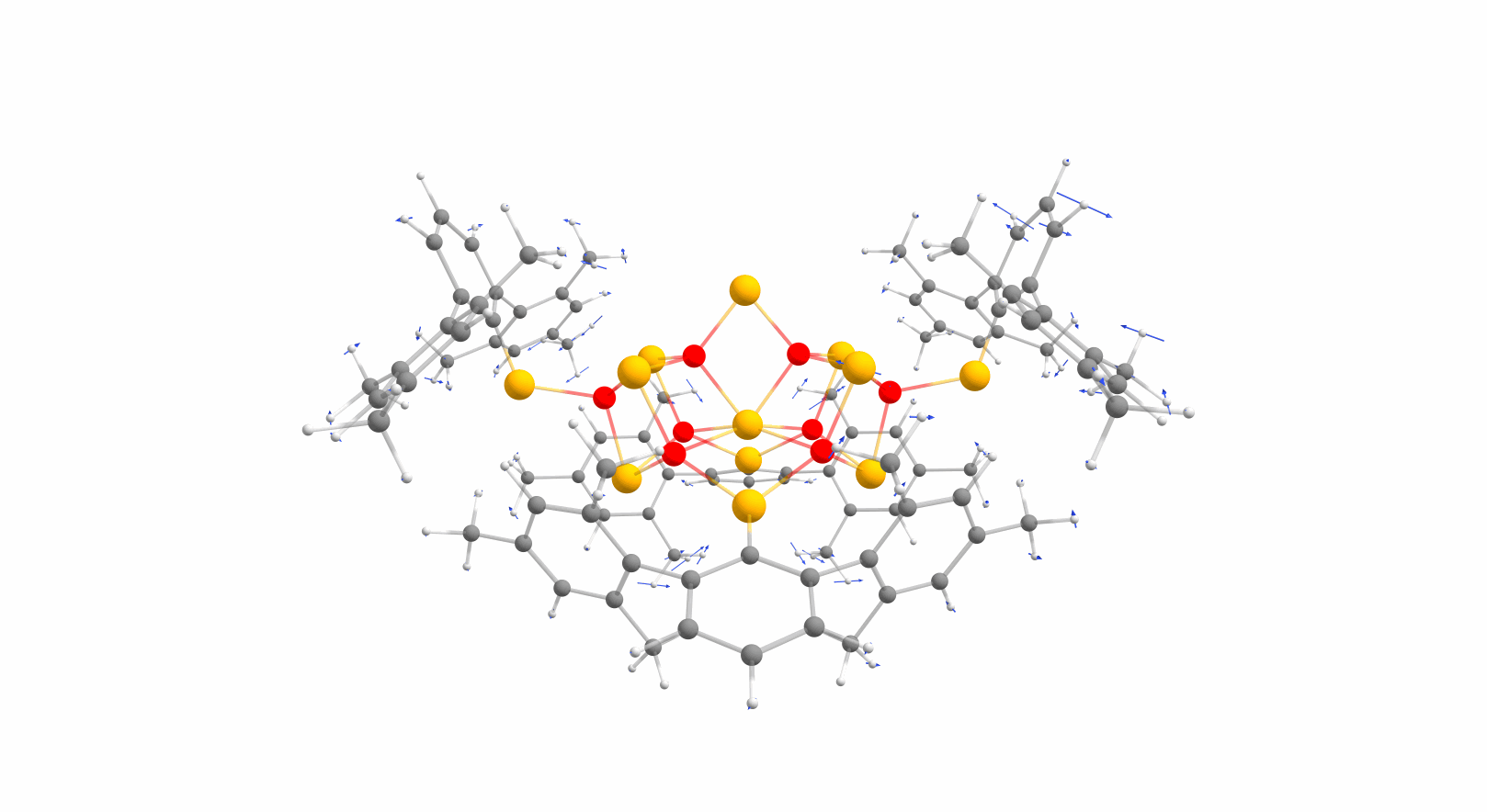

Supplement: Supplementary file 3 — GIF animations of broken-symmetry DFT-calculated normal modes of ildc. [file 41557_2025_1895_MOESM3_ESM.zip › ildc_mode#138_260cm-1.gif]

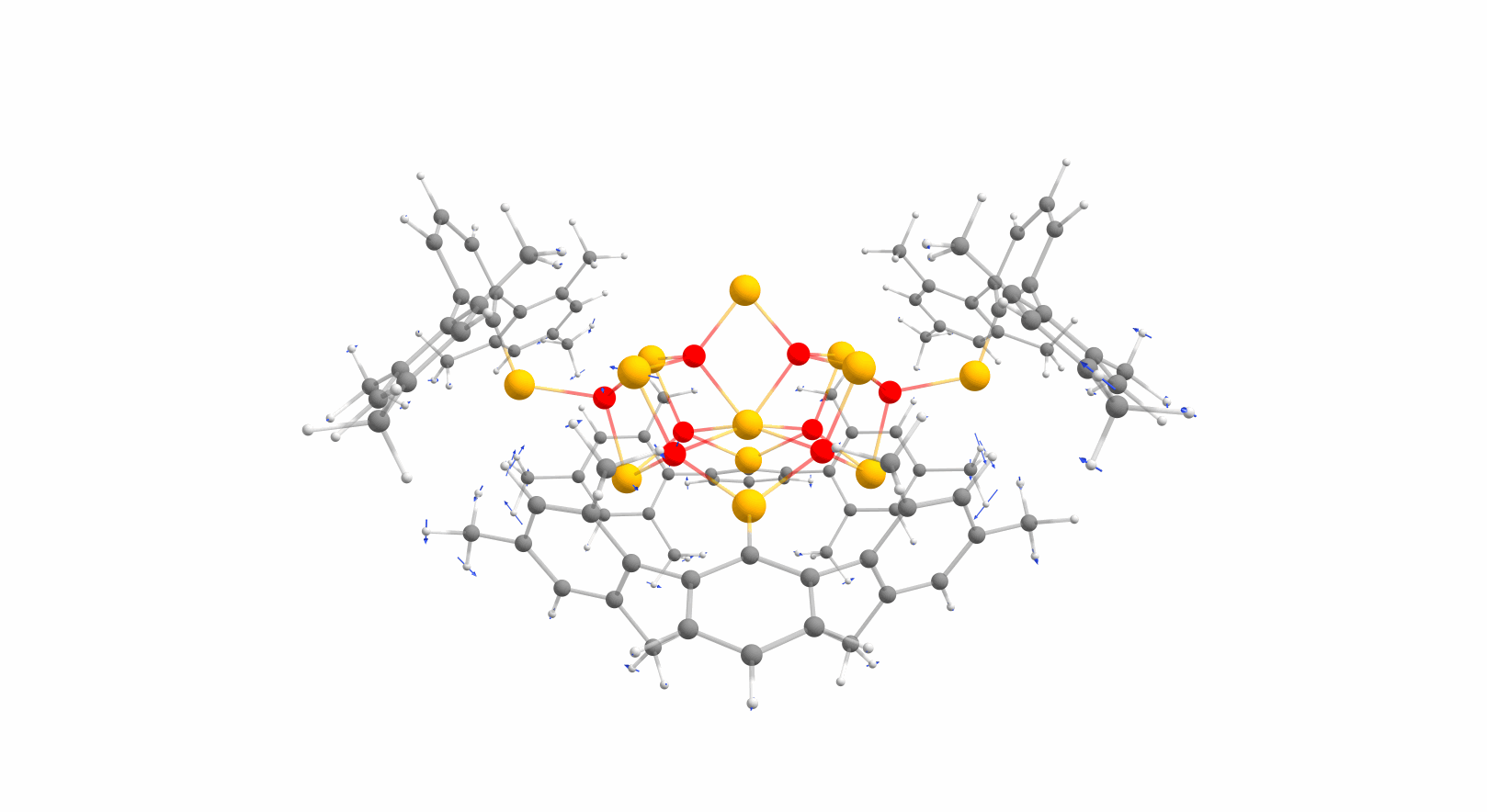

Supplement: Supplementary file 3 — GIF animations of broken-symmetry DFT-calculated normal modes of ildc. [file 41557_2025_1895_MOESM3_ESM.zip › ildc_mode#142_267cm-1.gif]

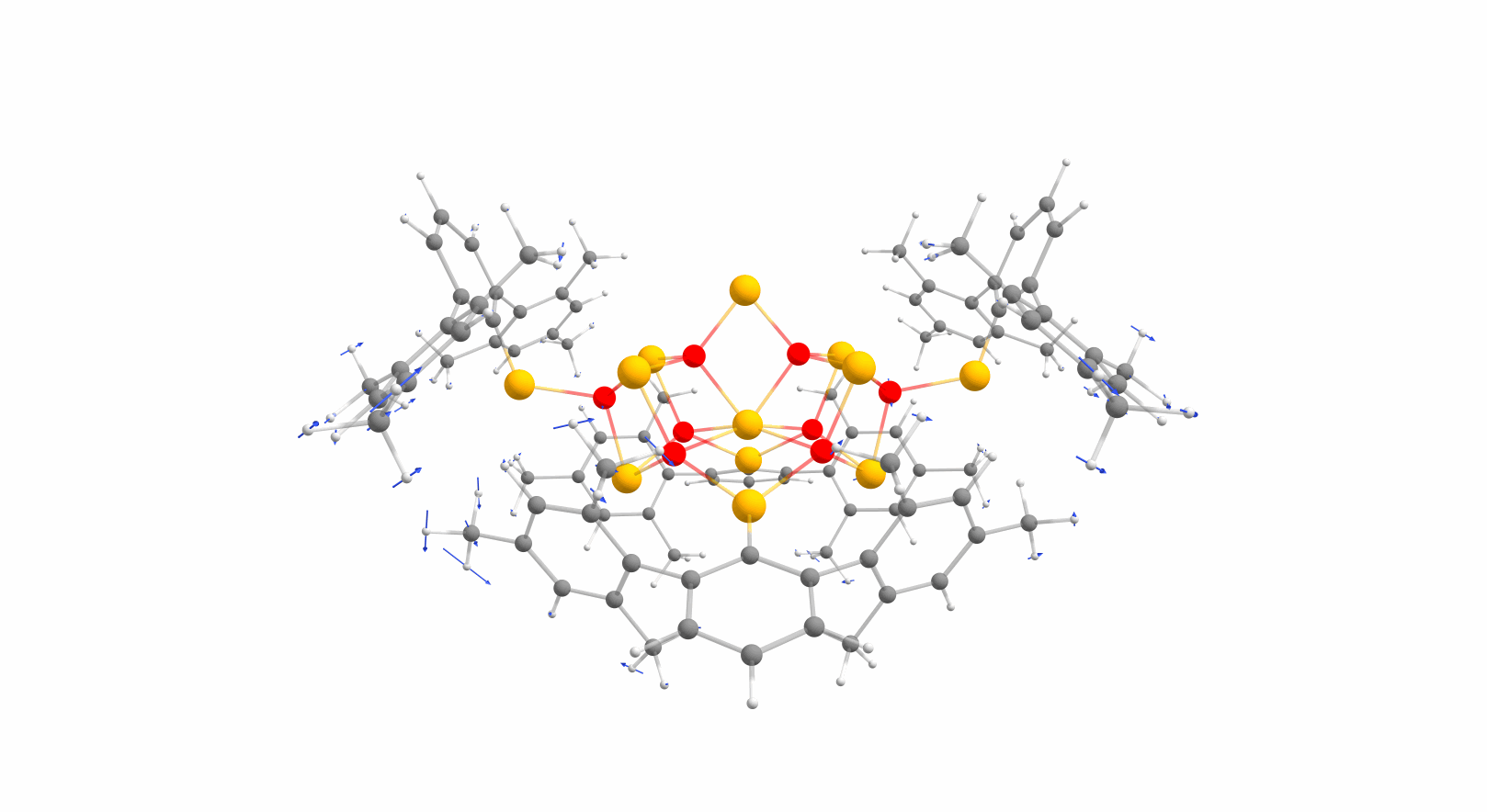

Supplement: Supplementary file 3 — GIF animations of broken-symmetry DFT-calculated normal modes of ildc. [file 41557_2025_1895_MOESM3_ESM.zip › ildc_mode#149_275cm-1.gif]

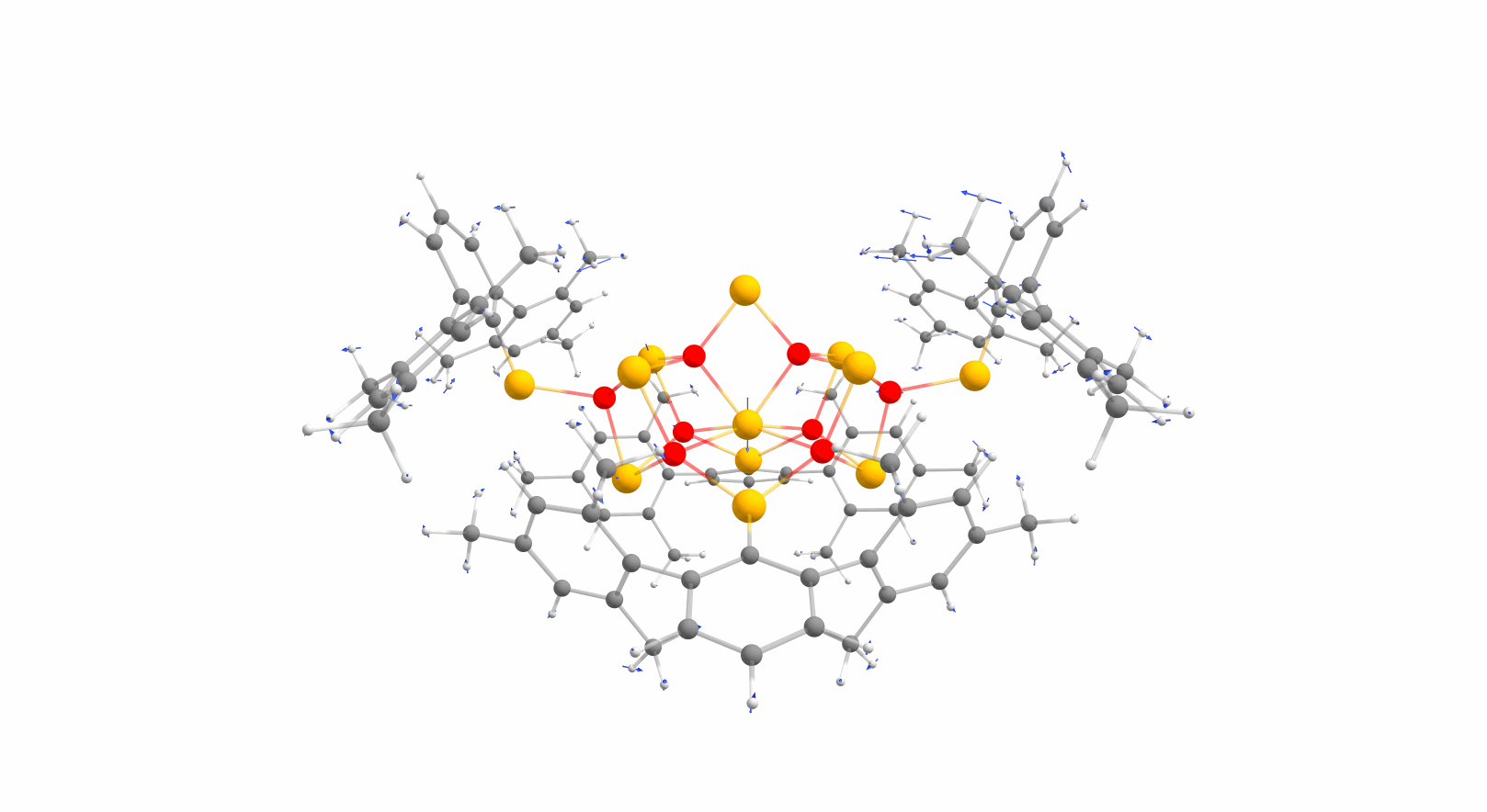

Supplement: Supplementary file 3 — GIF animations of broken-symmetry DFT-calculated normal modes of ildc. [file 41557_2025_1895_MOESM3_ESM.zip › ildc_mode#157_298cm-1.gif]

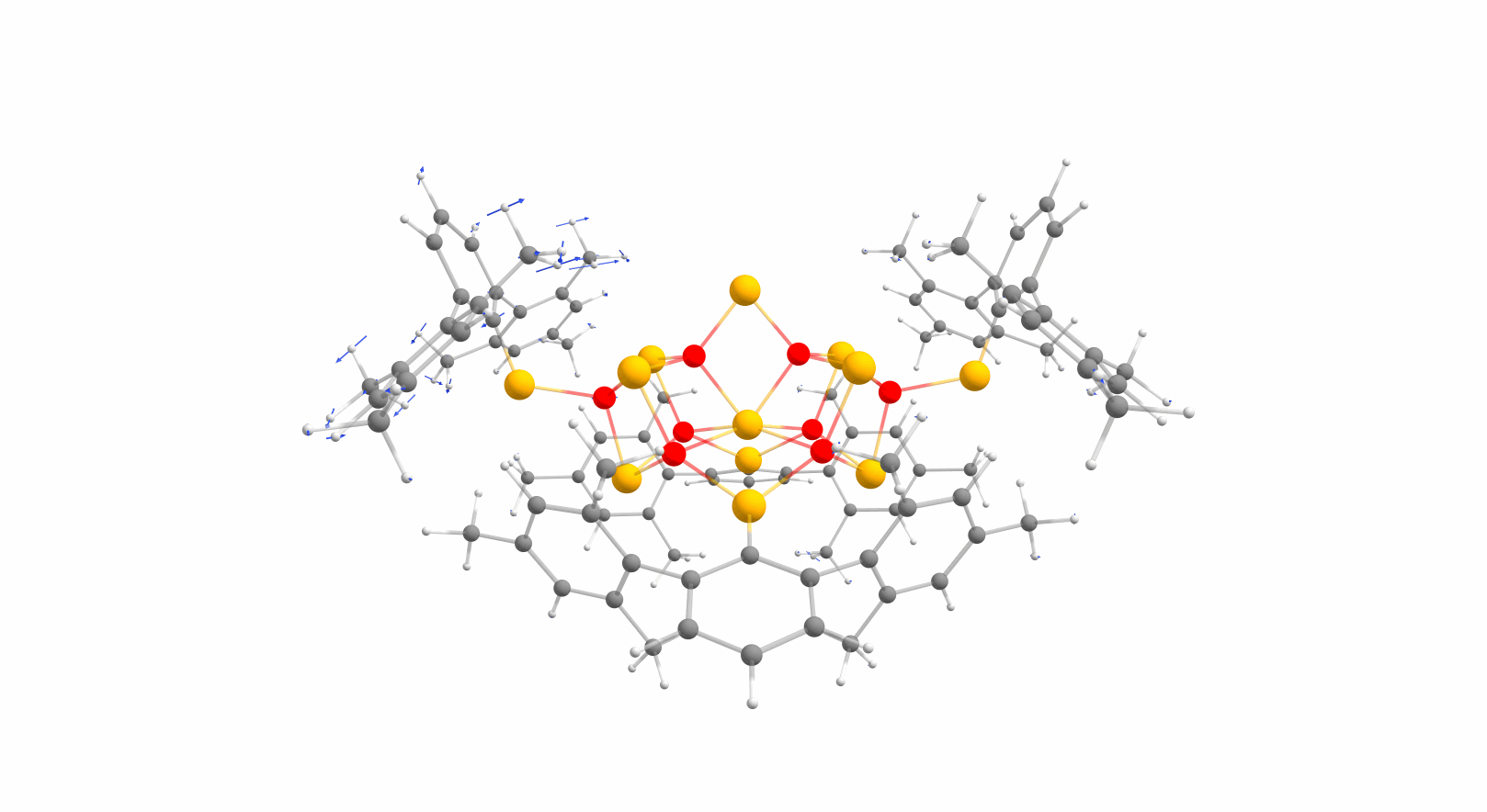

Supplement: Supplementary file 3 — GIF animations of broken-symmetry DFT-calculated normal modes of ildc. [file 41557_2025_1895_MOESM3_ESM.zip › ildc_mode#159_303cm-1.gif]
